# Discrimination of Radix Astragali from Different Growth Patterns, Origins, Species, and Growth Years by an H^1^-NMR Spectrogram of Polysaccharide Analysis Combined with Chemical Pattern Recognition and Determination of Its Polysaccharide Content and Immunological Activity

**DOI:** 10.3390/molecules28166063

**Published:** 2023-08-15

**Authors:** Yali Guo, Bing Wang, Lifei Gu, Guo Yin, Shuhong Wang, Meifang Li, Lijun Wang, Xie-An Yu, Tiejie Wang

**Affiliations:** 1 School of Pharmacy, Shenyang Pharmaceutical University, Shenyang 110016, China; yuoyaqwei2000@163.com; 2 NMPA Key Laboratory for Quality Research and Evaluation of Traditional Chinese Medicine, Shenzhen Institute for Drug Control, Shenzhen 518057, China; wangbingszyj@163.com (B.W.); liphiegu@gmail.com (L.G.); ayinguoa@126.com (G.Y.); szlimeifang@126.com (M.L.);

**Keywords:** Radix Astragali, immunomodulatory activity, polysaccharide content, chemical pattern recognition, quality evaluation

## Abstract

The fraud phenomenon is currently widespread in the traditional Chinese medicine Radix Astragali (RA) market, especially where high-quality RA is substituted with low-quality RA. In this case, focused on polysaccharides from RA, the classification models were established for discrimination of RA from different growth patterns, origins, species, and growth years. 1H Nuclear Magnetic Resonance (H^1^-NMR) was used to establish the spectroscopy of polysaccharides from RA, which were used to distinguish RA via chemical pattern recognition methods. Specifically, orthogonal partial least squares discriminant analysis (OPLS-DA) and linear discriminant analysis (LDA) were used to successfully establish the classification models for RA from different growth patterns, origins, species, and growth years. The satisfactory parameters and high accuracy of internal and external verification of each model exhibited the reliable and good prediction ability of the developed models. In addition, the polysaccharide content and immunological activity were also tested, which was evaluated by the phagocytic activity of RAW 264.7. And the result showed that growth patterns and origins significantly affected the quality of RA. However, there was no significant difference in the aspects of origins and growth years. Accordingly, the developed strategy combined with chemical information, biological activity, and multivariate statistical method can provide new insight for the quality evaluation of traditional Chinese medicine.

## 1. Introduction

Radix Astragali (RA), popularly known as “Huangqi” in China, has been used for over 2000 years [1]. It is one of the most popular herbal medicines in traditional Chinese medicine (TCM) [2]. Traditional Chinese medicine believes that RA is a remedy for “Qi deficiency” and its main effects include tonifying qi and lifting yang, promoting fluid production, and nourishing blood [3]. Clinical studies have shown that RA possesses various biological activities such as enhancing the body’s immunity, as well as anti-tumor and antivirus properties [4,5,6]. Modern pharmacological research shows that the function of invigorating qi with RA is mainly through enhancing the hematopoietic function, improving material metabolism, enhancing the immune function of the body, strengthening the heart, as well as antioxidant and other processes [7]. The main chemical components of RA include flavonoids, saponins, APS, amino acids, and other components [8,9]. Among them, flavonoids, saponins, and polysaccharides are widely recognized as active ingredients of RA [10], and their various substances such as Astragaloside IV, Astragaloside I, II, III, Calycosin-7-glucoside, Ononin, and Astragalus polysaccharides (APSs), have been proven to have strong immune regulatory activity [11]. Furthermore, APSs have been widely used as a good adjuvant therapy in immune enhancement and tumor treatment because of the multifaceted immune regulatory effects [12,13]. However, regarding APS, the most abundant component in RA [14], the content determination methods have not been included in the pharmacopoeias of various countries. In order to establish a quality evaluation method for pharmacological substances of RA, APS should also be regarded as an important indicator for evaluating the quality of RA.

The quality evaluation of RA is closely related to its source. For example, different regional environments can directly affect the activity of RA [15]. And as a perennial plant, traditional Chinese medicine recorded that the oldest specimens are the best [16], but the impact of growth years on APS has not yet been determined. Also, in recent years, due to the increasing demand in the market and the shortage of wild RA, cultivated RA often passed off as wild RA [17]. The growth patterns, origins, species, and growth years may be key factors affecting the quality of RA. To solve the problem of confusion in the market, it is urgent to conduct in-depth research on the quality of RA from different sources and establish effective quality evaluation methods. 

However, due to the difficulty in separation and preparation of APS, as well as the inability to accurately determine the sugar chain sequence and branching structure [18], there have been limitations in the application of this standard in various editions of the Chinese Pharmacopoeia. The monosaccharide composition of polysaccharides, molecular weight distribution of polysaccharides, and infrared spectroscopy of polysaccharides have been used to compare differences in RA [19,20,21,22]. However, these methods were poor at distinguishing the sources of medicinal materials with polysaccharides as the main active substances. Therefore, it is of great significance to adopt new technological means to break through the research bottleneck of polysaccharides and establish a quality evaluation method for polysaccharides.

1H Nuclear Magnetic Resonance (H^1^-NMR) technology has become the preferred method, as its chemical shift of the hydrogen signals represents the chemical environment, which reflects the spatial structural information of APS [23]. And it has the advantage of simple sample preparation, in addition to being non-selective and non-destructive [24]. In addition, chemometrics is an effective method for extracting and utilizing valuable information from complex analytical data. These mainly include principal component analysis (PCA), linear discriminant analysis (LDA), and orthogonal partial least squares discriminant analysis (OPLS-DA) [25]. H^1^-NMR-based spectroscopy combined with chemometrics has proven to be an effective method for quality control of traditional Chinese medicine. For example, Sun Lili et al. combined the H^1^-NMR fingerprints of Polygoni Multiflori Radix and PLS-DA, which could be used for distinguishing the adulteration (Cynanchi Auriculati Radix) [26]. By using NMR technology and chemometrics, Venditti A et al. successfully discriminated Mentha species grown in different origins of Algeria [27]. However, there are few reports on the use of H^1^-NMR spectra of APS combined with chemometrics to evaluate the quality of RA.

In this study, the H^1^-NMR spectrum combined with chemical pattern recognition strategies were established and the polysaccharide content and immune activity were also determined, which was used to evaluate the impact of different factors on the quality of RA. Firstly, the H^1^-NMR spectra of 109 batches of RA samples were obtained, and the key factors affecting the quality of APS were screened out by chemometrics methods. Based on the screened markers, the classification models were successfully established. In addition, the content of polysaccharides was also determined and the effect of APS on the phagocytosis of neutral red by RAW264.7 was determined to evaluate the immune regulatory activity of AR, which was further used as reference for the classification results of models.

## 2. Results and Discussion

### 2.1. Information on the Collected Herb RA

A total of 109 batches of representative RA samples from different growth patterns, origins, species, and growth years were collected from January 2021 to August 2022. All samples have been identified by Chief Pharmacist Zhang Ji, former Director of the Traditional Chinese Medicine Herbarium of the National Institutes for Food and Drug Control. The specific information of the RA samples is shown in Table 1. The voucher samples were stored in the cold sample room of the Shenzhen Institute for Drug Control.

### 2.2. Establishment of Data for Chemical Pattern Recognition 

The H^1^-NMR measurements were performed on all batches of RA samples; an example NMR spectrum is displayed in Table S2. After spectra were appropriately processed, the data matrix consisting of 109 samples and 377 variables (segmented integral values of each NMR spectrum) was obtained. The data matrix was standardized for chemical pattern recognition, which can be found in Table S1.

### 2.3. Identification and Analysis of Different Growth Patterns

**Figure 1:**
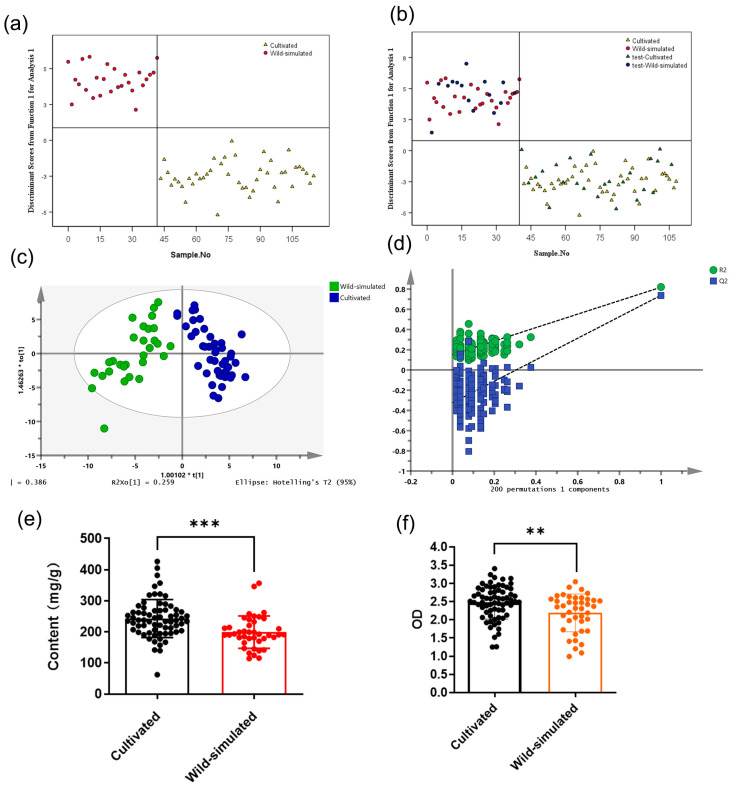
The classification models for polysaccharide content and immune regulatory activity of RA samples based on growth patterns. (**a**) LDA scores plot for training set of cultivated and wild-simulated samples. (**b**) LDA scores plot for testing set samples. (**c**) OPLS-DA scores plot for training set samples. (**d**) Permutation test result of OPLS-DA. (**e**) The polysaccharide content of cultivated and wild-simulated samples. (**f**) Effect of cultivated and wild-simulated samples’ treatment on taking neutral red of RAW 264.7 cells (** *p* < 0.01, *** *p* < 0.001).

Wild-simulated RA was recognized as a genuine medicinal herb in the industry, occupying the high-end market, with high price and short supply. Therefore, cultivated RA was often sold as wild-simulated RA because of their morphological similarity in the market [28]. This study applied two chemical pattern recognition methods, LDA and OPLS-DA, to establish accurate identification of different growth patterns of RA. LDA analysis was conducted by using IBM SPSS (Version 26.0) software. Firstly, a training set for the LDA model was constructed based on standardization data matrix of 70 × 377. Then the “Canonical Discriminant Function Coefficients” of eigenvalues (the important chemical shift) were given by the software (Table S3) and 39 batches of RA were used as unknown samples to test the model’s classification ability. The external validation of the model adopts a cross-validation method, where the classification of each case was based on the functions of all cases except for that case. The discrimination results indicated that the accurate classification rates of the original case and cross-validation were 100% (Table 2 and Figure 1a), and all 39 samples in the testing set were correctly classified into their respective categories, which was considered to be a very satisfactory LDA classification model (Figure 1b). In addition, OPLS-DA analysis was also used to successfully establish a pattern recognition model for RA. And the eigenvalues were filtered as much as possible under the premise of ensuring that the model has good parameters (Table S4). The results indicated that the model had high classification accuracy (Figure 1c) and satisfactory parameters: R^2^X was 0.714, R^2^Y was 0.819, and Q^2^ was 0.74 (Table 2). And the permutation test (200 permutations) was used to measure the robustness and prediction ability of the model. The results indicated that both R^2^ and Q^2^ on the right side were higher than all points on the left side, proving that the model had good predictability (Figure 1d). In general, the training set accuracies of LDA analysis and OPLS-DA analysis were both 100% (Table 2), indicating a strong correlation between APS and growth patterns. In addition, the 100% accuracy of their testing set indicated the ability to distinguish the growth patterns of unknown samples based on the H^1^-NMR spectrum data of APS. On the other hand, the polysaccharide content and phagocytic activity of cultivated RA samples were significantly higher than those of wild RA samples (Figure 1e,f), which proved that the growth mode significantly affected the polysaccharide composition and immunomodulatory activity of RA. These results reminded us that cultivated RA may not be inferior to wild-simulated RA and can provide reference for further research on the differences between the two and the selection of planting methods.

### 2.4. Identification and Analysis of Different Growth Years

**Figure 2:**
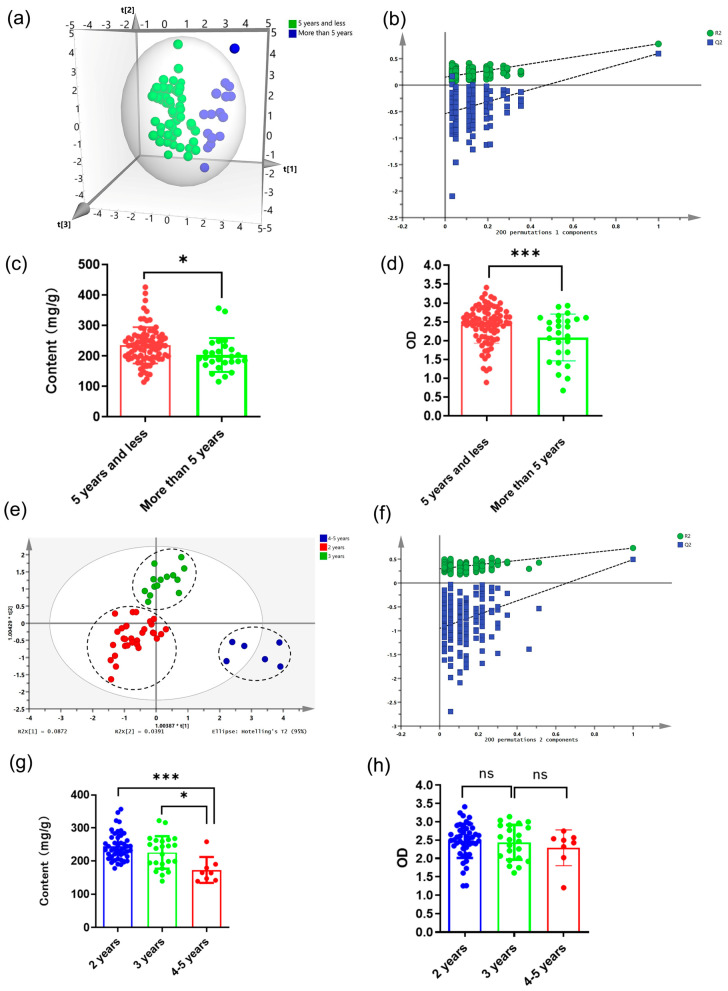
The classification models for polysaccharide content and immune regulatory activity for RA samples based on growth years. OPLS-DA scores plot for training set samples at the limit of five years (**a**) and at more accurate age of 5 years and below (**e**). Permutation test result of OPLS-DA for RA samples at the limit of five years (**b**) and at more accurate age of 5 years and below (**f**). The polysaccharide content for RA samples at the limit of five years (**c**) and at more accurate age of 5 years and below (**g**). Effect of RA samples at the limit of five years (**d**) and at more accurate age of 5 years and below (**h**). Treatment on taking neutral red of RAW 264.7 cells (* *p* < 0.05, *** *p* < 0.001, “ns” means “not significant”).

The chemical composition of RA could be affected by the gene expression and the accumulation of metabolites, which were age-related. Apart from that, the degree to which RA was affected by the external environment varies according to its age [29]. So, it was necessary to distinguish RA with different growth years. The group standard T/CACM 1021.4-2018 [30] divided medicinal materials of RA into two specifications: cultivation and wild simulation for more than 5 years [31]. Therefore, the classification between the over and within five years was first performed. OPLS-DA was used to construct a recognition model for RA samples of two types of years. The OPLS-DA model was established via the training set shown in Table 3, and the eigenvalues were shown in Table S5. The R^2^X, R^2^Y, and Q^2^ of the model (Table 3) were 0.643, 0.769, and 0.596, respectively. These values showed that the OPLS-DA model was reliable and had good prediction ability [25]. As shown in Figure 2a, there was an obvious separation between the samples of RA with two types of years, indicating a significant difference in the APS composition of them. The performance test results of the established model showed that the model was not overfitting (Figure 2b), and 92.1% of the testing set was correctly classified into their respective categories (Table 3), indicating that the model could distinguish between two main categories of years. Similarly, polysaccharide content and immunological activity of RA with two types of years showed a significant difference (Figure 2c,d), and all samples within 5 years showed a significant advantage. 

At present, low-age RA was the main species circulating in the market [28]. In order to solve the problem of difficulty in discriminating their growth years, the next model was developed to distinguish RA within 5 years. In addition, due to the small number of samples of RA aged 1.5, 2.5, and 3.5 years, RA aged 1.5 years was classified into the 2 years category, and RA aged 2.5 and 3.5 years was classified into the 3 years category. And the results showed that the OPLS-DA model was initially successful (Figure 2e,f and Table 3); the eigenvalues are given in Table S6. Subsequently, 23 batches of samples were employed to test the predictive ability of unknown samples. Table 3 showed that except for a 2-year-old RA and 3-year-old RA, almost all samples were correctly classified by the OPLS-DA model (92%). Therefore, this method was reliable and suitable for the identification of the growth years of RA samples. Consistent with these results, the polysaccharide content of RA samples in 2 years was significantly higher than that of RA samples in 4–5 years (Figure 2g). Although there was no significant difference in immunological activity of RA in the three types of years, the phagocytic activity of RAW264.7 cells treated with RA decreased with the increase in years (Figure 2h). However, traditional Chinese medicine believed that the higher the age of RA, the better. The above results proved the irrationality of this conclusion. In addition, the results reflect the rationality that 2-year-old transplanted RA had become the mainstream variety in the current RA market.

### 2.5. Identification and Analysis of Different Species

**Figure 3:**
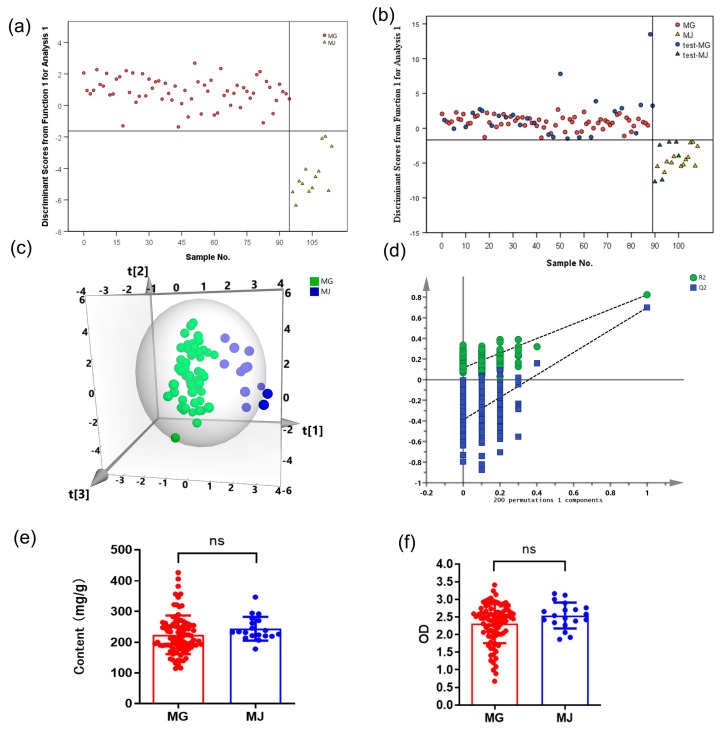
The classification models for polysaccharide content and immune regulatory activity for RA samples based on species. (**a**) LDA scores plot for training set of MG and MJ. (**b**) LDA scores plot for testing set samples. (**c**) OPLS-DA scores plot for training set samples. (**d**) Permutation test result of OPLS-DA. (**e**) The polysaccharide content of MG and MJ samples. (**f**) Effect of MG and MJ samples treatment on taking neutral red of RAW 264.7 cells (“ns” means “not significant”).

The dispute over the classification of A. membranaceus var. Mongholicus (MG) and A. membranaceus (MJ) had been going on for decades [32,33]. Because TCM syndrome differentiation played a vital role in pharmacology and clinical efficacy, it was urgent to find a feasible method to distinguish MG from MJ. The chemical pattern recognition model of MG and MJ was constructed by the eigenvalues (Table S7). The results of the LDA analysis of RA samples of two species showed that the origins of all samples were correctly classified by the model (Figure 3a,b). In addition, the OPLS-DA method was also used in an attempt to distinguish RA. It is worth noting that the close distribution of samples in the training set (Figure 3c) showed that the sample preparation method had good repeatability and the measurement accuracy of the NMR instrument was very high. The eigenvalues (Table S8) and the Y- scrambling analysis signified the effectiveness of our method (Figure 3d), and the correct classification percentage of the testing set was almost 100.0% (Table 4). Interestingly, there was no significant difference in polysaccharide content and phagocytic activity between MG and MJ (Figure 3e,f). The results suggested that although there were significant differences in polysaccharide composition between MG and MJ, this difference did not significantly affect their phagocytosis-enhancing ability. 

### 2.6. Identification and Analysis of Different Origins

**Figure 4:**
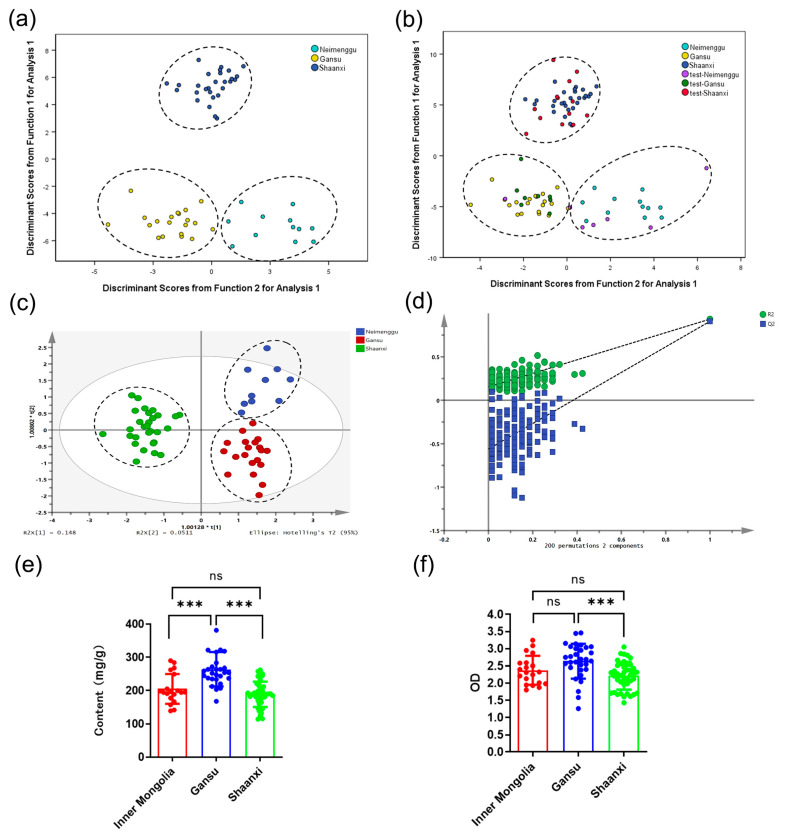
The classification models for polysaccharide content and immune regulatory activity for RA samples based on origins. (**a**) LDA scores plot for training set samples from the areas of Inner Mongolia, Gansu, and Shaanxi. (**b**) LDA scores plot for testing set samples. (**c**) OPLS-DA scores plot for training set samples. (**d**) Permutation test result of OPLS-DA. (**e**) The polysaccharide content of these three areas samples. (**f**) Effect of these three areas samples treatment on taking neutral red of RAW 264.7 cells (*** *p* < 0.001, “ns” means “not significant”).

Inner Mongolia, Gansu, Shanxi, and Shaanxi are the main producing areas of RA in China [34]. The environment of these regions varies greatly due to their geographical location. This will affect the chemical composition of AR. Therefore, RA from Inner Mongolia, Gansu, Shanxi, and Shaanxi were distinguished. In this study, the reasons for the selection of these four sites were that the number of samples was sufficient, and they were all the main producing areas of RA. However, it was not possible to distinguish the origins of RA samples from the four main origins. But interestingly, after distinguishing the species, the three origins can be distinguished (Figure 4a), which was possibly because these differences were much smaller compared to the differences caused by the origins. In order to strictly test the practical applicability of the model, we used 28 samples as the testing set (7 batches in Inner Mongolia, 9 batches in Gansu, and 12 batches in Shaanxi) to verify the coefficients of the eigenvalues (Table S9). The prediction accuracy for Inner Mongolia, Gansu, and Shaanxi regions is 71.4%, 100%, and 100%, respectively (Table 5 and Figure 4b). There were two misclassifications in the Inner Mongolia region (Figure 4b), which may be due to its small sample size. Subsequently, the OPLS-DA model was further developed to classify RA from different areas. The results evidenced that the model had good classification performance (Table S10 and Figure 4c,d). The external verification results showed that except for one sample from Inner Mongolia, all other groups were accurately classified (Table 5). The results displayed were also consistent with the results below. The content of polysaccharides in RA samples from Gansu was significantly higher than that from Inner Mongolia and Shaanxi. In addition, the RA samples from Gansu showed higher phagocytic activity than those from other regions, while there was no statistical difference between RA samples from Inner Mongolia and Shaanxi regions in these two indicators (Figure 4e,f). It can be speculated that RA in the Gansu area may have better quality. 

## 3. Materials and Methods

### 3.1. Chemicals, Reagents, and Materials

RAW 264.7 was purchased from National Collection of Authenticated Cell Cultures. Deuterium oxide (D2O) was obtained from Shanghai Macklin Biochemical CO., LTD. (Shanghai, China). Neutral Red was acquired from Sigma Aldrich (St. Louis, MO, USA). D-(+)-Glucose, Phenol were purchased from Aladdin Co., Ltd. (Shanghai, China). DMEM media (Gibco, Grand Island, NY, USA), fetal bovine serum (FBS, Gibco), Penicillin-Streptomycin (Gibco), phosphate-buffered saline (PBS, Gibco) were used for cell culture. LPS was purchased from Shanghai Biyuntian Biotechnology Co., Ltd. (Shanghai, China). All other chemicals used in this study were of analytical grade. The specific RA sample information was listed in Table 1.

### 3.2. Sample Preparation

The extraction method of APS is based on a previous method but slightly modified [35]. Briefly, each batch of powdered RA (7 g) was weighed accurately and ultrasonically (80 W) extracted with 210 mL water twice (20 min each time) at 55 °C. The supernatant mixture was filtered, concentrated under reduced pressure at 55 °C, and cooled to room temperature. Then, 5/7 of the extract was separated for the preparation of APS, while the rest was directly freeze-dried as the total extract. For the preparation of APS, anhydrous ethanol was added to the extract until a final concentration amount of 80% (*v*/*v*) for the precipitate, and it was allowed to stew for 12 h at 4 °C. Precipitates were collected by centrifugation (5000 rpm for 10 min) and subsequently freeze-dried.

### 3.3. NMR Measurements

Each APS sample was dissolved in D2O at a concentration of 20 mg/mL. After centrifuging for 10 min at 10,000 rpm, the supernatant (500 μL) was transferred into a 5 mm NMR tube for H^1^-NMR spectra. All H^1^-NMR spectra were recorded at 25 °C on a Bruker 500 instrument, and the experimental parameters were slightly modified according to previous reports [36]. The specific parameters were as follows: 128 scans; temperature, 302.4 K; time data, 32,768 points; spectral width, 8.012 kHz; delay time, 4 s.

### 3.4. Determination of the Polysaccharide Content

The phrase ‘sweet taste is better for RA’ has been recorded in various dynasties of Chinese herbal medicine [37]. Therefore, sweet taste is an important traditional indicator for evaluating the quality of RA. In this study, the polysaccharide content was used as an indicator of sweetness and was determined in all RA samples by adopting the phenol-sulfuric acid method described in Chinese Pharmacopoeia with slight modifications [3]. Briefly, 2 mL solution of the total extract with a concentration of 0.05 mg/mL was mixed with 1.0 mL of 5% phenol reagent. Then 5 mL sulfuric acid was added rapidly and mixed. The mixture was transferred to 80 °C water bath for 30 min. Then the mixture was cooled for 10 min in ice water, and the absorption values were tested at 486 nm in parallel three times. A standard curve can be obtained through different concentrations of D-glucose standard solution, and the polysaccharide content can be inferred by the standard curve.

### 3.5. Cellular Phagocytosis of Neutral Red

Firstly, a 100 μL suspension of RAW 264.7 cells was cultured in a 96-well plate at a density of 1 × 10^6^ cells/well and incubated for 24 h. Thereafter, the cells were incubated with 1μg/mL LPS (as the positive group), FBS (as the control group), or 300 μg/mL APS for 24 h. After removing the medium, 100 μL of 0.1% neutral red solution was dissolved in saline and was added to wells, and a further one-hour incubation followed. To extract the dye engulfed by macrophages, the cells were washed with cold PBS 3 times and 100 μL cell lysate was added to each well, which consists of 10% acetic acid and ethanol at a ratio of 1:1. After lysis at room temperature for one night, the absorbance was measured at 540 nm to represent phagocytosis of neutral red.

### 3.6. Data Processing

The H^1^-NMR spectra were processed using MestReNova (version 14.2.0, Mestrelab Research, Santiago, Spain). All NMR spectra were automatically phased and baseline-corrected and calibrated with the D2O signal at 4.71 ppm. The regions ranging from 1 to 1.16 and 4.6 to 4.9 ppm were removed from the NMR spectrum as residual water and interference peaks. Then, the region of δ 0.5–8.50 of spectra was automatically integrated by area with a bin width of 0.02 ppm. All integrated bins were normalized to the total integral of the spectral regions and then converted into ASCII format for further chemometric analyses.

Except NMR measurements, each experiment was performed in triplicate. Statistical difference was determined using a two-tailed Student’s *t*-test, with * *p* < 0.05, ** *p* < 0.01, and *** *p* < 0.001 indicating statistical significance.

## 4. Conclusions

In this present study, the H^1^-NMR spectrum of APS was obtained, and classification models of RA were successfully established by LDA and OPLS-DA. The results strongly demonstrated that the constructed method can serve as a powerful tool for distinguishing RA from different growth patterns, origins, species, and growth years. And the classification results were further validated by using two indicators: polysaccharide content and immune regulatory activity. Accordingly, this method achieved rapid and effective identification of RA and it was of great significance to solve the problem of mixed use of RA. More importantly, it provided a new perspective for evaluating the quality of RA by analyzing the information of APS using new technological methods.

## Figures and Tables

**Table 1:** Detailed information of 109 batches of samples.

Sample No.	Cultivation Patterns	Species	Origins (Province)	Growth Years
S1	Cultivated	*A. membranaceus* var. *Mongholicus*	Shanxi	2
S2	Cultivated	*A. membranaceus* var. *Mongholicus*	Heilongjiang	2
S3	Cultivated	*A. membranaceus* var. *Mongholicus*	Inner Mongolia	1.5
S4–S9	Cultivated	*A. membranaceus* var. *Mongholicus*	Inner Mongolia	2
S10	Cultivated	*A. membranaceus* var. *Mongholicus*	Inner Mongolia	2.5
S11–S14	Cultivated	*A. membranaceus* var. *Mongholicus*	Inner Mongolia	3
S15–S19	Cultivated	*A. membranaceus* var. *Mongholicus*	Inner Mongolia	3.5
S20	Cultivated	*A. membranaceus* var. *Mongholicus*	Inner Mongolia	4
S21–S23	Cultivated	*A. membranaceus* var. *Mongholicus*	Gansu	1
S24	Cultivated	*A. membranaceus* var. *Mongholicus*	Gansu	1.5
S25–S38	Cultivated	*A. membranaceus* var. *Mongholicus*	Gansu	2
S39–S41	Cultivated	*A. membranaceus* var. *Mongholicus*	Gansu	2.5
S42–S44	Cultivated	*A. membranaceus* var. *Mongholicus*	Gansu	3
S45–S48	Cultivated	*A. membranaceus* var. *Mongholicus*	Gansu	3.5
S49–S50	Cultivated	*A. membranaceus*	Xinjiang	2
S51–S58	Cultivated	*A. membranaceus*	Shanxi	2
S59–S60	Cultivated	*A. membranaceus*	Qinghai	2
S61–S68	Cultivated	*A. membranaceus*	Gansu	2
S69–S71	Wild-simulated	*A. membranaceus* var. *Mongholicus*	Shaanxi	1
S72–S74	Wild-simulated	*A. membranaceus* var. *Mongholicus*	Shaanxi	2
S75–S77	Wild-simulated	*A. membranaceus* var. *Mongholicus*	Shaanxi	3
S78–S81	Wild-simulated	*A. membranaceus* var. *Mongholicus*	Shaanxi	4
S82–S84	Wild-simulated	*A. membranaceus* var. *Mongholicus*	Shaanxi	5
S85–S91	Wild-simulated	*A. membranaceus* var. *Mongholicus*	Shaanxi	6
S92–S99	Wild-simulated	*A. membranaceus* var. *Mongholicus*	Shaanxi	7
S100–S104	Wild-simulated	*A. membranaceus* var. *Mongholicus*	Shaanxi	8
S105–S106	Wild-simulated	*A. membranaceus* var. *Mongholicus*	Shaanxi	9
S107–S109	Wild-simulated	*A. membranaceus* var. *Mongholicus*	Shaanxi	10

Wild-simulated: Under the artificial simulated wild mode, the seeds were directly planted and grown, usually for more than 5 years, and had some characteristics of wild RA.

**Table 2:** The classification results from the LDA and OPLS-DA models of growth patterns.

Items of Model	Categories	Number of Samples		R^2^X	R^2^Y	Q^2^	Number of Correct Classification (%)		Permutation Test (200 Permutations)	LOOCV Accuracy
		Training Set	Testing Set				Training Set	Testing Set		
LDA	Cultivated	44	24	−	−	−	70 (100%)	39 (100%)	−	100%
	Wild-simulated	26	15							
OPLS-DA	Cultivated	43	25	0.714	0.819	0.74	71 (100%)	38 (100%)	0.149	−
	Wild-simulated	28	13							

**Table 3:** The classification results from the OPLS-DA models of growth years.

Items of OPLS-DA	Categories	Number of Samples		R^2^X	R^2^Y	Q^2^	Number of Correct Classification (%)		Permutation Test (200 Permutations)
		Training Set	Testing Set				Training Set	Testing Set	
Growth years	5 years and less	55	29	0.643	0.769	0.596	71 (100%)	35 (92.1%)	0.158
	More than 5 years	16	9						
Growth years (5 years and less)	2 years	31	9	0.911	0.806	0.518	51 (100%)	23 (92%)	0.298
	3 years	14	14						
	4~5 years	6	2						

**Table 4:** The classification results from the LDA and OPLS-DA models of species.

Items of Model	Categories	Number of Samples		R^2^X	R^2^Y	Q^2^	Number of Correct Classification (%)		Permutation Test (200 Permutations)	LOOCV Accuracy
		Training Set	Testing Set				Training Set	Testing Set		
LDA	MG	64	26	−	−	−	77 (100%)	32 (100%)	−	96.1%
	MJ	13	6							
OPLS-DA	MG	60	30	0.847	0.823	0.699	72 (100%)	36 (97.3%)	0.115	−
	MJ	12	7							

**Table 5:** The classification results from the LDA and OPLS-DA models of origins.

Items of Model	Categories	Number of Samples		R^2^X	R^2^Y	Q^2^	Number of Correct Classification (%)		Permutation Test (200 Permutations)	LOOCV Accuracy
		Training Set	Testing Set				Training Set	Testing Set		
LDA	Inner Mongolia	12	7	−	−	−	61 (100%)	26 (92.9%)	−	100%
	Gansu	20	9							
	Shaanxi	29	12							
OPLS-DA	Inner Mongolia	10	9	0.805	0.808	0.685	59 (100%)	29 (96.6%)	0.154	−
	Gansu	20	9							
	Shaanxi	29	12							

## Data Availability

The data are available within this article and its Supplementary Materials.

## Supplementary Materials

The following supporting information can be downloaded at: https://www.mdpi.com/article/10.3390/molecules28166063/s1, Table S1: Results of data analysis of 377 integrated areas of 109 batch samples. Table S2. The example 1H-NMR spectrum of APS. Table S3. Canonical Discriminant Function Coefficients of LDA models of growth patterns. Table S4. Regression Coefficients of OPLS-DA models of growth patterns. Table S5. Regression Coefficients of OPLS-DA models of growth years over and within 5 years. Table S6. Regression Coefficients of OPLS-DA models of growth years within 5 years. Table S7. Canonical Discriminant Function Coefficients of LDA models of species. Table S8. Regression Coefficients of OPLS-DA models of species. Table S9. Canonical Discriminant Function Coefficients of LDA models of origins. Table S10. Regression Coefficients of OPLS-DA models of origins.

## Sample Availability

Samples of the Radix Astragali are available from the authors.
