# Supplementary material for: Discrimination of Radix Astragali from Different Growth Patterns, Origins, Species, and Growth Years by an H1-NMR Spectrogram of Polysaccharide Analysis Combined with Chemical Pattern Recognition and Determination of Its Polysaccharide Content and Immunological Activity
Source: Molecules. 2023 Aug 15;28(16):6063. doi: 10.3390/molecules28166063 (PMC10458787; doi:10.3390/molecules28166063)
Supplement: Supplementary file 1 [file molecules-28-06063-s001.zip › molecules-2539783-supplementary.pdf]

Supporting Information

Discrimination of Radix Astragali from Different Growth Patterns, Origins, Species, and Growth Years by an H¹-NMR Spectrogram of Polysaccharide Analysis Combined with Chemical Pattern Recognition and Determination of Its Polysaccharide Content and Immunological Activity

Yali Guo [1,†], Bing Wang [2,†], Lifei Gu [2,†], Guo Yin [2], Shuhong Wang [2], Meifang Li [2], Lijun Wang [2], Xie-An Yu [2,*] and Tiejie Wang [1,2,*]

[1] School of Pharmacy, Shenyang Pharmaceutical University, Shenyang 110016, China; yuoyaqwei2000@163.com

[2] NMPA Key Laboratory for Quality Research and Evaluation of Traditional Chinese Medicine, Shenzhen Institute for Drug Control, Shenzhen 518057, China; wangbingszyj@163.com (B.W.); liphiegu@gmail.com (L.G.); ayinguoa@126.com (G.Y.); szlimeifang@126.com (M.L.)

* Correspondence: yuxieananalj@126.com (X.-A.Y.); szyjwtj@163.com (T.W.)

† These authors contributed equally to this work.

Table S1. Results of data analysis of 377 integrated areas of 109 batch samples.

Sample No.	integrated areas														
	8.50 .. 8.48	8.48 .. 8.46	8.46 .. 8.44	8.44 .. 8.42	8.42 .. 8.40	8.40 .. 8.38	8.38 .. 8.36	8.36 .. 8.34	8.34 .. 8.32	8.32 .. 8.30	8.30 .. 8.28	8.28 .. 8.26	8.26 .. 8.24	8.24 .. 8.22	8.22 .. 8.20
S1	-0.14274	-0.17809	-0.19835	-0.21408	-0.3921	-0.16864	-0.25795	-0.2763	-0.15302	-0.23842	-0.23014	-0.12047	-0.20704	-0.16431	-0.2169
S2	-0.14274	-0.17809	-0.19835	-0.21408	0.39542	0.73773	-0.25795	-0.2763	-0.15302	-0.23842	-0.23014	-0.12047	-0.20704	-0.16431	-0.2169
S3	-0.14274	-0.17809	-0.19835	-0.21408	-0.3921	-0.04116	-0.25795	-0.2763	-0.15302	-0.23842	-0.23014	-0.12047	-0.20704	-0.16431	-0.2169
S4	-0.14274	-0.17809	-0.19835	-0.21408	-0.3921	-0.46398	-0.25795	-0.2763	-0.15302	-0.23842	-0.23014	-0.12047	-0.20704	-0.16431	-0.2169
S5	-0.14274	-0.17809	-0.19835	-0.21408	-0.3921	-0.46398	-0.25795	-0.2763	-0.15302	-0.23842	-0.23014	-0.12047	-0.20704	-0.16431	-0.2169
S6	-0.14274	-0.17809	-0.19835	-0.21408	-0.3921	-0.21845	-0.25795	-0.2763	-0.15302	-0.23842	-0.23014	-0.12047	-0.20704	-0.16431	-0.2169
S7	-0.14274	-0.17809	-0.19835	-0.21408	-0.3921	-0.46398	-0.25795	-0.2763	-0.15302	-0.23842	-0.23014	-0.12047	-0.20704	-0.16431	-0.2169
S8	-0.14274	-0.17809	-0.19835	-0.21408	-0.3921	-0.25453	-0.25795	-0.2763	-0.15302	-0.23842	-0.23014	-0.12047	-0.20704	-0.16431	-0.2169
S9	-0.14274	0.719	-0.12051	0.13941	-0.3921	-0.06926	-0.25795	-0.2763	-0.15302	-0.23842	-0.23014	-0.12047	0.92846	10.40439	1.01499
S10	-0.14274	3.18357	2.36576	1.56523	2.71031	0.52396	1.35374	-0.2763	0.04956	0.07683	0.30269	0.087	0.48039	0.35853	9.97927
S11	-0.14274	-0.17809	-0.19835	-0.21408	-0.3921	-0.46398	-0.25795	-0.2763	-0.15302	-0.23842	-0.23014	-0.12047	-0.20704	-0.16431	-0.2169
S12	-0.14274	-0.17809	-0.19835	-0.21408	-0.3921	-0.20533	-0.25795	-0.2763	-0.15302	-0.23842	-0.23014	-0.12047	-0.20704	-0.16431	-0.2169
S13	2.3429	0.41381	-0.19835	0.11875	-0.3921	-0.26015	-0.25795	-0.2763	-0.15302	-0.23842	8.0741	10.55722	10.0579	0.13526	-0.2169
S14	-0.14274	-0.17809	-0.19835	-0.21408	-0.3921	0.63232	-0.25795	-0.2763	-0.15302	-0.23842	-0.23014	-0.12047	0.16444	0.38019	1.29733
S15	10.04679	9.48344	8.77173	8.52007	5.36284	1.01994	4.71642	1.95526	3.9044	6.82799	4.4461	-0.12047	-0.20704	-0.16431	-0.2169
S16	-0.14274	-0.17809	-0.19835	-0.21408	-0.3921	-0.37889	-0.25795	-0.2763	-0.15302	-0.23842	-0.23014	-0.12047	-0.20704	-0.16431	-0.2169

S17	-0.14274	-0.17809	-0.19835	-0.21408	4.58005	2.51409	-0.25795	-0.2763	-0.15302	-0.23842	-0.23014	-0.12047	-0.20704	-0.16431	-0.2169
S18	-0.14274	-0.17809	1.37551	1.55602	1.471	0.77631	0.46322	-0.2763	-0.15302	-0.12258	-0.23014	-0.12047	-0.20704	-0.16431	0.16537
S19	-0.14274	-0.17809	-0.19835	-0.21408	0.06398	0.62064	-0.25795	-0.2763	-0.15302	-0.23842	-0.23014	-0.12047	-0.20704	-0.07732	-0.2169
S20	-0.14274	-0.07843	-0.18133	-0.21408	1.10328	0.69792	-0.25795	-0.2763	-0.15302	-0.23842	-0.23014	-0.12047	-0.00608	-0.16431	-0.2169
S21	-0.14274	-0.17809	-0.19835	-0.21408	-0.3921	-0.42012	-0.25795	-0.2763	-0.15302	-0.23842	-0.23014	-0.12047	-0.20704	-0.16431	-0.2169
S22	-0.14274	-0.17809	-0.19835	-0.21408	-0.3921	0.10717	-0.25795	-0.2763	-0.15302	-0.23842	-0.23014	-0.12047	-0.20704	-0.16431	-0.2169
S23	-0.14274	-0.17809	-0.19835	-0.21408	0.18248	0.08591	0.35581	0.96892	0.08676	0.53364	0.8131	0.11769	0.5115	0.40406	0.8142
S24	-0.14274	-0.17809	-0.19835	-0.21408	-0.3921	-0.46398	-0.25795	-0.2763	-0.15302	-0.23842	-0.23014	-0.12047	-0.20704	-0.16431	-0.2169
S25	-0.14274	2.65072	4.27141	4.55965	3.54769	1.28967	4.21699	5.42158	0.44402	3.32464	2.09496	0.44172	1.16046	0.40987	0.28137
S26	0.35294	0.29086	1.15303	0.42428	1.83348	0.36691	5.796	3.41915	0.35026	0.74521	1.04545	0.28341	0.68427	0.68164	0.92652
S27	-0.14274	-0.17809	-0.19835	-0.21408	-0.3921	0.0009	-0.25795	-0.2763	-0.15302	-0.23842	-0.23014	-0.12047	-0.20704	-0.16431	-0.2169
S28	-0.14274	-0.17809	-0.19835	-0.21408	-0.3921	-0.46398	-0.25795	-0.2763	-0.15302	-0.23842	-0.23014	-0.12047	-0.20704	-0.16431	-0.2169
S29	-0.14274	-0.17809	-0.19835	-0.21408	0.47524	-0.46398	-0.25795	-0.2763	-0.15302	-0.23842	-0.23014	-0.12047	-0.20704	-0.16431	-0.2169
S30	-0.14274	-0.17809	-0.19835	-0.21408	-0.3921	0.24847	-0.25795	-0.2763	-0.15302	-0.23842	-0.23014	-0.12047	-0.03864	-0.02362	-0.2169
S31	-0.14274	-0.17809	-0.19835	-0.21408	-0.14099	0.06001	-0.25795	-0.2763	-0.15302	-0.23842	-0.23014	-0.12047	-0.20704	-0.06807	-0.2169
S32	-0.14274	-0.17809	-0.19835	-0.21408	-0.3921	-0.46398	-0.25795	-0.2763	-0.15302	-0.23842	-0.23014	-0.12047	-0.20704	-0.16431	-0.2169
S33	-0.14274	-0.17809	-0.19835	-0.21408	-0.3921	0.17644	-0.25795	-0.2763	-0.15302	-0.23842	-0.23014	-0.12047	-0.20704	-0.16431	-0.2169
S34	-0.14274	-0.17809	-0.19835	-0.21408	-0.3921	-0.25504	-0.25795	-0.2763	-0.15302	-0.23842	-0.23014	-0.12047	-0.20704	-0.16431	-0.2169
S35	-0.14274	-0.17809	-0.19835	-0.21408	-0.3921	-0.38836	-0.25795	-0.2763	-0.15302	-0.23842	-0.23014	-0.12047	-0.20704	-0.16431	-0.01517
S36	-0.14274	-0.17809	-0.19835	-0.21408	-0.3921	-0.46398	-0.25795	-0.2763	-0.15302	-0.23842	-0.23014	-0.12047	-0.20704	-0.16431	-0.2169
S37	-0.14274	-0.17809	-0.19835	-0.21408	-0.3921	0.56285	-0.25795	-0.2763	-0.15302	-0.23842	-0.23014	-0.12047	-0.20704	-0.16431	-0.2169
S38	-0.14274	-0.17809	-0.19835	-0.21408	1.13188	0.77762	1.18591	1.45087	0.17344	0.50972	0.75294	0.03885	0.47604	0.25782	0.65399
S39	-0.14274	-0.17809	-0.19835	-0.21408	-0.3921	-0.26057	-0.25795	-0.2763	-0.15302	-0.23842	-0.23014	-0.12047	-0.20704	-0.16431	-0.2169
S40	-0.14274	-0.17809	-0.19835	-0.21408	-0.3921	-0.28648	-0.25795	-0.2763	-0.15302	-0.23842	0.64033	-0.12047	-0.04168	-0.16431	0.21814
S41	-0.14274	-0.17809	-0.19835	-0.21408	-0.3921	-0.3476	-0.25795	-0.2763	-0.15302	-0.23842	-0.23014	-0.12047	-0.20704	-0.16431	-0.2169
S42	0.83544	0.71299	1.29145	-0.14196	1.23257	0.13698	-0.24461	0.9239	-0.15302	-0.23842	-0.23014	-0.12047	0.23076	-0.16431	0.57125
S43	-0.14274	-0.17809	-0.19835	-0.21408	-0.3921	0.72396	-0.25795	-0.2763	-0.15302	-0.23842	-0.23014	-0.12047	-0.20704	-0.16431	-0.2169
S44	-0.14274	-0.17809	-0.19835	-0.21408	-0.3921	-0.46398	-0.25795	-0.2763	-0.15302	-0.23842	-0.23014	-0.12047	-0.20704	-0.16431	-0.2169
S45	-0.14274	-0.17809	-0.19835	-0.21408	1.19815	6.82523	-0.25795	-0.14133	-0.15302	-0.23842	-0.23014	-0.11048	-0.20704	-0.16431	-0.03255
S46	-0.14274	-0.17809	-0.19835	-0.21408	-0.3921	-0.46398	-0.25795	-0.2763	-0.15302	-0.23842	-0.23014	-0.12047	-0.20704	-0.16431	-0.2169
S47	-0.14274	-0.17809	-0.19835	-0.21408	-0.3921	-0.26863	-0.25795	-0.2763	-0.15302	-0.23842	-0.23014	-0.12047	-0.20704	-0.16431	-0.2169
S48	-0.14274	-0.17809	-0.19835	-0.21408	-0.3921	-0.46398	-0.25795	-0.2763	-0.15302	-0.23842	-0.23014	-0.12047	-0.20704	-0.16431	-0.2169
S49	-0.14274	1.50127	2.03215	2.32716	2.36433	0.47672	-0.25795	0.37828	0.09284	1.44808	1.13972	-0.12047	0.69182	-0.11233	0.11063
S50	-0.14274	-0.17809	-0.19835	-0.21408	-0.3921	0.16659	-0.25795	-0.2763	-0.15302	-0.23842	-0.23014	-0.12047	-0.20704	0.04726	0.11713
S51	-0.14274	-0.17809	-0.19835	-0.21408	1.37027	0.4429	-0.25795	-0.2763	-0.15302	-0.23842	-0.23014	-0.12047	-0.20704	-0.16431	-0.2169
S52	-0.14274	-0.17809	-0.19835	-0.21408	-0.3921	-0.46398	-0.25795	-0.2763	-0.15302	-0.23842	-0.23014	-0.12047	-0.20704	-0.16431	-0.2169
S53	-0.14274	-0.17809	-0.19835	-0.21408	1.46956	5.64602	-0.25795	-0.2763	-0.15302	-0.23842	-0.23014	-0.12047	-0.20704	-0.16431	0.22742
S54	-0.14274	-0.17809	-0.19835	-0.21408	-0.3921	0.01806	-0.25795	-0.2763	-0.15302	-0.23842	-0.23014	-0.12047	-0.20704	-0.16431	-0.2169
S55	2.12347	-0.17809	-0.19835	-0.21408	-0.3921	0.09185	-0.25795	6.43601	9.77125	5.82383	-0.23014	-0.12047	-0.20704	-0.16431	-0.2169
S56	-0.14274	-0.17809	-0.19835	-0.21408	0.88626	0.79634	1.75606	-0.2763	0.0902	0.73925	-0.23014	-0.06016	0.79582	0.57279	0.59429
S57	-0.14274	-0.17809	-0.19835	-0.21408	-0.3921	0.33482	-0.25795	-0.2763	-0.15302	-0.23842	-0.23014	-0.12047	-0.20704	-0.16431	-0.2169
S58	-0.14274	-0.17809	-0.19835	-0.21408	-0.3921	-0.08708	-0.25795	-0.2763	-0.15302	-0.23842	-0.23014	-0.12047	-0.20704	-0.16431	-0.2169
S59	-0.14274	-0.17809	-0.19835	-0.21408	-0.3921	-0.46398	-0.25795	-0.2763	-0.15302	-0.23842	-0.23014	-0.12047	-0.20704	-0.16431	-0.2169
S60	-0.14274	-0.17809	-0.13252	1.26214	2.53809	0.73529	3.05865	2.55265	0.56007	3.3608	3.78337	0.80121	1.67214	0.92372	1.72332

S61	-0.14274	-0.17809	-0.19835	-0.21408	-0.3921	-0.27639	-0.25795	-0.2763	-0.15302	-0.23842	-0.23014	-0.12047	-0.20704	-0.16431	-0.2169
S62	-0.14274	-0.17809	-0.19835	-0.21408	-0.3921	-0.46398	-0.25795	-0.2763	-0.15302	-0.23842	-0.23014	-0.12047	-0.20704	-0.16431	-0.2169
S63	-0.14274	-0.17809	-0.19835	-0.21408	1.08049	1.21712	4.16888	2.54182	0.39103	1.23222	0.84171	0.30056	0.26363	0.69318	0.92879
S64	-0.14274	-0.17809	-0.19835	-0.21408	-0.3921	0.06666	-0.25795	-0.2763	-0.15302	-0.23842	-0.23014	-0.12047	-0.20704	-0.16431	-0.2169
S65	-0.14274	-0.17809	-0.19835	-0.21408	-0.3921	-0.11786	-0.25795	-0.2763	-0.15302	-0.23842	-0.23014	-0.12047	-0.20704	-0.16431	-0.2169
S66	-0.14274	-0.17809	-0.19835	-0.21408	-0.3921	0.21299	-0.25795	1.69179	-0.15302	-0.23842	-0.23014	-0.05939	0.09331	0.43712	0.57639
S67	-0.14274	-0.17809	-0.19835	-0.21408	0.66225	2.15883	-0.25795	-0.2763	-0.15302	-0.23842	-0.23014	-0.12047	-0.20704	-0.16431	-0.2169
S68	-0.14274	-0.17809	-0.19835	-0.21408	0.55483	-0.34371	-0.25795	0.07288	-0.15302	-0.23842	-0.23014	-0.12047	-0.20704	-0.16431	-0.2169
S69	-0.14274	-0.17809	-0.19835	-0.21408	-0.3921	-0.46398	-0.25795	-0.2763	-0.15302	-0.23842	-0.23014	-0.12047	-0.20704	-0.16431	-0.2169
S70	-0.14274	-0.17809	-0.19835	-0.21408	-0.3921	-0.46398	-0.25795	-0.2763	-0.15302	-0.23842	-0.23014	-0.12047	-0.20704	-0.16431	-0.2169
S71	-0.14274	-0.17809	-0.19835	-0.21408	-0.3921	-0.46398	-0.25795	-0.2763	-0.15302	-0.23842	-0.23014	-0.12047	-0.20704	-0.16431	-0.2169
S72	-0.14274	-0.17809	-0.19835	-0.21408	-0.3921	-0.46398	-0.25795	-0.2763	-0.15302	-0.23842	-0.23014	-0.12047	-0.20704	-0.16431	-0.2169
S73	-0.14274	-0.17809	-0.19835	1.93346	-0.3921	-0.46398	-0.25795	-0.2763	-0.15302	-0.23842	-0.23014	-0.12047	-0.20704	-0.16431	-0.2169
S74	-0.14274	-0.17809	-0.19835	-0.21408	-0.3921	-0.01227	-0.25795	0.23492	-0.15302	-0.18044	-0.23014	0.01098	0.33174	0.35269	0.45291
S75	-0.14274	-0.17809	-0.19835	-0.21408	-0.3921	-0.46398	-0.25795	-0.2763	-0.15302	-0.23842	-0.23014	-0.12047	-0.20704	-0.16431	-0.2169
S76	-0.14274	-0.17809	-0.19835	-0.21408	-0.3921	-0.46398	-0.25795	-0.2763	-0.15302	-0.23842	-0.23014	-0.12047	-0.20704	-0.16431	-0.2169
S77	-0.14274	-0.17809	-0.19835	-0.21408	-0.3921	-0.46398	-0.25795	-0.2763	-0.15302	-0.23842	-0.23014	-0.12047	-0.20704	-0.16431	-0.2169
S78	-0.14274	-0.17809	-0.19835	-0.21408	-0.3921	-0.46398	-0.25795	-0.2763	-0.15302	-0.23842	-0.23014	-0.12047	-0.20704	-0.16431	-0.2169
S79	-0.14274	-0.17809	-0.19835	-0.21408	-0.3921	-0.46398	-0.25795	-0.2763	-0.15302	-0.23842	-0.23014	-0.12047	-0.20704	-0.16431	-0.2169
S80	-0.14274	-0.17809	-0.19835	-0.21408	-0.3921	-0.46398	-0.25795	-0.2763	-0.15302	-0.23842	-0.23014	-0.12047	-0.20704	-0.16431	-0.2169
S81	-0.14274	-0.17809	-0.19835	-0.21408	-0.3921	-0.46398	-0.25795	-0.2763	-0.15302	-0.23842	-0.23014	-0.12047	-0.20704	-0.16431	-0.2169
S82	-0.14274	-0.17809	-0.19835	-0.21408	-0.3921	-0.46398	-0.25795	-0.2763	-0.15302	-0.23842	-0.23014	-0.12047	-0.20704	-0.16431	-0.2169
S83	-0.14274	-0.17809	-0.19835	-0.21408	-0.3921	-0.46398	-0.25795	-0.2763	-0.15302	-0.23842	-0.23014	-0.12047	-0.20704	-0.16431	-0.2169
S84	-0.14274	-0.17809	-0.19835	-0.21408	-0.3921	-0.46398	-0.25795	-0.2763	-0.15302	-0.23842	-0.23014	-0.12047	-0.20704	-0.16431	-0.2169
S85	-0.14274	-0.17809	-0.19835	-0.21408	-0.3921	-0.46398	-0.25795	-0.2763	-0.15302	-0.23842	-0.23014	-0.12047	-0.20704	-0.16431	-0.2169
S86	-0.14274	-0.17809	-0.19835	-0.21408	-0.3921	-0.46398	-0.25795	-0.2763	-0.15302	-0.23842	-0.23014	-0.12047	-0.20704	-0.16431	-0.2169
S87	-0.14274	-0.17809	-0.19835	-0.21408	-0.3921	-0.46398	-0.25795	-0.2763	-0.15302	-0.23842	-0.23014	-0.12047	-0.20704	-0.16431	-0.2169
S88	-0.14274	-0.17809	-0.19835	-0.21408	-0.3921	-0.46398	-0.25795	-0.2763	-0.15302	-0.23842	-0.23014	-0.12047	-0.20704	-0.16431	-0.2169
S89	-0.14274	-0.17809	-0.19835	-0.21408	-0.3921	-0.29457	-0.25795	-0.2763	-0.15302	-0.23842	-0.23014	-0.12047	-0.20704	-0.16431	-0.2169
S90	-0.14274	-0.17809	-0.19835	-0.21408	-0.3921	-0.46398	-0.25795	-0.2763	-0.15302	-0.23842	-0.23014	-0.12047	-0.20704	-0.16431	-0.2169
S91	-0.14274	-0.17809	-0.19835	-0.21408	-0.3921	-0.46398	-0.25795	-0.2763	-0.15302	-0.23842	-0.23014	-0.12047	-0.20704	-0.16431	-0.2169
S92	-0.14274	-0.17809	-0.19835	-0.21408	-0.3921	-0.46398	-0.25795	-0.2763	-0.15302	-0.23842	-0.23014	-0.12047	-0.20704	-0.16431	-0.2169
S93	-0.14274	-0.17809	-0.19835	-0.21408	-0.3921	-0.46398	-0.25795	-0.2763	-0.15302	-0.23842	-0.23014	-0.12047	-0.20704	-0.16431	-0.2169
S94	-0.14274	-0.17809	-0.19835	-0.21408	-0.3921	-0.46398	-0.25795	-0.2763	-0.15302	-0.23842	-0.23014	-0.12047	-0.20704	-0.16431	-0.2169
S95	-0.14274	-0.17809	-0.19835	-0.21408	-0.3921	-0.46398	-0.25795	-0.2763	-0.15302	-0.23842	-0.23014	-0.12047	-0.20704	-0.16431	-0.2169
S96	-0.14274	-0.17809	-0.19835	-0.21408	-0.3921	-0.46398	-0.25795	-0.2763	-0.15302	-0.23842	-0.23014	-0.12047	-0.20704	-0.16431	-0.2169
S97	-0.14274	-0.17809	-0.19835	-0.21408	-0.3921	-0.46398	-0.25795	-0.2763	-0.15302	-0.23842	-0.23014	-0.12047	-0.20704	-0.16431	-0.2169
S98	-0.14274	-0.17809	-0.19835	-0.21408	-0.3921	-0.46398	-0.25795	-0.2763	-0.15302	-0.23842	-0.23014	-0.12047	-0.20704	-0.16431	-0.2169
S99	-0.14274	-0.17809	-0.19835	-0.21408	-0.3921	-0.46398	-0.25795	-0.2763	-0.15302	-0.23842	-0.23014	-0.12047	-0.20704	-0.16431	-0.2169
S100	-0.14274	-0.17809	-0.19835	-0.21408	-0.3921	-0.46398	-0.25795	-0.2763	-0.15302	-0.23842	-0.23014	-0.12047	-0.20704	-0.16431	-0.2169
S101	-0.14274	-0.17809	-0.19835	-0.21408	-0.3921	-0.46398	-0.25795	-0.2763	-0.15302	-0.23842	-0.23014	-0.12047	-0.20704	-0.16431	-0.2169
S102	-0.14274	-0.17809	-0.19835	-0.21408	-0.3921	-0.46398	-0.25795	-0.2763	-0.15302	-0.23842	-0.23014	-0.12047	-0.20704	-0.16431	-0.2169
S103	-0.14274	-0.17809	-0.19835	-0.21408	-0.3921	0.14535	-0.25795	-0.2763	-0.15302	-0.23842	-0.23014	-0.12047	-0.20704	-0.16431	-0.2169
S104	-0.14274	-0.17809	-0.19835	-0.21408	-0.3921	-0.46398	-0.25795	-0.2763	-0.15302	-0.23842	-0.23014	-0.12047	-0.20704	-0.16431	-0.2169

S105	-0.14274	-0.17809	-0.19835	-0.21408	-0.3921	-0.46398	-0.25795	-0.2763	-0.15302	-0.23842	-0.23014	-0.12047	-0.20704	-0.16431	-0.2169
S106	-0.14274	-0.17809	-0.19835	-0.21408	-0.3921	-0.46398	-0.25795	-0.2763	-0.15302	-0.23842	-0.23014	-0.12047	-0.20704	-0.16431	-0.2169
S107	-0.14274	-0.17809	-0.19835	-0.21408	-0.3921	-0.46398	-0.25795	-0.2763	-0.15302	-0.23842	-0.23014	-0.12047	-0.20704	-0.16431	-0.2169
S108	-0.14274	-0.17809	-0.19835	-0.21408	-0.3921	-0.46398	-0.25795	-0.2763	-0.15302	-0.23842	-0.23014	-0.12047	-0.20704	-0.16431	-0.2169
S109	-0.14274	-0.17809	-0.19835	-0.21408	-0.3921	-0.46398	-0.25795	-0.2763	-0.15302	-0.23842	-0.23014	-0.12047	-0.20704	-0.16431	-0.2169

Sample No.	integrated bins														
	8.20 .. 8.18	8.18 .. 8.16	8.16 .. 8.14	8.14 .. 8.12	8.12 .. 8.10	8.10 .. 8.08	8.08 .. 8.06	8.06 .. 8.04	8.04 .. 8.02	8.02 .. 8.00	8.00 .. 7.98	7.98 .. 7.96	7.96 .. 7.94	7.94 .. 7.92	7.92 .. 7.90
S1	-0.32636	-0.20033	-0.27468	-0.42227	-0.282	-0.30282	-0.23131	-0.30772	-0.33458	2.12303	1.24926	-0.31159	-0.44426	-0.45817	-0.50846
S2	0.9067	0.00616	0.02747	0.37353	4.64068	4.3463	-0.11057	-0.16143	-0.03492	-0.01992	-0.17571	0.31824	0.40478	0.15044	0.06937
S3	-0.32636	-0.20033	-0.27468	-0.38134	-0.282	6.34277	0.05797	-0.30772	-0.33458	-0.32257	-0.29824	-0.31159	-0.06092	-0.45817	-0.50846
S4	-0.32636	-0.20033	-0.27468	-0.17227	-0.282	-0.30282	-0.23131	-0.30772	-0.33458	0.00649	-0.29824	-0.31159	-0.44426	-0.45817	-0.50846
S5	-0.32636	-0.20033	-0.27468	-0.42227	-0.282	-0.30282	-0.23131	-0.28318	-0.20577	-0.20386	0.05611	4.86764	6.93642	-0.45817	-0.50846
S6	-0.32636	-0.20033	-0.27468	-0.2414	-0.282	-0.30282	-0.23131	-0.30772	-0.31736	-0.32257	-0.29824	0.0947	3.55976	-0.45817	-0.50846
S7	-0.32636	-0.20033	-0.2736	-0.39463	-0.282	-0.30282	-0.23131	-0.30772	-0.25942	-0.32257	-0.29824	-0.31159	-0.35414	-0.43131	1.06995
S8	-0.09592	-0.20033	-0.27468	-0.23472	-0.282	-0.30282	0.00935	3.30766	-0.33458	-0.32257	-0.29824	-0.31159	0.4682	-0.45817	-0.50846
S9	-0.32636	-0.20033	-0.27468	-0.42227	-0.282	-0.30282	-0.23131	-0.30772	-0.33458	-0.32257	-0.29824	-0.31159	-0.44426	-0.45817	-0.50846
S10	9.06127	0.19716	0.12003	-0.07989	-0.12902	-0.00675	-0.06212	0.00166	0.01091	0.24633	0.05959	0.59001	0.52488	0.62683	0.10767
S11	-0.32636	-0.20033	-0.27468	-0.42227	-0.282	-0.30282	-0.23131	-0.30772	-0.33458	-0.32257	-0.29824	-0.31159	-0.44426	-0.45817	-0.50846
S12	-0.32636	-0.20033	-0.27468	-0.42227	-0.282	-0.30282	-0.23131	-0.30772	-0.33458	-0.32257	-0.29824	0.76854	1.57553	-0.45817	-0.50846
S13	-0.32636	-0.20033	-0.27468	-0.38165	-0.282	-0.30282	-0.23131	-0.30772	-0.33458	-0.32257	-0.29824	-0.31159	-0.44426	0.85725	-0.50846
S14	0.6464	-0.10543	-0.23318	0.03634	-0.2692	-0.15022	-0.18127	-0.09534	-0.04315	-0.11663	-0.05662	0.424	0.30725	-0.10979	0.30945
S15	0.06111	-0.20033	-0.27468	-0.33855	-0.282	-0.30282	-0.23131	-0.30772	-0.33458	-0.32257	-0.29824	-0.31159	-0.44426	-0.45817	-0.50846
S16	-0.17748	-0.20033	-0.27468	-0.22544	-0.282	-0.30282	-0.23131	1.82571	1.66682	-0.21493	-0.29824	-0.31159	-0.27706	-0.43421	0.4073
S17	-0.32636	-0.20033	0.40233	3.9244	2.27489	-0.30282	-0.23131	-0.30772	-0.33458	-0.32257	-0.29824	-0.31159	-0.44426	0.20056	-0.50846
S18	2.29675	7.51708	-0.27468	-0.17622	-0.282	-0.30282	-0.23131	-0.30772	-0.33458	-0.32257	-0.29824	-0.31159	-0.44426	1.5905	-0.1381
S19	0.18366	0.37481	1.4532	5.5976	-0.11992	-0.30282	-0.23131	-0.27551	-0.16721	0.05761	0.00703	0.2485	-0.17375	1.29516	1.67975
S20	0.03228	-0.02348	7.6906	1.01744	-0.282	-0.30282	-0.23131	-0.30772	-0.33458	-0.32257	-0.29824	-0.31159	-0.0279	-0.45817	-0.50846
S21	1.00636	3.86191	-0.27468	-0.42227	-0.282	-0.30282	-0.23131	-0.30772	-0.33458	-0.32257	-0.29824	-0.31159	-0.44426	-0.45817	-0.50846
S22	0.10706	-0.20033	-0.26552	-0.23161	-0.282	-0.1875	-0.23131	-0.30772	-0.31309	-0.22778	-0.05669	0.24019	0.03475	-0.10857	-0.03934
S23	0.49298	0.0485	0.23666	0.05666	0.06863	0.0595	-0.11231	-0.00746	0.05113	0.49352	3.04207	0.89617	-0.13076	0.14634	0.66636
S24	-0.32636	-0.20033	-0.27468	-0.42227	-0.282	-0.30282	-0.23131	-0.30772	-0.33458	-0.32257	-0.29824	-0.31159	-0.0753	6.40174	0.59991
S25	0.41939	0.06701	4.60104	3.2506	0.51134	0.47799	0.00996	0.10619	0.0773	0.36598	-0.07543	0.27563	0.22425	1.15066	0.65182
S26	0.47375	0.13001	0.28396	0.09032	0.08632	-0.11592	-0.15029	-0.02611	5.5963	1.0663	0.35624	0.64315	-0.15618	0.07041	2.54108
S27	0.26646	-0.20033	0.40715	2.52617	-0.282	-0.30282	-0.23131	-0.30772	-0.2008	-0.32257	-0.29824	-0.31159	0.22582	0.26506	-0.50846
S28	-0.32636	-0.20033	-0.27468	-0.11906	3.13867	2.83617	-0.23131	-0.30772	-0.33458	-0.32257	-0.29824	-0.31159	-0.08928	-0.45817	-0.50846
S29	-0.2206	-0.20033	-0.27468	-0.25317	-0.10265	1.71228	7.41698	-0.30772	-0.33458	-0.32257	-0.29824	-0.31159	0.0011	-0.45817	-0.50846
S30	0.07075	-0.20033	-0.20691	0.12448	-0.11995	-0.11803	2.19462	4.55288	-0.1457	-0.25021	-0.29824	-0.31159	0.70164	-0.32168	-0.50846
S31	-0.32636	-0.20033	0.03885	-0.1249	-0.2519	0.17075	4.18666	0.17841	-0.14219	-0.23338	-0.29824	-0.31159	0.24589	-0.45817	-0.50846
S32	-0.32636	-0.20033	-0.27468	-0.42227	-0.282	-0.30282	-0.23131	0.5747	6.36531	-0.32257	-0.29824	-0.31159	-0.44426	-0.45817	-0.50846
S33	-0.32636	-0.20033	-0.27468	-0.42227	-0.282	-0.30282	-0.23131	-0.30772	-0.02099	2.57329	-0.29824	-0.31159	-0.44426	-0.45817	-0.47591
S34	-0.20757	-0.20033	-0.27468	-0.25185	-0.282	-0.30282	-0.23131	1.57498	0.98054	-0.12414	-0.29824	-0.31159	-0.44426	0.09035	0.27002
S35	-0.01593	-0.20033	-0.27468	-0.36951	-0.282	0.47676	4.92794	0.12815	-0.16853	-0.32257	-0.29824	-0.31159	0.45306	-0.45817	-0.50846
S36	-0.32636	-0.20033	-0.27468	-0.42112	-0.282	-0.30282	1.94388	1.43257	-0.33458	-0.32257	-0.29824	-0.31159	-0.17739	-0.45817	-0.50846

S37	0.17384	-0.19552	-0.07264	-0.18928	-0.282	-0.30282	-0.23131	-0.30772	-0.32223	-0.24071	-0.12106	-0.31159	-0.17682	-0.45817	-0.26311
S38	0.72888	0.04264	0.04159	0.04503	-0.15777	-0.07688	-0.14883	-0.14658	0.21477	3.72805	0.41831	0.92316	0.56605	0.36723	0.20851
S39	-0.32636	-0.20033	-0.27468	-0.42227	-0.282	-0.30282	-0.23131	-0.30772	-0.33458	-0.32257	-0.29824	-0.31159	0.10616	0.29528	-0.50846
S40	0.52435	0.80117	4.73029	-0.42227	-0.282	-0.30282	-0.23131	-0.26802	-0.05324	-0.12203	-0.29824	-0.09903	0.29529	-0.26256	-0.47027
S41	-0.06981	-0.20033	1.11871	3.33347	-0.282	-0.30282	-0.23131	-0.30772	-0.01094	-0.06134	-0.29824	-0.31159	0.67564	-0.44851	-0.50846
S42	1.23944	6.14814	0.2203	-0.23498	-0.282	-0.30282	-0.22701	-0.30772	0.00936	-0.16298	-0.29824	-0.1696	0.47167	0.07478	-0.43812
S43	-0.32636	-0.20033	-0.27468	-0.42227	-0.282	-0.30282	-0.23131	-0.30772	-0.33458	-0.32257	-0.29824	-0.31159	-0.44426	-0.45817	-0.50846
S44	-0.32636	-0.20033	-0.27468	-0.42227	-0.282	-0.30282	-0.23131	-0.30772	-0.33458	-0.28363	-0.29824	-0.31159	-0.44426	-0.45817	-0.50846
S45	0.4214	-0.19202	-0.13264	-0.02475	-0.11696	-0.23659	-0.20876	-0.30772	-0.25072	-0.28965	-0.1752	-0.27098	-0.44426	-0.45817	0.11758
S46	-0.32636	-0.20033	-0.0857	3.24348	0.20852	-0.30282	-0.23131	-0.30772	-0.09405	-0.22793	-0.29824	-0.31159	0.01504	0.11181	-0.50846
S47	-0.32636	-0.20033	-0.26397	-0.24077	3.98089	2.50644	-0.23131	-0.30772	-0.23968	-0.28227	-0.29824	-0.31159	0.15574	-0.45817	-0.50846
S48	-0.32636	-0.20033	-0.27468	-0.34359	2.51233	2.83543	-0.23131	-0.30772	-0.33458	-0.32257	-0.29824	-0.31159	-0.44426	0.65759	-0.50846
S49	-0.31698	-0.19617	-0.20893	0.04163	3.49731	4.35176	-0.19384	-0.2431	-0.1829	-0.25285	-0.29824	-0.31159	0.04903	-0.08532	-0.4687
S50	-0.25128	-0.20033	0.0391	-0.13626	-0.282	-0.23103	-0.17352	-0.12504	-0.04271	-0.02067	-0.18805	0.44445	0.46598	3.52607	2.9353
S51	-0.32636	-0.20033	-0.18125	0.25063	5.94099	1.20921	-0.23131	-0.30772	-0.33458	-0.32257	-0.29824	-0.31159	0.21427	-0.25756	-0.50846
S52	-0.32636	-0.20033	-0.27468	-0.42227	-0.282	-0.30282	-0.23131	-0.30772	-0.33458	-0.22539	6.26338	-0.31159	-0.44426	-0.45817	0.19785
S53	-0.32636	-0.20033	0.15615	0.13727	-0.282	-0.30282	-0.23131	-0.30772	-0.19878	-0.07185	2.20081	6.73516	-0.44426	-0.45817	2.20046
S54	-0.32636	-0.20033	-0.27468	-0.25973	-0.282	-0.30282	-0.23131	-0.30772	-0.29477	0.033	4.31989	-0.31159	-0.44426	-0.45817	1.00057
S55	-0.32636	-0.20033	-0.27468	-0.42227	-0.282	-0.30282	-0.23131	-0.30772	-0.33458	-0.32257	-0.29824	-0.31159	-0.44426	1.65956	-0.50846
S56	0.42907	0.017	0.15274	-0.07814	-0.09202	-0.08979	-0.01876	0.02406	0.74247	4.93427	0.19931	0.22063	-0.17221	-0.15312	1.02888
S57	-0.23648	-0.20033	-0.13196	-0.0913	-0.282	-0.30282	-0.23131	-0.30772	-0.26217	0.03612	4.8761	-0.31159	-0.44426	-0.45817	1.20306
S58	-0.32636	-0.20033	-0.27468	-0.42227	-0.282	-0.30282	-0.23131	-0.30772	-0.33458	-0.32257	2.66451	-0.31159	-0.44426	-0.45817	-0.33346
S59	-0.32636	-0.20033	-0.27468	-0.42227	-0.282	-0.30282	-0.23131	-0.30772	-0.33458	-0.32257	-0.29824	-0.31159	-0.44426	2.78964	1.08294
S60	1.74579	0.25135	0.61916	0.55289	0.05065	0.4343	-0.0042	6.0557	2.31259	1.53791	0.86655	2.65482	0.33247	1.27103	4.115
S61	-0.32636	-0.20033	-0.27468	-0.4183	-0.282	-0.30282	-0.23131	-0.30772	-0.33458	-0.32257	-0.29824	-0.31159	3.59166	1.53684	1.19891
S62	-0.32636	-0.20033	0.37634	2.16039	-0.282	-0.30282	-0.23131	-0.30772	-0.33458	-0.32257	-0.29824	-0.31159	-0.27257	-0.45817	-0.50846
S63	0.08227	0.04411	0.49871	0.15427	-0.11804	-0.00724	-0.13112	-0.25308	1.94657	4.42651	0.5126	0.41397	-0.028	0.18922	2.3009
S64	0.31759	-0.20033	-0.27468	-0.19972	-0.282	-0.30282	-0.18106	-0.25386	1.19873	5.64247	-0.29824	-0.31159	-0.31814	-0.45817	0.38971
S65	-0.32636	-0.20033	-0.27468	-0.42227	-0.282	-0.30282	-0.0902	2.80809	-0.33458	-0.32257	-0.29824	-0.31159	-0.44426	-0.45817	-0.37291
S66	0.14826	-0.07612	-0.06707	0.02066	-0.14855	0.3603	-0.09347	0.06289	2.85724	0.45334	-0.06989	0.50663	-0.23113	0.2175	1.9885
S67	0.13805	-0.20033	-0.21272	-0.05811	-0.282	-0.30282	-0.23131	-0.30772	-0.33458	-0.32257	-0.29824	-0.12217	1.42201	-0.45817	0.1305
S68	-0.13473	-0.20033	0.51019	2.76548	-0.282	-0.30282	-0.23131	-0.30772	-0.32237	-0.14013	-0.29824	-0.31159	-0.44426	0.86109	-0.35187
S69	-0.32636	-0.20033	-0.27468	-0.38554	-0.282	-0.30282	-0.23131	-0.30772	-0.33458	-0.32257	-0.29824	-0.31159	-0.35255	-0.45817	-0.50846
S70	-0.32636	-0.20033	-0.27468	-0.42227	-0.282	-0.30282	-0.23131	-0.30772	-0.33458	-0.32257	-0.29824	-0.31159	-0.44426	-0.45817	-0.50846
S71	-0.32636	-0.20033	-0.27468	-0.42227	-0.282	-0.30282	-0.23131	-0.30772	-0.33458	-0.32257	-0.29824	-0.31159	-0.44426	-0.45817	0.85819
S72	-0.32636	-0.20033	-0.27468	-0.42227	-0.282	-0.30282	-0.23131	-0.30772	-0.33458	-0.32257	-0.29824	-0.31159	0.31623	2.94255	-0.50846
S73	1.25113	-0.20033	-0.04335	0.67209	-0.282	-0.30282	-0.23131	-0.30772	-0.33458	-0.32257	-0.29824	-0.31159	-0.28233	-0.45817	-0.50846
S74	0.13359	-0.05246	-0.12957	-0.01903	-0.06056	-0.014	-0.07568	-0.23139	0.06257	0.00725	0.01516	0.03441	0.04821	0.92883	3.65704
S75	-0.32636	-0.20033	-0.27468	-0.42227	-0.282	-0.30282	-0.23131	-0.30772	-0.33458	-0.32257	-0.29824	-0.31159	-0.44426	-0.45817	-0.50846
S76	-0.32636	-0.20033	-0.27468	-0.42227	-0.282	-0.30282	-0.23131	-0.30772	-0.33458	-0.32257	-0.29824	-0.279	-0.44426	-0.45817	-0.50846
S77	-0.32636	-0.20033	-0.27468	-0.42227	-0.282	-0.30282	-0.23131	-0.30772	-0.33458	-0.32257	-0.29824	-0.31159	-0.44426	-0.45817	0.77369
S78	-0.32636	-0.20033	-0.27468	-0.42227	-0.282	-0.30282	-0.23131	-0.30772	-0.33458	-0.32257	-0.29824	-0.31159	-0.44426	-0.45817	-0.50846
S79	-0.32636	-0.20033	-0.27468	-0.42227	-0.282	-0.30282	-0.23131	-0.30772	-0.29252	-0.32257	-0.29824	-0.20628	0.71898	3.22172	-0.50846
S80	-0.32636	-0.20033	-0.27468	-0.33861	-0.282	-0.30282	-0.23131	-0.30772	-0.33458	-0.32257	0.40552	-0.31159	-0.20248	-0.45817	-0.50846

S81	-0.32636	-0.20033	-0.27468	-0.42227	-0.282	-0.30282	-0.23131	-0.30772	-0.33458	-0.32257	-0.29824	-0.31159	-0.21268	-0.45817	-0.50846
S82	-0.32636	-0.20033	-0.27468	-0.42227	-0.282	-0.30282	-0.23131	-0.30772	-0.33458	-0.32257	-0.29824	-0.31159	-0.44426	-0.45817	-0.50846
S83	-0.32636	-0.20033	-0.27468	-0.42227	-0.282	-0.30282	-0.23131	-0.30772	-0.33458	-0.32257	-0.29824	-0.31159	-0.44426	-0.45817	-0.50846
S84	-0.32636	-0.20033	-0.27468	-0.42227	-0.282	-0.30282	-0.23131	-0.30772	-0.33458	-0.32257	-0.29824	-0.31159	-0.44426	-0.45817	-0.50846
S85	-0.32636	-0.20033	-0.27468	-0.42227	-0.282	-0.30282	-0.23131	-0.30772	-0.33458	-0.32257	-0.29824	-0.31159	-0.44426	-0.45817	-0.50846
S86	-0.32636	-0.20033	-0.27468	-0.42227	-0.282	-0.30282	-0.23131	-0.30772	-0.1982	-0.32257	-0.29824	-0.31159	-0.09553	-0.40159	4.4735
S87	-0.32636	-0.20033	-0.27468	-0.42227	-0.282	-0.30282	-0.23131	-0.30772	-0.33458	-0.32257	-0.29824	-0.31159	-0.44426	-0.45817	-0.50846
S88	-0.32636	-0.20033	-0.27468	-0.42227	-0.282	-0.30282	-0.23131	-0.30772	-0.33458	-0.32257	-0.29824	-0.31159	-0.44426	-0.35095	-0.50846
S89	-0.28364	-0.20033	-0.27468	-0.42227	-0.282	-0.30282	-0.23131	-0.25978	-0.33458	-0.32257	-0.29824	4.55779	-0.13089	-0.45817	-0.50846
S90	-0.32636	-0.20033	-0.27468	-0.42227	-0.282	-0.30282	-0.23131	-0.30772	-0.33458	-0.32257	-0.29824	-0.31159	-0.44426	0.16463	-0.50846
S91	-0.32636	-0.20033	-0.27468	-0.42227	-0.282	-0.30282	-0.23131	-0.30772	-0.33458	-0.32257	-0.29824	-0.31159	-0.44426	-0.45817	-0.50846
S92	-0.32636	-0.20033	-0.27468	-0.42227	-0.282	-0.30282	-0.23131	-0.30772	-0.33458	-0.32257	-0.29824	-0.31159	-0.28833	-0.45817	-0.50846
S93	-0.32636	-0.20033	-0.27468	-0.42227	-0.282	-0.30282	-0.23131	-0.30772	-0.33458	-0.32257	-0.29824	-0.31159	-0.44426	-0.45817	-0.28844
S94	-0.32636	-0.20033	-0.27468	-0.42227	-0.282	-0.30282	-0.23131	-0.30772	-0.33458	-0.32257	-0.29824	-0.31159	-0.44426	-0.45817	-0.50846
S95	-0.32636	-0.20033	-0.27468	-0.42227	-0.282	-0.30282	-0.23131	-0.30772	-0.33458	-0.32257	-0.29824	-0.31159	-0.44426	-0.45817	-0.50846
S96	-0.04908	-0.20033	-0.27468	-0.27591	-0.282	-0.30282	-0.23131	-0.30772	-0.10475	-0.32257	-0.29824	-0.12018	-0.41158	0.99693	0.96704
S97	-0.32636	-0.20033	-0.27468	-0.42227	-0.282	-0.30282	-0.23131	-0.30772	-0.33458	-0.32257	-0.29824	-0.31159	-0.44426	-0.45817	-0.50846
S98	-0.32636	-0.20033	0.05469	-0.42227	-0.282	-0.30282	-0.23131	-0.30772	-0.33458	-0.32257	-0.29824	-0.31159	-0.44426	-0.45817	-0.50846
S99	-0.32636	-0.20033	-0.27468	-0.42227	-0.282	-0.30282	-0.23131	-0.30772	-0.33458	-0.32257	-0.29824	-0.31159	-0.44426	-0.45817	-0.50846
S100	-0.32636	-0.20033	-0.27468	-0.42227	-0.282	-0.30282	-0.23131	-0.30772	-0.33458	-0.32257	-0.29824	-0.31159	-0.41601	-0.45817	-0.50846
S101	-0.32636	-0.20033	-0.27468	-0.42227	-0.282	-0.30282	-0.23131	-0.30772	-0.33458	-0.32257	-0.29824	-0.31159	-0.44426	-0.45817	-0.50846
S102	-0.32636	-0.20033	-0.27468	-0.42227	-0.282	-0.30282	-0.23131	-0.30772	-0.33458	-0.32257	-0.29824	-0.31159	-0.44426	-0.45817	-0.50846
S103	-0.32636	-0.20033	-0.27468	-0.42227	-0.282	-0.30282	-0.21713	-0.30772	-0.33458	-0.32257	-0.29824	-0.31159	-0.39044	0.64574	-0.18298
S104	-0.32636	-0.20033	-0.27468	-0.42227	-0.282	-0.30282	-0.23131	-0.30772	-0.33458	-0.32257	-0.29824	-0.31159	-0.28409	-0.45817	-0.50846
S105	-0.32636	-0.20033	-0.27468	-0.42227	-0.282	-0.30282	-0.23131	-0.30772	-0.33458	-0.32257	-0.29824	-0.31159	-0.39206	-0.45817	-0.50846
S106	-0.32636	-0.20033	-0.27468	-0.42227	-0.282	-0.30282	-0.23131	-0.30772	-0.33458	-0.32257	-0.29824	-0.31159	-0.44426	-0.45817	-0.50846
S107	-0.32636	-0.20033	-0.27468	-0.42227	-0.282	-0.30282	-0.23131	-0.30772	-0.33458	-0.32257	-0.29824	-0.31159	-0.44426	-0.45817	-0.50846
S108	-0.32636	-0.20033	-0.27468	-0.42227	-0.282	-0.30282	-0.23131	-0.30772	-0.33458	-0.32257	-0.29824	-0.31159	-0.44426	-0.45817	-0.50846
S109	-0.32636	-0.20033	-0.27468	-0.42227	-0.282	-0.30282	-0.23131	-0.30772	-0.33458	-0.32257	-0.29824	-0.31159	-0.44426	-0.45817	-0.50846

Sample No.	integrated bins														
	7.90 .. 7.88	7.88 .. 7.86	7.86 .. 7.84	7.84 .. 7.82	7.82 .. 7.80	7.80 .. 7.78	7.78 .. 7.76	7.76 .. 7.74	7.74 .. 7.72	7.72 .. 7.70	7.70 .. 7.68	7.68 .. 7.66	7.66 .. 7.64	7.64 .. 7.62	7.62 .. 7.60
S1	-0.30614	-0.28063	-0.24761	-0.6964	-0.83629	-0.59396	-0.58558	-0.40756	-0.40556	-0.41988	-0.48839	-0.56977	-0.50972	-0.5519	-0.61732
S2	0.12632	0.59386	-0.0028	1.51834	2.25195	1.89391	2.23979	1.97173	2.55308	1.45312	2.64335	2.25147	2.60465	2.57899	2.11774
S3	-0.30614	-0.28063	-0.24761	-0.6964	-0.83629	-0.59396	-0.58558	-0.40756	-0.40556	-0.41988	-0.48839	-0.56977	-0.50972	-0.5519	-0.61732
S4	-0.30614	-0.28063	-0.24761	-0.6964	-0.83629	-0.59396	-0.58558	-0.40756	-0.40556	-0.41988	-0.48839	-0.56977	-0.50972	-0.5519	-0.61732
S5	-0.30614	-0.28063	-0.24761	-0.6964	-0.83629	-0.59396	-0.58558	-0.40756	-0.40556	-0.41988	-0.48839	-0.56977	-0.50972	-0.5519	-0.61732
S6	-0.30614	-0.28063	-0.24761	-0.37918	0.08238	0.20952	0.55196	-0.40756	-0.40556	-0.41988	-0.48839	-0.56977	-0.50972	-0.5519	-0.37847
S7	-0.30614	-0.13198	3.59713	-0.29201	-0.60337	0.05402	0.02594	-0.38995	0.08899	-0.41988	0.10778	0.1195	-0.39762	-0.25307	-0.30072
S8	-0.30614	-0.28063	-0.24761	0.03777	0.07894	-0.04874	-0.07619	-0.40756	-0.40556	-0.41988	-0.48839	-0.56977	-0.50972	-0.5519	-0.61732
S9	-0.30614	-0.28063	-0.24761	-0.27862	2.43182	-0.59396	-0.41843	-0.40756	-0.40556	-0.41988	-0.48839	-0.56977	-0.50972	-0.5519	-0.09781
S10	0.45444	0.50175	0.30044	1.22878	1.22377	1.84062	2.0048	2.64944	1.79078	1.68601	2.25417	2.29811	1.9992	2.1411	2.69227
S11	-0.30614	-0.28063	-0.24761	-0.53464	-0.81803	-0.14272	-0.04396	0.20662	1.01693	1.62936	1.43102	2.30628	2.63844	2.63035	2.73323
S12	-0.30614	-0.28063	-0.24761	-0.6964	-0.7819	-0.59396	-0.58558	-0.40756	-0.40556	-0.41988	-0.48839	-0.26932	-0.50972	-0.53669	-0.61732

S13	-0.30614	-0.28063	-0.24761	-0.6964	-0.83629	-0.59396	-0.58558	-0.40756	-0.40556	-0.41988	-0.48839	-0.56977	-0.50972	-0.5519	-0.61732
S14	0.88607	-0.09204	1.77536	5.98607	1.09086	1.79083	1.93945	0.94531	0.81451	1.31729	1.49487	1.24365	0.72728	1.09101	1.40123
S15	-0.30614	-0.28063	-0.24761	-0.6964	-0.13019	0.00076	-0.58558	-0.40756	-0.40556	-0.41988	-0.48839	-0.56977	-0.50972	-0.5519	-0.61732
S16	-0.30614	-0.28063	-0.24761	0.53168	0.31464	0.57917	0.58397	-0.35367	0.05027	0.15154	-0.26252	0.37295	0.00155	0.22638	0.98391
S17	-0.30614	-0.28063	-0.24761	-0.39187	0.08958	-0.59396	-0.58558	-0.40756	-0.40556	-0.41988	-0.48839	-0.56977	-0.50972	-0.5519	-0.61732
S18	-0.30614	-0.28063	-0.24761	0.01435	1.70138	-0.14951	0.1205	0.00782	-0.40556	-0.34298	0.00117	0.18748	0.21422	0.13329	0.59412
S19	0.22555	0.45969	0.30692	0.39765	2.4178	3.51648	3.65532	2.89282	2.87489	2.76975	2.72984	2.86879	3.61841	3.16519	3.042
S20	-0.30614	-0.28063	-0.24761	-0.24488	0.0106	-0.2486	-0.42231	-0.40756	-0.40556	-0.41988	-0.48839	-0.56977	-0.50972	-0.5519	-0.20592
S21	-0.30614	-0.28063	-0.24761	-0.6964	-0.83629	-0.59396	-0.58558	-0.40756	-0.40556	-0.41988	-0.48839	-0.56977	-0.50972	-0.5519	-0.61732
S22	0.53412	4.20567	0.186	0.50736	0.2717	0.41433	0.40206	0.48052	1.34292	1.1995	1.36406	1.01465	1.34508	1.01822	0.50832
S23	0.13818	-0.01445	0.02449	-0.07723	0.84981	0.75726	0.88726	0.85259	1.14852	0.91505	1.33501	1.64797	1.19703	1.41902	1.20989
S24	-0.30614	-0.28063	-0.24761	-0.35715	-0.7719	-0.55663	-0.58558	-0.40756	-0.40556	-0.41988	-0.48839	-0.56977	-0.50972	-0.5519	-0.61732
S25	0.70391	0.50699	0.13553	0.86001	1.43173	1.9958	2.24106	3.25656	2.30281	2.20876	1.62064	1.68994	1.51017	1.90003	1.97478
S26	0.68736	0.07524	0.05422	1.00864	2.70183	1.0119	0.93797	1.19981	1.73178	1.84683	1.90288	1.94049	1.15934	1.04666	1.72441
S27	-0.30614	-0.28063	-0.24761	0.20041	0.02873	0.5877	0.52888	-0.40756	-0.40556	-0.41988	-0.48839	-0.56977	-0.50972	-0.5519	-0.10202
S28	-0.30614	-0.28063	-0.24761	-0.6964	-0.83629	-0.59396	-0.58558	-0.40756	-0.40556	-0.41988	-0.48839	-0.56977	-0.50972	-0.5519	-0.61732
S29	-0.30614	-0.28063	-0.24761	-0.6964	-0.83629	-0.59396	-0.58558	-0.40756	-0.40556	-0.41988	-0.48839	-0.56977	-0.50972	-0.5519	-0.61732
S30	-0.30614	-0.28063	-0.24761	0.63986	1.44767	1.55164	2.01907	-0.40756	-0.40556	-0.41988	-0.42832	0.07881	-0.04015	-0.06295	0.02104
S31	-0.30614	-0.28063	-0.24761	0.34088	0.61224	0.38526	0.25497	0.12433	0.18128	-0.41988	-0.28849	0.10082	0.23672	0.30783	0.26932
S32	-0.30614	-0.28063	-0.24761	-0.6964	-0.80374	-0.59396	-0.58558	-0.40756	-0.40556	-0.41988	-0.48839	-0.56977	-0.50972	-0.5519	-0.06093
S33	-0.30614	-0.28063	-0.24761	-0.68446	-0.69829	-0.59396	-0.58558	-0.40756	-0.40556	-0.41988	-0.37851	-0.56977	-0.50972	-0.5519	-0.61732
S34	-0.30614	-0.28063	-0.24761	0.09672	0.43538	0.10437	0.19995	-0.40756	-0.40556	-0.41988	-0.48839	-0.1368	-0.26687	0.30473	0.24372
S35	-0.30614	-0.28063	-0.24761	-0.10244	0.5789	0.83098	1.31417	-0.40756	-0.07958	-0.36779	-0.38519	0.33755	-0.21274	0.23208	0.61926
S36	-0.30614	-0.28063	-0.24761	-0.56702	0.00261	-0.26203	-0.47792	-0.40756	-0.40556	-0.41988	-0.48839	-0.56977	-0.50972	-0.5519	-0.61732
S37	-0.30614	3.72404	-0.24761	-0.15614	-0.28557	-0.40551	-0.2566	-0.40756	-0.40556	-0.41988	-0.22006	0.23084	-0.40541	-0.16874	-0.4246
S38	0.73444	0.24255	0.11221	0.65199	1.15777	1.86914	1.81176	1.28231	1.54892	1.3182	1.04609	1.53723	1.00221	1.07321	0.93277
S39	-0.30614	-0.28063	-0.24761	-0.6964	-0.83591	-0.59396	-0.58558	-0.40756	-0.40556	-0.41988	-0.48839	-0.56977	-0.50972	-0.5519	-0.61732
S40	-0.07051	0.03685	0.21553	1.4016	1.30025	1.49461	1.51493	1.6587	1.46805	1.75742	1.61825	1.97314	1.90981	1.73942	1.96254
S41	-0.30614	-0.28063	-0.1707	0.33076	0.22525	1.26799	1.22968	0.60524	0.29177	-0.18515	0.48921	0.00242	0.09212	0.17595	0.62452
S42	-0.30614	-0.28063	-0.12742	0.47769	0.94525	1.55951	1.58053	0.57865	-0.37119	0.45786	0.41336	0.42575	0.23534	0.67302	0.7251
S43	-0.30614	-0.28063	-0.24761	-0.6964	-0.83629	4.12529	-0.58558	-0.40756	-0.40556	-0.41988	-0.48839	-0.56977	-0.50972	-0.5519	-0.61732
S44	-0.30614	-0.28063	-0.24761	1.33067	-0.83629	-0.59396	-0.58558	-0.40756	-0.40556	-0.41988	-0.48839	-0.56977	-0.50972	-0.5519	-0.61732
S45	0.53221	-0.28063	0.17513	1.06218	0.85816	0.52044	0.35775	0.21293	-0.2319	0.00433	0.33106	0.64054	0.61849	0.20439	0.18243
S46	-0.30614	-0.28063	-0.24761	0.88751	0.66101	-0.01196	-0.05439	0.28752	-0.40476	-0.41988	-0.24113	0.28507	0.19385	0.0456	0.47083
S47	-0.30614	-0.28063	-0.24761	-0.09757	0.48968	0.36021	0.88014	-0.40756	-0.40556	-0.41988	-0.46655	-0.20777	-0.27	-0.23878	0.18045
S48	-0.30614	-0.28063	-0.24761	-0.40513	-0.0974	-0.59396	-0.36134	-0.40756	-0.40556	-0.41988	-0.48839	-0.56977	-0.50972	-0.5519	-0.61732
S49	-0.30614	-0.28063	-0.24761	-0.48814	0.43983	0.38796	0.74726	-0.40756	0.03839	-0.41988	-0.48839	-0.50986	-0.31205	-0.26692	0.0073
S50	-0.30614	-0.28063	-0.24761	1.109	0.95282	0.31194	0.06684	-0.01055	0.34222	0.6307	0.83441	1.37226	0.89708	0.75051	0.85277
S51	-0.30614	-0.28063	-0.24761	0.53617	1.19387	0.5934	0.73444	-0.40756	-0.28005	-0.41988	-0.16369	0.04557	-0.23702	0.15179	0.07524
S52	-0.30614	-0.28063	-0.24761	-0.65163	-0.0937	-0.59396	-0.58558	-0.40756	-0.40556	-0.41988	-0.48839	-0.56977	-0.50972	-0.5519	-0.61732
S53	-0.30614	-0.28063	-0.24761	-0.11005	0.57994	-0.38922	-0.29818	-0.40756	-0.40556	-0.41988	-0.48839	-0.16188	-0.5091	-0.18085	-0.17517
S54	-0.30614	-0.28063	-0.24761	0.29684	0.34016	-0.59396	-0.48851	-0.40756	-0.40556	-0.41988	-0.43381	-0.56977	-0.50972	-0.48716	-0.16227
S55	-0.30614	-0.28063	-0.24761	-0.6964	-0.83629	-0.59396	-0.58558	-0.40756	-0.40556	-0.41988	-0.45074	-0.33976	-0.50972	-0.32254	0.25168
S56	0.42649	0.32956	0.03859	0.75275	1.05508	1.08393	1.03732	2.05974	1.18765	2.1912	2.33301	1.92556	1.41294	1.68015	1.31809

S57	-0.30614	-0.28063	-0.24761	0.07018	0.4809	-0.59396	-0.54473	-0.40756	-0.40556	-0.41988	-0.48839	-0.5297	-0.50972	-0.5519	-0.61732
S58	-0.30614	-0.28063	-0.24761	-0.6964	-0.83629	-0.59396	-0.58558	-0.40756	-0.40556	-0.41988	-0.48839	-0.56977	-0.50972	-0.5519	-0.61732
S59	-0.30614	-0.28063	-0.24761	-0.6964	-0.83629	-0.59396	-0.58558	-0.40756	-0.40556	-0.41988	-0.40218	-0.56977	-0.30192	-0.5519	-0.12589
S60	3.36442	2.1252	1.09345	2.77498	3.37143	3.9866	4.74078	5.9314	6.40329	6.24664	5.33397	4.5327	5.37026	4.83456	4.96823
S61	-0.30614	-0.28063	-0.24761	-0.6964	-0.81211	-0.59396	-0.58558	-0.40756	-0.40556	-0.41988	-0.48839	-0.56977	-0.50972	-0.5519	-0.61732
S62	-0.30614	-0.28063	-0.24761	0.47644	-0.38101	-0.59396	-0.58558	-0.40756	-0.40556	-0.41988	-0.48839	-0.56977	-0.50972	-0.5519	-0.61732
S63	0.26797	0.02756	0.06318	0.93392	2.9133	0.72211	0.71227	1.55879	1.23877	2.02633	1.96041	1.54147	1.27369	1.29169	1.05653
S64	-0.30614	-0.28063	-0.24761	1.55149	1.30132	-0.59396	-0.58558	-0.40756	-0.40556	-0.41988	-0.48839	-0.56977	-0.48322	-0.33044	-0.1073
S65	-0.30614	-0.28063	-0.24761	-0.6964	-0.83629	-0.59396	-0.58558	-0.40756	-0.40556	-0.41988	-0.48839	-0.56977	-0.50972	-0.5519	-0.61732
S66	0.24002	-0.18806	0.10152	0.95899	1.3522	0.56276	0.85638	1.71774	1.08903	0.77997	1.24897	1.80158	1.45289	1.58862	1.4063
S67	-0.30614	-0.28063	-0.24761	-0.6964	-0.83629	-0.59396	-0.58558	-0.40756	-0.40556	-0.41988	-0.48839	-0.56977	-0.50972	-0.5519	-0.61732
S68	-0.30614	-0.28063	-0.23399	0.23764	0.70391	0.27688	1.03958	0.24376	-0.15119	0.62617	-0.24745	-0.46594	0.44625	0.7493	0.84387
S69	-0.30614	7.93945	-0.24761	-0.6964	-0.73175	-0.59396	-0.58558	-0.40756	-0.40556	-0.41988	-0.33199	-0.56977	-0.50972	-0.4651	-0.61732
S70	1.29996	-0.28063	-0.24761	-0.6964	-0.74991	-0.59396	-0.58558	-0.40756	-0.40556	-0.41988	-0.48839	-0.56977	-0.50972	-0.5519	-0.61732
S71	-0.30614	-0.28063	-0.24761	-0.6964	-0.83629	-0.59396	-0.58558	-0.40756	-0.40556	-0.41988	-0.48839	-0.56977	-0.50972	-0.5519	-0.61732
S72	-0.30614	-0.28063	-0.24761	-0.6964	-0.83629	-0.59396	-0.58558	-0.40756	-0.40556	-0.41988	-0.48839	-0.56977	-0.50972	-0.5519	-0.61732
S73	-0.30614	-0.28063	-0.24761	-0.26495	-0.83629	-0.59396	-0.58558	-0.40756	-0.40556	-0.41988	-0.48839	-0.56977	-0.50972	-0.5519	-0.61732
S74	0.62438	0.46936	0.24092	1.00802	1.27535	0.91421	1.346	1.67363	1.72978	1.76674	1.95907	2.19108	2.18628	2.47639	1.97348
S75	-0.30614	-0.28063	-0.24761	0.74325	-0.83629	-0.59396	-0.58558	-0.40756	-0.40556	-0.41988	-0.48839	-0.56977	-0.50972	-0.5519	-0.61732
S76	-0.30614	-0.28063	6.19371	-0.6964	-0.83629	-0.59396	-0.58558	-0.40756	-0.40556	-0.41988	-0.48839	-0.56977	-0.50972	-0.5519	-0.61732
S77	-0.30614	-0.28063	-0.24761	-0.6964	-0.83629	-0.59396	-0.58558	-0.40756	-0.40556	-0.41988	-0.48839	-0.56977	-0.50972	-0.5519	-0.61732
S78	-0.30614	-0.28063	-0.24761	0.01112	-0.83629	-0.59396	-0.58558	-0.40756	-0.40556	-0.41988	-0.48839	-0.56977	-0.50972	-0.5519	-0.61732
S79	-0.30614	-0.28063	-0.24761	-0.14375	-0.28246	-0.59396	-0.43936	-0.33997	-0.40556	-0.41988	0.05377	-0.20033	-0.37167	0.06457	-0.0866
S80	-0.30614	-0.02494	7.22055	-0.59515	-0.23419	-0.59396	-0.58558	-0.40756	-0.40556	-0.41988	-0.48839	-0.13812	-0.02339	-0.24609	-0.38014
S81	0.01348	0.54141	-0.17102	0.58213	0.65593	-0.53474	-0.41114	-0.40756	-0.40556	-0.41988	-0.30916	-0.08112	-0.25565	0.05158	-0.2984
S82	-0.30614	-0.28063	-0.24761	-0.6964	-0.83629	1.93578	-0.58558	-0.40756	-0.40556	-0.41988	-0.48839	-0.56977	-0.50972	-0.5519	-0.61732
S83	-0.30614	-0.28063	-0.24761	-0.6964	-0.83629	-0.59396	-0.58558	-0.40756	-0.40556	-0.41988	-0.48839	-0.56977	-0.50972	-0.5519	-0.61732
S84	-0.30614	-0.28063	-0.24761	-0.6964	-0.83629	-0.59396	-0.58558	-0.40756	-0.40556	-0.41988	-0.48839	-0.56977	-0.50972	-0.5519	-0.61732
S85	-0.30614	-0.28063	-0.24761	-0.6964	-0.38908	-0.59396	-0.58558	-0.40756	-0.40556	-0.41988	-0.48839	-0.56977	-0.50972	-0.5519	-0.61732
S86	-0.30614	-0.28063	-0.24761	-0.6964	-0.64589	-0.59396	-0.41398	-0.40756	-0.40556	-0.41988	-0.07078	-0.1467	-0.1564	-0.23711	-0.61732
S87	-0.30614	-0.28063	-0.24761	-0.34382	0.52148	-0.59396	-0.58558	-0.40756	-0.40556	-0.41988	-0.48839	-0.56977	-0.50972	-0.5519	-0.61732
S88	-0.30614	-0.28063	-0.24761	0.06226	-0.08813	-0.59396	-0.58558	-0.40756	-0.40556	-0.41988	-0.48839	-0.56977	-0.2269	-0.37558	-0.61732
S89	-0.30614	-0.28063	-0.24761	-0.05469	-0.16552	-0.223	-0.28846	-0.40756	-0.40556	-0.04638	0.53672	0.2568	-0.13074	0.26818	0.06122
S90	6.27875	-0.28063	-0.24761	-0.6964	-0.54716	-0.18523	-0.58558	-0.40756	-0.40556	-0.41988	-0.48839	-0.39499	-0.50972	-0.5519	-0.54688
S91	-0.30614	-0.28063	-0.24761	-0.6964	-0.05131	-0.59396	0.93443	-0.40756	-0.40556	-0.41988	-0.48839	-0.56977	-0.50972	-0.5519	-0.61732
S92	-0.30614	-0.28063	-0.24761	0.36827	-0.26794	-0.59396	-0.58558	-0.40756	-0.40556	-0.41988	-0.48839	-0.56977	-0.50972	-0.53782	-0.46346
S93	-0.30614	-0.28063	-0.24761	-0.6964	-0.83629	-0.59396	-0.58558	-0.40756	-0.40556	-0.41988	-0.48839	-0.56977	-0.50972	-0.5519	-0.61732
S94	-0.30614	-0.28063	-0.24761	-0.6964	-0.83629	-0.59396	-0.58558	-0.40756	-0.40556	-0.41988	-0.48839	-0.56977	-0.50972	-0.5519	-0.61732
S95	-0.30614	-0.28063	0.25231	-0.6964	-0.83629	-0.59396	-0.58558	-0.40756	-0.40556	-0.41988	-0.48839	-0.56977	-0.50972	-0.5519	-0.61732
S96	-0.18001	-0.28063	-0.13694	0.48857	0.68637	0.12891	-0.09093	0.11435	0.65661	0.76253	1.77715	1.74254	1.27954	0.8847	0.56258
S97	-0.30614	0.18293	-0.24761	-0.62563	-0.34208	-0.59396	-0.58558	-0.40756	-0.40556	-0.2567	-0.48839	-0.31211	-0.50972	-0.52076	-0.24246
S98	-0.30614	-0.28063	-0.24761	-0.6964	-0.83629	-0.59396	-0.58558	-0.40756	-0.40556	-0.41988	-0.48839	-0.56977	-0.50972	-0.5519	-0.61732
S99	-0.30614	-0.28063	-0.24761	-0.6964	-0.83629	-0.59396	-0.58558	-0.40756	-0.40556	-0.41988	-0.48839	-0.56977	-0.50972	-0.5519	-0.61732
S100	4.21739	-0.28063	-0.24761	-0.6964	-0.6181	-0.59396	-0.58558	-0.40756	-0.40556	-0.41988	-0.48839	-0.45413	-0.50972	-0.5519	-0.61732

S101	-0.30614	2.27545	-0.24761	-0.6964	-0.83629	-0.59396	-0.58558	-0.40756	-0.40556	-0.41988	-0.48839	-0.56977	-0.50972	-0.5519	-0.61732
S102	-0.30614	0.40369	-0.24761	-0.32421	-0.62916	-0.59396	-0.58558	-0.40756	-0.40556	-0.41988	-0.48839	-0.56977	-0.50972	-0.5519	-0.61732
S103	-0.30614	-0.28063	-0.24761	-0.6964	-0.77225	-0.59396	-0.58558	-0.40756	-0.40556	-0.41988	-0.48839	-0.56977	0.53183	-0.43072	-0.61732
S104	5.46195	-0.28063	-0.24761	-0.68488	-0.41963	-0.59396	-0.58558	-0.40756	-0.40556	-0.41988	-0.00526	0.02871	-0.38612	-0.07097	-0.18899
S105	-0.30614	0.67203	-0.24761	-0.6964	-0.83629	-0.59396	-0.58558	-0.40756	-0.40556	-0.41988	-0.48839	-0.56977	-0.50972	-0.5519	-0.61732
S106	-0.30614	-0.28063	-0.24761	1.0887	-0.52895	-0.59396	-0.58558	-0.40756	-0.40556	-0.41988	-0.48839	-0.56977	-0.41154	-0.5519	-0.61732
S107	-0.30614	-0.28063	-0.24761	1.2495	-0.83629	-0.59396	-0.58558	-0.40756	-0.40556	-0.41988	-0.48839	-0.56977	-0.50972	-0.5519	-0.61732
S108	-0.30614	-0.28063	-0.24761	-0.6964	-0.78876	-0.59396	-0.58558	-0.40756	-0.40556	-0.41988	-0.48839	-0.56977	-0.50972	-0.5519	-0.61732
S109	-0.30614	-0.28063	-0.24761	3.8429	-0.09722	-0.59396	-0.58558	-0.40756	-0.40556	-0.41988	-0.48839	-0.56977	-0.50972	-0.5519	-0.61732

Sample No.	integrated bins														
	7.60 .. 7.58	7.58 .. 7.56	7.56 .. 7.54	7.54 .. 7.52	7.52 .. 7.50	7.50 .. 7.48	7.48 .. 7.46	7.46 .. 7.44	7.44 .. 7.42	7.42 .. 7.40	7.40 .. 7.38	7.38 .. 7.36	7.36 .. 7.34	7.34 .. 7.32	7.32 .. 7.30
S1	-0.6427	-0.69766	-0.8417	-0.87497	-0.85858	-0.73334	-0.71753	-0.67782	-0.68917	-0.67138	-0.77721	-1.10463	-1.01546	-0.68525	-0.13253
S2	2.58531	2.05155	1.76796	1.52695	1.61459	2.16969	1.76246	2.05043	1.78738	2.08867	2.04026	1.72004	1.79891	1.73546	1.54204
S3	-0.6427	-0.69766	-0.8417	-0.87497	-0.85858	-0.73334	-0.71753	-0.67782	-0.68917	-0.67138	-0.77721	-0.86496	-0.30927	-0.17115	-0.51831
S4	-0.6427	-0.69766	-0.8417	-0.73584	-0.85858	-0.73334	-0.71753	-0.67782	-0.60158	-0.25969	-0.62134	-0.08159	-0.33168	-0.52286	-0.37442
S5	-0.6427	-0.69766	-0.8417	-0.87497	-0.85858	-0.73334	-0.71753	-0.67782	-0.68917	-0.67138	-0.44631	0.16715	0.03926	0.02417	-0.1883
S6	-0.4208	-0.26167	0.30197	0.41143	0.216	-0.22137	-0.08591	-0.20152	-0.15726	-0.05585	0.00393	0.2171	0.00864	-0.00267	0.09542
S7	-0.05225	0.08468	0.72436	-0.14636	-0.10246	0.52598	0.40958	0.08811	0.14975	0.38647	0.23983	0.50738	0.18546	0.00986	-0.21933
S8	-0.6427	-0.69766	-0.8417	-0.87497	-0.85858	-0.73334	-0.71753	-0.67782	-0.68917	-0.67138	-0.77721	-0.67784	-0.87303	-0.61626	-0.58054
S9	-0.11603	0.35041	0.41206	0.60326	0.22953	-0.18718	-0.12093	-0.05062	0.23503	0.04093	0.65183	0.50968	0.80799	0.69938	0.96414
S10	2.41594	2.45185	2.67216	2.47866	2.03665	1.71139	1.45741	1.65504	1.54386	1.45242	1.45031	0.9181	0.91749	0.87215	1.08377
S11	2.8642	2.98635	2.877	2.51297	3.24402	4.07894	4.1487	4.65634	4.8373	5.42652	5.09973	3.84589	3.88677	3.54836	3.18201
S12	-0.63288	-0.4271	0.35733	-0.28237	-0.22473	-0.39404	-0.66458	-0.67782	-0.68917	-0.67138	-0.62851	0.05903	-0.24452	-0.53314	-0.79966
S13	-0.6427	-0.54541	-0.54049	-0.13589	-0.52534	0.77562	0.92201	-0.67782	-0.68917	-0.67138	-0.33232	0.00701	-0.38789	-0.2178	-0.03443
S14	0.68133	0.4999	0.69646	0.3179	0.45979	0.53449	0.35843	0.38663	0.5045	0.57577	0.47997	0.81245	0.42597	0.0661	0.07236
S15	-0.6427	-0.69766	-0.8417	-0.78638	-0.85858	-0.73334	-0.71753	-0.67782	-0.68917	-0.67138	-0.77721	-1.10463	-0.88276	-0.37812	-0.49348
S16	1.17162	0.85973	0.75214	0.52312	0.37058	-0.09375	0.22551	0.15431	0.14377	0.17694	0.31362	0.36125	0.31306	0.34713	0.35199
S17	-0.6427	-0.69766	-0.8417	-0.44488	-0.85858	-0.73334	-0.71753	-0.67782	-0.68917	-0.67138	-0.55776	-0.03591	0.10231	0.54917	0.62953
S18	0.53385	0.14591	0.65653	0.60153	0.627	0.54621	0.76807	0.71947	0.74123	0.59019	0.80656	0.99391	1.11801	1.40258	1.51018
S19	3.42482	3.4412	2.66299	2.4366	3.0097	3.32845	3.22013	3.1512	3.04723	3.28555	2.82741	2.35763	3.40935	2.57454	2.63167
S20	-0.30053	-0.27651	-0.31413	-0.18473	-0.65745	-0.73334	-0.71753	-0.67782	-0.68917	-0.67138	-0.77721	-0.28139	-0.18506	-0.19425	0.39318
S21	-0.6427	-0.69766	-0.8417	-0.87497	-0.85858	-0.73334	-0.71753	-0.67782	-0.68917	-0.67138	-0.77721	-1.10463	-1.10848	-1.38037	-1.38198
S22	0.89268	0.45766	0.63333	0.11423	0.3873	0.94515	1.16242	1.07612	1.04471	1.24608	1.18614	0.60452	0.37348	0.18555	0.07781
S23	1.47599	1.69078	0.76777	0.31616	0.55268	0.68418	0.78468	0.71177	0.77458	0.66727	0.49408	0.229	0.2241	0.02911	0.0606
S24	-0.6427	-0.69766	-0.8417	-0.87497	-0.85858	-0.73334	-0.71753	-0.67782	-0.68917	-0.67138	-0.77277	-0.58655	-0.75865	-0.53758	-0.56087
S25	1.88953	1.99093	1.52846	1.6435	1.6009	1.32432	1.31186	1.36166	1.1619	0.90217	0.74291	0.69165	0.8102	0.75907	0.7334
S26	1.5374	1.06961	1.20042	1.15212	0.94534	1.0625	0.63839	0.42615	0.74896	0.60046	0.52492	0.59012	0.65355	0.63508	0.55984
S27	-0.47954	-0.45553	-0.02382	0.16897	-0.30498	-0.73334	-0.71753	-0.67782	-0.68917	-0.67138	-0.65939	-0.64909	-0.59839	-0.25181	-0.19456
S28	-0.6427	-0.61849	-0.78003	-0.58454	-0.85858	-0.73334	-0.71753	-0.67782	-0.68917	-0.67138	-0.77721	-1.10463	-0.91926	-0.53485	-0.52897
S29	-0.6427	-0.69766	-0.8417	-0.87497	-0.85858	-0.73334	-0.71753	-0.67782	-0.68917	-0.67138	-0.77721	-1.10463	-1.10848	-0.61416	0.02687
S30	-0.09122	0.00283	0.08685	0.35634	0.23206	0.52826	0.41297	0.70159	0.86584	0.70441	0.93662	2.22595	2.08085	1.86886	1.79993
S31	0.33335	0.37834	0.44217	0.48655	0.25259	0.37892	0.03816	0.06096	-0.50477	-0.37422	0.52115	1.13955	0.76613	0.52829	0.02907
S32	-0.6427	-0.69766	0.68178	0.60494	-0.46989	-0.73334	-0.71753	-0.67782	-0.68917	-0.67138	-0.77721	-0.15838	0.12473	1.02336	1.50858

S33	-0.17394	-0.30647	-0.49186	-0.75956	-0.67014	-0.73334	-0.71753	-0.67782	-0.68917	-0.67138	-0.77721	-0.65791	-0.6297	-0.53288	-0.58515
S34	0.47559	0.39827	0.36701	0.09598	-0.10416	-0.21957	-0.15527	-0.12196	-0.31372	-0.23379	-0.18755	0.01592	-0.07349	-0.03865	-0.1876
S35	1.32431	1.23983	0.88193	0.78736	0.52934	0.29914	0.12817	0.47647	0.5324	0.3382	0.57167	0.29596	0.75207	0.80746	0.78452
S36	-0.3646	-0.43013	-0.52526	-0.45366	-0.54425	-0.73334	-0.71753	-0.67782	-0.68917	-0.61578	-0.52487	-0.31859	-0.20406	0.03616	-0.25886
S37	0.07222	-0.33791	-0.624	-0.65605	-0.38319	-0.05293	-0.276	0.57072	0.52366	-0.02614	0.07489	0.28277	0.36456	0.67489	1.16087
S38	0.92305	1.03493	0.53344	0.39647	0.59024	0.71794	0.58217	0.68675	0.5198	0.59541	0.5715	0.37834	0.3808	0.35272	0.38976
S39	-0.6427	-0.69766	-0.8417	-0.87497	-0.85858	-0.73334	-0.71753	-0.67782	-0.68917	-0.67138	-0.77721	-0.62489	-0.73184	-0.90567	-1.08414
S40	2.04076	2.1445	1.99723	1.71678	1.5552	1.58219	1.36161	1.64313	1.8173	1.82816	2.02456	1.49144	1.59527	1.49503	1.16082
S41	0.70319	0.66048	1.3547	1.32165	0.646	0.37089	-0.09846	0.37099	0.25303	0.08718	0.10551	0.17697	0.2033	0.23884	0.25621
S42	0.55885	1.12174	0.89208	0.97675	0.9066	0.42256	0.43667	0.33774	0.26154	-0.03065	0.46233	0.47392	0.49457	0.60632	0.43137
S43	-0.6427	-0.69766	-0.8417	-0.87497	-0.85858	-0.73334	-0.71753	-0.67782	-0.68917	-0.67138	-0.70862	-0.61711	-0.88121	-0.933	-1.18044
S44	-0.6427	-0.69766	-0.8417	-0.85284	-0.85858	-0.73334	-0.71753	-0.63441	-0.68917	-0.66362	-0.6986	-0.23381	-0.5403	-0.45865	-0.69323
S45	0.0666	-0.03928	-0.26852	-0.32371	0.02938	0.04678	0.45562	0.22963	0.14952	-0.00946	0.15809	-0.17182	-0.17288	-0.25995	-0.59496
S46	0.56703	0.69145	0.95993	0.7301	0.44445	-0.00076	-0.25602	-0.08268	-0.06517	-0.01178	0.05111	0.07277	0.29817	0.0741	0.02574
S47	0.65101	0.64301	0.91209	1.01383	0.32295	0.11872	-0.04514	0.10633	0.18365	0.00349	0.25187	-0.004	0.25547	0.14765	0.25455
S48	-0.6427	-0.48787	-0.52834	-0.10183	-0.85631	-0.73334	-0.71753	-0.67782	-0.68917	-0.67138	-0.77721	-0.64471	-0.56862	-0.56837	-0.28924
S49	0.10198	-0.02798	0.14496	0.71934	0.38449	-0.20747	-0.58733	-0.60762	-0.59521	-0.6152	-0.60513	-0.32893	-0.23751	-0.00954	-0.07925
S50	0.44582	0.81661	0.28445	0.16613	0.50725	0.99536	1.07835	1.24043	1.30832	1.62747	1.83964	1.37998	1.31832	1.20591	1.11987
S51	-0.40434	-0.18991	-0.26947	-0.29953	-0.37552	0.18015	0.00245	0.39467	0.2561	0.2644	0.5913	1.1485	1.09184	1.14024	0.92837
S52	-0.6427	-0.69766	-0.8417	-0.74248	-0.53875	-0.73334	0.1911	0.00766	0.23694	0.06311	1.0382	1.8277	1.79005	2.19475	1.81344
S53	-0.6427	-0.65287	-0.72028	-0.55602	-0.23439	0.48002	0.99185	-0.3397	-0.04767	-0.51451	-0.34293	-0.03263	0.23917	0.24085	0.29844
S54	-0.08661	-0.06911	-0.20972	-0.47958	-0.29993	-0.21135	-0.48435	-0.34754	-0.43643	-0.37458	-0.37716	-0.12164	-0.03263	0.12259	0.15487
S55	-0.2997	-0.07177	-0.2573	-0.27985	0.08947	1.49034	2.36211	0.61392	0.34868	0.8842	0.9505	1.08786	0.98462	1.27995	1.39273
S56	1.26196	1.54662	0.72173	0.68186	1.15635	1.56941	1.70599	1.98093	1.69024	1.7559	1.83504	1.65731	1.58586	1.70957	1.64177
S57	-0.6427	-0.69766	-0.8417	-0.73322	-0.61442	-0.33486	-0.29648	-0.2789	-0.01402	-0.16446	-0.53203	0.11639	0.01494	0.40766	0.25113
S58	-0.6427	-0.69766	-0.8417	-0.87497	-0.85858	-0.73334	-0.71753	-0.67782	-0.68917	-0.67138	-0.77721	-0.11071	-0.52192	-0.6036	-0.78253
S59	-0.6427	-0.68884	-0.52649	-0.48806	-0.55065	0.1477	0.21463	-0.31129	-0.14711	-0.51725	-0.10346	-0.04455	-0.09884	-0.03877	0.02121
S60	4.55176	4.40072	4.20946	3.75553	4.02686	3.93623	3.54187	3.76392	3.61698	3.44042	2.82209	2.57946	2.65421	2.74564	2.41032
S61	-0.6427	-0.69766	-0.8417	-0.87497	-0.85858	-0.73334	-0.71753	-0.67782	-0.68917	-0.67138	-0.77721	-1.10463	-1.10848	-0.92366	-0.49407
S62	-0.6427	-0.69766	-0.40283	-0.33662	-0.85858	-0.73334	-0.71753	-0.67782	-0.68917	-0.67138	-0.77721	-0.58718	-0.62413	-0.66688	-0.71304
S63	0.99754	0.42333	0.91468	0.95145	1.24344	1.04399	1.28867	1.08493	1.08283	0.91073	1.16508	1.72472	1.69339	1.56779	1.51318
S64	0.17513	0.00584	0.08787	-0.4167	-0.23106	0.01849	0.77541	0.94384	0.95671	0.59716	1.37092	1.74241	1.56826	2.09082	2.46486
S65	-0.6427	-0.69766	-0.8417	-0.87497	-0.85858	-0.73334	-0.71753	-0.67782	-0.68917	-0.67138	-0.77721	-1.10463	-1.0597	-0.60402	-0.44312
S66	1.3588	1.19424	1.35498	1.45153	1.40188	1.73087	2.03525	1.86057	1.5707	1.1448	1.40373	1.29762	1.10938	0.9192	0.79418
S67	-0.6427	-0.69766	-0.8417	-0.84816	-0.85858	-0.69843	0.33863	-0.67782	-0.68917	-0.67138	-0.77721	0.13551	-0.41264	-0.33932	-0.47459
S68	0.53632	0.98331	2.4412	3.54341	1.78296	0.64992	0.13789	0.21071	0.28793	0.24908	0.18173	0.43992	0.28705	0.47088	0.46641
S69	-0.13307	-0.21452	0.58265	0.72008	0.89908	-0.56806	-0.55434	0.32086	-0.3485	-0.09159	0.01203	0.52113	0.16282	0.56761	0.78239
S70	-0.6427	-0.69766	-0.20688	-0.04199	-0.00617	-0.6845	-0.71753	-0.67782	-0.68917	-0.67138	-0.77721	-0.8961	-0.96271	-0.9872	-0.85288
S71	-0.6427	-0.69766	0.35289	0.4009	-0.00717	-0.73334	-0.71753	-0.67782	-0.68917	-0.67138	-0.77721	-1.10463	-0.78841	-0.71655	-0.76424
S72	-0.6427	-0.69766	0.64468	0.75123	-0.52013	-0.73334	-0.71753	-0.67782	-0.68917	-0.67138	-0.77721	-1.10463	-0.98453	-0.41703	-0.43053
S73	-0.6427	-0.69766	-0.8417	-0.87497	-0.85858	-0.73334	-0.71753	-0.67782	-0.68917	-0.67138	-0.77721	-1.10463	-1.10848	-1.38037	-1.52733
S74	1.68362	1.65167	1.91545	2.26187	2.88696	2.21215	1.73097	1.51329	1.89991	1.45801	1.1819	0.58342	0.82023	0.45517	0.40221
S75	-0.6427	-0.69766	-0.63265	-0.42165	-0.4959	-0.73334	-0.71753	-0.67782	-0.68917	-0.67138	-0.77721	-1.10463	-1.10848	-1.00962	-1.1681
S76	-0.45482	-0.21974	0.57247	1.76849	1.1723	-0.26576	-0.39117	-0.50165	-0.14397	-0.67138	-0.25847	-0.11941	-0.17212	-0.04891	-0.02906

S77	-0.6427	-0.69766	-0.65365	-0.44957	-0.58966	-0.73334	-0.71753	-0.67782	-0.68917	-0.67138	-0.77721	-1.10463	-1.10848	-1.17892	-1.19325
S78	-0.6427	-0.69766	-0.8417	-0.87497	-0.85858	-0.73334	-0.71753	-0.67782	-0.68917	-0.67138	-0.77721	-1.10463	-1.10848	-1.3146	-1.22663
S79	-0.05911	0.22438	0.88442	0.70825	0.7176	0.03754	0.22717	0.32099	0.26424	0.16088	0.57234	0.97576	0.69202	0.69233	0.35821
S80	-0.54004	-0.48687	0.27929	0.64875	0.62559	0.1977	0.06071	0.42215	0.70065	0.51726	0.23574	0.30243	0.51155	0.7761	0.98648
S81	-0.16423	0.33392	-0.45215	-0.61369	-0.05978	0.20603	0.10478	0.01055	0.08315	0.03339	-0.23411	-0.32341	-0.27599	-0.41933	-0.48157
S82	-0.6427	-0.69766	-0.8417	-0.87497	-0.85858	-0.73334	-0.71753	-0.67782	-0.68917	-0.67138	-0.77721	-1.10463	-1.10848	-1.38037	-1.57048
S83	-0.6427	-0.69766	-0.8417	-0.87497	-0.85858	-0.73334	-0.71753	-0.67782	-0.68917	-0.67138	-0.77721	-1.10463	-1.10848	-1.38037	-1.57048
S84	-0.6427	-0.69766	-0.8417	-0.87497	-0.85858	-0.73334	-0.71753	-0.67782	-0.68917	-0.67138	-0.77721	-1.10463	-1.10848	-1.38037	-1.57048
S85	-0.6427	-0.69766	-0.8417	-0.87497	-0.85695	-0.73334	-0.51887	-0.67782	-0.59957	0.38934	0.89389	1.38374	0.80065	0.71881	0.90797
S86	-0.27543	-0.20014	0.00327	0.13239	0.13899	-0.32755	-0.5696	-0.28132	-0.09737	-0.19258	-0.30737	-0.51957	-0.18168	-0.28816	-0.21425
S87	-0.6427	-0.69766	-0.8417	-0.87497	-0.85858	-0.73334	-0.71753	-0.67782	-0.68917	-0.67138	-0.77721	-0.98097	-0.82218	-1.0055	-0.94462
S88	-0.29846	0.06658	-0.56628	-0.51034	0.30511	0.01234	-0.29572	0.13095	-0.10752	-0.25038	-0.0053	-0.18809	-0.24769	-0.03065	-0.20976
S89	0.49408	0.69556	0.18762	0.23839	0.61275	0.12378	0.22853	0.35858	0.39262	0.57802	0.09451	0.12627	0.26058	0.29361	0.02063
S90	-0.6427	0.21677	0.21328	-0.55884	-0.27925	-0.04977	-0.17033	-0.42031	-0.33017	-0.35365	-0.62954	-0.36778	-0.17586	-0.60355	-0.55202
S91	-0.61375	-0.69766	-0.20998	0.20597	0.32599	-0.31412	-0.05328	-0.20639	0.29691	0.56153	0.59176	0.60568	0.47815	0.35077	0.57023
S92	-0.33093	-0.19234	-0.54643	-0.5905	-0.45466	-0.41629	-0.71753	-0.51382	-0.53318	-0.53347	-0.47263	-0.65306	-0.82893	-0.78173	-0.67403
S93	-0.6427	-0.69766	-0.8417	-0.87497	-0.85858	-0.73334	-0.71753	-0.67782	-0.68917	-0.67138	-0.77721	-0.03979	-0.41166	-0.86441	-0.71642
S94	-0.6427	-0.69766	-0.8417	-0.87497	-0.85858	-0.73334	-0.71753	-0.67782	-0.68917	-0.67138	-0.77721	-1.10463	-1.10848	-1.38037	-1.57048
S95	-0.6427	-0.69766	-0.8417	-0.87497	-0.85858	-0.73334	-0.71753	-0.67782	-0.68917	-0.67138	-0.77721	-1.10463	-1.10848	-1.11028	-1.05794
S96	0.81454	0.73455	0.44075	0.24313	0.75244	1.03867	1.0455	1.07686	1.09188	1.04289	1.34717	0.84536	0.83632	0.59439	0.40048
S97	-0.28404	-0.19813	-0.5018	-0.61581	-0.34719	-0.12505	-0.10038	-0.25083	-0.23253	-0.1764	-0.41112	-0.60548	-0.39721	-0.60802	-0.8298
S98	-0.6427	-0.69766	-0.8417	-0.87497	-0.85858	-0.73334	-0.71753	-0.67782	-0.68917	-0.67138	-0.77721	-1.10463	-1.10848	-1.38037	-1.57048
S99	-0.6427	-0.69766	-0.8417	-0.87497	-0.85858	-0.73334	-0.71753	-0.67782	-0.68917	-0.67138	-0.77721	-1.10463	-1.10848	-1.36276	-1.47356
S100	-0.62552	-0.26766	-0.16224	-0.04789	0.24814	-0.22983	-0.63365	-0.38973	-0.56894	-0.38081	-0.27148	-0.57248	-0.26124	-0.29664	-0.40821
S101	-0.6427	-0.69766	-0.8417	-0.87497	-0.85858	-0.73334	-0.71753	-0.67782	-0.68917	-0.67138	-0.77721	-1.03122	-0.74282	-0.72818	-0.83117
S102	-0.6427	-0.69766	-0.8417	-0.87497	-0.85858	-0.73334	-0.71753	-0.67782	-0.68917	-0.67138	-0.77721	-1.10463	-1.10848	-1.38037	-1.30818
S103	-0.6427	-0.69766	-0.71982	0.11543	1.27989	1.13406	0.23534	0.01551	0.10187	0.33436	-0.00095	0.02794	0.43173	0.35041	0.29438
S104	-0.15436	-0.4085	-0.17584	0.12601	0.51878	0.21059	-0.06929	-0.25785	-0.30516	-0.14886	-0.01906	0.01062	0.12435	-0.0496	-0.05313
S105	-0.6427	-0.69766	-0.8417	-0.87497	-0.85858	-0.73334	-0.71753	-0.67782	-0.68917	-0.67138	-0.7514	-0.94855	-0.84466	-0.77024	-0.71873
S106	-0.55093	-0.69766	-0.8417	-0.64378	0.23853	0.03076	0.2466	-0.28847	-0.29693	-0.26768	-0.07074	0.16304	-0.20509	-0.23711	-0.24919
S107	-0.6427	-0.69766	-0.8417	-0.87497	-0.85858	-0.73334	-0.71753	-0.67782	-0.68917	-0.67138	-0.77721	-1.10463	-1.10848	-1.38037	-1.25624
S108	-0.6427	-0.69766	-0.8417	-0.87497	-0.85858	-0.73334	-0.71753	-0.67782	-0.68917	-0.67138	-0.77721	-1.10463	-1.10848	-1.33515	-1.27556
S109	-0.6427	-0.69766	-0.8417	-0.87497	-0.85858	-0.73334	-0.71753	-0.67782	-0.68917	-0.67138	-0.77721	-0.71905	-0.85343	-0.82241	-0.54157

Sample No.	integrated bins														
	7.30 .. 7.28	7.28 .. 7.26	7.26 .. 7.24	7.24 .. 7.22	7.22 .. 7.20	7.20 .. 7.18	7.18 .. 7.16	7.16 .. 7.14	7.14 .. 7.12	7.12 .. 7.10	7.10 .. 7.08	7.08 .. 7.06	7.06 .. 7.04	7.04 .. 7.02	7.02 .. 7.00
S1	-0.00627	-0.33491	-0.36859	-0.59823	-0.01937	0.06686	-0.1145	-0.25468	-0.12277	1.84397	-0.41561	-0.59517	-0.54115	-0.85559	-1.00132
S2	1.48851	1.3178	1.44164	0.96887	1.17646	1.16956	0.99512	3.05604	0.72572	0.42748	0.40529	1.03334	0.87158	1.30716	1.58048
S3	-0.79948	-0.878	-0.76194	-0.6744	-0.35343	-0.47845	-0.39679	0.40401	1.08581	-0.80763	-0.99204	-1.03933	-1.02255	-1.29097	-1.00132
S4	0.28483	-0.17207	-0.4154	-0.40702	-0.14419	-0.43171	-0.46576	-0.61087	-0.59321	0.10545	-0.13672	-0.02716	-0.1402	-0.02146	-0.38803
S5	-0.39934	0.17989	-0.66078	-0.73465	-0.71313	-0.69548	-0.4609	-0.18669	-0.15784	0.24476	4.8047	-1.20403	-1.30681	-1.29097	-1.00132
S6	-0.00408	-0.35121	-0.19257	-0.45305	-0.14436	-0.04199	-0.0848	-0.09557	-0.1375	-0.07202	2.1406	-0.0387	0.09016	0.0307	-0.00656
S7	-0.16283	-0.36427	-0.4174	-0.66801	-0.48445	-0.33221	-0.2455	-0.38626	-0.42552	-0.42545	-0.37711	-0.2875	1.7748	-0.10275	-0.05435
S8	-0.61131	-0.52609	-0.61248	-0.74377	-0.46283	-0.59367	-0.65728	-0.51689	1.07162	-0.76781	-0.9669	-0.96777	-0.78737	-1.00154	-1.00132

S9	0.99808	1.21227	1.45776	1.12385	1.85083	4.13974	0.9526	0.16073	0.0803	-0.01401	0.07236	0.4306	0.37951	0.57171	0.14949
S10	1.21093	0.84737	1.149	0.77233	1.176	3.85915	1.74298	0.0994	-0.02574	0.08451	0.22777	0.61157	0.54547	0.70693	0.89108
S11	2.93647	2.60246	2.8742	2.17472	2.08535	2.1266	1.99082	1.56031	1.41634	1.30584	1.59847	2.59013	2.1971	3.4606	4.26644
S12	-0.87037	-0.59021	-0.96415	-0.86158	-1.00711	-0.7127	-0.76215	-0.64275	-0.61054	-0.59766	0.78314	-1.15705	-1.03165	-1.0596	-0.83177
S13	-0.33603	0.23896	0.12111	0.47793	5.08957	0.80653	-0.42174	-0.33325	-0.36561	-0.60695	-0.61956	-0.84844	-0.88904	-0.65259	-0.68613
S14	-0.16707	-0.11658	-0.09247	-0.53874	-0.36441	-0.24204	-0.20358	-0.08479	-0.16962	-0.21905	-0.30949	0.01139	1.85032	0.24139	0.24981
S15	-0.23738	-0.1243	0.17123	1.59383	0.99412	0.34859	-0.33218	-0.64113	-0.7182	-0.74544	-0.74972	-0.7181	-0.62192	-0.85426	-1.00132
S16	0.21224	0.38511	0.18891	0.00935	0.04509	0.0667	-0.09927	-0.13275	1.11204	0.38646	-0.18241	0.01336	-0.08616	-0.00511	0.12594
S17	0.55284	0.44676	0.51549	0.28737	0.6644	0.56252	0.73617	3.0875	-0.39319	-0.41837	-0.32176	-0.15978	-0.28273	-0.64603	-0.919
S18	1.47711	1.2982	1.70438	1.20216	1.53847	1.8934	4.89982	0.48272	0.15172	0.13652	0.28514	0.87908	0.65947	0.71772	0.62564
S19	2.51484	2.26923	2.97786	1.85092	2.36242	2.16554	2.2461	4.04643	0.88169	1.08627	1.25632	2.17902	1.83965	2.50221	2.46318
S20	0.42876	0.11818	0.33061	0.14655	0.37514	0.46611	2.26972	1.06998	-0.34124	-0.36831	-0.28339	-0.17226	-0.22699	-0.30978	-0.3568
S21	-1.50923	-0.95092	-1.23122	-1.19728	-0.73492	-0.49751	1.46725	-0.86585	-0.96567	-1.1372	-1.33785	-1.68263	-1.33482	-1.29097	-1.00132
S22	-0.09437	-0.20585	-0.08617	-0.33264	-0.05818	-0.021	-0.01451	-0.15224	-0.18706	-0.20466	-0.16353	0.5596	0.89043	0.52263	0.66858
S23	0.08511	-0.17243	0.27201	-0.26079	-0.01077	0.06072	-0.02167	-0.23909	-0.15026	0.42489	0.39148	-0.18344	-0.03557	0.30591	0.3673
S24	-0.72859	-0.98513	-0.72019	-0.89486	-0.63771	-0.55592	-0.63076	-0.62518	-0.59268	-0.56547	1.17984	0.31493	-0.67406	-0.70532	-0.704
S25	0.80954	0.73919	0.86196	0.32464	0.7632	0.91389	2.08077	2.32671	0.29651	0.21887	0.30116	0.65449	0.7325	1.15222	0.97526
S26	0.39181	0.44094	0.56259	0.19201	0.55582	0.59312	0.46169	0.27245	0.82383	2.85849	0.36772	0.64879	0.59707	0.77928	0.75944
S27	-0.33013	-0.5452	-0.43976	-0.53239	-0.24972	-0.21514	-0.03327	0.46082	-0.67151	-0.63763	-0.66761	-0.64037	-0.64733	-0.69748	-0.78735
S28	-0.77445	-0.78373	-0.61706	-0.77819	-0.44563	-0.42269	-0.35417	1.86259	-0.2941	-0.84651	-1.07749	-1.21486	-1.17603	-1.29097	-1.00132
S29	0.18971	-0.55653	-0.29066	-0.60063	0.0493	0.26262	-0.15099	0.13611	4.16332	-1.12048	-1.22404	-1.075	-0.98141	-1.29097	-1.00132
S30	2.22382	2.06904	1.81294	1.53832	1.73093	1.69807	1.2178	0.72968	3.52494	0.52206	0.76066	1.30656	1.07123	1.20987	0.82582
S31	-0.29511	0.50818	-0.14073	-0.57856	-0.28171	-0.30488	-0.21461	0.22264	2.76722	-0.24209	-0.57533	-0.71531	-0.5927	-0.52697	-0.17764
S32	1.93096	1.54477	1.51363	0.93651	1.58488	1.74058	1.20289	0.73761	1.41248	3.33334	0.01523	0.56227	0.58871	0.10051	-0.64607
S33	-0.84011	-0.79866	-0.90498	-0.78621	-0.69394	-0.68807	-0.6592	-0.6868	-0.5004	0.83728	-0.70028	-1.00043	-0.80213	-0.70725	-0.55456
S34	-0.33723	-0.40596	-0.47114	-0.55253	-0.30082	-0.36879	-0.49906	-0.53535	0.53557	-0.1424	-0.60644	-0.61574	-0.49036	-0.44566	-0.2658
S35	0.66547	0.4208	0.65185	0.34794	0.66441	0.78826	0.38149	0.36091	3.26722	0.04736	-0.05082	0.25316	0.22858	0.53364	0.38211
S36	-0.12883	-0.22597	-0.35552	-0.34534	-0.07248	-0.03551	-0.13918	-0.07956	1.9475	-0.47026	-0.64605	-0.47828	-0.35282	-0.61376	-0.54266
S37	1.15529	0.91708	0.98576	0.4191	0.79428	0.83061	0.19776	-0.08	-0.15889	-0.02515	0.18242	1.03842	1.55658	0.57073	0.40538
S38	0.15505	-0.08203	0.18454	-0.21892	0.08564	0.17205	0.07534	-0.21478	-0.25302	1.45326	0.02646	0.21374	0.24356	0.49823	0.54667
S39	-1.19652	-1.3006	-1.31076	-1.34486	-1.15076	-1.01724	-0.97562	-0.90203	-0.81769	-0.81675	-0.46263	-1.10393	-1.06954	-0.95882	-0.73645
S40	1.21661	1.00776	1.20476	0.59495	0.98652	1.16803	2.45919	0.59135	0.07499	0.10492	0.1834	0.58122	0.49336	0.98401	1.09397
S41	0.32489	0.05203	0.10846	-0.21212	0.21853	0.36152	0.7063	1.47127	-0.35773	-0.29975	-0.32138	-0.155	-0.03683	0.02334	0.09444
S42	0.34228	0.27225	0.48373	-0.02742	0.49467	0.80778	3.3425	0.04666	-0.25134	-0.19227	-0.17221	0.00884	0.15381	0.25978	0.13528
S43	-1.21738	-0.7657	-1.28408	-1.15744	-1.35758	-1.24927	-1.1427	-0.74267	-0.75959	-0.7865	-0.98685	-1.23589	-1.0129	1.06743	-1.00132
S44	-0.77822	-0.53357	-0.77537	-0.68036	-1.00877	-0.97606	-0.92056	-0.80713	-0.65717	-0.5779	-0.59652	-0.68334	-0.17561	-0.19641	-0.32466
S45	-0.58657	-0.72513	-0.62599	-0.48147	-0.57743	-0.63535	-0.71851	-0.7267	-0.70801	-0.55183	-0.73241	-0.73824	-0.21959	0.11919	-0.06892
S46	0.17039	-0.16852	-0.06844	-0.28746	0.1547	0.18511	0.19541	1.52953	-0.37371	-0.33472	-0.38394	-0.21031	-0.15098	-0.00773	0.01042
S47	0.12302	0.06254	0.15187	-0.07441	0.20851	0.35146	0.30657	2.19362	0.14618	-0.28159	-0.23381	-0.01885	0.08301	0.29359	0.11527
S48	-0.22052	-0.4674	-0.46142	-0.54622	-0.17174	-0.0709	-0.13419	1.67338	-0.3152	-0.61941	-0.62297	-0.50229	-0.49788	-0.5725	-0.56804
S49	-0.21209	-0.22081	-0.19196	-0.35411	0.04888	0.11327	-0.03975	1.74886	0.16623	-0.39565	-0.48441	-0.32492	-0.16892	0.03357	-0.14658
S50	1.0249	0.71998	0.58856	0.17244	0.45896	0.48931	0.39006	0.18268	0.14064	0.16447	0.70608	2.22439	0.15982	0.33851	0.45983
S51	0.65212	0.98777	0.69909	0.36005	0.748	0.74304	0.71313	3.02819	0.4318	-0.04841	0.02204	0.36661	0.37825	0.26435	0.12192
S52	1.33447	1.59045	1.53014	1.08421	1.1547	1.2065	1.23086	1.20901	1.19158	2.67961	2.07682	0.68738	0.43899	0.17147	-0.12843

S53	0.11862	-0.03309	-0.18035	-0.36264	0.05608	-0.01451	-0.20211	-0.22432	-0.26989	0.42977	1.5657	-0.00394	-0.14151	-0.19252	-0.15739
S54	-0.06847	-0.22771	-0.06641	-0.31045	-0.10756	-0.06116	-0.11815	-0.23302	-0.16533	1.38889	0.14036	-0.22713	-0.31492	-0.34324	-0.27479
S55	1.18334	1.46249	1.86333	5.64711	1.49267	1.12846	0.38669	0.26604	0.16061	-0.08233	0.01844	0.41941	0.32453	0.41349	0.24711
S56	1.36007	1.31827	1.44768	0.72656	1.01817	1.22092	0.96655	0.62715	0.65783	2.90491	0.67979	1.11123	0.9106	1.35253	1.13971
S57	0.08335	0.09471	-0.07301	-0.1526	0.11205	0.17175	-0.04208	-0.16571	-0.07527	1.80384	0.26088	0.01641	-0.03457	-0.14103	-0.34352
S58	-0.8463	-0.67558	-1.06952	-1.09653	-0.90316	-0.9045	-0.79299	-0.66975	-0.58695	0.42962	-0.4174	-1.1043	-1.08196	-1.20532	-1.00132
S59	-0.14317	-0.25216	-0.19424	-0.423	-0.1586	-0.05977	-0.21233	-0.29699	-0.23594	-0.31928	0.05981	1.05068	-0.19698	-0.24871	-0.11841
S60	2.17475	1.93091	2.26351	1.41957	1.86566	1.81785	1.50359	0.97918	3.09207	2.08952	1.52376	2.16805	1.91721	2.84952	2.96155
S61	-0.45915	-0.70588	-0.62944	-0.78568	-0.34528	-0.3326	-0.30557	-0.51509	-0.42544	-0.41297	2.88435	-0.14482	-0.63635	-1.06594	-1.00132
S62	-0.79088	-0.66474	-0.97077	-1.03816	-0.69886	-0.62839	-0.09912	0.69123	-0.67473	-0.80292	-0.97186	-1.00456	-0.82831	-0.91162	-0.78347
S63	0.95932	1.70709	1.41043	0.72579	0.94853	0.96512	0.73066	0.6245	0.69282	3.49454	0.76955	1.1032	0.9958	1.35745	1.35287
S64	2.39934	2.00692	1.62211	1.07311	1.59944	1.61644	1.04921	0.77148	0.94673	3.60616	0.42761	0.89349	0.75342	0.60074	0.32049
S65	-0.76302	-0.57903	-0.60357	-0.75056	-0.42049	-0.347	-0.30173	-0.23999	1.38131	-0.51477	-0.71861	-0.66831	-0.27606	-0.73711	-1.00132
S66	0.53332	0.65728	0.79082	0.2767	0.50449	0.48646	0.34209	0.1816	0.67957	1.54503	0.18514	0.49733	0.36834	0.75054	0.8267
S67	-0.92843	0.08996	-0.84326	-1.03888	-0.99203	-0.93714	-0.77601	-0.29385	-0.14626	-0.27577	1.05886	-1.03706	-0.87612	-1.0007	-1.00132
S68	0.47065	0.38464	0.40855	0.20857	0.51977	0.52713	0.76978	1.21153	-0.24389	-0.21516	-0.0538	0.16297	0.04561	0.26965	0.20377
S69	1.05609	1.18731	0.77457	0.74573	0.38898	0.28268	0.03998	0.01675	0.09221	0.18805	0.6349	1.34013	2.54973	0.40585	0.47961
S70	-0.85054	-0.74808	-0.54924	-0.2544	-0.64406	-0.76941	-0.83425	-0.86309	-0.78697	-0.70348	-0.77302	-0.29035	-0.79369	-0.78157	-0.52978
S71	-0.66611	-0.62449	-0.58863	-0.15826	-0.63755	-0.84518	-0.91367	-0.83353	-0.70635	-0.6419	-0.43812	0.3968	-0.71864	-0.88933	-0.88382
S72	-0.05356	-0.02527	0.05118	0.2062	-0.34795	-0.40408	-0.53109	-0.57263	-0.43802	-0.12498	1.54783	-0.17345	-0.56863	-0.48645	-0.43686
S73	-1.292	-1.06077	-1.62615	-1.34154	-1.53362	-1.40026	-1.21071	-0.97618	-1.15452	-1.17583	-1.36325	-1.48085	-1.33482	-1.29097	-1.00132
S74	0.50673	0.79059	1.04101	1.29973	0.56769	0.42307	0.22488	0.00911	-0.04314	0.274	0.54018	1.98246	0.53611	1.03366	1.28164
S75	-1.02792	-0.98801	-0.95796	-0.53812	-1.02826	-0.92635	-0.86787	-0.91987	-0.8739	-0.74937	-0.85306	-0.95243	-0.43087	-0.68875	-0.58686
S76	0.24629	0.36463	0.30605	0.55503	-0.01406	-0.07475	-0.14327	-0.25039	-0.28838	-0.11437	0.05429	0.50411	3.60984	0.98459	0.03338
S77	-1.08384	-1.08991	-1.01135	-0.61427	-0.98907	-0.90301	-0.87506	-0.88915	-0.79097	-0.67916	-0.72084	-0.11087	-0.9129	-0.81942	-0.71604
S78	-0.92049	-0.29542	-0.5436	0.63977	-0.77244	-1.20265	-0.98737	-0.93425	-0.92937	-0.66323	-0.65576	-0.7854	-0.48924	-0.82625	-0.70029
S79	0.2475	0.91192	0.3434	0.174	-0.16005	-0.18909	-0.30668	-0.0925	-0.04614	0.10452	1.24961	0.11609	-0.18211	-0.0943	0.07923
S80	1.20038	1.42836	1.21862	1.47691	0.91497	0.57808	0.2489	0.02843	0.10824	0.43314	0.95422	1.83671	4.58866	1.36947	1.18817
S81	-0.27598	-0.22447	-0.06029	0.47129	-0.10283	-0.50716	-0.50592	-0.61991	-0.5256	-0.47836	-0.29628	0.14587	-0.15436	-0.08883	0.07879
S82	-1.78477	-2.02134	-1.82297	-1.69365	-1.53362	-1.40026	-1.21071	-1.2053	-1.15452	-1.17583	-1.40257	-1.68263	-1.33482	-0.05989	-1.00132
S83	-1.78477	-2.15381	-1.82297	-1.95147	-1.53362	-1.40026	-1.21071	-1.2053	-1.15452	-1.17583	-1.50365	-1.68263	-1.33482	-1.29097	-1.00132
S84	-1.78477	-2.15381	-1.82297	-1.95147	-1.53362	-1.40026	-1.21071	-1.2053	-1.15452	-1.17583	-1.50365	-1.68263	-1.33482	-1.29097	-1.00132
S85	1.62804	2.30162	1.27207	1.87436	0.29399	0.15507	0.11178	0.48088	0.40523	0.50845	0.96803	1.44823	1.32447	3.33289	2.68443
S86	-0.10684	-0.06724	0.13113	0.32818	-0.02738	-0.14598	-0.2465	-0.40133	-0.33394	-0.227	-0.01823	1.86224	-0.18929	0.23585	0.26214
S87	-0.91902	-0.60909	-0.72094	0.03223	-0.79057	-0.90512	-0.82388	-0.72402	-0.71095	-0.57831	-0.55654	-0.41616	-0.5737	0.07005	-0.25386
S88	0.26336	0.76844	0.36702	1.37094	-0.40916	-0.40424	-0.40388	-0.42537	-0.38673	-0.08533	0.36549	0.21657	-0.22202	0.06408	0.49917
S89	0.20651	0.18288	0.31964	0.29977	0.02779	-0.12787	-0.21259	-0.28798	-0.27521	-0.14668	0.84911	-0.03777	-0.00349	0.21555	0.34248
S90	-0.59818	-0.06986	0.41038	0.20768	0.14058	-0.52461	-0.37946	-0.65148	-0.55632	-0.47686	-0.32638	1.28166	0.47373	-0.0173	0.4599
S91	0.61509	0.63995	0.46656	0.93296	0.13647	0.11497	0.25927	0.02965	-0.04531	0.17618	0.50221	1.00054	0.80766	1.81629	2.57474
S92	-0.82746	-0.85956	-0.62564	-0.45848	-0.74918	-0.66474	-0.65339	-0.78926	-0.70901	-0.61908	-0.63939	-0.68425	-0.50647	-0.25678	-0.27886
S93	-0.64233	-0.58161	-0.65562	-0.36479	-0.69155	-0.62459	-0.52053	-0.6902	-0.55654	-0.405	-0.28164	0.18415	-0.5934	-0.49816	-0.46278
S94	-1.31426	-1.5721	-1.46761	-0.91628	-1.53362	-1.40026	-1.21071	-1.2053	-1.15452	-0.9493	-1.02437	-1.51761	-1.0567	-1.29097	-1.00132
S95	-0.85886	-0.7677	-0.78236	-0.27567	-0.96787	-0.85717	-0.92809	-0.95896	-0.85535	-0.68595	-0.6968	-0.89673	-0.22536	-0.5876	-0.61469
S96	0.20238	0.27927	0.18607	0.03804	-0.14406	-0.01214	-0.11617	-0.05493	-0.07352	0.01299	0.1633	0.60292	0.01607	0.39153	0.99458

S97	-0.76106	-1.01001	-0.68706	-0.58889	-0.7705	-0.67928	-0.71898	-0.69964	-0.71094	-0.62823	-0.71884	-0.62009	-0.54109	-0.4189	-0.18358
S98	-1.78477	-1.72355	-1.52313	-0.95522	-1.37196	-1.35398	-0.97273	-1.14558	-1.15452	-1.17583	-1.49457	-1.68263	-1.33482	-1.29097	-1.00132
S99	-1.3561	-1.55373	-1.42555	-1.06082	-1.34477	-1.19958	-1.07863	-1.14264	-1.02616	-0.97933	-1.23993	-1.4656	-1.1757	-1.12812	-1.00132
S100	-0.38751	-0.34276	-0.22607	-0.18181	-0.28201	-0.36968	-0.40575	-0.37747	-0.28196	-0.22643	-0.03734	1.11732	-0.15658	0.1549	0.21769
S101	-0.78563	-0.62746	-0.81623	-0.47456	-0.97633	-0.9577	-0.84642	-0.82654	-0.65419	-0.48136	-0.29016	-0.50576	0.03042	-1.05017	-0.96981
S102	-1.16446	-1.0744	-0.88445	-0.11508	-0.97525	-1.1101	-1.10049	-1.09733	-0.98025	-0.92553	-0.87188	-0.9391	-0.75011	-1.04236	-0.939
S103	0.50261	0.72743	0.70586	0.95891	0.36064	0.17653	0.08587	-0.14284	-0.04966	0.18732	0.69816	1.84631	0.36637	0.81145	0.76563
S104	-0.06487	-0.06842	-0.00719	-0.12322	-0.2398	-0.16255	-0.19291	-0.3381	-0.27124	-0.18653	-0.05081	1.47021	-0.08399	0.31244	0.43444
S105	-0.8323	-0.88159	-0.53007	-0.38081	-0.5944	-0.61132	-0.55734	-0.67999	-0.58333	-0.44342	-0.3515	0.24059	-0.09164	-0.2872	-0.24348
S106	-0.11122	0.75121	0.32867	1.36469	-0.15238	-0.60647	-0.51555	-0.52903	-0.46345	-0.28171	-0.10981	-0.05747	0.25173	0.13426	0.30803
S107	-1.06278	-0.90794	-1.27296	-0.42973	-1.22876	-1.40026	-1.21071	-1.18079	-1.09391	-0.8697	-0.92271	-0.94941	-0.33169	-1.08003	-1.00132
S108	-1.29895	-1.62573	-1.39677	-1.25146	-1.16554	-1.21048	-1.07158	-1.04946	-0.98197	-0.84682	-0.99517	-1.05028	-0.89098	-0.44755	-0.63757
S109	-0.56281	-0.58571	-0.79226	-0.49086	-0.71271	-0.72597	-0.58486	-0.61185	-0.62755	-0.50675	-0.23167	-0.13694	0.41833	0.48992	-0.27623

Sample No.	integrated bins														
	7.00 .. 6.98	6.98 .. 6.96	6.96 .. 6.94	6.94 .. 6.92	6.92 .. 6.90	6.90 .. 6.88	6.88 .. 6.86	6.86 .. 6.84	6.84 .. 6.82	6.82 .. 6.80	6.80 .. 6.78	6.78 .. 6.76	6.76 .. 6.74	6.74 .. 6.72	6.72 .. 6.70
S1	-1.04561	-0.97081	-0.98058	-1.06387	-1.03534	-1.03242	-0.99154	-1.15072	-1.10862	-1.00643	-0.78048	-0.41121	-0.60389	-0.8419	-0.70036
S2	1.62153	1.60292	1.72811	1.65946	1.53864	1.52806	1.6452	1.56958	1.62601	1.6171	1.5323	1.42205	1.51802	1.62284	1.75036
S3	-1.04561	-0.97081	-0.98058	-1.06387	-1.03534	-1.03242	-0.99154	-1.15072	-1.07589	-1.00643	-0.77298	-0.99265	-1.21371	-0.8419	-0.70036
S4	-0.54478	-0.52326	-0.53883	-0.21797	0.07233	-0.16991	-0.34134	-0.41107	-0.43529	-0.38289	-0.36937	-0.22945	-0.20494	-0.33461	-0.40291
S5	-1.04561	-0.97081	-0.98058	-1.06387	-1.03534	-1.03242	-0.99154	-0.54048	-0.83719	-0.96056	-0.81368	-1.01057	-1.11236	-0.8419	-0.70036
S6	0.01831	0.02736	0.11176	0.11829	0.04821	0.08032	0.06605	0.38165	0.21813	0.08025	0.08696	0.08959	0.23974	0.1178	-0.12445
S7	-0.00633	0.12351	0.13175	-0.00493	-0.03459	-0.07679	0.0241	0.27435	0.09811	-0.03837	0.02422	-0.10272	-0.05834	0.15541	0.26814
S8	-1.04561	-0.97081	-0.98058	-1.06387	-1.03534	-1.03242	-0.99154	-0.90112	-1.03592	-0.97354	-0.83988	-0.82329	-0.95775	-0.8419	-0.70036
S9	0.161	-0.0207	0.23695	0.42174	0.8366	0.4966	0.49449	0.01151	0.09573	0.03086	0.3504	0.64842	0.76534	0.28923	0.07428
S10	0.92836	0.91571	0.88995	0.71515	0.7613	0.78044	0.87914	0.93696	0.86102	0.95565	1.03033	1.04826	1.06737	1.06299	1.0606
S11	4.53647	4.78529	5.0734	5.07361	5.07235	5.17126	5.3163	5.24102	5.1302	5.47614	5.05716	4.57214	4.82659	5.87829	6.52988
S12	-0.66955	-0.80852	-0.60695	-0.71563	-0.71814	-0.92235	-0.7841	-0.51408	-0.52057	-0.73076	-0.78156	-0.9334	-0.96098	-0.8419	-0.70036
S13	-0.67868	-0.86946	-0.60464	-0.81343	-0.63317	-0.60617	-0.80061	-0.28635	-0.42249	-0.69195	-0.69328	-0.81311	-0.84652	-0.8419	-0.70036
S14	0.14385	0.24699	0.23779	0.14959	0.24899	0.07101	0.23686	0.581	0.55069	0.12139	0.16958	-0.01391	0.00302	0.17827	0.22663
S15	-1.04561	-0.97081	-0.98058	-1.06387	-1.03534	-1.03242	-0.99154	-1.15072	-1.10862	-1.00643	-1.13142	-0.84787	-0.8509	-0.8419	-0.70036
S16	0.25209	0.30681	0.37378	0.29394	0.23184	0.26958	0.40165	0.51522	0.46522	0.23697	0.37989	0.23629	0.10114	0.08659	0.11533
S17	-1.04561	-0.90661	-0.95539	-0.96633	-0.91283	-0.89818	-0.90967	-0.7492	-0.8063	-0.68502	-0.20738	0.04911	-0.30339	-0.8419	-0.70036
S18	0.5877	0.55291	0.43908	0.30355	0.40406	0.45672	0.25692	0.34493	0.40284	0.52656	0.80872	1.05568	1.05812	0.79035	0.50993
S19	2.37149	2.44097	2.40155	2.37635	2.4768	2.56968	2.51142	2.41075	2.93177	2.8405	2.5225	2.60781	2.58912	2.81695	2.64562
S20	-0.51294	-0.73031	-0.64126	-0.86089	-0.7655	-0.72877	-0.59382	-0.36651	-0.49338	-0.51221	-0.24965	-0.13721	-0.23868	-0.64088	-0.70036
S21	-1.04561	-0.97081	-0.98058	-1.06387	-1.03534	-1.03242	-0.99154	-1.15072	-1.10862	-1.00643	-1.20325	-1.32239	-1.21371	-0.8419	-0.70036
S22	0.72141	0.70803	0.77695	0.73349	0.71126	0.73949	0.7537	0.64431	0.71096	0.6505	0.69063	0.46525	0.54119	0.85842	0.9298
S23	0.16557	0.20001	0.20327	0.19912	0.27463	0.33124	0.44109	0.6112	0.53601	0.35645	0.20346	0.19974	0.40967	0.59308	0.54308
S24	-0.80527	-0.60983	-0.6037	-0.62075	-0.57392	-0.70885	-0.55077	-0.63178	-0.59883	-0.44771	-0.38075	-0.38613	-0.44525	-0.58935	-0.39237
S25	1.00148	0.97361	0.93069	0.76446	0.60384	0.70081	0.72957	0.78744	0.7238	0.75219	0.89178	0.91324	0.9022	1.02175	0.7076
S26	0.63643	0.71586	0.64409	0.6058	0.69529	0.68531	0.63812	0.65076	0.60682	0.55392	0.72431	0.82403	0.82875	0.73099	0.49209
S27	-0.70862	-0.70005	-0.68344	-0.69518	-0.92028	-0.76781	-0.71792	-0.51411	-0.64568	-0.73856	-0.61956	-0.42725	-0.53081	-0.81085	-0.70036
S28	-1.04561	-0.97081	-0.98058	-1.06387	-1.03534	-1.03242	-0.99154	-1.01954	-1.10862	-1.00643	-0.86569	-0.96043	-1.21371	-0.8419	-0.70036

S29	-1.04561	-0.97081	-0.98058	-1.06387	-1.03534	-1.03242	-0.99154	-1.15072	-1.10862	-1.00643	-1.20325	-0.65728	-0.90506	-0.8419	-0.70036
S30	0.71891	0.61966	0.62691	0.60619	0.58267	0.69737	0.63965	0.59667	0.52365	0.68408	1.07637	1.39145	1.24105	0.70578	0.42656
S31	-0.218	-0.14339	-0.09498	0.00892	-0.07423	-0.156	-0.14082	0.48941	0.36722	0.09455	0.0671	-0.3541	-0.35378	-0.20735	-0.12905
S32	-0.65044	-0.57807	-0.33908	-0.24662	-0.2097	-0.22958	-0.5747	-0.6679	-0.57741	-0.43403	0.26173	0.97302	0.76207	-0.45608	-0.70036
S33	-0.48588	-0.47203	-0.42171	-0.60955	-0.57039	-0.47252	-0.39221	-0.39035	-0.4429	-0.47753	-0.48197	-0.63185	-0.64993	-0.55351	-0.60524
S34	-0.3482	-0.25595	-0.21476	-0.20304	-0.2729	-0.18092	-0.15221	0.08385	-0.17636	-0.06013	-0.07848	-0.22273	-0.22072	-0.20258	-0.27641
S35	0.27027	0.25572	0.26822	0.47961	0.2859	0.3752	0.53506	0.68539	0.48112	0.51451	0.51965	0.67256	0.77483	0.53387	0.46632
S36	-0.61095	-0.56731	-0.2952	-0.24645	-0.53601	-0.44099	-0.29726	-0.27758	-0.29416	-0.25987	-0.12979	-0.09085	-0.09443	-0.30815	-0.38981
S37	0.16391	0.00292	-0.0637	-0.02935	-0.09268	-0.2128	-0.05322	0.07003	0.10068	0.23754	0.63346	0.86919	0.54011	0.37563	0.15743
S38	0.5012	0.50602	0.50809	0.4303	0.33392	0.35252	0.32831	0.37919	0.34325	0.43595	0.4223	0.37841	0.46369	0.55193	0.55864
S39	-0.66398	-0.72065	-0.76066	-0.85532	-0.79422	-0.75345	-0.7469	-0.68265	-0.69948	-0.76041	-0.88599	-0.96791	-0.97714	-0.80104	-0.70036
S40	1.15094	1.07734	1.18913	1.14405	1.21427	1.21214	1.3551	1.32868	1.18872	1.26004	1.20444	1.14443	1.17886	1.21554	1.36751
S41	0.09505	0.11782	0.11787	0.16537	0.12318	0.04841	0.12794	0.23315	0.29356	0.27643	0.17506	0.26116	0.28946	0.32666	0.23029
S42	0.31236	0.21763	0.09485	0.06002	0.17592	0.04392	0.10603	0.21282	0.18416	0.22041	0.24333	0.35082	0.35053	0.19721	0.13019
S43	-1.04114	-0.97081	-0.98058	-1.05149	-0.93189	-0.92403	-0.90974	-0.63368	-0.71491	-0.87205	-0.96832	-1.23285	-1.1408	-0.8419	-0.70036
S44	-0.19998	-0.42684	-0.62086	-0.57437	-0.46782	-0.71349	-0.70477	-0.44224	-0.51236	-0.44109	-0.59017	-0.71723	-0.79212	-0.60512	-0.6447
S45	-0.01223	-0.07847	-0.11334	-0.21323	-0.23896	-0.28265	-0.24216	-0.29716	-0.28865	-0.09731	-0.30732	-0.47531	-0.41501	-0.19859	-0.095
S46	0.00296	0.10334	0.03622	0.00901	0.08906	0.1114	0.12701	0.13188	0.19233	0.11268	0.14083	0.14502	0.1591	0.19392	0.1221
S47	0.29295	0.21112	0.23351	0.17079	0.26341	0.2273	0.217	0.15721	0.18153	0.12811	0.18146	0.31617	0.38978	0.20693	0.15604
S48	-0.58942	-0.68487	-0.58731	-0.69477	-0.51388	-0.54766	-0.54208	-0.61254	-0.58796	-0.66447	-0.47478	-0.26862	-0.38246	-0.55114	-0.70036
S49	-0.17628	-0.36287	-0.26663	-0.21872	-0.3134	-0.36235	-0.32977	-0.39727	-0.38939	-0.39105	-0.34012	-0.07211	-0.0154	-0.29664	-0.55092
S50	0.37362	0.54417	0.56282	0.47675	0.70398	0.67846	0.68411	0.71752	0.75508	0.8919	0.95869	0.87176	0.75336	0.77839	0.86509
S51	0.10552	0.16398	0.02677	-0.05984	0.07134	0.04285	-0.03362	0.39382	0.47492	0.50278	0.69206	0.68992	0.5704	0.35761	0.07077
S52	0.09069	0.22904	0.37046	0.75377	0.76559	0.62719	0.54948	1.09026	1.10339	1.23514	1.79031	1.25233	0.82059	0.27481	0.14019
S53	-0.16776	-0.20695	-0.24351	-0.15888	-0.20274	-0.2495	-0.22166	-0.14513	-0.06241	-0.13999	0.15942	0.23185	0.06039	0.02831	-0.11435
S54	-0.3649	-0.2222	-0.30941	-0.22361	-0.15032	-0.2163	-0.13685	0.09288	-0.04003	-0.00395	0.12609	0.0464	-0.02859	-0.16492	-0.17933
S55	0.31568	0.4594	0.5193	0.473	0.3797	0.41599	0.4574	0.8275	0.57938	0.54431	0.74048	0.98134	0.76622	0.53822	0.30753
S56	1.14487	1.16913	1.26852	1.23551	1.16684	1.16413	1.10103	1.22903	1.32809	1.34429	1.50963	1.63553	1.49486	1.33241	1.38396
S57	-0.25199	-0.40152	-0.26454	-0.2362	-0.22003	-0.26252	-0.29771	-0.19467	-0.17442	-0.09389	0.16476	0.30782	0.10968	-0.15464	-0.25692
S58	-1.00763	-0.95463	-0.98058	-1.01434	-0.9442	-0.97877	-0.97614	-0.67173	-0.75207	-0.92093	-0.76013	-0.91448	-1.03855	-0.8419	-0.70036
S59	-0.2794	-0.14274	-0.10152	0.00389	-0.13659	-0.11062	-0.1274	-0.06266	-0.10231	0.02714	0.0542	0.21164	0.1379	0.15299	0.05716
S60	3.07928	3.08251	3.12808	3.06684	2.89846	2.81392	2.9019	2.76115	2.84776	2.85572	2.78826	2.6814	2.81976	2.97893	3.13041
S61	-1.04561	-0.97081	-0.98058	-1.06387	-1.03534	-1.03242	-0.99154	-1.15072	-1.10862	-1.00643	-0.90558	-0.58159	-0.80055	-0.8419	-0.70036
S62	-0.6917	-0.76191	-0.7609	-0.72988	-0.77107	-0.71127	-0.59768	-0.35092	-0.528	-0.69205	-0.73434	-0.76615	-0.66889	-0.75231	-0.70036
S63	1.11675	1.09285	1.19041	1.09104	1.1489	1.04245	1.04147	1.2803	1.33316	1.21826	1.32413	1.31368	1.32168	1.1326	0.99901
S64	0.2401	0.15048	0.17299	0.50493	0.82533	0.78948	0.60629	0.73294	0.59617	0.77893	1.37307	1.71901	1.31471	0.63919	0.33365
S65	-1.04561	-0.97081	-0.98058	-1.06387	-1.03534	-1.03242	-0.99154	-1.15072	-1.10242	-1.00643	-0.74546	-0.53518	-0.39548	-0.8419	-0.70036
S66	0.89829	0.90248	0.96389	0.83703	0.82104	0.76088	0.82176	1.05865	1.00457	1.09621	1.06944	0.82391	0.80286	0.98264	0.97151
S67	-1.04561	-0.97081	-0.98058	-0.9531	-0.9355	-1.01604	-0.98396	-0.39172	-0.44858	-0.8943	-0.76009	-0.98386	-1.00117	-0.8419	-0.70036
S68	0.12485	0.10839	0.43322	0.36029	0.06217	0.09205	0.19961	0.11958	0.07294	0.28519	0.29065	0.39084	0.34492	0.17443	0.19517
S69	0.39299	0.22101	0.29226	0.45517	0.21897	0.20862	0.05917	0.42755	0.61541	0.35524	0.37715	0.5307	0.50996	-0.0192	-0.18944
S70	-0.50399	-0.43922	-0.44206	-0.18782	-0.16387	-0.05578	-0.28855	-0.72573	-0.66835	-0.54349	-0.72282	-0.71132	-0.59017	-0.58549	-0.63763
S71	-0.74689	-0.8125	-0.64772	-0.46808	-0.61136	-0.59895	-0.79017	-0.89704	-0.75564	-0.8024	-0.69991	-0.7742	-0.68674	-0.8419	-0.70036
S72	-0.51006	-0.43367	-0.57735	0.12114	-0.40992	-0.06333	-0.24966	-0.54779	-0.48153	-0.50736	-0.48048	-0.33242	-0.19174	-0.6467	-0.70036

S73	-1.04561	-0.97081	-0.98058	-1.06387	-1.03534	-1.03242	-0.99154	-1.15072	-1.10862	-1.00643	-1.20325	-1.32239	-1.21371	-0.8419	-0.70036
S74	1.57266	1.59108	1.56676	1.64361	1.88945	1.78049	1.88361	1.16982	1.22562	1.15201	0.92649	0.77754	1.08144	1.33766	1.39005
S75	-0.50224	-0.43651	-0.51471	-0.40566	-0.4184	-0.31277	-0.47284	-0.61798	-0.54562	-0.64835	-0.79231	-0.73454	-0.61269	-0.49144	-0.57789
S76	0.48541	0.37965	0.35289	0.6858	0.27584	0.27997	0.10737	-0.08013	-0.07897	-0.00842	0.04772	0.09374	0.16119	0.01003	-0.141
S77	-0.63918	-0.9262	-0.6972	-0.56093	-0.68034	-0.7199	-0.80879	-0.8218	-0.72235	-0.92235	-0.97697	-0.98434	-0.74822	-0.83596	-0.70036
S78	-0.78287	-0.78024	-0.79775	-0.92433	-0.81949	-0.79953	-0.90191	-1.15072	-1.05136	-0.95563	-1.19091	-1.19953	-1.21371	-0.8419	-0.70036
S79	0.37756	0.27389	0.35649	0.36745	0.48524	0.39516	0.47022	0.43311	0.56019	0.31009	0.24528	0.03289	0.05241	0.23099	0.10609
S80	1.17877	1.09476	1.02207	1.0706	1.04803	1.25904	1.18376	0.65885	0.77258	0.77789	0.83407	0.89275	0.9259	0.69128	0.39192
S81	0.35886	0.1747	0.08315	0.15667	0.04241	0.01281	0.16961	0.05079	0.04455	0.08321	-0.12007	-0.10099	-0.08199	0.21577	0.15396
S82	-1.04561	-0.97081	-0.98058	-1.06387	-1.03534	-1.03242	-0.99154	-1.15072	-1.10862	-1.00643	-1.20325	-1.32239	-1.21371	-0.8419	-0.70036
S83	-1.04561	-0.97081	-0.98058	-1.06387	-1.03534	-1.03242	-0.99154	-1.15072	-1.10862	-1.00643	-1.20325	-1.32239	-1.21371	-0.8419	-0.70036
S84	-1.04561	-0.97081	-0.98058	-1.06387	-1.03534	-1.03242	-0.99154	-1.15072	-1.10862	-1.00643	-1.20325	-1.32239	-1.21371	-0.8419	-0.70036
S85	2.66073	2.70773	2.38151	2.37792	2.22951	2.25402	2.03046	1.86144	1.72274	1.41788	1.07173	1.00325	0.78964	0.61779	0.35131
S86	0.21956	0.1537	0.18338	0.24877	0.14501	0.30252	0.23939	0.12671	0.17799	0.136	0.02356	-0.04894	0.18285	0.06164	0.04941
S87	-0.15373	-0.1485	-0.25033	-0.24871	-0.39357	-0.37981	-0.33232	-0.53351	-0.54908	-0.66056	-0.86506	-0.89516	-0.84724	-0.81289	-0.70036
S88	0.83536	0.73996	0.63241	0.59235	0.50626	0.57153	0.4181	0.31261	0.3038	0.2944	0.11708	-0.02089	0.03669	0.14269	0.19095
S89	0.48981	0.44005	0.48469	0.4622	0.25974	0.31069	0.35052	0.4131	0.38178	0.32489	0.27622	0.2443	0.3688	0.34551	0.30943
S90	0.37847	0.58221	0.16749	0.10184	0.14993	0.23652	0.18374	-0.07923	0.26667	0.09191	-0.0915	-0.18836	0.15902	0.34738	0.14962
S91	1.6007	1.57649	1.42925	1.39721	1.37502	1.34756	1.141	1.11267	1.28485	1.37879	1.03316	0.79088	0.59413	0.92285	0.82089
S92	0.04227	-0.1396	-0.2236	-0.03675	-0.03727	0.04137	-0.12537	-0.40365	-0.24823	-0.30536	-0.40726	-0.45924	-0.38085	-0.25473	-0.23879
S93	-0.32031	-0.38684	-0.46057	-0.52228	-0.38324	-0.47008	-0.32853	-0.53283	-0.4847	-0.53127	-0.51314	-0.51091	-0.45782	-0.64734	-0.70036
S94	-1.04561	-0.97081	-0.98058	-1.06387	-1.03534	-1.03242	-0.99154	-1.15072	-1.10862	-1.00643	-1.20325	-1.32239	-1.21371	-0.8419	-0.70036
S95	-0.69788	-0.76839	-0.69093	-0.69198	-0.87843	-0.83393	-0.73741	-0.83771	-0.83715	-0.76877	-0.85927	-0.90322	-0.86478	-0.8419	-0.70036
S96	0.99192	1.16833	0.99159	1.02216	1.17264	1.01905	1.052	1.06886	0.98198	0.92737	0.63776	0.43072	0.46164	0.8124	1.03825
S97	-0.18897	0.0474	-0.10814	-0.17401	-0.08303	-0.10359	-0.07906	-0.07917	-0.17685	-0.11402	-0.30128	-0.50155	-0.32253	-0.04368	0.07499
S98	-1.04561	-0.97081	-0.98058	-1.06387	-1.03534	-1.03242	-0.99154	-1.15072	-1.10862	-1.00643	-1.20325	-1.32239	-1.21371	-0.8419	-0.70036
S99	-0.95604	-0.87953	-0.88575	-0.90128	-0.8704	-0.96986	-0.95926	-1.0275	-0.9132	-0.96245	-1.09353	-1.10549	-1.06953	-0.74052	-0.70036
S100	0.24873	0.1495	0.10481	0.27252	0.23627	0.25065	0.09285	0.02469	0.22556	0.16496	-0.02366	-0.2004	-0.05104	0.00934	-0.10255
S101	-0.96163	-0.91275	-0.78109	-0.71058	-0.62854	-0.62516	-0.66244	-0.85011	-0.72484	-0.72335	-0.94254	-0.89003	-0.92117	-0.8419	-0.70036
S102	-0.83314	-0.9328	-0.92825	-0.81565	-0.92848	-0.9054	-0.92183	-1.15072	-1.07504	-1.00643	-1.06798	-1.07973	-1.1522	-0.8419	-0.70036
S103	0.75312	0.71409	0.71085	0.65989	0.52026	0.61811	0.74993	0.54974	0.50102	0.59732	0.457	0.49636	0.55033	0.31858	0.19361
S104	0.64789	0.61469	0.61402	0.57986	0.6831	0.62811	0.41258	0.39935	0.3705	0.39145	0.24635	0.22178	0.15432	0.30941	0.18575
S105	-0.09462	-0.0189	-0.36966	-0.30187	-0.25993	-0.32164	-0.31323	-0.43581	-0.29478	-0.30152	-0.32965	-0.37534	-0.3748	-0.38799	-0.45487
S106	0.35554	0.1982	0.25186	0.16448	0.30026	0.25934	0.25522	0.03036	0.16919	0.16399	-0.168	-0.46212	-0.31479	-0.31474	-0.2888
S107	-1.04561	-0.97081	-0.98058	-1.06387	-1.03534	-1.03242	-0.99154	-1.15072	-1.10862	-1.00643	-1.20325	-1.32239	-1.21371	-0.8419	-0.70036
S108	-0.66409	-0.65696	-0.7431	-0.75775	-0.70287	-0.62357	-0.66841	-0.83367	-0.77721	-0.61993	-0.96278	-1.13206	-1.06339	-0.8299	-0.70036
S109	-0.20522	-0.31873	-0.3903	-0.31869	-0.29234	-0.2339	-0.22556	-0.35738	-0.42379	-0.37594	-0.48164	-0.54958	-0.67958	-0.49041	-0.68043

Sample No.	integrated bins														
	6.70 .. 6.68	6.68 .. 6.66	6.66 .. 6.64	6.64 .. 6.62	6.62 .. 6.60	6.60 .. 6.58	6.58 .. 6.56	6.56 .. 6.54	6.54 .. 6.52	6.52 .. 6.50	6.50 .. 6.48	6.48 .. 6.46	6.46 .. 6.44	6.44 .. 6.42	6.42 .. 6.40
S1	-0.66923	-0.681	-0.67616	-0.65686	-0.63959	-0.61233	-0.60602	-0.62333	-0.5677	-0.5514	-0.57309	-1.00397	-1.03982	-0.48299	-0.48358
S2	1.69201	1.73341	1.73325	1.65831	1.71665	1.65349	1.64661	1.55735	1.6384	1.72293	1.75723	0.92124	0.72842	1.48552	1.57722
S3	-0.66923	-0.681	-0.67616	-0.65686	-0.63959	-0.61233	-0.60602	-0.62333	-0.5677	-0.5514	-0.57309	-1.00397	-1.03982	-0.48299	-0.48358
S4	-0.34099	-0.32057	-0.28997	-0.25779	-0.25581	-0.1918	-0.1533	-0.2922	-0.29149	-0.12467	-0.13815	-0.52821	-0.65112	-0.11725	-0.03233

S5	-0.66923	-0.681	-0.67616	-0.65686	-0.63959	-0.61233	-0.60602	-0.62333	-0.5677	-0.5514	-0.57309	-1.00397	-1.03982	-0.48299	-0.48358
S6	-0.04025	-0.09138	-0.10115	-0.0633	-0.02114	0.01612	-0.18268	-0.01675	-0.06574	-0.09199	-0.1676	-0.19716	-0.20897	-0.17146	-0.09373
S7	0.27984	0.23941	0.2901	0.33274	0.32474	0.31158	0.25212	0.33213	0.41056	0.4809	0.41607	0.5561	0.37087	0.32638	0.34721
S8	-0.66923	-0.681	-0.67616	-0.65686	-0.63959	-0.61233	-0.60602	-0.62333	-0.5677	-0.5514	-0.57309	-0.94245	-1.03982	-0.48299	-0.48358
S9	-0.00174	0.15521	-0.02853	-0.00836	0.0665	-0.07812	0.02076	-0.14821	-0.1048	-0.00117	0.464	0.00168	0.14025	-0.11911	-0.13042
S10	1.00755	0.92278	1.05184	0.99289	1.04169	1.14603	0.85396	0.85202	0.927	0.77634	0.81093	0.38991	0.0163	0.88114	0.85228
S11	6.85671	6.93676	6.91893	7.051	7.15606	7.3051	7.21132	7.37944	7.66201	7.82955	7.71068	5.96991	5.13426	8.27731	8.30154
S12	-0.66923	-0.681	-0.67616	-0.65686	-0.63959	-0.61233	-0.60602	-0.62333	-0.5677	-0.5514	-0.57309	-0.74439	-1.03982	-0.48299	-0.48358
S13	-0.66923	-0.681	-0.67616	-0.65686	-0.63959	-0.61233	-0.60602	-0.62333	-0.5677	-0.5514	-0.57309	-0.86602	-1.03982	-0.48299	-0.48358
S14	0.23586	0.29574	0.20768	0.27968	0.15432	0.21634	0.12277	0.17604	0.31572	0.21212	0.24281	-0.17676	-0.18311	0.19065	0.19681
S15	-0.66923	-0.681	-0.67616	-0.65686	-0.63959	-0.61233	-0.60602	-0.62333	-0.5677	-0.5514	-0.57309	-1.00397	-1.03982	-0.48299	-0.48358
S16	0.16086	0.21319	0.16615	0.11195	0.16577	0.12372	0.16443	-0.00236	0.03999	0.05468	0.02519	-0.25119	-0.48619	0.01359	0.05157
S17	-0.66923	-0.681	-0.67616	-0.65686	-0.63959	-0.61233	-0.60602	-0.62333	-0.5677	-0.5514	-0.57309	-0.97666	-1.02375	-0.48299	-0.48358
S18	0.36428	0.36553	0.39405	0.33786	0.51416	0.40562	0.71576	0.33471	0.40373	0.32808	0.4468	-0.12751	0.02692	0.35834	0.35054
S19	2.50623	2.45683	2.56582	2.47273	2.59168	2.6805	2.92898	2.6122	2.52163	2.55135	2.51836	1.55135	2.36351	2.4869	2.4666
S20	-0.66923	-0.681	-0.67616	-0.65686	-0.63959	-0.61233	-0.60602	-0.62333	-0.5677	-0.5514	-0.57309	-0.81747	-0.93648	-0.48299	-0.48358
S21	-0.66923	-0.681	-0.67616	-0.65686	-0.63959	-0.61233	-0.60602	-0.62333	-0.5677	-0.5514	-0.57309	-1.00397	-1.03982	-0.48299	-0.48358
S22	0.93914	0.96401	0.99062	1.07015	1.02776	1.05597	0.95512	1.38173	1.0298	1.04883	1.12013	0.53841	0.3976	1.09022	1.08342
S23	0.50605	0.48689	0.52221	0.58859	0.50467	0.51336	0.40062	0.71164	0.65557	0.469	0.42025	-0.16304	0.57982	0.46207	0.44151
S24	-0.65405	-0.44522	-0.47774	-0.32466	-0.44591	-0.56172	-0.47	-0.47482	-0.49637	-0.53216	-0.4039	-0.77999	-0.69369	-0.45459	-0.48358
S25	0.73617	0.71591	0.6019	0.77604	0.6649	0.64904	0.68069	0.51794	0.46026	0.42484	0.37365	-0.06356	0.14348	0.38907	0.35468
S26	0.44532	0.45107	0.61431	0.59247	0.54619	0.36603	0.29948	0.33229	0.1515	0.05745	0.03016	-0.44073	-0.01799	0.06131	0.07365
S27	-0.66923	-0.681	-0.67616	-0.65686	-0.63959	-0.61233	-0.60602	-0.62333	-0.5677	-0.5514	-0.57309	-0.93263	-1.03982	-0.48299	-0.48358
S28	-0.66923	-0.681	-0.67616	-0.65686	-0.63959	-0.61233	-0.60602	-0.62333	-0.5677	-0.5514	-0.57309	-1.00397	-1.03982	-0.48299	-0.48358
S29	-0.66923	-0.681	-0.67616	-0.65686	-0.63959	-0.61233	-0.60602	-0.62333	-0.5677	-0.5514	-0.57309	-1.00397	-1.03982	-0.48299	-0.48358
S30	0.37416	0.30346	0.28638	0.41901	0.27321	0.2134	0.19289	0.14122	0.12038	0.21204	0.12703	-0.27821	-0.30992	0.06045	-0.0166
S31	-0.04171	-0.09291	-0.04516	-0.04628	-0.08775	-0.02533	-0.11606	-0.15867	-0.08865	-0.0598	-0.08856	-0.5816	-0.50977	-0.05938	-0.09493
S32	-0.66923	-0.681	-0.67616	-0.65686	-0.63959	-0.61233	-0.60602	-0.62333	-0.5677	-0.5514	-0.57309	-1.00397	-0.97403	-0.48299	-0.48358
S33	-0.55894	-0.56781	-0.59395	-0.65131	-0.57979	-0.59235	-0.60602	-0.5521	-0.5677	-0.5514	-0.57309	-0.63104	-0.68201	-0.48299	-0.48358
S34	-0.17611	-0.07219	-0.23026	-0.15161	-0.2037	-0.09309	-0.06902	-0.12077	-0.14714	-0.11359	-0.0661	0.01907	-0.49866	-0.11826	-0.04482
S35	0.48888	0.52362	0.43585	0.5227	0.50025	0.4103	0.40193	0.35804	0.43801	0.34008	0.57192	-0.1612	0.14399	0.31776	0.51337
S36	-0.5118	-0.37013	-0.20301	-0.25541	-0.23652	-0.30997	-0.40071	-0.34112	-0.2451	-0.35036	-0.31106	-0.67404	-0.58713	-0.28659	-0.29365
S37	0.14802	0.23331	0.17443	0.09349	0.04608	0.06322	0.02307	0.15392	0.05512	0.01028	0.05576	0.00989	-0.02582	0.03602	0.08356
S38	0.57444	0.45455	0.39493	0.38526	0.3987	0.33422	0.44407	0.37769	0.35766	0.2743	0.19602	0.02257	0.42049	0.33281	0.26487
S39	-0.66923	-0.63808	-0.67616	-0.65686	-0.63959	-0.61233	-0.60602	-0.62333	-0.5677	-0.5514	-0.57309	-0.08111	-0.95906	-0.48299	-0.48358
S40	1.29215	1.34853	1.18773	1.20784	1.28677	1.39329	1.69357	1.2057	1.36311	1.45968	1.52566	1.11917	0.98438	1.35593	1.38763
S41	0.21034	0.29944	0.30657	0.25476	0.1352	0.37325	0.72728	0.09033	0.29543	0.3125	0.15822	0.54262	0.03021	0.26336	0.27958
S42	0.10788	0.08812	-0.03541	0.03774	-0.05382	0.35009	0.07413	0.02196	0.07303	0.14862	0.04219	0.53308	-0.33544	-0.00705	-0.06547
S43	-0.66923	-0.681	-0.67616	-0.65686	-0.63959	-0.61233	-0.60602	-0.62333	-0.5677	-0.5514	-0.57309	-1.00397	-1.03982	-0.48299	-0.48358
S44	-0.52194	-0.58409	-0.50833	-0.63131	-0.54534	-0.52342	-0.60602	-0.62333	-0.5677	-0.5514	-0.57309	-0.89422	-1.0285	-0.48299	-0.48358
S45	-0.06208	-0.11605	-0.05853	-0.16845	-0.17865	-0.08904	-0.13014	-0.09284	-0.0145	-0.10431	-0.08535	-0.68492	-0.73685	-0.10308	-0.10094
S46	0.16123	0.18637	0.04312	0.10167	0.03717	0.18966	0.39417	0.05761	0.09244	0.11687	0.13443	0.49166	-0.14766	0.08944	0.10747
S47	0.16841	0.08504	0.08179	0.07223	0.1065	0.16083	0.9319	0.07789	0.0773	0.03013	0.12434	-0.0829	0.23045	0.0009	-0.0077
S48	-0.66923	-0.681	-0.67616	-0.65686	-0.63959	-0.61233	-0.33908	-0.62333	-0.5677	-0.5514	-0.57309	-0.41219	-0.75484	-0.48299	-0.48358

S49	-0.66923	-0.64605	-0.59699	-0.64264	-0.63959	-0.59831	-0.2809	-0.62333	-0.5677	-0.5514	-0.57309	-1.00397	-0.48024	-0.48299	-0.48358
S50	0.8882	0.97663	1.1247	1.1373	1.13995	1.08038	0.93932	1.44121	1.07438	1.12538	1.20512	1.10456	1.20454	0.83219	0.92291
S51	-0.01748	0.14631	0.11848	0.04431	0.0655	0.04586	0.30301	-0.05267	-0.02154	-0.13414	-0.06676	-0.372	-0.49695	-0.06272	-0.06029
S52	0.2718	0.43735	0.73856	0.83225	0.39265	0.20725	0.08731	0.39003	0.15635	0.33101	0.35662	0.05169	0.45096	0.04482	0.02511
S53	-0.19518	-0.13367	-0.12617	-0.06451	-0.15852	-0.1407	-0.22843	0.302	-0.2135	-0.20859	-0.29939	-0.6739	-0.7707	-0.26334	-0.23755
S54	-0.2645	-0.15633	-0.15859	-0.16177	-0.18701	-0.16357	-0.28447	-0.12625	-0.26487	-0.22454	-0.29161	-0.33584	0.1598	-0.28866	-0.29816
S55	0.43001	0.49259	0.39618	0.45061	0.71781	0.39098	0.36783	0.29422	0.35013	0.39451	0.43191	0.72488	-0.34517	0.26669	0.25779
S56	1.21979	1.23753	1.36134	1.30005	1.25726	1.28255	1.21522	1.21842	1.24124	1.25193	1.06055	0.44245	0.678	1.07445	1.06658
S57	-0.2638	-0.3055	-0.28313	-0.37	-0.33971	-0.36396	-0.39367	-0.19651	-0.46851	-0.36356	-0.38905	-0.70119	-0.48344	-0.41701	-0.41105
S58	-0.66923	-0.681	-0.67616	-0.65686	-0.63959	-0.61233	-0.60602	-0.62333	-0.5677	-0.5514	-0.57309	-0.40492	-1.03982	-0.48299	-0.48358
S59	0.08049	0.10102	0.19673	0.11001	0.12482	0.04423	0.05093	0.24685	0.11907	0.00733	0.16483	-0.09775	-0.10294	0.1997	0.14012
S60	3.05973	2.95704	2.93175	2.95218	2.95441	2.80116	2.81625	2.7284	2.65968	2.55381	2.63309	1.66142	1.92459	2.59777	2.52627
S61	-0.66923	-0.681	-0.67616	-0.65686	-0.63959	-0.61233	-0.60602	-0.62333	-0.5677	-0.5514	-0.57309	-1.00397	-1.03982	-0.48299	-0.48358
S62	-0.66923	-0.681	-0.67616	-0.65686	-0.63959	-0.6051	-0.57132	-0.62333	-0.5677	-0.5514	-0.57309	-0.34352	-0.86727	-0.48299	-0.48358
S63	0.99309	0.97037	0.93737	0.86841	0.85193	0.79426	0.58393	0.77999	0.63241	0.70987	0.59552	-0.06077	0.15984	0.50564	0.48575
S64	0.26849	0.38139	0.51513	0.39229	0.5571	0.44987	0.37416	0.58314	0.48999	0.71311	0.80861	0.51792	0.21594	0.37206	0.33338
S65	-0.66923	-0.681	-0.67616	-0.65686	-0.63959	-0.61233	-0.60602	-0.62333	-0.5677	-0.5514	-0.57309	-1.00397	-0.83079	-0.48299	-0.48358
S66	1.04049	0.99708	0.93183	0.98204	0.91451	0.94282	1.28902	1.35579	0.8913	0.88355	0.85406	0.40584	0.61296	0.93451	0.94958
S67	-0.66923	-0.681	-0.67616	-0.65686	-0.63959	-0.61233	-0.60602	-0.62333	-0.5677	-0.5514	-0.57309	-1.00397	-1.03982	-0.48299	-0.48358
S68	0.15111	-0.01892	0.03045	-0.01254	0.00661	0.11133	0.22629	0.04836	0.0155	-0.00288	-0.02194	-0.13498	-0.23848	-0.02485	-0.04941
S69	-0.36582	-0.38953	-0.4383	-0.45616	-0.51743	-0.55619	-0.60602	-0.37134	-0.5677	-0.5514	-0.57309	0.51962	1.17712	-0.48299	-0.48358
S70	-0.61709	-0.57685	-0.67616	-0.64242	-0.63959	-0.61233	-0.60602	-0.59758	-0.5677	-0.42923	-0.35319	0.72418	0.38719	-0.48299	-0.48358
S71	-0.66923	-0.681	-0.67616	-0.65686	-0.63959	-0.61233	-0.60602	-0.62333	-0.5677	-0.5514	-0.57309	0.91877	1.00412	-0.48299	-0.48358
S72	-0.66923	-0.681	-0.67616	-0.65686	-0.63959	-0.61233	-0.60602	-0.62333	-0.5677	-0.5514	-0.57309	0.43242	0.45995	-0.48299	-0.48358
S73	-0.66923	-0.681	-0.67616	-0.65686	-0.63959	-0.61233	-0.60602	-0.62333	-0.5677	-0.5514	-0.57309	1.60558	-1.03982	-0.48299	-0.48358
S74	1.15536	1.36546	1.04288	0.98301	0.97338	0.94172	0.87776	0.97226	0.85534	0.90816	0.98008	1.35424	1.79206	0.87975	0.72267
S75	-0.59634	-0.625	-0.55706	-0.6429	-0.59361	-0.50025	-0.59276	-0.39576	-0.52214	-0.47621	-0.50389	0.80792	-0.01064	-0.48299	-0.48358
S76	0.06729	0.0153	-0.22238	-0.12629	-0.13909	-0.13365	-0.328	-0.11948	0.06512	-0.17842	-0.21344	-0.34715	0.49737	-0.3156	-0.2284
S77	-0.66923	-0.681	-0.67616	-0.65686	-0.63959	-0.61233	-0.60602	-0.62333	-0.5677	-0.5514	-0.57309	0.57969	-0.28383	-0.48299	-0.48358
S78	-0.66923	-0.681	-0.67616	-0.65686	-0.63959	-0.61233	-0.60602	-0.62333	-0.5677	-0.5514	-0.57309	-0.4724	0.16613	-0.48299	-0.48358
S79	0.15949	0.14672	0.13681	0.17478	0.20033	0.1383	0.12128	0.30425	0.09598	0.17348	0.3814	1.86906	1.64022	0.08036	0.05312
S80	0.37516	0.39131	0.18618	0.12532	0.20952	0.10248	0.06133	0.11278	0.2116	0.04016	0.23789	0.12557	1.49954	-0.12101	-0.21108
S81	0.18672	0.20628	0.2225	0.12917	0.12096	0.1989	0.23153	0.24877	0.21981	0.28478	0.23608	0.48206	1.54804	0.31914	0.20665
S82	-0.66923	-0.681	-0.67616	-0.65686	-0.63959	-0.61233	-0.60602	-0.62333	-0.5677	-0.5514	-0.57309	-1.00397	-1.03982	-0.48299	-0.48358
S83	-0.66923	-0.681	-0.67616	-0.65686	-0.63959	-0.61233	-0.60602	-0.62333	-0.5677	-0.5514	-0.57309	-1.00397	2.03557	-0.48299	-0.48358
S84	-0.66923	-0.681	-0.67616	-0.65686	-0.63959	-0.61233	-0.60602	-0.62333	-0.5677	-0.5514	-0.57309	-1.00397	-1.03982	-0.48299	-0.48358
S85	0.29735	0.19084	0.33855	0.22757	0.03976	-0.27178	-0.479	-0.62333	-0.5677	-0.5514	-0.57309	-0.64389	-0.49135	-0.48299	-0.48358
S86	-0.02815	-0.11844	-0.08779	-0.11045	-0.14247	-0.14301	-0.31276	-0.03517	-0.15868	-0.2272	-0.19165	-0.2906	0.60411	-0.31351	-0.22608
S87	-0.66923	-0.681	-0.67616	-0.65686	-0.63959	-0.61233	-0.60602	-0.62333	-0.5677	-0.5514	-0.57309	-0.07607	0.80723	-0.48299	-0.48358
S88	0.17108	0.09445	0.13279	-0.02187	-0.03254	0.00745	-0.09575	0.18768	-0.06321	-0.10069	0.00545	1.96961	-0.04194	-0.19121	-0.1246
S89	0.30687	0.20201	0.16077	0.15884	0.11612	0.17037	0.03055	0.35496	0.11937	0.19864	0.08079	0.01198	0.70621	0.10541	0.10154
S90	-0.05583	-0.11044	-0.23778	-0.22734	-0.25789	-0.26781	-0.22958	-0.37451	-0.24914	-0.34539	-0.46164	-0.83317	0.4128	-0.42959	-0.42428
S91	1.01662	0.88537	1.11914	1.17802	0.95664	0.63099	0.60974	0.63638	1.21034	0.53006	0.50464	0.51165	1.06668	0.25417	0.20777
S92	-0.32175	-0.2883	-0.31211	-0.4433	-0.2671	-0.30615	-0.39883	-0.37354	-0.28767	-0.26965	-0.33398	0.68628	0.64458	-0.29363	-0.38064

S93	-0.66923	-0.681	-0.67616	-0.65686	-0.63959	-0.61233	-0.60602	-0.62333	-0.5677	-0.5514	-0.57309	0.88467	1.08385	-0.48299	-0.48358
S94	-0.66923	-0.681	-0.67616	-0.65686	-0.63959	-0.61233	-0.60602	-0.62333	-0.5677	-0.5514	-0.57309	-1.00397	-1.03982	-0.48299	-0.48358
S95	-0.66923	-0.681	-0.67616	-0.65686	-0.63959	-0.61233	-0.60602	-0.62333	-0.5677	-0.5514	-0.57309	2.48722	-0.03122	-0.48299	-0.48358
S96	1.05126	1.00025	1.00736	0.95158	0.87405	1.0674	0.90516	1.06447	0.99069	1.03225	1.07577	2.01435	2.17653	0.91711	0.83298
S97	0.06369	0.07041	0.07406	0.18602	0.17579	0.10911	0.08214	0.17586	0.09389	0.2047	0.18638	1.08805	0.1928	0.16137	0.16448
S98	-0.66923	-0.681	-0.67616	-0.65686	-0.63959	-0.61233	-0.60602	-0.62333	-0.5677	-0.5514	-0.57309	-0.87687	-1.03982	-0.48299	-0.48358
S99	-0.66923	-0.681	-0.67616	-0.65686	-0.63959	-0.61233	-0.60602	-0.62333	-0.5677	-0.5514	-0.57309	0.68208	-0.76704	-0.48299	-0.48358
S100	-0.09964	-0.1144	-0.18093	-0.24982	-0.17966	-0.27464	-0.30462	0.02797	-0.30826	-0.26323	-0.26743	0.04799	0.80207	-0.46943	-0.48358
S101	-0.66923	-0.681	-0.67616	-0.65686	-0.63959	-0.61233	-0.60602	-0.62333	-0.5677	-0.5514	-0.57309	0.65984	0.9655	-0.48299	-0.48358
S102	-0.66923	-0.681	-0.67616	-0.65686	-0.63959	-0.61233	-0.60602	-0.62333	-0.5677	-0.5514	-0.57309	0.76384	0.12438	-0.48299	-0.48358
S103	0.23982	0.10742	0.11062	-0.05227	-0.04873	-0.25823	-0.31637	-0.06352	-0.40666	-0.5514	-0.41261	-0.03424	1.52162	-0.48299	-0.48358
S104	0.15937	0.16199	0.14014	0.13378	0.03603	0.04764	0.09761	0.24848	-0.04914	0.02612	0.01441	0.18966	0.89977	-0.18862	-0.0828
S105	-0.40934	-0.47957	-0.47849	-0.39203	-0.36409	-0.61233	-0.60602	-0.54056	-0.41321	-0.5514	-0.52469	-0.31807	0.15695	-0.48299	-0.48358
S106	-0.54598	-0.47667	-0.4295	-0.42026	-0.40957	-0.57782	-0.60602	-0.62333	-0.5677	-0.5514	-0.52543	0.23422	1.1736	-0.48299	-0.48358
S107	-0.66923	-0.681	-0.67616	-0.65686	-0.63959	-0.61233	-0.60602	-0.62333	-0.5677	-0.5514	-0.57309	-1.00397	-1.02481	-0.48299	-0.48358
S108	-0.66923	-0.681	-0.56473	-0.65686	-0.63959	-0.61233	-0.60602	-0.62333	-0.5677	-0.5514	-0.57309	-0.33052	-0.3499	-0.48299	-0.48358
S109	-0.66923	-0.62058	-0.67616	-0.65686	-0.63959	-0.61233	-0.60602	-0.62333	-0.5677	-0.5514	-0.57309	0.00675	-0.00953	-0.48299	-0.48358

Sample No.	integrated bins														
	6.40 .. 6.38	6.38 .. 6.36	6.36 .. 6.34	6.34 .. 6.32	6.32 .. 6.30	6.30 .. 6.28	6.28 .. 6.26	6.26 .. 6.24	6.24 .. 6.22	6.22 .. 6.20	6.20 .. 6.18	6.18 .. 6.16	6.16 .. 6.14	6.14 .. 6.12	6.12 .. 6.10
S1	-0.4803	-0.48038	-0.47773	-0.47501	-0.47415	-0.4687	-0.46639	-0.46863	-0.47755	-0.47145	-0.4753	-0.4829	-0.48882	-0.50067	-0.50809
S2	1.53578	1.53464	1.58947	1.54897	1.55311	1.52851	1.50589	1.47426	1.49819	1.46572	1.43594	1.37729	1.45966	1.4276	1.42997
S3	-0.4803	-0.48038	-0.47773	-0.47501	-0.47415	-0.4687	-0.46639	-0.46863	-0.47755	-0.47145	-0.4753	-0.4829	-0.48882	-0.50067	-0.50809
S4	0.01977	0.07338	0.01643	0.10676	0.20938	0.25606	0.3224	0.42157	0.37201	0.3788	0.47325	0.47079	0.49531	0.58637	0.60412
S5	-0.4803	-0.48038	-0.47773	-0.47501	-0.47415	-0.4687	-0.46639	-0.46863	-0.47755	-0.47145	-0.4753	-0.4829	-0.48882	-0.50067	-0.50809
S6	-0.04913	-0.00683	-0.12441	-0.08489	-0.11034	-0.06364	-0.02068	-0.0863	-0.0907	-0.06234	-0.04458	-0.04984	0.00126	0.0227	0.02265
S7	0.3633	0.42456	0.36243	0.41487	0.44687	0.44821	0.39244	0.45179	0.48184	0.50155	0.487	0.55197	0.51839	0.53474	0.54537
S8	-0.4803	-0.48038	-0.47773	-0.47501	-0.47415	-0.4687	-0.46639	-0.46863	-0.47755	-0.47145	-0.4753	-0.4829	-0.48882	-0.50067	-0.50809
S9	-0.07356	-0.16363	-0.1168	-0.10932	-0.12602	-0.13335	-0.09224	-0.12489	-0.08411	-0.05677	-0.05023	0.00291	0.02583	0.04097	-0.0148
S10	0.82769	0.73692	0.80071	0.76077	0.65307	0.64411	0.73542	0.69536	0.68658	0.64911	0.65033	0.64253	0.61562	0.59044	0.62303
S11	8.36509	8.41959	8.46087	8.51317	8.55722	8.59848	8.63953	8.71277	8.74338	8.75275	8.78933	8.79151	8.79491	8.80984	8.8199
S12	-0.4803	-0.48038	-0.47773	-0.47501	-0.47415	-0.4687	-0.46639	-0.46863	-0.47755	-0.47145	-0.4753	-0.4829	-0.48882	-0.50067	-0.46318
S13	-0.4803	-0.48038	-0.47773	-0.47501	-0.47415	-0.4687	-0.46639	-0.46863	-0.47755	-0.47145	-0.4753	-0.4829	-0.48882	-0.50067	-0.50809
S14	0.22415	0.20943	0.21026	0.19527	0.17873	0.20781	0.21763	0.20022	0.14095	0.17696	0.16819	0.22226	0.19974	0.22587	0.2545
S15	-0.4803	-0.48038	-0.47773	-0.47501	-0.47415	-0.4687	-0.46639	-0.46863	-0.47755	-0.47145	-0.4753	-0.4829	-0.48882	-0.50067	-0.50809
S16	0.05031	0.08563	0.07193	0.05611	0.05528	0.06883	0.08868	0.0579	0.06514	0.07877	0.06125	0.12997	0.13019	0.11351	0.10962
S17	-0.4803	-0.48038	-0.47773	-0.47501	-0.47415	-0.4687	-0.46639	-0.46863	-0.47755	-0.47145	-0.4753	-0.4829	-0.48882	-0.50067	-0.50809
S18	0.23041	0.30385	0.3797	0.36293	0.36992	0.35599	0.30572	0.34536	0.35618	0.36925	0.33641	0.35886	0.4064	0.3111	0.33482
S19	2.49826	2.46818	2.44713	2.39624	2.44595	2.42911	2.37574	2.34415	2.40371	2.41085	2.29845	2.27572	2.29781	2.27999	2.27432
S20	-0.4803	-0.48038	-0.47773	-0.47501	-0.47415	-0.4687	-0.46639	-0.46863	-0.47755	-0.47145	-0.4753	-0.4829	-0.48882	-0.50067	-0.50809
S21	-0.4803	-0.48038	-0.47773	-0.47501	-0.47415	-0.4687	-0.46639	-0.46863	-0.47755	-0.47145	-0.4753	-0.4829	-0.48882	-0.50067	-0.50809
S22	1.04574	1.06168	1.0374	0.98907	0.98316	1.04764	0.99936	1.04375	0.89678	1.02712	1.04792	1.0225	0.97146	0.96476	1.00188
S23	0.47551	0.54876	0.48572	0.47899	0.46043	0.45522	0.45463	0.43839	0.44646	0.45023	0.46557	0.44409	0.47833	0.46062	0.42092
S24	-0.47521	-0.41785	-0.43839	-0.39595	-0.39131	-0.37696	-0.4604	-0.4073	-0.24464	-0.29419	-0.30645	-0.33553	-0.3327	-0.36028	-0.33499

S25	0.33892	0.27149	0.32161	0.20969	0.31106	0.23608	0.19189	0.16702	0.17364	0.1377	0.09438	0.15589	0.14633	0.09763	0.08275
S26	-0.02675	0.02048	-0.0022	-0.04378	-0.09737	-0.15866	-0.13197	-0.14361	-0.1467	-0.17084	-0.19375	-0.2107	-0.18894	-0.21635	-0.26925
S27	-0.4803	-0.48038	-0.47773	-0.47501	-0.47415	-0.4687	-0.46639	-0.46863	-0.47755	-0.47145	-0.4753	-0.4829	-0.48882	-0.50067	-0.50809
S28	-0.4803	-0.48038	-0.47773	-0.47501	-0.47415	-0.4687	-0.46639	-0.46863	-0.47755	-0.47145	-0.4753	-0.4829	-0.48882	-0.50067	-0.50809
S29	-0.4803	-0.48038	-0.47773	-0.47501	-0.47415	-0.4687	-0.46639	-0.46863	-0.47755	-0.47145	-0.4753	-0.4829	-0.48882	-0.50067	-0.50809
S30	0.03448	0.02814	-0.00307	0.01786	0.09881	0.04022	0.03765	0.08391	0.08532	0.02009	0.07123	0.09464	0.063	0.05881	0.09229
S31	-0.09416	-0.09547	-0.09191	-0.12466	-0.15417	-0.12564	-0.17392	-0.10105	-0.07108	-0.09495	-0.09399	-0.1112	-0.12175	-0.11122	-0.14725
S32	-0.4803	-0.48038	-0.47773	-0.47501	-0.47415	-0.4687	-0.46639	-0.46863	-0.47755	-0.47145	-0.4753	-0.4829	-0.48882	-0.50067	-0.50809
S33	-0.4803	-0.48038	-0.47773	-0.47501	-0.47415	-0.4687	-0.46639	-0.46863	-0.47755	-0.47145	-0.4753	-0.4829	-0.47476	-0.43111	-0.50512
S34	-0.04495	-0.06463	-0.07906	-0.09703	-0.03969	-0.06402	0.01825	-0.02292	-0.03664	-0.04462	0.0285	-0.02803	0.02017	-0.00816	-0.0451
S35	0.42105	0.3787	0.43093	0.35756	0.4743	0.42233	0.37998	0.33914	0.36637	0.37423	0.37561	0.44714	0.44835	0.41696	0.37085
S36	-0.33199	-0.30348	-0.30039	-0.21988	-0.24523	-0.24253	-0.19188	-0.18332	-0.18779	-0.20365	-0.18527	-0.08688	-0.09935	-0.14435	-0.08713
S37	0.09832	-0.04527	-0.00977	0.03884	0.005	0.056	0.03241	-0.01442	-0.02009	-0.05912	-0.00794	0.00383	-0.01302	-0.02662	-0.01622
S38	0.23926	0.20008	0.12915	0.20577	0.16011	0.16637	0.1117	0.13268	0.1177	0.0203	0.02884	0.00326	-0.00474	-0.0069	-0.05231
S39	-0.4803	-0.48038	-0.47773	-0.47501	-0.47415	-0.4687	-0.46639	-0.46863	-0.47755	-0.47145	-0.47277	-0.46847	-0.48882	-0.42191	-0.44372
S40	1.32167	1.36295	1.36697	1.37457	1.34767	1.41575	1.43132	1.42298	1.35891	1.4048	1.39729	1.37706	1.368	1.38671	1.40937
S41	0.19264	0.22513	0.22869	0.27458	0.22134	0.25107	0.25544	0.30212	0.28518	0.28954	0.34669	0.21607	0.31008	0.27175	0.25369
S42	-0.06491	-0.04033	-0.0408	-0.02772	-0.03767	0.06029	-0.02587	0.06441	0.00027	0.01751	-0.02061	0.01749	0.03635	-0.04067	-0.02495
S43	-0.4803	-0.48038	-0.47773	-0.47501	-0.47415	-0.4687	-0.46639	-0.46863	-0.47755	-0.47145	-0.4753	-0.4829	-0.48882	-0.46733	-0.50809
S44	-0.4803	-0.48038	-0.47773	-0.47501	-0.47415	-0.4687	-0.46639	-0.46863	-0.47281	-0.47145	-0.45972	-0.4829	-0.48882	-0.45559	-0.38878
S45	-0.16449	-0.14339	-0.08698	-0.12687	-0.14723	-0.12386	-0.12886	-0.12464	-0.06004	-0.12991	-0.14904	-0.1796	-0.15366	-0.123	-0.06132
S46	0.13349	0.1036	0.12597	0.10563	0.15778	0.12488	0.10185	0.12406	0.13015	0.14984	0.14731	0.15811	0.2074	0.18584	0.1567
S47	0.01201	0.03147	0.02807	0.02309	0.03379	-0.00073	0.02034	0.043	0.1117	0.05232	0.09093	0.10682	0.01267	0.06275	0.05222
S48	-0.4803	-0.48038	-0.47773	-0.47501	-0.47415	-0.4687	-0.46639	-0.46863	-0.47755	-0.47145	-0.4753	-0.4829	-0.48882	-0.50067	-0.50809
S49	-0.4803	-0.48038	-0.47773	-0.47501	-0.47415	-0.4687	-0.46639	-0.46863	-0.47755	-0.47145	-0.4753	-0.4829	-0.48882	-0.50067	-0.50809
S50	1.04633	1.0597	0.99891	1.06684	1.02892	1.08616	1.05141	1.08768	1.089	1.07846	1.09692	1.15353	1.12572	1.11034	1.09374
S51	-0.15296	-0.05476	-0.04534	-0.06706	-0.14247	-0.05931	-0.04337	-0.0394	0.00818	-0.01185	-0.02133	-0.08416	-0.02024	-0.00637	-0.00453
S52	0.15431	0.24008	0.24073	0.25944	0.21565	0.13518	0.17343	0.2554	0.25124	0.28469	0.23279	0.34337	0.37837	0.35116	0.34919
S53	-0.23749	-0.26629	-0.29435	-0.26276	-0.24439	-0.31294	-0.265	-0.20829	-0.2417	-0.25786	-0.26877	-0.25505	-0.25057	-0.25523	-0.23691
S54	-0.29566	-0.27472	-0.21973	-0.24223	-0.29638	-0.24168	-0.29374	-0.20434	-0.28681	-0.18268	-0.24561	-0.21027	-0.21116	-0.19324	-0.15894
S55	0.32494	0.27621	0.34523	0.32197	0.34261	0.26484	0.28177	0.34583	0.30374	0.30475	0.33828	0.34773	0.37953	0.3314	0.36414
S56	1.06108	1.10979	0.97183	1.02558	1.0293	0.99723	0.99946	1.02544	0.93199	0.93657	0.91754	0.90303	0.90632	0.84263	0.87628
S57	-0.45526	-0.4443	-0.38789	-0.43935	-0.39198	-0.42993	-0.40285	-0.42825	-0.33782	-0.32741	-0.35535	-0.37932	-0.29713	-0.32304	-0.31846
S58	-0.4803	-0.48038	-0.47773	-0.47501	-0.47415	-0.4687	-0.46639	-0.46863	-0.47755	-0.47145	-0.4753	-0.4829	-0.48882	-0.50067	-0.50809
S59	0.08681	0.20651	0.12623	0.15063	0.1504	0.22686	0.16475	0.15896	0.18843	0.19906	0.14246	0.23969	0.22758	0.2559	0.28587
S60	2.44963	2.41986	2.36085	2.29014	2.21127	2.18374	2.12126	2.02879	1.9944	1.96188	1.92979	1.90358	1.86771	1.82247	1.73669
S61	-0.4803	-0.48038	-0.47773	-0.47501	-0.47415	-0.4687	-0.46639	-0.46863	-0.47755	-0.47145	-0.4753	-0.4829	-0.48882	-0.50067	-0.50809
S62	-0.4803	-0.48038	-0.47773	-0.47501	-0.47415	-0.4687	-0.46639	-0.46863	-0.47755	-0.47145	-0.4753	-0.4829	-0.4634	-0.48545	-0.47573
S63	0.54417	0.44881	0.37135	0.34998	0.36147	0.34643	0.27716	0.22483	0.27747	0.20647	0.21694	0.13977	0.09918	0.18023	0.05983
S64	0.4889	0.46262	0.51222	0.4146	0.60288	0.59415	0.51664	0.55157	0.52496	0.53219	0.56559	0.61598	0.50513	0.60439	0.59051
S65	-0.4803	-0.48038	-0.47773	-0.47501	-0.47415	-0.4687	-0.46639	-0.46863	-0.47755	-0.47145	-0.4753	-0.4829	-0.48882	-0.50067	-0.50809
S66	0.91984	1.00718	0.90547	0.9046	0.86914	0.75787	0.88766	0.82721	0.76673	0.80825	0.83176	0.82042	0.79692	0.79685	0.79715
S67	-0.4803	-0.48038	-0.47773	-0.47501	-0.47415	-0.4687	-0.46639	-0.46863	-0.47755	-0.47145	-0.4753	-0.4829	-0.48882	-0.50067	-0.50809
S68	-0.06703	-0.00175	-0.03748	-0.02589	0.01354	0.02717	0.01597	-0.05326	0.04831	0.03223	0.01442	0.03312	-0.05248	0.00476	0.0291

S69	-0.4803	-0.48038	-0.47773	-0.47501	-0.47415	-0.4687	-0.46639	-0.46863	-0.47755	-0.47145	-0.4753	-0.4829	-0.48882	-0.50067	-0.50809
S70	-0.4803	-0.48038	-0.47773	-0.47501	-0.47415	-0.4687	-0.46639	-0.46863	-0.47755	-0.47145	-0.43295	-0.42933	-0.36164	-0.36924	-0.28406
S71	-0.4803	-0.48038	-0.47773	-0.47501	-0.47415	-0.4687	-0.46639	-0.46863	-0.47755	-0.47145	-0.4753	-0.4829	-0.48882	-0.50067	-0.50809
S72	-0.4803	-0.48038	-0.47773	-0.47501	-0.47415	-0.4687	-0.46639	-0.46863	-0.47755	-0.47145	-0.4753	-0.4829	-0.48882	-0.50067	-0.50809
S73	-0.4803	-0.48038	-0.47773	-0.47501	-0.47415	-0.4687	-0.46639	-0.46863	-0.47755	-0.47145	-0.4753	-0.4829	-0.48882	-0.50067	-0.50809
S74	0.72846	0.65483	0.63072	0.61064	0.56248	0.50724	0.4623	0.49194	0.46852	0.39852	0.47908	0.42718	0.37212	0.42493	0.41384
S75	-0.4803	-0.48038	-0.43313	-0.43703	-0.47415	-0.44946	-0.43804	-0.44533	-0.33692	-0.4221	-0.38309	-0.31375	-0.32662	-0.22575	-0.18507
S76	-0.2713	-0.35689	-0.33913	-0.22876	-0.26784	-0.3054	-0.22381	-0.26314	-0.19663	-0.15791	-0.19152	-0.18643	-0.12494	-0.00883	0.02841
S77	-0.4803	-0.48038	-0.47773	-0.47501	-0.47415	-0.4687	-0.46639	-0.46863	-0.47755	-0.47145	-0.4753	-0.4829	-0.48882	-0.50067	-0.50809
S78	-0.4803	-0.48038	-0.47773	-0.47501	-0.47415	-0.4687	-0.46639	-0.46863	-0.47755	-0.47145	-0.4753	-0.4829	-0.48882	-0.50067	-0.50809
S79	0.06101	0.01433	0.11542	0.07093	0.1209	0.10306	0.1483	0.07843	0.15402	0.16915	0.20065	0.18013	0.18031	0.23049	0.19473
S80	-0.19193	-0.26202	-0.17424	-0.2408	-0.19064	-0.23758	-0.30743	-0.25324	-0.24432	-0.27467	-0.19262	-0.24924	-0.16115	-0.17231	-0.22898
S81	0.29385	0.23987	0.28751	0.24199	0.26305	0.21736	0.20975	0.21648	0.2408	0.22795	0.2249	0.28031	0.26936	0.26005	0.25807
S82	-0.4803	-0.48038	-0.47773	-0.47501	-0.47415	-0.4687	-0.46639	-0.46863	-0.47755	-0.47145	-0.4753	-0.4829	-0.48882	-0.50067	-0.50809
S83	-0.4803	-0.48038	-0.47773	-0.47501	-0.47415	-0.4687	-0.46639	-0.46863	-0.47755	-0.47145	-0.4753	-0.4829	-0.48882	-0.50067	-0.50809
S84	-0.4803	-0.48038	-0.47773	-0.47501	-0.47415	-0.4687	-0.46639	-0.46863	-0.47755	-0.47145	-0.4753	-0.4829	-0.48882	-0.50067	-0.50809
S85	-0.4803	-0.48038	-0.47773	-0.47501	-0.47415	-0.4687	-0.46639	-0.46863	-0.47755	-0.47145	-0.4753	-0.4829	-0.48882	-0.50067	-0.50809
S86	-0.29153	-0.2405	-0.30563	-0.23259	-0.26658	-0.31249	-0.31005	-0.30759	-0.30302	-0.25318	-0.24072	-0.20649	-0.20187	-0.19662	-0.19832
S87	-0.4803	-0.48038	-0.47773	-0.47501	-0.47415	-0.4687	-0.46639	-0.46863	-0.47755	-0.47145	-0.4753	-0.4829	-0.48882	-0.50067	-0.50809
S88	-0.11747	-0.10791	-0.14256	-0.12376	-0.10951	-0.04041	-0.08558	-0.06222	-0.05386	-0.00927	-0.01037	0.00407	-0.02253	0.04245	0.09414
S89	0.13421	0.03277	0.06584	0.00458	0.03474	0.00988	0.05091	0.06714	0.07493	0.01415	0.08615	-0.02945	0.05056	0.0791	0.02688
S90	-0.47202	-0.45759	-0.42424	-0.47501	-0.44941	-0.4687	-0.46639	-0.46863	-0.46146	-0.44088	-0.4753	-0.41936	-0.42133	-0.46216	-0.36229
S91	0.30355	0.24617	0.23888	0.34264	0.1726	0.22163	0.2714	0.16745	0.24054	0.15856	0.10012	0.17669	0.20118	0.1557	0.16573
S92	-0.3765	-0.38749	-0.42176	-0.4223	-0.45407	-0.43789	-0.46639	-0.39035	-0.40117	-0.44018	-0.43109	-0.34822	-0.4337	-0.3785	-0.37421
S93	-0.4803	-0.48038	-0.47773	-0.47501	-0.47415	-0.4687	-0.46639	-0.46863	-0.47755	-0.47145	-0.4753	-0.4829	-0.48882	-0.50067	-0.50809
S94	-0.4803	-0.48038	-0.47773	-0.47501	-0.47415	-0.4687	-0.46639	-0.46863	-0.47755	-0.47145	-0.4753	-0.4829	-0.48882	-0.50067	-0.50809
S95	-0.4803	-0.48038	-0.47773	-0.47501	-0.47415	-0.4687	-0.46639	-0.46863	-0.47755	-0.47145	-0.4753	-0.4829	-0.48882	-0.50067	-0.50809
S96	0.86174	0.87442	0.88041	0.86113	0.82885	0.74681	0.80277	0.80161	0.80735	0.76901	0.80738	0.78532	0.78964	0.82055	0.80838
S97	0.19166	0.17449	0.1438	0.18528	0.23339	0.24827	0.2692	0.22951	0.24475	0.28488	0.25133	0.27577	0.29624	0.37098	0.43385
S98	-0.4803	-0.48038	-0.47773	-0.47501	-0.47415	-0.4687	-0.46639	-0.46863	-0.47755	-0.47145	-0.4753	-0.4829	-0.48882	-0.50067	-0.50809
S99	-0.4803	-0.48038	-0.47773	-0.47501	-0.47415	-0.4687	-0.46639	-0.46863	-0.47755	-0.47145	-0.4753	-0.4829	-0.4655	-0.48556	-0.39699
S100	-0.4803	-0.48038	-0.47773	-0.47501	-0.47415	-0.4687	-0.46639	-0.46863	-0.47755	-0.47145	-0.4753	-0.4829	-0.48882	-0.49639	-0.4508
S101	-0.4803	-0.48038	-0.47773	-0.47501	-0.47415	-0.4687	-0.46639	-0.46863	-0.47755	-0.47145	-0.4753	-0.4829	-0.48882	-0.50067	-0.50809
S102	-0.4803	-0.48038	-0.47773	-0.47501	-0.47415	-0.4687	-0.46639	-0.46863	-0.47755	-0.47145	-0.4753	-0.4829	-0.48882	-0.50067	-0.50809
S103	-0.4803	-0.48038	-0.47773	-0.47501	-0.47415	-0.4687	-0.46639	-0.46863	-0.47755	-0.47145	-0.4753	-0.4829	-0.48882	-0.50067	-0.50809
S104	-0.18287	-0.08065	-0.14131	-0.1569	-0.13312	-0.1851	-0.18119	-0.18781	-0.17762	-0.15994	-0.141	-0.15228	-0.14824	-0.06505	-0.13727
S105	-0.4803	-0.42483	-0.47773	-0.47501	-0.47415	-0.4687	-0.46639	-0.4396	-0.45502	-0.44982	-0.33231	-0.45132	-0.38967	-0.33747	-0.26691
S106	-0.4803	-0.48038	-0.47773	-0.47501	-0.47415	-0.4687	-0.46639	-0.46863	-0.47755	-0.47145	-0.4753	-0.4829	-0.48882	-0.50067	-0.50809
S107	-0.4803	-0.48038	-0.47773	-0.47501	-0.47415	-0.4687	-0.46639	-0.46863	-0.47755	-0.47145	-0.4753	-0.4829	-0.48882	-0.50067	-0.50809
S108	-0.4803	-0.48038	-0.47773	-0.47501	-0.47415	-0.4687	-0.46639	-0.46863	-0.47755	-0.47145	-0.4753	-0.4829	-0.48882	-0.50067	-0.50809
S109	-0.4803	-0.48038	-0.47773	-0.47501	-0.47415	-0.4687	-0.46639	-0.46863	-0.47755	-0.47145	-0.4753	-0.4829	-0.48882	-0.50067	-0.50809
Sample No.	integrated bins														
	6.10 .. 6.08	6.08 .. 6.06	6.06 .. 6.04	6.04 .. 6.02	6.02 .. 6.00	6.00 .. 5.98	5.98 .. 5.96	5.96 .. 5.94	5.94 .. 5.92	5.92 .. 5.90	5.90 .. 5.88	5.88 .. 5.86	5.86 .. 5.84	5.84 .. 5.82	5.82 .. 5.80

S1	-0.5148	-0.54096	-0.56937	-0.56286	-0.64189	-0.62667	-0.57598	-0.58588	-0.58011	-0.5593	-0.55356	-0.60712	-0.83884	-0.63362	-0.54355
S2	1.42815	1.39763	1.38084	1.34195	1.3591	1.43407	1.38087	1.31722	1.40969	1.45714	1.34045	1.40154	1.69249	1.40283	1.37476
S3	-0.5148	-0.54096	-0.56937	-0.56286	-0.64189	-0.62667	-0.57598	-0.58588	-0.58011	-0.5593	-0.55356	-0.60712	-0.2687	-0.63362	-0.54355
S4	0.65485	0.68064	0.69854	0.80145	0.92459	0.96068	0.89864	0.95954	1.03316	1.11703	1.25754	1.4276	1.59299	1.36015	1.34955
S5	-0.5148	-0.54096	-0.56937	-0.56286	-0.64189	-0.62667	-0.57598	-0.58588	-0.58011	-0.5593	-0.55356	-0.53526	-0.48816	-0.55794	-0.54355
S6	0.03052	0.00743	0.031	0.05743	0.23315	0.12414	0.04792	0.03401	0.07063	0.03453	0.0346	0.17957	0.38358	0.21926	0.06352
S7	0.52351	0.59236	0.55604	0.61867	0.65778	0.66635	0.6334	0.57561	0.6447	0.53667	0.56791	0.49743	0.44462	0.48201	0.51044
S8	-0.5148	-0.54096	-0.56937	-0.56286	-0.64189	-0.62667	-0.57598	-0.58588	-0.58011	-0.5593	-0.55356	-0.60712	-0.06344	-0.63362	-0.54355
S9	-0.00843	0.03078	-0.01641	-0.00093	-0.07652	-0.07894	0.00624	0.04234	0.0729	0.09858	0.12077	0.12598	0.68066	0.31571	0.26373
S10	0.61156	0.58863	0.56775	0.55834	0.598	0.59858	0.58653	0.58293	0.54803	0.57013	0.53227	0.5663	0.60509	0.49549	0.49792
S11	8.81985	8.78219	8.73632	8.77907	8.54517	8.47717	8.70842	8.68575	8.64692	8.66959	8.7032	8.54379	7.73216	8.25199	8.67704
S12	-0.42154	-0.41157	-0.38282	-0.28428	-0.27805	-0.32617	-0.35162	-0.36874	-0.42561	-0.44757	-0.49996	-0.51931	-0.54093	-0.56142	-0.54355
S13	-0.5148	-0.54096	-0.56937	-0.56286	-0.64189	-0.62667	-0.57598	-0.58588	-0.58011	-0.5593	-0.55356	-0.60712	-0.83884	-0.63362	-0.54355
S14	0.22373	0.20751	0.11791	0.2116	0.41571	0.34464	0.18534	0.2577	0.19706	0.16049	0.17527	0.31788	0.50137	0.25523	0.14071
S15	-0.5148	-0.54096	-0.56937	-0.56286	-0.64189	-0.62667	-0.57598	-0.58588	-0.58011	-0.5593	-0.55356	-0.60712	-0.83884	-0.63362	-0.54355
S16	0.17792	0.19481	0.16567	0.1799	0.22482	0.27746	0.20594	0.18474	0.14965	0.16987	0.17177	0.31891	0.19657	0.16572	0.08881
S17	-0.5148	-0.54096	-0.56937	-0.56286	-0.64189	-0.62667	-0.57598	-0.58588	-0.58011	-0.5422	-0.46654	-0.485	-0.05058	-0.27483	-0.4443
S18	0.33087	0.2587	0.35091	0.31648	0.33249	0.33355	0.37066	0.3735	0.4458	0.43144	0.42252	0.3827	0.60399	0.55931	0.4088
S19	2.23706	2.17875	2.19363	2.17562	2.04054	2.40643	2.34856	2.23803	2.23776	2.35842	2.30718	2.172	2.16634	3.03399	2.35505
S20	-0.5148	-0.54096	-0.56937	-0.56286	-0.64189	-0.62667	-0.57598	-0.58588	-0.58011	-0.5593	-0.55356	-0.60712	-0.22155	-0.48133	-0.54355
S21	-0.5148	-0.54096	-0.56937	-0.56286	-0.64189	-0.62667	-0.57598	-0.58588	-0.58011	-0.5593	-0.55356	-0.60712	-0.83884	-0.63362	-0.54355
S22	1.00145	0.93883	0.9684	0.98292	0.92593	0.99705	0.90996	0.97194	0.968	0.92218	0.88393	0.91885	0.73663	0.78796	0.92304
S23	0.43945	0.42675	0.40447	0.41635	0.41581	0.44814	0.41253	0.42082	0.44345	0.44658	0.42845	0.37866	0.39542	0.45634	0.45616
S24	-0.32052	-0.34094	-0.33558	-0.27357	-0.24392	-0.25312	-0.28969	-0.26142	-0.24668	-0.27653	-0.23491	-0.28117	-0.20496	-0.10324	-0.18883
S25	0.06733	-0.00116	-0.0307	-0.07078	0.03382	0.03728	-0.04485	-0.06049	-0.07479	-0.02913	-0.09588	-0.11686	0.12438	0.02404	-0.06619
S26	-0.2914	-0.33453	-0.31279	-0.34086	-0.35218	-0.33624	-0.35359	-0.3768	-0.35849	-0.37768	-0.38756	-0.39467	-0.01392	-0.24375	-0.36244
S27	-0.5148	-0.54096	-0.56937	-0.56286	-0.57331	-0.58452	-0.57598	-0.58588	-0.58011	-0.5593	-0.55356	-0.53507	-0.24617	-0.48617	-0.54355
S28	-0.5148	-0.54096	-0.56937	-0.56286	-0.64189	-0.62667	-0.57598	-0.58588	-0.58011	-0.5593	-0.55356	-0.60712	-0.75634	-0.63362	-0.54355
S29	-0.5148	-0.54096	-0.56937	-0.56286	-0.64189	-0.62667	-0.57598	-0.58588	-0.58011	-0.5593	-0.55356	-0.60712	-0.65436	-0.63362	-0.54355
S30	0.11472	0.06698	0.04111	0.03799	0.33135	0.31199	0.08979	0.09414	0.16166	0.18994	0.14983	0.24312	1.03152	0.43004	0.14484
S31	-0.14733	-0.02876	0.00877	-0.12983	-0.0819	-0.09331	-0.16865	-0.17352	-0.13799	-0.17681	-0.11205	-0.05959	0.14545	-0.02814	-0.15568
S32	-0.5148	-0.54096	-0.56937	-0.56286	-0.64189	-0.62667	-0.57598	-0.58588	-0.58011	-0.5593	-0.55356	-0.60712	-0.2829	-0.63362	-0.54355
S33	-0.49291	-0.48181	-0.46697	-0.47981	-0.41998	-0.43368	-0.44385	-0.42665	-0.46148	-0.46339	-0.44847	-0.40238	-0.44774	-0.41212	-0.44933
S34	-0.01969	-0.02506	-0.02406	0.0216	0.02506	0.0145	0.01069	-0.00045	0.03232	0.04488	0.03027	0.05674	0.09162	0.08971	-0.01624
S35	0.32221	0.40116	0.35221	0.3592	0.47099	0.51429	0.33529	0.39492	0.40477	0.3934	0.40358	0.46387	0.86898	0.53452	0.45255
S36	-0.12156	-0.1244	-0.14907	-0.08873	0.07238	0.09589	-0.06606	-0.06799	-0.02092	-0.00263	0.01096	0.17249	0.51344	0.2096	0.06411
S37	0.00825	-0.01547	-0.02605	-0.05556	-0.05846	-0.06607	0.00135	-0.03167	-0.03217	-0.03574	-0.09458	-0.05886	-0.17732	-0.11921	-0.13239
S38	-0.06516	-0.05695	-0.09985	-0.11179	-0.01734	-0.02668	-0.107	-0.14403	-0.10823	-0.12113	-0.12508	0.02648	0.02766	-0.08805	-0.19093
S39	-0.36645	-0.37522	-0.34443	-0.34184	-0.35806	-0.35608	-0.36547	-0.35195	-0.36856	-0.38231	-0.45891	-0.34336	-0.55303	-0.51579	-0.47567
S40	1.39832	1.41283	1.40676	1.39077	1.41106	1.43681	1.36662	1.47506	1.51577	1.54052	1.50329	1.54111	1.65122	1.52736	1.5786
S41	0.22754	0.26169	0.22686	0.23841	0.42149	0.35324	0.24264	0.29997	0.30316	0.24517	0.28513	0.41968	0.67964	0.37133	0.2402
S42	-0.06094	-0.05322	0.00025	-0.02535	0.09499	0.0914	-0.09682	-0.02414	-0.02815	-0.03574	-0.01993	0.11149	0.47209	0.12297	0.03041
S43	-0.49286	-0.43026	-0.45953	-0.43654	-0.50445	-0.43465	-0.40338	-0.40843	-0.44631	-0.43986	-0.45554	-0.43759	-0.64338	-0.50014	-0.47268
S44	-0.3994	-0.28504	-0.34331	-0.34023	-0.31521	-0.28161	-0.3179	-0.32655	-0.36356	-0.39774	-0.41057	-0.44155	-0.61756	-0.50082	-0.45433

S45	-0.09719	-0.10233	-0.06585	-0.02569	-0.09692	-0.07687	-0.07841	-0.12611	-0.17046	-0.19237	-0.19392	-0.23877	-0.45659	-0.31517	-0.30749
S46	0.17766	0.2001	0.15506	0.19376	0.21109	0.181	0.15409	0.19674	0.25837	0.22234	0.2274	0.33965	0.50076	0.28049	0.22849
S47	0.0391	0.03824	0.04493	0.01177	0.10878	0.09466	0.0513	0.03968	0.03323	0.17634	0.11977	0.12172	0.41855	0.21908	0.11038
S48	-0.5148	-0.54096	-0.56937	-0.56286	-0.55299	-0.59129	-0.57598	-0.58588	-0.58011	-0.4649	-0.55153	-0.53704	-0.24404	-0.34681	-0.52162
S49	-0.5148	-0.54096	-0.56937	-0.56286	-0.64189	-0.62667	-0.57598	-0.58588	-0.58011	-0.5593	-0.55356	-0.60712	-0.47108	-0.63362	-0.54355
S50	1.14983	1.14563	1.0792	1.13225	1.16566	1.18565	1.17717	1.22538	1.27331	1.23886	1.30824	1.35091	1.44337	1.28078	1.31063
S51	0.00614	0.06822	0.17352	-0.04699	0.12852	0.10485	-0.03155	-0.03635	0.0788	0.01889	0.02472	0.10524	0.56149	0.23502	0.03838
S52	0.38023	0.39221	0.39097	0.43793	0.62856	0.57374	0.42549	0.49747	0.55635	0.47575	0.55011	0.60012	0.99977	0.82697	0.90485
S53	-0.24497	-0.24107	-0.22443	-0.19987	-0.19781	-0.20828	-0.25545	-0.27222	-0.20084	-0.18719	-0.23976	-0.20449	-0.13206	-0.08104	-0.16071
S54	-0.17487	-0.16742	-0.15424	-0.17094	-0.12459	-0.14668	-0.18367	-0.12666	-0.1099	-0.15762	-0.15718	-0.07234	0.09319	-0.03963	-0.17527
S55	0.35187	0.43595	0.39394	0.42734	0.46433	0.30281	0.40664	0.34771	0.39786	0.40209	0.41972	0.32271	0.09428	0.27391	0.43086
S56	0.87333	0.8873	0.85131	0.79199	0.82003	0.80611	0.84439	0.83587	0.81339	0.79338	0.76674	0.78425	0.78237	0.76656	0.77905
S57	-0.32539	-0.35759	-0.36213	-0.33087	-0.34094	-0.31851	-0.31147	-0.3516	-0.30793	-0.31056	-0.33416	-0.2976	-0.1176	-0.16527	-0.31132
S58	-0.5148	-0.54096	-0.56937	-0.56286	-0.6354	-0.62667	-0.57598	-0.58588	-0.58011	-0.5593	-0.55356	-0.57308	-0.60862	-0.62091	-0.54355
S59	0.24778	0.21083	0.28599	0.2581	0.22394	0.25447	0.27874	0.24685	0.27668	0.26139	0.20828	0.1695	-0.05783	0.16229	0.25591
S60	1.68583	1.62748	1.51078	1.56004	1.57868	1.63996	1.47667	1.43483	1.4224	1.42786	1.39667	1.43506	1.37352	1.46302	1.34908
S61	-0.5148	-0.54096	-0.56937	-0.56286	-0.64189	-0.62667	-0.57598	-0.58588	-0.58011	-0.5593	-0.55356	-0.60712	-0.83884	-0.63362	-0.54355
S62	-0.51407	-0.43324	-0.44715	-0.43583	-0.35732	-0.3482	-0.40554	-0.1241	-0.28154	-0.33049	-0.33543	-0.24309	0.06323	-0.1929	-0.33395
S63	0.00442	0.08435	0.04765	-0.0574	-0.06584	-0.06201	-0.0062	-0.01075	-0.02084	-0.03162	-0.05958	-0.02071	0.29076	0.12133	-0.04414
S64	0.58694	0.80976	1.02255	0.60294	0.67481	0.59436	0.65439	0.63886	0.75707	0.73985	0.7698	0.97669	1.37677	0.915	0.78088
S65	-0.5148	-0.54096	-0.56937	-0.56286	-0.64189	-0.62667	-0.57598	-0.58588	-0.58011	-0.5593	-0.55356	-0.60712	-0.68967	-0.63362	-0.54355
S66	0.76721	0.69245	0.72688	0.74598	0.69394	0.66709	0.73409	0.72057	0.75884	0.76069	0.75448	0.73164	0.6134	0.70216	0.71028
S67	-0.5148	-0.54096	-0.56937	-0.56286	-0.64189	-0.62667	-0.57598	-0.58588	-0.58011	-0.5593	-0.55356	-0.60712	-0.83884	-0.63362	-0.54355
S68	0.02032	-0.05234	0.00856	-0.01149	-0.00807	-0.00382	-0.00446	0.01699	0.03579	0.07716	0.00612	0.0191	0.11144	0.11568	0.08807
S69	-0.5148	-0.53118	-0.40509	-0.56286	-0.56665	-0.55676	-0.57117	-0.56814	-0.58011	-0.5593	-0.55356	-0.49683	-0.51469	-0.54859	-0.50591
S70	-0.22413	-0.09564	0.03185	0.06874	0.12124	0.12847	0.08213	0.01547	-0.12931	-0.22798	-0.23443	-0.3084	-0.47182	-0.39067	-0.37364
S71	-0.5148	-0.50231	-0.41511	-0.51112	-0.4389	-0.48279	-0.48285	-0.49723	-0.50185	-0.52323	-0.50167	-0.48808	-0.53146	-0.49745	-0.4881
S72	-0.5148	-0.54096	-0.56937	-0.56286	-0.64189	-0.62667	-0.57598	-0.58588	-0.58011	-0.5593	-0.55356	-0.52806	-0.56498	-0.5145	-0.54355
S73	-0.5148	-0.54096	-0.56937	-0.56286	-0.64189	-0.62667	-0.57598	-0.58588	-0.58011	-0.5593	-0.55356	-0.60712	-0.83884	-0.63362	-0.54355
S74	0.43449	0.45649	0.48883	0.55074	0.55021	0.4937	0.4684	0.43354	0.37474	0.31465	0.29791	0.2055	0.11534	0.16901	0.17455
S75	-0.21429	-0.15312	-0.05333	0.02089	0.04457	0.02004	0.04239	0.04197	-0.04819	-0.03516	-0.13626	-0.21766	-0.41345	-0.2692	-0.21212
S76	-0.0338	-0.08854	-0.05963	-0.11293	-0.00151	-0.05533	-0.00606	0.16653	0.01512	-0.03474	-0.02955	-0.05946	-0.17831	-0.00192	0.05624
S77	-0.5148	-0.54096	-0.56937	-0.56286	-0.51361	-0.52481	-0.57598	-0.5743	-0.58011	-0.5593	-0.55356	-0.60712	-0.83743	-0.63362	-0.54355
S78	-0.5148	-0.54096	-0.56937	-0.56286	-0.64189	-0.62667	-0.57598	-0.58588	-0.58011	-0.5593	-0.55356	-0.60712	-0.83884	-0.63362	-0.54355
S79	0.27029	0.37672	0.36395	0.32204	0.36561	0.30566	0.29364	0.44219	0.35127	0.2617	0.2464	0.30394	0.22905	0.21486	0.25069
S80	-0.1844	-0.12768	-0.13086	-0.15531	-0.10128	-0.09792	-0.08496	-0.00891	-0.07537	-0.11852	-0.05613	-0.10164	-0.02327	-0.07324	-0.00148
S81	0.32083	0.29866	0.36315	0.33726	0.30073	0.33112	0.35984	0.29516	0.32164	0.30175	0.29196	0.26927	0.08352	0.17659	0.24267
S82	-0.5148	-0.54096	-0.56937	-0.56286	-0.64189	-0.62667	-0.57598	-0.58588	-0.58011	-0.5593	-0.55356	-0.60712	-0.83884	-0.63362	-0.54355
S83	-0.5148	-0.54096	-0.56937	-0.56286	-0.64189	-0.62667	-0.57598	-0.58588	-0.58011	-0.5593	-0.55356	-0.60712	-0.83884	-0.63362	-0.54355
S84	-0.5148	-0.54096	-0.56937	-0.56286	-0.64189	-0.62667	-0.57598	-0.58588	-0.58011	-0.5593	-0.55356	-0.60712	-0.83884	-0.63362	-0.54355
S85	-0.5148	-0.54096	-0.56937	-0.56286	-0.64189	-0.62667	-0.57598	-0.58588	-0.58011	-0.5593	-0.55356	-0.60712	-0.78509	-0.63362	-0.54355
S86	-0.13715	-0.17523	-0.12077	-0.10813	-0.05402	-0.11678	-0.17413	-0.04364	-0.16117	-0.17617	-0.09986	-0.11352	-0.2358	-0.19713	-0.11778
S87	-0.5148	-0.54096	-0.56937	-0.56286	-0.64189	-0.62667	-0.57598	-0.58588	-0.58011	-0.5593	-0.55356	-0.60712	-0.83884	-0.63362	-0.54355
S88	0.10112	0.17018	0.23946	0.27704	0.30298	0.25855	0.28882	0.21402	0.20376	0.17834	0.17553	0.16231	-0.0123	0.06925	0.14811

S89	0.12032	0.05964	0.05746	0.11181	0.10002	0.08492	0.09643	0.08469	0.09558	0.07427	0.0952	0.07627	-0.00711	0.12184	0.06294
S90	-0.34356	-0.41761	-0.33837	-0.29607	-0.33069	-0.24199	-0.23376	-0.26609	-0.29523	-0.30228	-0.36062	-0.42732	-0.52732	-0.19847	-0.37544
S91	0.15164	0.19583	0.20742	0.24429	0.25771	0.27188	0.29468	0.36145	0.395	0.36034	0.44022	0.48389	0.41319	0.47434	0.43723
S92	-0.32541	-0.25503	-0.24475	-0.21488	-0.27001	-0.26611	-0.24014	-0.28131	-0.28838	-0.31814	-0.32784	-0.30931	-0.48749	-0.41691	-0.4223
S93	-0.5148	-0.54096	-0.56937	-0.56286	-0.62287	-0.62667	-0.57598	-0.58588	-0.58011	-0.5593	-0.55356	-0.60712	-0.7375	-0.63362	-0.54355
S94	-0.5148	-0.54096	-0.56937	-0.56286	-0.64189	-0.62667	-0.57598	-0.58588	-0.58011	-0.5593	-0.55356	-0.60712	-0.83884	-0.63362	-0.54355
S95	-0.5148	-0.54096	-0.56937	-0.47951	-0.52739	-0.54815	-0.56294	-0.58588	-0.58011	-0.5593	-0.55356	-0.60712	-0.83884	-0.63362	-0.54355
S96	0.84202	0.87535	0.92388	0.86486	0.86195	0.80901	0.847	0.81891	0.81107	0.79486	0.75796	0.72229	0.53469	0.66123	0.77168
S97	0.43148	0.42222	0.4986	0.60046	0.5737	0.52594	0.5211	0.50271	0.43191	0.38468	0.34537	0.32935	0.06471	0.23763	0.31995
S98	-0.5148	-0.54096	-0.56937	-0.56286	-0.64189	-0.62667	-0.57598	-0.58588	-0.58011	-0.5593	-0.55356	-0.60712	-0.83884	-0.63362	-0.54355
S99	-0.37801	-0.3325	-0.26046	-0.23021	-0.27579	-0.24446	-0.25835	-0.29161	-0.35426	-0.39963	-0.38156	-0.4294	-0.61334	-0.44538	-0.4223
S100	-0.46106	-0.38919	-0.38379	-0.37708	-0.29702	-0.36092	-0.37677	-0.35905	-0.38624	-0.40696	-0.41533	-0.39732	-0.43254	-0.42635	-0.43344
S101	-0.5148	-0.54096	-0.50926	-0.56286	-0.64189	-0.62667	-0.57598	-0.58588	-0.58011	-0.5593	-0.55356	-0.5725	-0.62929	-0.62035	-0.53848
S102	-0.5148	-0.54096	-0.56937	-0.56286	-0.57854	-0.61938	-0.57598	-0.58588	-0.58011	-0.5593	-0.55356	-0.60712	-0.83884	-0.63362	-0.54355
S103	-0.5148	-0.54096	-0.56937	-0.56286	-0.59139	-0.62667	-0.53372	-0.58588	-0.53258	-0.5593	-0.55356	-0.60274	-0.5651	-0.57129	-0.54355
S104	-0.14405	-0.10881	-0.03552	-0.02156	-0.00214	0.00241	-0.05332	-0.05139	-0.03311	-0.07794	-0.12033	-0.09557	-0.21357	-0.15438	-0.12638
S105	-0.29762	-0.15326	-0.13491	-0.21185	-0.16712	-0.16962	-0.11247	-0.11277	-0.19439	-0.17657	-0.09334	-0.01613	-0.16896	-0.17215	-0.14496
S106	-0.5148	-0.52775	-0.48673	-0.56286	-0.61295	-0.62567	-0.57598	-0.57399	-0.58011	-0.5593	-0.55356	-0.60712	-0.59255	-0.5474	-0.54355
S107	-0.5148	-0.54096	-0.56937	-0.56286	-0.64189	-0.62667	-0.57598	-0.58588	-0.58011	-0.5593	-0.55356	-0.60712	-0.83884	-0.63362	-0.54355
S108	-0.5148	-0.54096	-0.56937	-0.55533	-0.55116	-0.49985	-0.52495	-0.54198	-0.55646	-0.5593	-0.55356	-0.60712	-0.74942	-0.63362	-0.54355
S109	-0.5148	-0.54096	-0.56937	-0.56286	-0.48508	-0.52874	-0.49096	-0.58588	-0.51193	-0.55755	-0.48705	-0.38356	-0.38632	-0.49812	-0.50657

Sample No.	integrated bins														
	5.80 .. 5.78	5.78 .. 5.76	5.76 .. 5.74	5.74 .. 5.72	5.72 .. 5.70	5.70 .. 5.68	5.68 .. 5.66	5.66 .. 5.64	5.64 .. 5.62	5.62 .. 5.60	5.60 .. 5.58	5.58 .. 5.56	5.56 .. 5.54	5.54 .. 5.52	5.52 .. 5.50
S1	-0.56222	-0.68144	-0.70504	-0.60075	-0.55432	-0.5573	-0.59916	-0.63951	-0.61273	-0.6031	-0.59889	-0.61527	-0.6396	-0.83505	-0.88284
S2	1.38092	1.30739	1.3414	1.34259	1.31445	1.34876	1.32262	1.2555	1.26778	1.26916	1.24477	1.20368	1.16096	1.17582	1.06659
S3	-0.56222	-0.68144	-0.70504	-0.60075	-0.55432	-0.5573	-0.59916	-0.63951	-0.61273	-0.6031	-0.59889	-0.61527	-0.6396	-0.60755	-0.63314
S4	1.40213	1.43542	1.44596	1.57571	1.67807	1.76306	1.78463	1.78782	1.87607	1.97671	2.07192	2.16242	2.24514	2.4217	2.34012
S5	-0.56222	-0.40849	-0.49166	-0.50394	-0.4537	-0.47485	-0.38372	-0.42089	-0.3669	-0.3581	-0.31012	-0.26416	-0.25892	-0.31084	-0.26568
S6	0.04596	0.07653	0.09059	-0.00759	0.0413	0.03037	0.07129	0.07511	0.04564	0.03668	0.06841	0.07474	0.07188	0.13239	0.11873
S7	0.58507	0.42866	0.42006	0.47705	0.53448	0.5214	0.51725	0.54613	0.5471	0.50747	0.5354	0.57421	0.58949	0.71202	0.73872
S8	-0.56222	-0.68144	-0.70504	-0.60075	-0.55432	-0.5573	-0.59916	-0.63951	-0.61273	-0.6031	-0.59889	-0.61527	-0.6396	-0.63702	-0.7561
S9	0.24817	0.37323	0.59454	0.38638	0.23696	0.23923	0.2935	0.3876	0.27889	0.27404	0.27018	0.30101	0.26699	0.12313	0.15134
S10	0.48963	0.5162	0.57777	0.37054	0.45885	0.40877	0.40564	0.38048	0.37838	0.35428	0.33766	0.31072	0.2696	0.36881	0.19551
S11	8.60295	8.52637	8.35441	8.55827	8.63713	8.62357	8.56307	8.49734	8.57405	8.5742	8.54008	8.48925	8.46627	8.19068	8.02522
S12	-0.56222	-0.45893	-0.51249	-0.5768	-0.55432	-0.5573	-0.53397	-0.53315	-0.56545	-0.54157	-0.52724	-0.54524	-0.49701	-0.42764	-0.35987
S13	-0.56222	-0.63008	-0.50602	-0.60075	-0.55432	-0.5573	-0.59916	-0.63951	-0.61273	-0.6031	-0.59889	-0.61527	-0.6396	-0.83505	-0.88284
S14	0.14316	0.05555	0.04232	0.03749	0.06886	0.08153	0.06225	0.08123	0.03366	0.04295	0.00104	0.02813	-0.00323	0.05163	0.06916
S15	-0.56222	-0.68144	-0.70504	-0.60075	-0.55432	-0.5573	-0.59916	-0.63951	-0.61273	-0.6031	-0.59889	-0.61527	-0.6396	-0.83505	-0.88284
S16	0.09298	0.12343	0.09407	0.05922	0.11506	0.09824	0.10984	0.10744	0.0595	0.0888	0.06536	0.07288	0.05381	0.07781	0.05256
S17	-0.42499	-0.25952	-0.13141	-0.34535	-0.3532	-0.3392	-0.28782	-0.29852	-0.29839	-0.29397	-0.25321	-0.23788	-0.2067	-0.10395	-0.21133
S18	0.44104	0.52973	0.71005	0.3983	0.46021	0.48568	0.43845	0.47551	0.39063	0.42288	0.46021	0.42584	0.39884	0.37699	0.35414
S19	2.25234	2.12609	2.49727	2.36058	2.33215	2.2955	2.21812	2.26242	2.2439	2.2195	2.17521	2.16106	2.12415	1.91255	2.2278
S20	-0.56222	-0.6053	-0.53523	-0.60075	-0.55432	-0.5573	-0.59916	-0.63951	-0.61273	-0.6031	-0.59889	-0.61527	-0.6396	-0.60012	-0.66863

S21	-0.56222	-0.68144	-0.70504	-0.60075	-0.55432	-0.5573	-0.59916	-0.63951	-0.61273	-0.6031	-0.59889	-0.61527	-0.6396	-0.83505	-0.88284
S22	1.098	0.76155	0.715	0.80532	0.82186	0.83636	0.80771	0.77383	0.81124	0.78384	0.81138	0.80432	0.83414	0.89107	0.95787
S23	0.69961	0.57019	0.42092	0.38602	0.41468	0.41916	0.39684	0.36952	0.36461	0.39082	0.37842	0.39149	0.36599	0.33051	0.51752
S24	-0.27787	-0.27998	-0.29485	-0.25433	-0.23658	-0.23207	-0.21444	-0.26508	-0.22243	-0.22239	-0.20028	-0.21065	-0.18945	-0.12691	-0.19792
S25	-0.10962	-0.0826	0.05499	-0.13722	-0.13785	-0.15363	-0.12972	-0.19368	-0.2217	-0.27127	-0.24551	-0.27114	-0.30439	-0.43288	-0.40759
S26	-0.36054	-0.43144	-0.22828	-0.42301	-0.38631	-0.44034	-0.45887	-0.48734	-0.49575	-0.5248	-0.48652	-0.54435	-0.57649	-0.6344	-0.67651
S27	-0.56222	-0.68144	-0.64575	-0.60075	-0.55432	-0.5573	-0.59916	-0.63951	-0.61273	-0.6031	-0.59889	-0.61527	-0.6396	-0.58755	-0.73266
S28	-0.56222	-0.68144	-0.70504	-0.60075	-0.55432	-0.5573	-0.59916	-0.63951	-0.61273	-0.6031	-0.59889	-0.61527	-0.6396	-0.83505	-0.88284
S29	-0.56222	-0.68144	-0.70504	-0.60075	-0.55432	-0.5573	-0.59916	-0.63951	-0.61273	-0.6031	-0.59889	-0.61527	-0.6396	-0.83505	-0.88284
S30	0.16327	0.28099	0.34367	0.15357	0.18372	0.18677	0.19168	0.16646	0.2323	0.17877	0.21382	0.21034	0.21006	0.25547	0.14083
S31	-0.15605	-0.11816	-0.13455	-0.11378	-0.11794	-0.16963	-0.14258	-0.18658	-0.18697	-0.18121	-0.17907	-0.18596	-0.16967	-0.26396	-0.318
S32	-0.56222	-0.68144	-0.60906	-0.60075	-0.55432	-0.5573	-0.59916	-0.5816	-0.61273	-0.6031	-0.59889	-0.59092	-0.52764	-0.54123	-0.60124
S33	-0.45408	-0.44112	-0.4775	-0.49333	-0.43576	-0.43353	-0.4359	-0.46016	-0.4391	-0.43914	-0.43656	-0.44978	-0.4415	-0.43423	-0.41474
S34	-0.01318	-0.01634	-0.03565	-0.06069	-0.03881	-0.0457	0.00194	-0.10294	-0.06456	-0.06846	-0.03843	-0.02682	-0.03089	0.13016	-0.12732
S35	0.47062	0.3873	0.52775	0.43756	0.46251	0.49403	0.455	0.41598	0.41658	0.43212	0.41346	0.43724	0.43437	0.35902	0.33468
S36	0.08965	0.08199	0.11379	0.04059	0.11035	0.15041	0.19892	0.12891	0.18787	0.14575	0.17861	0.1811	0.2054	0.21438	0.20616
S37	-0.04964	-0.14933	-0.19741	-0.17447	-0.14683	-0.12852	-0.1684	-0.19639	-0.20158	-0.19385	-0.19982	-0.22705	-0.20325	-0.15623	-0.09975
S38	-0.25291	-0.25603	-0.22208	-0.3057	-0.28706	-0.30506	-0.3492	-0.41237	-0.405	-0.40658	-0.47622	-0.48668	-0.49546	-0.42219	-0.57971
S39	-0.5039	-0.53127	-0.59451	-0.56338	-0.49685	-0.47818	-0.4946	-0.53856	-0.49926	-0.47487	-0.48069	-0.47923	-0.44785	-0.31526	-0.34365
S40	1.55585	1.53491	1.56177	1.63803	1.687	1.68761	1.70127	1.68957	1.72732	1.75964	1.82954	1.85736	1.91229	1.84856	1.88439
S41	0.305	0.26615	0.31758	0.26175	0.3119	0.27434	0.29938	0.2381	0.28646	0.26225	0.28107	0.27191	0.26246	0.39787	0.22641
S42	-0.02175	0.00219	0.0518	0.02096	0.01245	0.02918	-0.01539	-0.09058	-0.08365	-0.05689	-0.05817	-0.07216	-0.05847	0.0196	-0.12466
S43	-0.50422	-0.32499	-0.43241	-0.44382	-0.44453	-0.42215	-0.42443	-0.38899	-0.23939	-0.36922	-0.35932	-0.33618	-0.31092	-0.22603	-0.13368
S44	-0.46047	-0.31401	-0.41542	-0.4652	-0.44132	-0.42254	-0.38111	-0.38626	-0.30279	-0.37497	-0.40163	-0.38922	-0.36558	-0.27992	-0.25555
S45	-0.31083	-0.15767	-0.22501	-0.30341	-0.35135	-0.36063	-0.41232	-0.42461	-0.40571	-0.43735	-0.42811	-0.43396	-0.42685	-0.40364	-0.31219
S46	0.22185	0.17195	0.2	0.19273	0.20962	0.19417	0.2217	0.16948	0.14405	0.18584	0.20562	0.14095	0.15281	0.35687	0.19013
S47	0.12128	0.08582	0.20945	0.10139	0.09226	0.12618	0.12812	0.09791	0.08208	0.10093	0.09841	0.08662	0.10647	0.09821	0.05482
S48	-0.54227	-0.49416	-0.41975	-0.48434	-0.49195	-0.4846	-0.45997	-0.49373	-0.53424	-0.50775	-0.47645	-0.48093	-0.49141	-0.34242	-0.50384
S49	-0.56222	-0.68144	-0.70504	-0.60075	-0.55432	-0.5573	-0.59916	-0.63951	-0.61273	-0.6031	-0.59889	-0.61527	-0.6396	-0.83505	-0.88284
S50	1.51621	1.48227	1.25833	1.39448	1.48174	1.50768	1.51073	1.57032	1.61206	1.65573	1.72932	1.7894	1.86602	1.80567	1.98355
S51	0.06391	0.13001	0.22002	0.22467	0.04138	0.03949	0.04115	0.08096	0.04508	0.06409	0.0663	0.05677	0.01315	-0.09751	-0.0227
S52	0.71454	0.87853	0.93753	0.74323	0.76565	0.77925	0.79841	0.92702	0.78429	0.79906	0.84322	0.82842	0.83217	0.65859	0.66791
S53	-0.16595	-0.13974	0.04183	-0.11055	-0.17799	-0.19472	-0.15289	-0.13864	-0.18103	-0.19393	-0.17736	-0.19232	-0.21349	-0.13301	-0.16708
S54	-0.21876	-0.19477	-0.17046	-0.18841	-0.18749	-0.18912	-0.16335	-0.19585	-0.20928	-0.20541	-0.19247	-0.21923	-0.18959	-0.13464	-0.15264
S55	0.48998	0.54807	0.69948	0.61416	0.4716	0.49324	0.45347	0.55433	0.53188	0.48369	0.50805	0.5194	0.54873	0.42977	0.43194
S56	0.78755	0.81839	0.87529	0.78	0.76999	0.76391	0.74935	0.80166	0.73352	0.71724	0.71958	0.69218	0.64205	0.5414	0.50902
S57	-0.27318	-0.22862	-0.2454	-0.31078	-0.30803	-0.26063	-0.28253	-0.22233	-0.31446	-0.28174	-0.27263	-0.26574	-0.26523	-0.41767	-0.35577
S58	-0.56222	-0.5888	-0.679	-0.60075	-0.55432	-0.5573	-0.59916	-0.6171	-0.61273	-0.58485	-0.5819	-0.57689	-0.5491	-0.56775	-0.52074
S59	0.2294	0.13536	0.19764	0.22539	0.22198	0.24011	0.23026	0.2419	0.25919	0.22475	0.27335	0.28391	0.28109	0.33844	0.36084
S60	1.28202	1.27039	1.31541	1.21294	1.22729	1.1645	1.13885	1.085	1.00952	0.98894	0.93876	0.90445	0.83417	0.7609	0.59812
S61	-0.56222	-0.68144	-0.70504	-0.60075	-0.55432	-0.5573	-0.59916	-0.63951	-0.61273	-0.6031	-0.59889	-0.61527	-0.6396	-0.83505	-0.88284
S62	-0.33368	-0.32801	-0.33948	-0.31474	-0.28445	-0.27195	-0.24872	-0.2711	-0.26221	-0.2534	-0.20144	-0.20923	-0.17397	-0.18077	-0.26115
S63	-0.0355	0.15474	0.05064	-0.07591	-0.0876	-0.10596	-0.10969	-0.06283	-0.16388	-0.15796	-0.14124	-0.19145	-0.23125	-0.43276	-0.40609
S64	0.78456	0.95668	0.91266	0.92222	0.85428	0.88132	0.8912	0.89926	0.85298	0.89284	0.9057	0.89667	0.89228	0.84826	0.82054

S65	-0.56222	-0.68144	-0.70504	-0.60075	-0.55432	-0.5573	-0.59916	-0.63951	-0.61273	-0.6031	-0.59889	-0.61527	-0.6396	-0.83505	-0.87663
S66	0.66531	0.70257	0.68816	0.64289	0.65365	0.62489	0.61798	0.63583	0.54257	0.55574	0.5719	0.5457	0.52812	0.42327	0.41508
S67	-0.56222	-0.68144	-0.70504	-0.60075	-0.55432	-0.5573	-0.59916	-0.63951	-0.61273	-0.6031	-0.59889	-0.61527	-0.6396	-0.83505	-0.83107
S68	0.0617	0.11042	0.25305	0.04935	0.09317	0.05868	0.13925	0.07176	0.02415	0.04039	0.09235	0.02762	-0.01288	0.04688	-0.05429
S69	-0.54923	0.05126	-0.06879	-0.08747	-0.5034	-0.48986	-0.48034	-0.24868	-0.4114	-0.36669	-0.45235	-0.42532	-0.41407	-0.44619	-0.35681
S70	-0.42426	-0.4364	-0.42865	-0.40619	-0.43117	-0.43113	-0.37753	-0.34516	-0.37527	-0.40781	-0.41029	-0.3651	-0.3035	-0.18197	-0.0085
S71	-0.45603	-0.28013	-0.33585	-0.4105	-0.4541	-0.45513	-0.32824	-0.34192	-0.35452	-0.30193	-0.3634	-0.29913	-0.24679	-0.13791	0.00028
S72	-0.52034	-0.29048	-0.34017	-0.46679	-0.43411	-0.40082	-0.30492	-0.27556	-0.27324	-0.33561	-0.28371	-0.26591	-0.25752	-0.10465	-0.07611
S73	-0.56222	-0.68144	-0.70504	-0.60075	-0.55432	-0.5573	-0.59916	-0.63951	-0.61273	-0.6031	-0.59889	-0.61527	-0.6396	-0.83505	-0.88284
S74	0.14683	0.20296	0.13899	0.04497	0.06586	0.02987	0.11807	0.10344	0.00971	-0.00555	-0.07773	-0.1171	-0.09081	-0.10046	0.01112
S75	-0.2229	-0.24122	-0.28325	-0.23127	-0.1972	-0.17394	-0.17475	-0.10189	-0.10439	-0.11626	-0.10053	-0.08308	0.00712	0.35271	0.29096
S76	0.11555	0.24759	0.33084	0.14496	0.10071	0.08913	0.2336	0.38285	0.36783	0.37611	0.20289	0.30504	0.28913	0.46464	0.59648
S77	-0.56222	-0.68144	-0.70504	-0.60075	-0.55432	-0.5573	-0.59916	-0.63951	-0.61273	-0.6031	-0.59889	-0.61075	-0.57369	-0.38952	-0.29747
S78	-0.56222	-0.68144	-0.70504	-0.60075	-0.55432	-0.5573	-0.59916	-0.63951	-0.61273	-0.6031	-0.59889	-0.61527	-0.6396	-0.83505	-0.88284
S79	0.27915	0.45649	0.33269	0.23375	0.26811	0.24714	0.29359	0.34648	0.34071	0.33877	0.29889	0.31664	0.32341	0.45578	0.5278
S80	-0.05536	0.18103	0.22394	0.04734	0.02597	0.02195	0.1181	0.21993	0.15217	0.08656	0.14205	0.09922	0.10685	0.20805	0.28334
S81	0.27653	0.29862	0.15987	0.23292	0.21697	0.20368	0.2507	0.29183	0.22966	0.26177	0.21412	0.2321	0.21644	0.3875	0.3261
S82	-0.56222	-0.68144	-0.70504	-0.60075	-0.55432	-0.5573	-0.59916	-0.63951	-0.61273	-0.6031	-0.59889	-0.61527	-0.6396	-0.58947	-0.55353
S83	-0.56222	-0.68144	-0.70504	-0.60075	-0.55432	-0.5573	-0.59916	-0.63951	-0.61273	-0.6031	-0.59889	-0.61527	-0.6396	-0.83505	-0.88284
S84	-0.56222	-0.68144	-0.70504	-0.60075	-0.55432	-0.5573	-0.59916	-0.63951	-0.61273	-0.6031	-0.59889	-0.61527	-0.6396	-0.83505	-0.88284
S85	-0.56222	-0.07566	0.00603	0.80938	-0.55432	-0.5573	-0.59916	-0.44607	-0.27686	-0.44052	-0.57383	-0.42938	-0.40233	-0.29201	-0.16467
S86	-0.12293	-0.11796	-0.17238	-0.2062	-0.17848	-0.17105	-0.08804	-0.1187	-0.13058	-0.10704	-0.07839	-0.14409	-0.08979	-0.04728	0.09119
S87	-0.56222	-0.68144	-0.70504	-0.60075	-0.55432	-0.5573	-0.59916	-0.63951	-0.61273	-0.6031	-0.59889	-0.61527	-0.6396	-0.83505	-0.88284
S88	0.21758	0.23448	0.10053	0.15627	0.18556	0.21898	0.27175	0.33565	0.29482	0.37263	0.31536	0.34926	0.3389	0.47947	0.51482
S89	0.06497	0.0256	-0.01431	-0.00795	0.06573	0.01981	0.06867	0.04623	0.0553	0.04374	0.00054	0.02732	0.02774	0.10072	0.1887
S90	-0.38668	-0.50494	-0.27525	-0.36557	-0.34868	-0.33085	-0.40392	-0.31589	-0.30754	-0.20271	-0.30862	-0.32055	-0.3261	-0.42927	-0.12409
S91	0.53814	0.59637	0.66466	0.66032	0.56893	0.69585	0.64786	0.69887	0.81846	0.71142	0.71563	0.79945	0.81972	0.86346	0.90903
S92	-0.42761	-0.36624	-0.38302	-0.43377	-0.45743	-0.43866	-0.40136	-0.39094	-0.38859	-0.40881	-0.42974	-0.40011	-0.38852	-0.31968	-0.21805
S93	-0.5609	-0.60088	-0.70504	-0.60075	-0.55432	-0.5573	-0.59916	-0.59003	-0.60703	-0.58259	-0.59889	-0.61527	-0.61839	-0.53006	-0.51811
S94	-0.56222	-0.68144	-0.70504	-0.60075	-0.55432	-0.5573	-0.59916	-0.63951	-0.61273	-0.6031	-0.59889	-0.61527	-0.6396	-0.83505	-0.88284
S95	-0.56222	-0.68144	-0.70504	-0.60075	-0.55432	-0.5573	-0.59916	-0.63951	-0.61273	-0.6031	-0.59889	-0.61527	-0.6396	-0.56267	-0.42135
S96	0.74235	0.77343	0.63371	0.75475	0.70997	0.71172	0.76388	0.80843	0.74226	0.7728	0.67562	0.74549	0.71699	0.76067	0.82649
S97	0.29339	0.26393	0.19748	0.28856	0.25219	0.26151	0.28519	0.25979	0.30848	0.26279	0.28661	0.33835	0.36421	0.57349	0.62609
S98	-0.56222	-0.68144	-0.70504	-0.60075	-0.55432	-0.5573	-0.59916	-0.63951	-0.61273	-0.6031	-0.59889	-0.61527	-0.6396	-0.83505	-0.88284
S99	-0.38303	-0.43769	-0.46165	-0.42123	-0.37033	-0.37377	-0.36683	-0.34659	-0.26341	-0.31796	-0.26402	-0.24657	-0.22182	-0.02846	0.02493
S100	-0.33751	-0.13065	-0.24099	-0.35433	-0.36987	-0.39925	-0.37765	-0.26244	-0.32218	-0.31103	-0.33012	-0.34394	-0.29143	-0.18581	-0.1752
S101	-0.50407	-0.4604	-0.47756	-0.40669	-0.47252	-0.43859	-0.36952	-0.25612	-0.3694	-0.26634	-0.24752	-0.24737	-0.18579	-0.10205	0.02488
S102	-0.56222	-0.68144	-0.70504	-0.60075	-0.55432	-0.5573	-0.59916	-0.63951	-0.61273	-0.6031	-0.59889	-0.61527	-0.6396	-0.52125	-0.45528
S103	-0.56222	-0.20989	-0.24451	-0.60075	-0.55432	-0.5573	-0.4715	-0.40241	-0.50279	-0.4743	-0.52899	-0.52978	-0.49474	-0.55547	-0.49029
S104	-0.06742	0.14915	0.09362	-0.13764	-0.13379	-0.14047	-0.11201	-0.06239	-0.08533	-0.05961	-0.10633	-0.09648	-0.10067	-0.04034	0.03664
S105	-0.11607	-0.1011	-0.0644	-0.02063	-0.05696	-0.0952	-0.01525	0.12836	0.0523	-0.02405	0.07353	0.07087	0.10952	0.13051	0.22098
S106	-0.56222	-0.48978	-0.5665	-0.49803	-0.55432	-0.5573	-0.59916	-0.46876	-0.40798	-0.39839	-0.46388	-0.44751	-0.43004	-0.43456	-0.33479
S107	-0.56222	-0.68144	-0.70504	-0.60075	-0.55432	-0.5573	-0.59916	-0.63951	-0.61273	-0.6031	-0.59889	-0.61527	-0.6396	-0.83505	-0.88284
S108	-0.56222	-0.67037	-0.70504	-0.51828	-0.55432	-0.5573	-0.59777	-0.58504	-0.53195	-0.56789	-0.58603	-0.53611	-0.46081	-0.33445	-0.3264

S109	-0.40513	-0.36174	-0.44345	-0.26626	-0.43514	-0.43908	-0.428	-0.27495	-0.28934	-0.33722	-0.39517	-0.29538	-0.33148	-0.28075	-0.26035

Sample No.	integrated bins														
	5.50 .. 5.48	5.48 .. 5.46	5.46 .. 5.44	5.44 .. 5.42	5.42 .. 5.40	5.40 .. 5.38	5.38 .. 5.36	5.36 .. 5.34	5.34 .. 5.32	5.32 .. 5.30	5.30 .. 5.28	5.28 .. 5.26	5.26 .. 5.24	5.24 .. 5.22	5.22 .. 5.20
S1	-0.74641	-0.80274	-0.82409	-0.89686	-0.98289	-0.92666	-1.14347	-1.25489	-0.58138	-0.40265	-0.36514	-0.48862	-0.74462	-0.71349	-0.46175
S2	1.0946	1.0735	1.02843	0.94696	0.72058	0.3041	-0.11144	-0.54809	-0.3443	0.11282	0.19693	0.46184	1.1466	1.18876	1.34658
S3	-0.64423	-0.63679	-0.59331	-0.53548	-0.55303	-0.54299	0.14202	-0.04588	-1.01176	-1.06662	-1.08859	-1.24969	-1.03762	-0.71677	-0.29653
S4	2.49448	2.58161	2.75473	2.94739	2.94943	2.49113	2.04508	0.21847	-0.31572	-0.29198	-0.16381	0.43854	2.11385	2.31784	1.92155
S5	-0.16282	-0.0124	0.05772	0.11061	1.44242	1.1303	-0.83974	-0.72935	0.14509	1.59042	1.75541	0.35631	-0.33689	-0.24657	-0.0642
S6	0.06037	0.05075	0.15153	0.15724	0.28285	0.25006	0.62029	0.56319	0.20077	0.38115	0.37195	0.21794	-0.11828	-0.19576	-0.26882
S7	0.63536	0.67152	0.6253	0.665	0.74546	1.00181	0.84186	1.57482	1.12744	0.85458	0.8222	0.57841	-0.02207	-0.09196	-0.21995
S8	-0.74641	-0.76728	-0.69699	-0.67474	-0.62966	-0.46731	1.13827	0.31541	-0.77738	-0.95377	-0.98586	-1.14933	-1.05731	-0.90759	-0.84158
S9	0.28285	0.22306	0.20514	0.27529	-0.15154	-0.48722	-1.6595	-2.05542	-1.22633	-0.99811	-0.94662	-0.71528	0.37219	0.81311	0.97085
S10	0.18505	0.09942	0.04311	-0.02129	-0.26168	-0.71228	0.46523	-0.31992	-1.25297	-1.18341	-1.19848	-1.06844	-0.20673	0.16243	0.10895
S11	8.23764	8.09568	8.0287	7.7894	7.11168	5.21473	0.74949	-0.74033	0.1561	1.06046	1.53503	3.173	7.68935	7.91432	7.07249
S12	-0.44734	-0.45485	-0.39828	-0.48551	-0.36946	-0.3185	-0.40985	0.61263	0.88614	0.98975	0.97271	0.73603	-0.20292	-0.57795	-0.7442
S13	-0.74641	-0.80274	-0.82409	-0.85753	-0.39911	-0.54426	-1.05089	-0.65063	-0.67077	-0.15246	-0.07244	-0.17989	-0.65711	-0.65676	-0.53093
S14	-0.00925	0.11253	-0.02783	-0.06627	0.10435	0.15258	-0.16488	0.80837	0.83466	1.4247	1.51054	0.60423	0.11097	-0.14338	-0.39344
S15	-0.74641	-0.80274	-0.82409	-0.89686	-1.1619	-1.41417	-1.67251	-1.80924	-1.44718	-1.51441	-1.64167	-1.76322	-1.22169	-0.90896	-0.51646
S16	0.08165	0.02112	0.08847	0.10112	0.62415	0.60736	-0.14656	-0.07188	-0.20944	-0.04523	0.04317	-0.02832	-0.00474	-0.10378	0.00794
S17	-0.17734	-0.16082	-0.11939	-0.13664	-0.18714	-0.15666	0.25478	-0.1922	-1.34279	-1.2194	-1.21661	-1.24215	-0.64426	-0.2204	0.40052
S18	0.37489	0.33505	0.3456	0.27289	0.04456	-0.20248	-0.56627	-0.20042	-0.85374	-0.84308	-0.77883	-0.63614	0.12936	0.38774	0.77972
S19	2.04586	1.93086	1.89251	1.76283	1.3666	0.53344	-1.01903	-0.13375	0.56117	-1.11172	-0.98016	-0.45302	1.40902	1.67846	1.17761
S20	-0.70476	-0.7294	-0.69456	-0.71424	-0.8113	-0.79558	0.12979	-0.20934	-0.99305	-1.22159	-1.26402	-1.34207	-0.93408	-0.66432	-0.2995
S21	-0.74641	-0.80274	-0.82409	-0.87795	-0.98474	-1.20961	-1.67278	-1.72575	-0.76931	-0.92853	-0.94478	-0.98048	-0.589	-0.34348	-0.50961
S22	0.76416	0.83919	0.8098	0.83433	1.12799	1.69821	0.84114	1.79618	1.13374	0.9114	0.86203	0.67533	0.29515	0.21875	-0.13442
S23	0.36296	0.30965	0.36474	0.31967	0.28765	0.45259	-0.80634	0.88911	0.82765	-0.1397	0.06126	0.50162	0.34007	-0.05412	-0.47095
S24	-0.19702	-0.19247	-0.15407	-0.18828	-0.03924	0.09007	0.08926	0.52418	0.14976	0.38999	0.51089	0.45178	-0.20854	-0.38612	-0.40579
S25	-0.40747	-0.50223	-0.53608	-0.60255	-0.84926	-1.04761	-1.08965	-1.02122	-1.21556	-1.15343	-1.16705	-1.18176	-0.55342	-0.31638	-0.15654
S26	-0.74355	-0.79226	-0.78408	-0.89122	-1.14775	-1.46842	-0.98681	-0.26702	-1.20213	-1.27329	-1.31757	-1.35709	-0.805	-0.58793	-0.70262
S27	-0.71239	-0.78572	-0.73918	-0.74935	-0.89072	-0.85813	0.60658	-0.05652	-1.24411	-1.31256	-1.37879	-1.49423	-1.05545	-0.75722	-0.5477
S28	-0.74641	-0.80274	-0.82409	-0.89686	-1.07384	-1.0975	-0.14013	-0.21778	-1.48772	-1.61457	-1.72747	-1.84083	-1.22169	-0.92588	-0.7549
S29	-0.74641	-0.80274	-0.82409	-0.89686	-0.89787	-0.85091	0.12907	-0.30974	-1.39373	-1.46832	-1.52828	-1.68583	-1.22169	-0.92588	-0.49231
S30	0.18253	0.13053	0.16814	0.14137	0.09206	-0.14455	0.05659	-0.27205	-1.13155	-0.69986	-0.53303	-0.72637	-0.12437	0.15364	0.36015
S31	-0.24673	-0.29462	-0.26778	-0.30566	-0.49277	-0.04677	-0.90259	-1.55144	-0.78553	-0.65901	-0.59447	-0.62819	-0.29409	-0.16753	-0.2318
S32	-0.48329	-0.46757	-0.30526	-0.26973	-0.25791	-0.46112	-0.91114	-1.40611	-1.35568	-1.2074	-1.20196	-1.1941	-0.45053	0.02111	0.45546
S33	-0.43205	-0.45576	-0.38333	-0.46582	-0.48298	-0.48036	-0.42917	-0.16519	0.091	0.38487	0.54646	0.32976	-0.19433	-0.40069	-0.62173
S34	-0.12981	-0.13332	-0.1349	-0.12016	-0.2156	-0.12574	1.98185	0.35711	-1.02847	-0.85625	-0.82621	-0.8932	-0.50182	-0.33744	-0.33402
S35	0.36024	0.27734	0.25384	0.2526	-0.02955	-0.37716	-0.63693	-0.57199	-1.24565	-1.22807	-1.22525	-1.08906	-0.04072	0.32859	0.44578
S36	0.17385	0.19953	0.21766	0.20424	0.09733	-0.07774	0.30925	0.08763	-0.82371	-0.9519	-0.93339	-0.87278	-0.29368	-0.01721	0.1914
S37	-0.23518	-0.26691	-0.30365	-0.34929	-0.20483	0.269	0.55464	1.52299	0.84043	0.42322	0.35263	0.1507	-0.1872	-0.2151	-0.3896
S38	-0.6471	-0.74511	-0.77551	-0.8827	-1.02963	-1.07875	0.69009	0.32944	-0.5363	-0.62757	-0.66633	-0.66998	-0.32672	-0.36931	-0.59702
S39	-0.43603	-0.44552	-0.3596	-0.39692	-0.3317	-0.16769	0.0047	1.33381	0.7739	0.9791	1.00143	0.80396	-0.27948	-0.68236	-1.02518
S40	1.98081	2.02716	2.11064	2.23174	2.17655	1.81215	0.20508	-0.35969	-0.94857	-0.87348	-0.76467	-0.36445	1.39078	1.75797	1.53852

S41	0.19849	0.14919	0.12209	0.10817	-0.01089	-0.17471	1.20927	0.41529	-1.28736	-1.29376	-1.30672	-1.21439	-0.40918	-0.05731	-0.11104
S42	-0.11747	-0.16807	-0.17133	-0.19244	-0.38335	-0.39236	0.58584	-0.11416	-1.07817	-1.15954	-1.174	-1.12059	-0.42661	-0.11012	-0.09226
S43	-0.05876	0.00109	-0.09628	0.01396	0.15885	0.37431	0.0735	0.35182	1.17298	2.54702	2.36212	1.80892	0.46145	-0.19399	0.06889
S44	-0.25031	-0.09281	-0.25695	-0.20574	0.27896	0.46744	0.21231	0.29244	1.06425	2.84929	2.68495	2.47612	0.45223	-0.39024	-0.38988
S45	-0.44884	-0.40419	-0.50838	-0.59271	-0.67093	-0.49315	-0.1022	1.0373	1.32181	1.85698	1.82818	1.85768	0.45817	-0.26992	-0.68874
S46	0.12407	0.0792	0.09109	0.08607	-0.015	-0.08211	1.31863	0.51352	-0.74758	-0.94312	-0.9403	-0.87977	-0.33177	-0.1177	-0.16855
S47	0.0396	-0.00135	-0.01597	-0.05437	-0.17654	-0.21152	0.009	-0.29275	-1.07019	-1.09	-1.0822	-1.02631	-0.29543	-0.02552	-0.05837
S48	-0.49419	-0.49756	-0.4762	-0.48649	-0.57472	-0.59316	0.86679	0.04553	-1.25804	-1.24356	-1.26707	-1.31217	-0.83906	-0.5205	-0.39792
S49	-0.74641	-0.80274	-0.82409	-0.89686	-1.1619	-1.20659	-0.82734	-0.6748	-0.87369	-1.08712	-1.11953	-1.2088	-0.95652	-0.80093	-0.72907
S50	2.03885	2.19798	2.30106	2.44137	2.36871	1.76884	-0.33036	-0.57562	-0.0125	-0.37675	-0.28854	0.02782	1.20662	1.55916	1.38941
S51	0.052	-0.00808	-0.01825	-0.07583	-0.25748	-0.67014	-1.58488	-1.4547	-0.72736	-0.66474	-0.63646	-0.72269	0.02932	0.74848	0.35253
S52	0.71769	0.72585	0.74724	0.67003	0.39717	0.03493	-1.1944	-1.89876	-1.15147	-0.81746	-0.73001	-0.44314	0.84062	1.33528	2.01114
S53	-0.2258	-0.28075	-0.2308	-0.25	-0.34575	-0.43519	0.30499	0.13219	-0.72951	-0.90407	-0.90027	-0.89882	-0.45908	-0.27639	-0.10694
S54	-0.21736	-0.22184	-0.22634	-0.26521	-0.25219	-0.25855	0.00179	0.23349	0.03303	-0.23131	-0.1715	-0.26192	-0.25329	-0.27227	-0.3135
S55	0.48272	0.43859	0.43589	0.5459	0.5593	0.30953	-0.91639	-0.96214	-0.14733	0.44711	0.59069	0.51165	0.53082	0.52998	0.88044
S56	0.56435	0.54458	0.59798	0.45138	0.41328	0.12668	-0.94689	-1.21578	-0.54239	0.29121	0.57373	0.76221	0.78269	0.62784	0.64319
S57	-0.32021	-0.35766	-0.27456	-0.36182	-0.35237	-0.52704	-1.18334	-1.01395	-0.28622	0.06734	0.18429	0.09661	-0.06476	-0.15311	0.05524
S58	-0.51155	-0.52149	-0.38597	-0.44151	-0.2149	-0.25748	-0.80584	-0.06169	-0.01922	0.34594	0.42376	0.16306	-0.46139	-0.58402	-0.57862
S59	0.30399	0.28177	0.369	0.33126	0.42017	0.56083	0.04107	0.7393	1.05595	1.31288	1.29904	1.29688	0.23349	-0.06662	-0.26521
S60	0.60552	0.4708	0.42592	0.28668	-0.08133	-0.33906	-0.20043	-0.84725	-1.04446	-0.74271	-0.64986	-0.52051	0.52075	0.68109	0.68211
S61	-0.74641	-0.80274	-0.82409	-0.89686	-1.1619	-0.87945	-0.5041	-0.82506	-0.84673	-1.09707	-1.11256	-1.3332	-1.22169	-0.92588	-0.74596
S62	-0.18754	-0.16938	-0.08586	-0.01397	-0.04411	0.64834	0.76985	-0.8141	-1.40849	-1.45262	-1.51553	-1.48731	-0.64443	-0.2053	-0.32385
S63	-0.32649	-0.39517	-0.37209	-0.46081	-0.67249	-1.03551	-1.63335	-1.86989	-0.90838	-0.44279	-0.38166	-0.42849	-0.05198	-0.02046	0.14153
S64	0.91874	0.85714	0.91986	0.84884	0.73132	0.85956	-0.313	-1.25661	-0.86252	-0.59945	-0.50684	-0.33185	0.68094	1.11526	1.71327
S65	-0.74641	-0.78315	-0.73796	-0.69945	-0.94658	-1.341	-1.92511	-2.19113	-1.45032	-1.47799	-1.51866	-1.40557	-0.526	-0.02954	0.02948
S66	0.43792	0.36297	0.38734	0.26781	0.00662	-0.37767	-1.34936	-1.24073	-0.42151	-0.58392	-0.53275	-0.28092	0.36794	0.44147	0.20963
S67	-0.71693	-0.74883	-0.6915	-0.68646	-0.898	-1.18172	-1.82783	-2.10946	-1.27503	-1.0602	-1.054	-0.77772	-0.32814	-0.02112	0.06769
S68	-0.05184	-0.07922	-0.08387	-0.0891	-0.1921	-0.37831	-0.63241	-0.57566	-1.03502	-0.90962	-0.85835	-0.83924	-0.22967	-0.08994	-0.37257
S69	-0.33034	-0.26498	-0.37747	-0.38294	-0.46494	-0.66879	-1.18184	-1.06274	-0.05922	0.30907	0.30633	0.27448	0.27112	0.64559	0.37858
S70	-0.21008	-0.16394	-0.14957	-0.14284	0.07604	0.62019	0.56378	1.73127	1.54403	1.07195	0.98839	0.8175	-0.083	-0.265	-0.42867
S71	-0.13946	-0.0836	-0.05614	0.05848	0.24938	0.55481	0.23553	0.97137	0.86486	0.29276	0.2035	0.11813	-0.37008	-0.23732	-0.17486
S72	-0.12562	-0.11638	-0.05982	0.03075	0.0443	-0.08816	0.5013	0.18105	-0.04039	-0.23885	-0.31798	-0.17787	-0.18929	-0.04817	0.34635
S73	-0.74641	-0.80274	-0.82409	-0.59733	-0.47028	-0.22279	2.81709	0.32626	-0.0833	-1.23122	-1.63088	-1.67067	-1.22169	-0.92588	-1.45778
S74	-0.14963	-0.16062	-0.23672	-0.32951	-0.35437	-0.45309	-0.2957	0.53024	1.07357	0.78768	0.74032	1.01277	0.73431	0.25864	-0.00137
S75	0.18894	0.27059	0.37284	0.76284	1.83885	4.36426	3.51605	2.52362	1.07479	1.23752	1.12044	0.49429	-0.10004	-0.17122	-0.44284
S76	0.53402	0.51065	0.42724	0.51075	0.24907	-0.1607	-0.76362	-0.63732	0.09642	0.18476	0.25859	1.24189	1.99809	2.08873	1.99541
S77	-0.4572	-0.44125	-0.35942	-0.32511	-0.04165	0.51429	0.68881	1.59034	1.29989	1.21914	1.08354	0.84214	-0.32675	-0.54383	-0.71541
S78	-0.74641	-0.80274	-0.82409	-0.89686	-0.8206	-0.51239	-0.50403	-0.03515	0.62412	0.64981	0.57289	0.40139	-0.43584	-0.50645	-0.20194
S79	0.42654	0.43383	0.39309	0.37974	0.32422	0.19118	-0.13867	0.12979	0.49649	0.34188	0.4118	0.76436	0.74479	0.68649	0.58898
S80	0.22466	0.27157	0.20066	0.22113	0.25358	0.01023	-0.33275	-0.13023	0.33766	0.24259	0.22575	0.58242	0.51315	0.48341	0.99437
S81	0.26874	0.28946	0.22403	0.20876	0.18172	0.01187	1.38486	0.81596	0.37466	-0.04403	-0.10872	0.02669	0.11395	0.00315	-0.24785
S82	-0.53443	-0.4118	-0.26961	0.01645	0.58988	1.32815	1.6673	1.27781	0.3549	0.2398	0.1036	-0.50612	-1.22169	-0.92588	-1.02179
S83	-0.74641	-0.80274	-0.82409	-0.89686	-1.1619	-1.70481	-1.85624	-0.8282	2.73889	-0.12476	-0.07586	0.46668	0.57039	-0.71637	-1.27193
S84	-0.74641	-0.80274	-0.82409	-0.89686	-1.1619	-1.70481	-2.06523	-1.29194	1.6706	-0.9596	-1.12737	-1.40557	-1.22169	-0.92588	-1.45778

S85	0.0016	0.2059	0.0108	0.22818	0.02821	-0.20073	-0.91162	-1.28405	-0.29778	0.62255	0.61343	1.1865	1.25746	1.3365	2.6934
S86	-0.02903	-0.02706	-0.0305	0.06802	0.12352	0.0775	0.09661	0.91552	1.07911	0.57499	0.56618	0.61174	-0.008	-0.3019	-0.45729
S87	-0.74641	-0.80274	-0.82409	-0.89686	-0.69949	-0.42362	-0.41652	-0.18485	0.49009	0.94928	0.84818	0.76119	-0.05243	-0.44267	-0.31859
S88	0.47384	0.54983	0.60953	0.63705	0.71854	0.6714	0.693	0.609	0.50006	0.67444	0.59886	0.62455	0.2481	0.0397	0.07959
S89	0.02392	0.00826	0.03983	0.04047	-0.01602	-0.22879	0.02806	0.914	0.63641	-0.0444	-0.08364	0.01377	-0.08908	-0.19983	-0.41606
S90	-0.19484	-0.28133	-0.10536	-0.24605	-0.35444	-0.52256	-1.3135	-0.51045	3.79859	0.64304	1.06598	1.94489	1.66398	-0.25814	-0.81708
S91	1.0142	1.10885	0.97483	1.10831	1.23908	1.31964	0.4251	0.29492	0.6193	0.82731	0.79719	0.96508	0.67322	0.59434	0.7599
S92	-0.38634	-0.29571	-0.42069	-0.34937	-0.23274	0.21832	0.78113	1.55455	1.17808	0.98599	0.8975	0.75434	-0.04118	-0.26026	-0.34668
S93	-0.5656	-0.54262	-0.51861	-0.53254	-0.43106	-0.14025	0.43581	0.27078	0.15613	0.14539	0.12307	-0.00894	-0.38628	-0.50705	-0.57961
S94	-0.74641	-0.80274	-0.82409	-0.80867	-0.63299	-0.29082	0.34884	0.12294	0.55403	1.29558	1.1998	1.13373	-0.65841	-0.92588	-0.46695
S95	-0.60111	-0.50812	-0.53304	-0.53271	-0.37418	0.07373	0.76272	1.6116	1.42792	0.95771	0.85112	0.7537	-0.35633	-0.69505	-0.91232
S96	0.78101	0.80663	0.78933	0.7456	0.65409	0.55732	0.08723	0.2958	0.81709	1.03365	1.06014	1.26351	0.79649	0.48571	0.42452
S97	0.45478	0.54818	0.47115	0.52757	0.92976	1.40596	1.51641	2.03149	1.46968	1.35321	1.24678	1.10848	0.13746	-0.13203	-0.42881
S98	-0.74641	-0.80274	-0.82409	-0.89686	-1.11299	-0.71086	0.86137	0.14397	0.20851	0.79445	0.73351	0.46758	-0.67532	-0.89884	-0.79215
S99	-0.07234	0.1288	-0.06253	0.06606	0.58081	1.34433	1.20867	2.10627	1.62145	2.08121	1.92118	1.5571	-0.17927	-0.63025	-0.94927
S100	-0.22805	-0.14157	-0.19325	-0.16505	-0.19185	-0.25655	0.31868	0.47924	0.62883	0.67066	0.66689	0.74399	0.0387	-0.24031	-0.27866
S101	0.06361	0.14149	0.19277	0.39035	0.7005	0.9088	0.44021	0.18374	0.49114	0.73843	0.69479	0.66275	0.0083	-0.03469	0.21937
S102	-0.61277	-0.57067	-0.59258	-0.50762	-0.46725	-0.19857	1.20414	1.63577	1.06198	0.80186	0.71588	0.53785	-0.39374	-0.5794	-0.74438
S103	-0.47139	-0.45309	-0.43359	-0.4615	-0.56212	-0.72743	-0.97732	-0.65804	-0.5471	-0.62173	-0.63987	-0.4138	-0.19273	-0.08906	0.07825
S104	-0.04205	0.0667	-0.00655	-0.02911	-0.04798	-0.16079	-0.17953	0.34506	0.74924	1.04734	1.07726	1.12897	0.40094	-0.08784	-0.15946
S105	0.16965	0.26186	0.18172	0.27444	0.28825	0.42588	1.12907	0.5006	0.16876	0.21091	0.17961	0.26365	0.31134	0.24966	0.36887
S106	-0.35603	-0.18836	-0.3333	-0.33464	-0.31461	-0.4806	-0.95524	-0.3537	0.59972	0.75943	0.64241	0.41784	0.17441	0.22448	0.45858
S107	-0.74641	-0.80274	-0.82409	-0.71112	-0.62881	-0.42296	0.00497	0.11249	0.20596	0.30093	0.23917	0.12601	-0.99837	-0.92588	-0.64815
S108	-0.3094	-0.19034	-0.24451	-0.1289	0.07664	0.7604	1.01269	0.94327	1.31457	2.4634	2.52387	2.38352	0.37754	-0.36571	-0.49665
S109	-0.21143	-0.17704	-0.18431	-0.10913	-0.14453	-0.12685	-0.32712	-0.433	-0.21437	0.13171	0.16711	0.17434	-0.00832	0.01258	0.3271

Sample No.	integrated bins														
	5.20 .. 5.18	5.18 .. 5.16	5.16 .. 5.14	5.14 .. 5.12	5.12 .. 5.10	5.10 .. 5.08	5.08 .. 5.06	5.06 .. 5.04	5.04 .. 5.02	5.02 .. 5.00	5.00 .. 4.98	4.98 .. 4.96	4.96 .. 4.94	4.94 .. 4.92	4.92 .. 4.90
S1	-0.72292	0.62628	-0.53767	-0.65141	-0.66323	-0.66263	-0.65752	-0.68667	-0.52135	-0.51525	-0.57342	-0.55238	-0.53403	-0.52925	-0.58284
S2	0.82649	-0.19163	0.98923	1.09586	1.10664	1.15639	1.10486	1.00796	1.08814	1.08844	1.04676	1.07406	1.08776	0.82087	0.84824
S3	-0.82268	-1.26367	-0.70043	-0.53566	-0.49147	-0.47605	-0.45948	-0.5476	-0.37547	-0.30052	-0.37919	-0.35179	-0.16003	-0.30059	-0.79969
S4	1.98938	-0.01557	2.17053	2.30122	2.3601	2.65778	2.50846	2.30874	2.05203	2.91145	2.98358	3.01062	2.93667	2.08852	2.39864
S5	1.17499	2.49444	-0.55789	-0.36127	-0.29152	-0.27915	-0.19269	0.37189	-0.05946	-0.13596	-0.12541	-0.08226	-0.04816	0.88855	0.79145
S6	-0.02494	0.61016	-0.30468	-0.24234	-0.21142	-0.12632	-0.14624	-0.16893	-0.32	-0.04187	-0.103	-0.05729	-0.11033	0.03912	0.04473
S7	1.00216	1.26111	-0.28691	-0.22828	-0.16231	0.0144	-0.03888	-0.16669	-0.34646	0.09773	0.11509	0.09054	0.18291	0.5139	0.61809
S8	-1.00021	-0.40089	-0.8879	-0.95534	-0.88404	-0.81046	-0.7959	-0.86243	-0.90767	-0.74836	-0.70015	-0.66053	-0.7189	-1.06543	-1.05222
S9	0.71322	0.46914	0.94941	1.02181	0.73757	0.81503	0.74622	0.82213	1.28251	0.80576	0.68151	0.66736	0.542	-0.19091	-0.13622
S10	-0.47521	-1.62887	0.10808	0.39556	0.40676	0.15828	0.14453	0.09373	0.21694	0.00732	-0.03018	-0.04184	-0.22183	-0.86674	-0.92486
S11	6.67775	1.11355	7.03873	7.53206	7.44715	7.78096	7.37895	7.05041	6.60354	7.65827	7.61144	7.48349	7.24581	5.66727	6.20697
S12	-0.07672	1.26912	-0.60154	-0.66281	-0.63579	-0.58786	-0.59223	-0.59283	-0.74069	-0.49429	-0.57584	-0.58371	-0.51515	-0.02468	0.10208
S13	-0.44831	1.0906	-0.69067	-0.63681	-0.63921	-0.69558	-0.59041	-0.47103	-0.55038	-0.59038	-0.69856	-0.70652	-0.77742	-0.77116	-0.76239
S14	0.81154	1.91544	-0.18364	-0.2361	-0.24862	-0.18398	-0.2943	-0.31965	-0.59129	-0.20737	-0.1903	-0.2241	-0.23244	0.53904	0.37495
S15	-1.06821	-1.31236	-0.36856	-0.24569	-0.53786	-0.78302	-0.73602	-0.44388	0.00758	-0.84349	-0.76753	-0.80077	-0.92577	-1.60072	-1.82647
S16	-0.00801	1.06414	-0.15658	-0.15108	-0.16309	-0.07625	-0.12363	0.03939	-0.23752	-0.10021	-0.05325	-0.02646	0.01619	-0.04761	-0.02896

S17	-0.36496	-1.2104	-0.26664	-0.11849	-0.07975	0.0345	0.04479	-0.09965	0.03199	0.06627	0.04152	0.12272	0.42965	0.11895	-0.57596
S18	0.21815	-0.90707	0.39691	0.4723	0.41978	0.49988	0.45426	0.27159	0.49726	0.45545	0.41959	0.45935	0.65776	0.4165	-0.08325
S19	1.86973	-1.40579	3.09046	1.67843	1.72277	2.17974	1.78199	1.5584	1.65793	2.08322	1.97851	1.84117	1.72875	1.39088	0.94302
S20	-0.95005	-1.46273	-0.41409	-0.60728	-0.62085	-0.48472	-0.51101	-0.65917	-0.58231	-0.53853	-0.52145	-0.45186	-0.38924	-0.6671	-1.07635
S21	-0.48405	-0.6258	-0.32051	-0.15893	-0.06695	0.08198	0.11171	0.12549	0.76865	0.24407	0.22269	0.29929	0.32511	-0.12814	0.33889
S22	0.76363	0.84585	0.01337	0.04044	0.07588	0.23629	0.14079	0.01743	-0.16463	0.24614	0.3096	0.29298	0.32663	0.51284	0.57106
S23	-0.66006	0.82491	0.07397	-0.11115	-0.14081	0.14802	-0.02566	-0.18728	-0.19711	0.13703	0.21228	0.20073	0.09817	-0.28208	0.52224
S24	-0.09718	1.65458	-0.54984	-0.55012	-0.50791	-0.38827	-0.4102	-0.49587	-0.57195	-0.31755	-0.27812	-0.27244	-0.26765	-0.10509	0.11567
S25	-0.68067	-0.4578	-0.19394	-0.25962	-0.32031	-0.26325	-0.33727	-0.43149	-0.2983	-0.35982	-0.406	-0.40176	-0.36425	-0.70627	-1.17756
S26	-1.18688	-1.09106	-0.55751	-0.72509	-0.73163	-0.61854	-0.63122	-0.85872	-0.80327	-0.70207	-0.66468	-0.65814	-0.73064	-1.21731	-1.46842
S27	-1.11969	-0.81626	-0.71779	-0.73582	-0.72189	-0.68948	-0.71572	-0.82591	-0.85269	-0.70892	-0.6554	-0.61523	-0.54852	-0.93403	-1.33671
S28	-1.29622	-1.39289	-0.85436	-0.88573	-0.86301	-0.75761	-0.76424	-0.91838	-0.79758	-0.72724	-0.65627	-0.61083	-0.49562	-0.85604	-1.46077
S29	-1.07807	-0.97634	-0.97319	-0.88799	-0.81193	-0.77406	-0.70157	-0.7989	-0.57604	-0.70307	-0.6405	-0.58379	-0.38968	-0.7127	-1.28267
S30	0.03456	-0.07925	0.03128	0.14667	0.15567	0.18336	0.20872	0.15266	0.25997	0.17325	0.14853	0.18433	0.08226	-0.22658	-0.40264
S31	0.22173	1.29134	-0.01005	-0.05774	-0.10243	-0.17339	-0.22413	-0.30604	-0.32814	-0.19036	-0.19348	-0.18686	-0.24201	-0.53757	0.46851
S32	-0.01327	-0.49697	-0.1814	-0.08993	-0.10917	-0.021	-0.00766	-0.06788	0.11056	0.01989	0.13086	0.19389	0.30294	-0.11717	-0.41511
S33	-0.40166	1.28742	-0.49827	-0.56945	-0.56484	-0.49173	-0.50203	-0.56537	-0.60642	-0.39997	-0.42678	-0.4414	-0.4362	-0.27745	-0.02598
S34	-0.58384	-0.02347	-0.42715	-0.42817	-0.39926	-0.27349	-0.28557	-0.47124	-0.60031	-0.21815	-0.19194	-0.17185	-0.07932	-0.41882	-0.64189
S35	-0.0489	-0.41773	0.27908	0.3545	0.32942	0.46957	0.40753	0.19267	0.30902	0.45357	0.45581	0.45997	0.68438	0.41218	-0.4135
S36	-0.24522	-0.52058	-0.09168	0.03662	0.05557	0.15272	0.12558	0.00893	0.02351	0.21259	0.25583	0.27287	0.39443	0.01653	-0.29954
S37	0.08533	0.35487	-0.29645	-0.26789	-0.25719	-0.30344	-0.3217	-0.37898	-0.51489	-0.38665	-0.35824	-0.37121	-0.40887	-0.3258	-0.2413
S38	-0.9526	-0.85694	-0.34332	-0.44579	-0.48904	-0.49818	-0.54111	-0.69423	-0.73939	-0.61785	-0.62142	-0.56627	-0.75455	-1.09083	-1.10993
S39	0.71594	2.67273	-0.72585	-0.88333	-0.8608	-0.68598	-0.76986	-0.88522	-1.05384	-0.68545	-0.63194	-0.65892	-0.58498	-0.01024	0.06326
S40	1.25784	-0.46062	1.61336	1.91402	1.92136	2.17221	2.09749	1.94757	1.98841	2.46252	2.58292	2.69189	2.66121	1.7474	2.03643
S41	-0.64136	-1.39593	-0.08478	0.02386	0.04932	0.0971	0.06409	-0.04526	-0.12692	0.08392	0.07391	0.0836	0.05045	-0.56091	-0.75302
S42	-0.62487	-1.04147	-0.08049	0.0447	0.00423	-0.04063	-0.05148	-0.09228	-0.11318	-0.06528	-0.10715	-0.06256	-0.12967	-0.6942	-0.81461
S43	1.27518	1.13796	-0.43949	-0.28851	-0.27995	-0.41694	-0.46407	-0.27867	-0.24145	-0.38001	-0.35266	-0.33799	-0.13048	1.09795	1.30221
S44	0.52771	0.95534	-0.61784	-0.6003	-0.58925	-0.52475	-0.54816	-0.34322	-0.57253	-0.56397	-0.51894	-0.52197	-0.28493	1.13571	1.06342
S45	0.09628	0.88735	-0.42621	-0.52842	-0.53129	-0.4935	-0.54481	-0.72708	-0.88337	-0.5667	-0.57404	-0.61275	-0.52569	0.38256	0.41876
S46	-0.5831	-0.99148	-0.13808	-0.08535	-0.07385	0.02018	-0.02298	-0.12724	-0.22355	0.03827	0.06382	0.09353	0.06159	-0.44381	-0.51841
S47	-0.4969	-0.65168	0.00401	0.06591	0.02302	0.09301	0.07397	-0.01739	-0.00424	0.12345	0.11407	0.11028	0.0366	-0.50862	-0.61027
S48	-0.86023	-0.97173	-0.42297	-0.32185	-0.354	-0.37796	-0.35637	-0.40007	-0.40319	-0.36802	-0.37523	-0.36649	-0.39452	-0.87559	-1.08444
S49	-1.16848	-0.60348	-0.5347	-0.66277	-0.7193	-0.67131	-0.73806	-0.80607	-0.63693	-0.7215	-0.75427	-0.74791	-0.77513	-1.13542	-1.33505
S50	1.79702	0.93472	0.96795	1.4457	1.66226	2.00378	2.06861	2.02051	2.19543	2.83488	3.23439	3.37121	3.43616	2.6201	3.37259
S51	0.26923	1.47469	0.48819	0.89224	0.70483	0.34908	0.39554	0.4539	0.62051	0.23148	0.16231	0.1084	-0.00663	-0.35988	-0.40404
S52	1.67295	1.65705	0.89515	1.18599	1.23523	1.21362	1.25762	1.24125	1.47396	1.25151	1.21207	1.23548	1.39934	1.02443	0.43054
S53	-0.57266	-0.76402	-0.30487	-0.29643	-0.2598	-0.19453	-0.2066	-0.36281	-0.3063	-0.21026	-0.22122	-0.21732	-0.10503	-0.35507	-0.75922
S54	-0.3751	0.54506	-0.33268	-0.35837	-0.32079	-0.22366	-0.2651	-0.38855	-0.41373	-0.22699	-0.18588	-0.17054	-0.1569	-0.29464	-0.27789
S55	0.93163	1.4089	0.23769	0.33741	0.37386	0.40317	0.37353	0.46176	0.51207	0.40013	0.41225	0.43154	0.50428	0.7935	0.58854
S56	0.48977	0.97842	0.58656	0.51864	0.50812	0.54245	0.46528	0.42721	0.48567	0.48301	0.47009	0.47812	0.50182	0.82746	0.65015
S57	0.00504	1.37248	-0.27923	-0.30207	-0.28355	-0.2271	-0.25562	-0.28495	-0.21498	-0.19501	-0.18552	-0.17151	-0.08501	0.27447	0.02593
S58	0.15758	2.59738	-0.7558	-0.82049	-0.73446	-0.67761	-0.67671	-0.64055	-0.78787	-0.57442	-0.5419	-0.5347	-0.44936	-0.06646	-0.04413
S59	0.28282	0.82196	-0.20537	-0.20896	-0.18176	-0.07469	-0.14398	-0.24463	-0.32926	-0.0451	-0.0123	-0.0436	0.05695	0.58432	0.79674
S60	0.08362	0.309	0.61379	0.49964	0.4572	0.55007	0.47923	0.25608	0.24513	0.38459	0.37499	0.42707	0.37549	-0.07993	-0.39168

S61	-1.10284	0.1395	-0.976	-0.95647	-0.90319	-0.83486	-0.85833	-0.97018	-0.86354	-0.84349	-0.76753	-0.78369	-0.37985	-0.04763	-1.19908
S62	-0.53028	-0.13879	-0.35199	-0.28695	-0.24401	-0.13908	-0.14885	-0.25188	-0.30115	-0.04679	0.02672	0.06617	-0.03849	-0.6905	-0.74801
S63	0.29567	1.48137	0.20745	0.06692	-0.05333	-0.12413	-0.12953	-0.11764	-0.01245	-0.24668	-0.27176	-0.3056	-0.35774	-0.43202	-0.39079
S64	1.32867	1.12464	1.01453	1.28039	1.16273	1.02205	1.03045	1.09973	1.13282	1.01806	1.00113	1.05626	1.46585	1.46838	0.38183
S65	0.08932	1.31361	-0.19128	-0.08861	-0.08414	0.00786	0.02382	0.00629	0.25177	0.19222	0.34364	0.42282	0.46617	0.26238	-0.03914
S66	0.22988	1.59755	0.44993	0.42794	0.40848	0.4422	0.41077	0.32805	0.23851	0.39324	0.44248	0.39648	0.4178	1.12697	0.30509
S67	0.42764	0.15016	-0.03562	0.08162	-0.00537	-0.02538	0.01348	0.1367	0.26989	0.07797	0.23315	0.35281	0.99182	4.64098	0.96028
S68	-0.54926	0.1984	-0.1694	-0.1743	-0.16944	-0.0477	-0.07615	-0.1652	-0.20124	-0.01797	0.00798	0.05807	-0.11708	-0.63011	-0.4988
S69	0.26317	0.65605	0.51413	0.62626	0.59038	0.1475	0.34239	0.76758	0.54038	0.05848	0.19393	-0.026	-0.31464	-0.31422	-0.09915
S70	-0.07051	-0.26338	-0.25464	-0.28071	-0.28319	-0.39269	-0.33071	-0.26165	-0.40155	-0.38348	-0.40175	-0.4144	-0.44961	-0.09805	0.2858
S71	0.04274	0.02325	-0.16315	0.04368	0.07104	-0.23714	-0.07654	0.18566	0.03329	-0.20362	-0.2379	-0.32964	-0.41976	-0.39151	-0.07783
S72	0.06174	-0.86188	0.20349	0.45292	0.41694	0.05613	0.23196	0.54174	0.52901	0.04524	-0.02841	-0.01083	-0.11379	-0.31874	-0.15385
S73	-1.73549	-1.05437	-1.02741	-1.05835	-1.03334	-0.83486	-0.90894	-1.13168	-1.27362	-0.84349	-0.76753	-0.80077	-0.92577	-1.60018	-1.61627
S74	0.05189	-0.10615	0.33391	0.24306	0.10589	0.00139	-0.00639	0.0295	-0.11812	-0.15062	-0.21801	-0.24221	-0.36427	-0.23479	0.08119
S75	-0.26837	-1.39051	-0.4169	-0.41956	-0.34161	-0.35283	-0.30781	-0.31973	-0.52595	-0.37282	-0.36625	-0.33964	-0.35544	0.16137	0.11392
S76	1.48199	-0.42091	1.62737	2.59699	2.87539	2.00336	3.23226	2.98271	3.38724	1.25523	0.78446	0.66837	0.61182	0.09633	0.34667
S77	-0.0818	-0.29799	-0.5593	-0.58086	-0.4914	-0.61018	-0.51123	-0.41947	-0.60384	-0.57951	-0.62708	-0.63956	-0.64533	-0.11647	0.21498
S78	-0.32704	-0.13587	-0.5396	-0.39081	-0.41121	-0.7417	-0.62734	-0.42927	-0.31994	-0.80199	-0.76649	-0.78688	-0.87219	-0.64809	-0.32709
S79	0.62331	0.55911	0.45861	0.8491	0.98359	0.55146	0.85068	0.96822	0.97314	0.40369	0.28564	0.19594	0.07205	-0.02779	0.41687
S80	0.66581	-0.4292	0.52926	0.82537	0.70329	0.44427	0.55186	0.71395	1.11752	0.26662	0.16026	0.13636	0.01049	-0.19743	0.20885
S81	-0.38158	-0.84084	-0.04419	-0.12949	-0.11155	-0.06766	-0.11201	-0.1601	-0.21141	-0.09162	-0.08855	-0.10276	-0.17026	-0.41316	-0.15399
S82	-0.78264	-1.06503	-1.02741	-1.05835	-1.00374	-0.83486	-0.90894	-0.69299	-0.66424	-0.84349	-0.76753	-0.80077	-0.87602	-0.54519	-0.33054
S83	-1.41512	-1.55033	1.80697	-0.77933	-0.9377	-0.61234	-0.90894	-0.97327	-0.63502	-0.73224	-0.76753	-0.80077	-0.92577	-1.60072	-0.92678
S84	-2.06569	-1.97509	0.34187	-1.05835	-1.03334	-0.83486	-0.90894	-1.13168	-1.27362	-0.84349	-0.76753	-0.80077	-0.92577	-1.60072	-1.82647
S85	1.586	-0.09531	1.06321	1.78184	1.90467	1.09198	1.57533	2.7828	2.51455	0.79382	0.61717	0.66638	0.5435	0.34429	0.49183
S86	-0.44328	-0.70153	-0.31691	-0.16636	-0.14913	-0.21707	-0.20779	-0.16957	-0.25742	-0.18254	-0.24355	-0.25444	-0.34902	-0.29015	0.20632
S87	-0.11552	-0.0842	-0.49839	-0.61545	-0.66233	-0.80187	-0.74415	-0.56267	-0.58265	-0.80715	-0.76753	-0.79109	-0.69578	-0.23674	-0.13075
S88	0.44601	0.7131	-0.21774	-0.08573	0.00635	0.0311	0.02911	0.05365	-0.0846	0.0655	0.05908	0.07292	0.10403	0.35627	0.37617
S89	-0.58329	-0.55165	-0.31891	-0.19855	-0.19619	-0.14449	-0.18421	-0.2306	-0.30922	-0.09352	-0.15422	-0.2037	-0.28647	-0.48604	-0.14696
S90	-0.92718	-0.70552	3.34583	-0.43084	-0.48435	-0.18625	-0.49883	-0.53005	-0.29185	-0.26083	-0.32463	-0.43366	-0.53946	-0.92012	0.53306
S91	0.67783	-0.319	0.45915	0.74839	0.73929	0.69026	0.64389	0.82031	0.64258	0.67668	0.66722	0.71092	0.66493	0.65722	1.10068
S92	0.03302	0.06433	-0.38691	-0.44509	-0.41432	-0.46933	-0.42581	-0.45317	-0.5231	-0.4962	-0.52623	-0.53718	-0.57813	-0.19524	-0.02147
S93	-0.52068	0.48921	-0.50569	-0.54287	-0.5015	-0.56812	-0.50516	-0.38554	-0.41458	-0.52583	-0.56785	-0.56988	-0.63664	-0.54656	-0.4289
S94	-0.68301	-1.23634	-1.02114	-0.7691	-0.72157	-0.83486	-0.78741	-0.55794	-0.29239	-0.84349	-0.76753	-0.80077	-0.9062	-0.22246	0.19926
S95	-0.47313	-0.41349	-0.7714	-0.77825	-0.75723	-0.7792	-0.75143	-0.73627	-0.84351	-0.73958	-0.76753	-0.7657	-0.77279	-0.36469	-0.0386
S96	0.63121	0.44797	0.29184	0.54469	0.52872	0.40724	0.37271	0.44089	0.40217	0.3919	0.35709	0.30253	0.25514	0.45623	1.04659
S97	0.37437	-0.26329	-0.25583	-0.19223	-0.10985	-0.12715	-0.13769	-0.0849	-0.36253	-0.11149	-0.0671	-0.04606	-0.09998	0.44932	0.71005
S98	-0.66625	-0.96344	-0.99573	-0.81071	-0.88982	-0.83486	-0.90894	-0.89849	-0.90356	-0.84349	-0.76753	-0.80077	-0.92577	-0.52738	-0.38394
S99	0.3491	0.24123	-0.78503	-0.76633	-0.69267	-0.64197	-0.64731	-0.69951	-0.93067	-0.57916	-0.54774	-0.53008	-0.46282	0.61219	0.63772
S100	-0.25743	-0.53348	-0.21455	-0.17859	-0.2402	-0.31423	-0.21877	-0.14239	-0.24045	-0.2449	-0.34438	-0.37227	-0.40838	-0.18902	0.07506
S101	0.56625	-0.07294	-0.08555	0.05908	-0.01786	-0.10725	-0.01353	0.17673	0.17937	0.00029	0.00517	0.06251	0.08055	0.27082	0.83691
S102	-0.35751	-0.5919	-0.73344	-0.74746	-0.69677	-0.73215	-0.72268	-0.69931	-0.761	-0.70757	-0.75274	-0.78439	-0.7689	-0.39339	-0.20783
S103	-0.45575	-0.84149	-0.19344	-0.02606	-0.11073	-0.15632	-0.09998	-0.09923	0.16472	-0.18389	-0.2951	-0.29518	-0.38008	-0.73718	-0.68334
S104	-0.07946	0.42217	-0.1786	-0.05271	-0.10185	-0.12276	-0.04502	0.01805	-0.14107	-0.06995	-0.17801	-0.22827	-0.18681	0.19941	0.3588

S105	0.22502	-0.46252	0.17581	0.31361	0.24744	0.11971	0.11678	0.31551	0.34438	0.13883	0.13583	0.12701	0.04775	0.00775	0.21554
S106	0.39903	1.00471	0.08734	0.28254	0.23895	-0.08185	0.00819	0.23606	0.28813	-0.12508	-0.09892	-0.16591	-0.24966	-0.08772	0.26748
S107	-0.82528	-0.87504	-0.83092	-0.70323	-0.74133	-0.83486	-0.79838	-0.58078	-0.31633	-0.77752	-0.76753	-0.80077	-0.90342	-0.7628	-0.30274
S108	0.14115	-0.03794	-0.46882	-0.37699	-0.25665	-0.4512	-0.49173	-0.46538	-0.54665	-0.48628	-0.49578	-0.47182	-0.45179	0.61588	1.0866
S109	0.26667	0.74096	-0.12501	0.07282	0.02851	-0.16995	-0.12538	0.12928	0.0893	-0.06717	-0.14227	-0.14018	-0.16049	-0.16009	-0.09877

Sample No.	integrated bins														
	4.60 .. 4.58	4.58 .. 4.56	4.56 .. 4.54	4.54 .. 4.52	4.52 .. 4.50	4.50 .. 4.48	4.48 .. 4.46	4.46 .. 4.44	4.44 .. 4.42	4.42 .. 4.40	4.40 .. 4.38	4.38 .. 4.36	4.36 .. 4.34	4.34 .. 4.32	4.32 .. 4.30
S1	1.29064	1.67004	0.83422	0.88355	0.79423	0.49823	0.54129	0.56535	0.68403	0.55835	0.39488	0.35156	0.19569	0.02254	-0.01429
S2	-1.0887	-0.86607	-0.5698	-0.6228	-0.62639	-0.63679	-0.67578	-0.77119	-0.87893	-0.84917	-0.91591	-0.92371	-0.80514	-0.82893	-0.90296
S3	-0.89799	-0.69243	-0.5698	-0.20345	-0.31795	-0.63679	-0.67578	-0.77119	-0.64324	-0.70004	-0.7546	-0.61386	-0.80514	-0.82893	-0.90296
S4	-0.82802	-0.85981	-0.5698	-0.6228	-0.62639	-0.63679	-0.67578	-0.77119	-0.87893	-0.83238	-0.65666	-0.48883	-0.33271	-0.22296	0.02292
S5	1.98006	1.47882	-0.5698	-0.6228	-0.62639	-0.63679	-0.67578	-0.77119	-0.87893	-0.84917	-0.91591	-0.94044	-0.80514	-0.82893	-0.90296
S6	0.30117	-0.34501	-0.5698	-0.6228	-0.62639	-0.63679	-0.67578	-0.76636	-0.61651	-0.62116	-0.59343	-0.56723	-0.66336	-0.67414	-0.58643
S7	0.54685	-0.85308	-0.5698	-0.6228	-0.62639	-0.63679	-0.67578	-0.77119	-0.87893	-0.84917	-0.91591	-0.94044	-0.80514	-0.82893	-0.90296
S8	-0.01029	0.13604	-0.05229	0.05948	-0.00234	-0.25047	-0.32203	-0.28934	-0.284	-0.31433	-0.32152	-0.31469	-0.45308	-0.57697	-0.57621
S9	-0.19258	0.7457	-0.5698	-0.6228	-0.62639	-0.63679	-0.67578	-0.77119	-0.78505	-0.79177	-0.7627	-0.47901	-0.39892	-0.29462	-0.16192
S10	-1.53502	-0.99981	-0.26139	-0.20134	-0.17729	-0.06799	0.16632	0.11621	0.29247	0.29214	0.33593	0.37939	0.49019	0.58352	0.81884
S11	-1.42852	-1.39217	-0.5698	-0.6228	-0.62639	-0.63679	-0.67578	-0.77119	-0.87893	-0.84917	-0.91591	-0.75508	-0.53482	-0.28909	-0.01873
S12	1.37481	0.44145	0.06565	-0.12646	-0.1259	-0.10226	-0.17257	-0.11056	-0.13365	-0.19747	0.04419	-0.05901	-0.29488	-0.33941	-0.33398
S13	1.68875	1.47192	1.03071	1.00591	1.00644	1.03131	1.08776	1.1388	1.1141	0.96847	1.06802	0.77944	0.63167	0.56459	0.54919
S14	1.75715	-0.23306	-0.29933	-0.27252	-0.28999	-0.31432	-0.17155	-0.07448	-0.08263	-0.10575	-0.16531	-0.22499	-0.1131	-0.18119	-0.14789
S15	0.74518	1.69985	3.82054	3.99643	3.95761	3.9318	3.68748	3.47595	3.52996	3.53881	3.48849	3.16103	3.66344	3.77974	4.02702
S16	0.71517	0.61987	-0.5698	-0.47221	-0.48886	-0.63679	-0.67578	-0.71659	-0.7048	-0.63664	-0.62107	-0.53412	-0.62052	-0.55797	-0.43659
S17	-1.52993	-0.89586	-0.5698	-0.6228	-0.62639	-0.63679	-0.67578	-0.77119	-0.87893	-0.84917	-0.91591	-0.94044	-0.80514	-0.82893	-0.90296
S18	-1.24348	-0.84906	-0.5698	-0.27939	-0.27528	-0.63679	-0.67578	-0.77119	-0.63078	-0.57968	-0.52614	-0.27808	-0.3935	-0.4187	-0.21462
S19	-2.32354	-1.57034	-0.5698	-0.6228	-0.62639	-0.63679	-0.67578	-0.77119	-0.87893	-0.84917	-0.91591	-0.94044	-0.80514	-0.82893	-0.90296
S20	-0.64681	0.02434	0.73892	1.17582	1.14464	0.39534	0.40528	0.31416	0.40089	0.45516	0.41096	0.41664	0.39985	0.23849	0.41665
S21	0.30528	0.7337	1.32899	1.14456	1.13349	1.33151	1.22348	1.07612	1.04829	1.06601	1.00065	0.94746	0.99223	0.96484	0.96054
S22	0.16733	-0.57706	-0.5698	-0.6228	-0.62639	-0.63679	-0.67578	-0.77119	-0.87893	-0.84917	-0.91591	-0.94044	-0.80514	-0.82893	-0.90296
S23	1.25243	-0.57137	-0.26371	-0.33534	-0.44169	-0.5725	-0.59934	-0.66714	-0.61321	-0.57051	-0.64559	-0.47134	-0.54019	-0.62118	-0.64169
S24	1.38876	0.16699	-0.5698	-0.50208	-0.53158	-0.63679	-0.56921	-0.36505	-0.27012	-0.3755	-0.38343	-0.49767	-0.7444	-0.79185	-0.82012
S25	0.54449	1.40832	1.75362	2.23218	2.34241	1.87043	1.94381	2.01364	2.09747	2.16262	2.03337	1.89627	2.05373	1.95848	2.16022
S26	-0.03442	0.74095	1.71189	2.00571	2.07462	1.84059	1.85438	1.86392	1.82202	1.89189	1.81905	1.75377	1.62749	1.70814	1.86885
S27	-0.25776	0.49819	0.34	0.68386	0.70643	0.10712	0.07903	0.05829	-0.02316	0.00746	0.03967	0.05724	0.03166	-0.02554	0.06523
S28	-0.61784	0.209	0.39207	0.70754	0.61778	-0.0501	0.00205	-0.03823	-0.01462	-0.08135	-0.19984	-0.1958	-0.3372	-0.55117	-0.48161
S29	-0.39921	0.31338	-0.00707	0.28995	0.25299	-0.33302	-0.19163	0.11765	0.29246	0.01152	-0.26206	-0.25495	-0.51769	-0.77032	-0.78679
S30	-0.43636	0.29322	-0.5698	-0.6228	-0.62639	-0.63679	-0.65308	-0.5279	-0.43177	-0.35903	-0.3435	-0.14386	-0.16953	-0.11416	0.06916
S31	1.177	1.89631	-0.27075	-0.1109	-0.10729	-0.28344	-0.25711	-0.17585	-0.05996	0.00768	-0.05281	-0.00514	-0.09559	-0.18008	-0.16549
S32	-0.49999	0.23445	-0.5698	-0.26312	-0.40334	-0.63679	-0.67578	-0.41138	-0.04261	-0.08013	-0.22063	-0.11527	-0.43274	-0.45164	-0.21013
S33	1.32526	0.94442	-0.04484	-0.07244	-0.02318	0.01615	-0.08895	-0.06309	-0.06291	-0.06327	-0.09353	-0.10632	-0.15319	-0.24572	-0.26859
S34	-0.03806	-0.21835	-0.5698	-0.37591	-0.48406	-0.63679	-0.67578	-0.76299	-0.75034	-0.71582	-0.5904	-0.58772	-0.74651	-0.68669	-0.58248
S35	-0.78313	0.0145	-0.5698	-0.6228	-0.62639	-0.63679	-0.67578	-0.77119	-0.87893	-0.84917	-0.82821	-0.51531	-0.72759	-0.74275	-0.67875
S36	-0.98052	-0.69605	-0.5698	-0.6228	-0.62639	-0.63679	-0.67578	-0.77119	-0.87893	-0.84917	-0.91591	-0.88468	-0.80514	-0.82893	-0.90296

S37	0.58569	0.00538	0.18621	0.20949	0.2316	0.29845	0.32143	0.42336	0.4849	0.56108	0.53261	0.38476	0.56732	0.62062	0.6936
S38	0.16103	0.42977	1.61034	1.54772	1.5739	1.58642	1.61496	1.56651	1.60493	1.66869	1.57185	1.37476	1.55029	1.51109	1.68047
S39	2.65622	0.40116	-0.42416	-0.55463	-0.56979	-0.57706	-0.59873	-0.62021	-0.64769	-0.66423	-0.7241	-0.78703	-0.77266	-0.77322	-0.86354
S40	-1.79211	-1.3718	-0.5698	-0.6228	-0.62639	-0.63679	-0.67578	-0.77119	-0.87893	-0.84917	-0.91591	-0.94044	-0.80514	-0.82893	-0.58855
S41	-1.61225	-1.10413	-0.5698	-0.6228	-0.62639	-0.63679	-0.67578	-0.77119	-0.65243	-0.57843	-0.46143	-0.28349	-0.38575	-0.44185	-0.21983
S42	-0.81745	-0.28036	-0.1253	0.12421	0.15424	-0.07854	-0.02583	0.01231	0.23173	0.28375	0.32686	0.43316	0.39051	0.3046	0.54336
S43	1.08044	0.13615	-0.5698	-0.6228	-0.62639	-0.63679	-0.56803	-0.43319	-0.53196	-0.70272	-0.79776	-0.80035	-0.80514	-0.82893	-0.90296
S44	1.19105	0.42579	-0.29059	-0.44142	-0.4302	-0.27034	-0.17309	-0.15846	-0.27469	-0.35298	-0.33139	-0.48033	-0.59134	-0.56151	-0.58579
S45	1.22431	0.31304	0.42078	0.37357	0.40342	0.43633	0.45272	0.46726	0.38453	0.39761	0.40528	0.23427	0.30694	0.41124	0.41052
S46	-1.11192	-0.86528	-0.5698	-0.6228	-0.62639	-0.63679	-0.67578	-0.77119	-0.75315	-0.67196	-0.57737	-0.46689	-0.60629	-0.61651	-0.45171
S47	-0.7662	-0.19037	-0.5698	-0.49983	-0.51623	-0.63679	-0.6528	-0.54374	-0.39707	-0.32485	-0.27884	-0.1381	-0.289	-0.31852	-0.17095
S48	-0.7125	-0.16307	-0.19759	0.03884	-0.00492	-0.34218	-0.29346	-0.29073	-0.18676	-0.16419	-0.11149	-0.02051	-0.30947	-0.27722	-0.13248
S49	0.3732	1.01302	1.52442	1.69194	1.69604	1.41907	1.38664	1.38482	1.53748	1.54982	1.37117	1.24647	1.23933	1.08727	1.17528
S50	-0.7023	-0.74611	-0.5698	-0.6228	-0.62639	-0.63679	-0.67578	-0.77119	-0.87893	-0.84917	-0.91591	-0.94044	-0.80514	-0.82893	-0.90296
S51	1.09648	1.39703	-0.52272	-0.43245	-0.16519	-0.10199	-0.36701	-0.23257	-0.15443	-0.08791	-0.01993	0.16242	0.54491	0.17584	0.13095
S52	0.08549	0.83012	-0.5698	-0.6228	-0.62639	-0.63679	-0.67578	-0.77119	-0.87893	-0.84917	-0.91591	-0.94044	-0.80514	-0.82893	-0.90296
S53	-0.61047	-0.3229	-0.33075	-0.00927	0.03126	-0.45146	-0.35081	-0.27618	-0.16934	-0.12911	-0.1244	-0.02668	-0.18312	-0.21212	-0.10086
S54	0.56736	0.31689	-0.22536	-0.1372	-0.12444	-0.33739	-0.41076	-0.31474	-0.28609	-0.22717	-0.27306	-0.25127	-0.36716	-0.41128	-0.35032
S55	0.64735	0.6809	-0.5698	-0.6228	-0.62639	-0.63679	-0.67578	-0.77119	-0.86654	-0.84917	-0.91591	-0.80804	-0.80514	-0.82893	-0.76114
S56	0.41448	0.70827	-0.5698	-0.6228	-0.62639	-0.63679	-0.65254	-0.39829	-0.2978	-0.28937	-0.25892	-0.14659	-0.14247	-0.15159	0.02828
S57	1.44821	1.49688	0.05262	0.2449	0.24934	0.00774	0.01515	0.16019	0.24138	0.1485	0.07063	0.07275	-0.05158	-0.06923	-0.03733
S58	2.40878	1.393	-0.54553	-0.471	-0.47481	-0.63465	-0.67578	-0.69118	-0.73351	-0.77872	-0.8112	-0.79801	-0.80514	-0.82893	-0.90296
S59	0.41323	-0.17972	-0.5698	-0.6228	-0.62639	-0.63679	-0.67578	-0.77119	-0.87893	-0.84917	-0.91591	-0.87132	-0.80514	-0.82893	-0.85578
S60	0.63616	1.51851	0.98421	1.49536	1.6175	1.41903	1.5813	1.83131	2.00198	2.06401	2.07097	2.0084	2.09033	2.30145	2.60494
S61	0.90366	1.39646	0.79752	1.01889	0.92877	0.55179	0.49551	0.63497	0.81653	0.66891	0.38515	0.30126	0.21373	0.07499	-0.01951
S62	-0.28883	0.48946	-0.5698	-0.6228	-0.62639	-0.63679	-0.67578	-0.77119	-0.87893	-0.84917	-0.91591	-0.87174	-0.80514	-0.82893	-0.85586
S63	2.06027	3.08206	1.55927	1.87509	2.06863	1.85602	1.92574	2.10835	2.17695	2.13443	1.85816	1.72054	1.85391	1.90103	2.04528
S64	-0.29968	0.19217	-0.5698	-0.6228	-0.62639	-0.63679	-0.67578	-0.77119	-0.87893	-0.84917	-0.91591	-0.94044	-0.80514	-0.82893	-0.90296
S65	1.54722	2.60338	0.10578	-0.03242	-0.10541	-0.03909	-0.14021	-0.09267	-0.22053	-0.21751	-0.31276	-0.11558	-0.24956	-0.1244	-0.02679
S66	1.00638	1.60683	-0.5698	-0.6228	-0.62639	-0.63679	-0.66248	-0.52044	-0.41824	-0.40293	-0.39395	-0.25479	-0.29875	-0.18973	-0.02509
S67	0.39959	1.2491	0.15697	0.15523	0.1928	0.33845	0.38403	0.46248	0.16808	0.10927	0.00039	0.0572	0.00296	0.12061	0.11875
S68	0.00705	0.53885	-0.5698	-0.6228	-0.62639	-0.63679	-0.64041	-0.5818	-0.43569	-0.29162	-0.1663	-0.04027	-0.28981	-0.02943	0.3044
S69	0.68138	0.8058	0.56695	0.51552	0.59083	0.9344	0.81011	0.93665	0.85784	1.00998	1.26135	1.14102	1.57737	1.55797	1.3479
S70	-0.23065	-0.66142	0.02992	-0.08166	-0.13539	-0.015	-0.09219	-0.09805	-0.10146	-0.08808	-0.01189	-0.08487	-0.09937	-0.0759	-0.20181
S71	-0.26787	-0.52064	-0.5698	-0.6228	-0.62639	-0.6006	-0.60331	-0.54637	-0.50562	-0.47485	-0.26737	-0.27267	-0.47047	-0.39905	-0.48577
S72	-1.05321	-1.05798	-0.5698	-0.6228	-0.62639	-0.63679	-0.67578	-0.77119	-0.50815	-0.6081	-0.54877	-0.551	-0.80514	-0.79738	-0.88579
S73	-0.92491	-0.78854	-0.5698	-0.6228	-0.62639	-0.63679	-0.67578	-0.77119	-0.87893	-0.84917	-0.91591	-0.94044	-0.80514	-0.82893	-0.90296
S74	0.097	-0.10786	0.98504	0.98161	1.06167	1.36926	1.4838	1.43297	1.46969	1.54953	1.57428	1.36209	1.6573	1.80788	1.87347
S75	-1.41446	-1.36834	-0.5698	-0.6228	-0.62639	-0.56184	-0.52843	-0.57755	-0.59925	-0.62887	-0.61949	-0.64263	-0.64106	-0.57162	-0.61268
S76	-0.98004	-0.76793	0.83236	0.07669	-0.07878	0.59143	0.1571	0.33381	0.73821	0.83868	3.15353	4.92373	3.23161	2.74797	0.96534
S77	-0.21552	-0.62769	-0.04519	-0.14252	-0.12741	0.03927	-0.0456	-0.07279	-0.08899	-0.14	-0.10545	-0.14112	-0.20095	-0.17507	-0.25718
S78	0.36079	0.29098	1.17963	1.04193	1.11339	1.55483	1.60964	1.63304	1.3178	1.20241	1.10877	0.90477	1.12717	1.01609	0.80012
S79	-0.05809	-0.26167	-0.5698	-0.6228	-0.62639	-0.522	-0.58577	-0.55121	-0.41315	-0.35832	0.11543	0.63482	0.36003	0.34801	0.04156
S80	-0.68387	-0.61148	-0.44339	-0.52719	-0.46517	0.09455	0.294	0.37932	0.48464	0.39633	0.44371	0.41905	0.44447	0.45452	0.42417

S81	-1.21739	-1.28199	-0.5698	-0.6228	-0.62639	-0.63679	-0.67578	-0.64763	-0.68578	-0.68464	-0.67958	-0.66903	-0.59419	-0.44461	-0.44512
S82	-1.18264	-1.4821	-0.5698	-0.6228	-0.62639	-0.63679	-0.67578	-0.77119	-0.87893	-0.84917	-0.91591	-0.94044	-0.80514	-0.82893	-0.90296
S83	0.26968	0.89685	2.30402	2.10461	2.10014	2.2572	2.30611	2.20948	2.24196	2.4512	2.14815	2.13659	2.42254	2.27555	2.52775
S84	3.13272	3.6546	6.95544	6.29614	6.09001	6.1808	5.9369	5.51202	5.26367	5.333	4.60772	4.2778	4.60357	4.47556	4.41101
S85	-0.47103	-0.26944	-0.46487	-0.6228	-0.62639	0.08721	0.68382	0.97863	0.85741	0.81666	1.34103	1.17163	1.45047	1.80431	0.4268
S86	-0.85501	-1.08816	-0.56935	-0.6228	-0.59948	-0.42778	-0.37414	-0.36241	-0.22051	-0.22736	-0.10882	-0.11179	-0.26194	-0.24111	-0.1775
S87	0.72381	0.53078	1.29913	1.23212	1.27945	1.59833	1.64002	1.76171	1.475	1.46496	1.28985	0.95536	1.17195	1.26804	1.24977
S88	-0.09718	-0.71662	-0.5698	-0.6228	-0.62639	-0.63679	-0.67578	-0.77119	-0.87893	-0.84917	-0.91591	-0.94044	-0.80514	-0.82893	-0.90296
S89	-0.76965	-1.01196	-0.5698	-0.6228	-0.62639	-0.54619	-0.49248	-0.46883	-0.3656	-0.22618	-0.11592	-0.1105	-0.26041	-0.19759	0.0014
S90	0.33222	0.62532	-0.24596	-0.38786	-0.44872	-0.39166	-0.26821	-0.1987	-0.27433	-0.02412	-0.27047	-0.16544	-0.10562	-0.26183	-0.23463
S91	-1.56443	-1.57034	-0.5698	-0.6228	-0.62639	-0.63679	-0.67578	-0.77119	-0.87893	-0.84917	-0.91591	-0.94044	-0.80514	-0.82893	-0.90296
S92	0.06479	-0.28573	0.01512	-0.01618	0.03628	0.27328	0.31752	0.33097	0.27805	0.23054	0.21525	0.02575	0.15778	0.21493	0.22236
S93	0.47279	0.17683	-0.20877	-0.27975	-0.26021	-0.04457	-0.10813	-0.07243	-0.14686	-0.16122	-0.09467	-0.21829	-0.13544	-0.02055	-0.14033
S94	-0.64006	-0.76394	0.14155	-0.04865	-0.11361	0.28753	0.32634	0.1314	-0.02127	-0.2212	-0.31235	-0.37589	-0.58508	-0.63668	-0.77656
S95	-0.17157	-0.63854	0.06338	-0.00643	-0.01984	0.11305	0.10474	0.07882	0.02373	-0.00085	-0.1004	-0.18761	-0.21067	-0.10676	-0.12565
S96	-0.27897	-0.70381	-0.5698	-0.6228	-0.62639	-0.63679	-0.67578	-0.72436	-0.55196	-0.49746	-0.4838	-0.4028	-0.4302	-0.18352	-0.14102
S97	-0.85392	-1.40616	-0.5698	-0.6228	-0.62639	-0.63679	-0.67578	-0.77119	-0.87893	-0.84917	-0.91591	-0.94044	-0.80514	-0.82893	-0.90296
S98	-0.1556	-0.36923	0.78034	0.71948	0.73731	0.97116	0.91603	0.9384	0.85122	0.5981	0.38508	0.0878	0.15408	0.14362	0.00585
S99	0.16428	-0.88228	-0.5698	-0.6228	-0.62639	-0.63679	-0.67578	-0.77119	-0.87893	-0.84917	-0.91591	-0.94044	-0.80514	-0.82893	-0.90296
S100	-0.54811	-0.81361	-0.24649	-0.30954	-0.25192	-0.00371	0.0968	0.14542	0.18292	0.13539	0.27563	0.15841	0.11934	0.23871	0.27489
S101	-0.33377	-0.64483	-0.5698	-0.6228	-0.62639	-0.63679	-0.67578	-0.6923	-0.61301	-0.62989	-0.56538	-0.55486	-0.60577	-0.5339	-0.49783
S102	-0.35042	-0.75662	-0.02508	-0.09606	-0.06719	0.04371	0.00405	0.16079	0.06533	0.03179	-0.08072	-0.22904	-0.2043	-0.18323	-0.2112
S103	-0.69852	-0.17921	0.22002	0.26142	0.31625	0.58383	0.77587	0.79102	0.93774	0.98262	1.07893	0.80367	0.88529	1.1358	1.3643
S104	0.15608	-0.3745	-0.50111	-0.54069	-0.5041	-0.31612	-0.26834	-0.11269	-0.08278	0.02683	0.23961	0.10526	-0.05931	0.12075	0.15294
S105	-1.06501	-1.19625	-0.5698	-0.6228	-0.62639	-0.63679	-0.67578	-0.77119	-0.72483	-0.84917	-0.91591	-0.94044	-0.80514	-0.82893	-0.90296
S106	0.89726	0.34939	0.07817	0.05248	0.06714	0.56367	0.64648	0.80907	0.57326	0.47115	0.39902	0.36708	0.5691	0.56412	0.36155
S107	-0.32897	-0.53237	-0.01731	-0.24819	-0.3712	-0.10117	0.00738	-0.18009	-0.1154	-0.20382	-0.28967	-0.40582	-0.71772	-0.77414	-0.77277
S108	0.22719	-0.89943	-0.17669	-0.23129	-0.21848	0.01449	-0.0905	-0.11924	-0.07358	-0.22226	-0.25093	-0.41117	-0.32226	-0.20641	-0.40483
S109	0.52027	0.17249	-0.5698	-0.6228	-0.62639	-0.40722	-0.1637	-0.1562	-0.1615	-0.1616	-0.32756	-0.3749	-0.31319	-0.27275	-0.38548

Sample No.	integrated bins														
	4.30 .. 4.28	4.28 .. 4.26	4.26 .. 4.24	4.24 .. 4.22	4.22 .. 4.20	4.20 .. 4.18	4.18 .. 4.16	4.16 .. 4.14	4.14 .. 4.12	4.12 .. 4.10	4.10 .. 4.08	4.08 .. 4.06	4.06 .. 4.04	4.04 .. 4.02	4.02 .. 4.00
S1	0.03552	-0.4959	-0.56255	-0.10321	0.12853	0.08938	-0.91135	-0.91771	0.15538	0.13115	-0.28029	-0.45121	0.78975	1.19148	0.86767
S2	-0.64923	-0.51868	-0.66126	-0.64097	-0.24186	-0.0792	0.12121	0.01146	-0.27107	-0.12681	0.34791	0.56515	1.29757	1.29082	0.16824
S3	-0.99006	-0.84193	-0.71092	-0.65336	-0.86685	-0.98284	1.14697	1.22402	-0.24264	-0.58342	-0.63553	-0.16687	0.68824	1.27195	0.95669
S4	0.00798	-0.56447	-0.73003	-0.40814	-0.05047	0.26429	1.22789	1.16213	0.11429	0.26346	-0.20253	-0.55203	-0.62413	-0.37296	0.183
S5	-1.18925	-1.13194	-1.01972	-1.04532	-1.13302	-0.82272	-1.40378	-1.3727	-1.00239	-1.24607	-1.05349	-1.26754	-0.77732	-0.62048	-0.95683
S6	-0.51976	-0.65269	-0.37455	0.00498	-0.69309	-0.62947	0.0218	-0.033	-0.48924	-0.58269	-0.59094	-0.92706	-0.93869	-0.17857	0.42097
S7	-1.18925	-1.11894	-1.20861	-1.23302	-1.24441	-1.35309	-1.00939	-0.95985	-0.9456	-1.35074	-1.44673	-1.44387	-1.35841	-1.45175	-1.36996
S8	-0.57334	-0.71229	-0.5225	-0.1413	-0.68793	-1.06531	1.37615	1.4581	-0.30857	-1.05403	-1.00657	-0.71005	-0.18786	0.24692	0.28222
S9	0.40806	0.09214	-0.1921	-0.26845	0.8366	0.76027	-0.79758	-0.90861	-0.07468	0.99626	1.59413	3.86228	3.05241	2.04324	-0.77859
S10	0.91071	0.09241	-0.02853	0.25693	0.69057	0.71957	1.7565	1.52273	-0.11059	0.24287	0.74551	0.60657	-0.00489	-0.16642	-0.23259
S11	0.07018	-0.36633	-0.55506	-0.20214	0.45004	0.70578	-0.39994	-0.47527	0.18937	1.18916	0.6773	0.22058	-0.00727	-0.04424	-0.75053
S12	-0.35563	-0.4477	-0.30021	-0.0831	-0.64177	-0.77631	-1.31469	-1.30457	-0.8435	-1.08954	-1.12521	-1.23569	-0.59835	-0.22998	-0.17198

S13	0.68334	0.56881	0.27168	-0.06104	0.1953	0.46997	-0.46755	-0.52454	0.10363	0.16257	0.51537	1.57866	1.53794	0.30204	-1.24034
S14	-0.20664	-0.69684	-0.76257	-0.53717	-0.64691	-0.65889	-1.15663	-1.10087	-0.40566	-0.3297	-0.80193	-1.34261	-1.20534	-1.31102	-1.4046
S15	4.01665	0.92765	0.34736	1.26411	2.80279	2.73793	0.23933	0.06111	1.23948	3.27483	3.07933	3.17451	1.7945	0.32925	-0.74049
S16	-0.43906	-0.56605	-0.76237	-0.73021	-0.47948	-0.71465	-0.0543	-0.06406	-0.57119	-0.76513	-0.7576	-0.64496	-0.2406	0.53775	-0.34457
S17	-0.92831	-0.6872	-0.64209	-0.59173	-0.60787	-0.71055	1.56063	1.5356	-0.45122	-0.86807	-0.50994	0.43815	1.47283	1.55476	1.26097
S18	0.01	-0.36839	-0.45388	-0.24288	0.10831	0.27884	0.9851	0.9516	-0.05757	0.1296	0.54253	1.39976	1.60602	1.62629	0.42271
S19	-1.18925	-1.165	-0.77231	-0.27141	-1.03502	-0.51872	-1.09511	1.60518	7.63522	-0.45763	-1.26225	-0.20561	0.09708	1.91032	0.80626
S20	0.37231	-0.30019	-0.36029	-0.08104	-0.12618	-0.08911	1.41433	1.65909	0.43533	-0.18338	-0.18643	0.26525	0.86195	1.08556	0.71509
S21	1.09641	0.61461	0.76554	0.9785	0.57207	0.70859	-0.86541	-0.93431	-0.10596	0.98326	1.35545	2.14269	1.9081	1.34153	-0.72628
S22	-1.00604	-0.96761	-1.06503	-0.99934	-1.01855	-1.11112	-1.14876	-1.07057	-0.69788	-0.82499	-1.14659	-1.4721	-0.92752	-0.8899	-0.03515
S23	-0.73956	-0.90722	-0.99299	-0.81835	-0.97263	-1.1176	-0.42004	0.4109	-0.45867	-0.81813	-1.40482	-1.20799	-0.86908	-0.16402	0.39001
S24	-0.85391	-0.89885	-0.83711	-0.64241	-1.05282	-1.07689	-0.68847	-0.62292	-0.56917	-0.91479	-1.0905	-1.39385	-0.60058	0.02884	0.46566
S25	2.03932	0.25599	-0.03051	0.59319	1.25826	1.29992	0.66939	0.58158	0.74879	1.70341	1.11483	1.21052	1.90712	1.27977	0.24011
S26	1.70467	0.01742	-0.07748	0.58193	0.48232	0.5212	1.34269	1.3395	0.17598	0.47375	-0.08645	-0.24848	0.32375	1.53377	1.08825
S27	-0.00785	-0.44632	-0.58012	-0.38741	-0.3558	-0.69809	1.79573	1.85573	-0.10661	-0.50076	-0.41186	-0.14025	0.80045	0.25138	0.44246
S28	-0.49361	-0.69631	-0.69203	-0.53875	-0.73664	-0.9792	1.57564	1.80313	0.1308	-0.56615	-0.65251	-0.26454	0.63913	0.37684	0.56867
S29	-0.65927	-0.77489	-0.82755	-0.66984	-0.52211	-0.7236	1.46698	1.58425	0.24995	-0.27303	-0.36639	-0.16765	0.59322	0.75123	0.65113
S30	0.17549	-0.14527	-0.08546	0.08569	-0.09301	0.1315	1.46645	1.31132	-0.24396	-0.21207	-0.09912	-0.18887	-0.20223	0.35703	0.24528
S31	-0.15529	-0.59877	-0.63732	0.1974	0.34926	-0.21858	-0.93869	-0.84277	0.73759	0.45337	-0.05404	-0.03294	1.38607	0.84813	-0.30345
S32	0.05674	-0.30275	-0.04083	0.56604	0.72884	0.89078	0.1258	0.0322	0.21623	0.7266	0.6132	0.68974	1.06085	2.52209	1.23242
S33	-0.37308	-0.37106	-0.2099	-0.01219	-0.45051	-0.71315	-0.81766	-0.74343	-0.30634	-0.68405	-0.62779	-0.67429	0.09424	0.04732	-0.72973
S34	-0.60037	-0.84334	-0.99614	-0.86221	-0.89654	-1.01725	1.74199	1.69241	-0.50669	-0.94245	-1.09591	-0.99715	-0.30641	0.08149	0.41771
S35	-0.5493	-0.6125	-0.61164	-0.38778	-0.4214	-0.42439	0.89414	0.925	-0.08933	-0.17258	-0.16551	0.08488	1.10193	1.88036	1.0022
S36	-0.9794	-0.91148	-0.94611	-0.80855	-0.7849	-0.80745	1.14238	1.15781	-0.18257	-0.43403	-0.51841	-0.19572	0.59268	1.23806	0.77605
S37	0.5349	-0.38145	-0.51193	-0.12146	-0.08494	0.03299	-0.52393	-0.54707	-0.02827	0.18537	-0.18982	-1.06028	-0.79383	-0.89974	-0.08427
S38	1.40984	-0.06369	-0.09072	0.66725	0.53284	0.55525	1.84092	1.78646	0.4753	0.70169	0.08725	-0.40649	-0.4566	0.16277	0.63675
S39	-1.03027	-0.92332	-0.80791	-0.62199	-1.28885	-1.53209	-1.54836	-1.41102	-0.93245	-1.52655	-1.72123	-1.63989	-0.99912	-1.01503	-1.37788
S40	-0.27157	-0.50644	-0.56426	0.03924	0.63712	0.74908	1.15474	1.02664	0.45516	1.00667	0.78616	1.08043	1.09961	0.68245	0.29908
S41	-0.04332	-0.51747	-0.59175	-0.35616	-0.17278	-0.27081	2.67302	2.54374	-0.19	-0.23672	-0.15293	0.48969	0.77396	0.47582	0.91797
S42	0.61566	-0.2115	-0.38871	-0.00931	0.55959	0.25325	1.78472	1.71647	0.30626	0.41789	0.68201	1.39255	1.12459	0.90928	0.42308
S43	-0.97243	-0.14582	-0.53237	-1.28887	-0.82303	-0.713	-1.31617	-1.40102	-0.92479	-1.02052	-0.67263	-1.26866	-1.16536	-1.44218	-1.95454
S44	-0.68716	-0.25015	-0.43563	-0.83618	-0.78144	-0.79483	-1.36688	-1.36894	-0.60653	-0.74551	-0.77347	-1.26501	-0.9727	-1.36468	-1.56332
S45	0.16211	-0.57283	-1.02094	-0.91583	-0.50943	-0.6864	-1.34435	-1.27578	-0.48484	-0.43119	-0.84962	-1.02822	-0.95675	-1.34435	-1.52303
S46	-0.3297	-0.63271	-0.72433	-0.48077	-0.41718	-0.60318	1.96171	1.91626	-0.26487	-0.55027	-0.50835	0.05961	0.65012	0.40178	0.5746
S47	-0.094	-0.51618	-0.52077	0.26451	0.53365	-0.19479	1.11641	1.19314	0.3754	-0.03178	-0.25823	0.50156	1.44296	1.33006	0.91354
S48	-0.05678	-0.49341	-0.56021	-0.21821	-0.13456	-0.48221	1.95594	1.95346	-0.05334	-0.30774	-0.3313	0.21515	1.06485	0.87568	1.04694
S49	1.0764	-0.15847	-0.28995	0.60929	1.0102	0.44631	0.43184	0.54816	0.78759	0.66913	0.03312	0.551	1.61577	1.68201	0.7882
S50	-1.18925	-1.13861	-0.99098	-0.83757	-0.75043	-0.35657	-1.14032	-1.09784	-0.27794	0.47999	0.06303	-0.37732	0.31447	0.0342	1.03098
S51	0.27193	-0.19473	-0.28845	0.02762	0.40517	0.30124	-0.98141	-0.93632	-0.15661	0.60294	1.03444	0.94791	1.8931	0.42034	-0.92943
S52	-1.18925	-0.88666	-0.40395	-0.00101	-0.47102	-0.27308	-0.93761	-0.98807	-0.0277	0.25383	0.53088	0.01961	1.32424	0.99834	1.37388
S53	-0.10571	-0.65635	-0.96543	-0.72055	-0.25827	-0.30097	1.3016	1.32577	-0.02424	-0.23276	-0.22308	-0.7029	-0.25652	0.31375	1.79406
S54	-0.31935	-0.70426	-0.64876	-0.23236	-0.46337	-0.5618	0.03173	0.10266	-0.05991	-0.44315	-0.64308	-0.79374	-0.2078	0.12165	0.36235
S55	-0.5451	-0.36437	-0.65054	-0.76923	-0.09792	0.11261	-1.05314	-1.1139	-0.05557	0.15943	0.75042	1.35895	1.91312	-0.20521	-1.66509
S56	0.13855	-0.33489	-0.32457	0.11442	0.2073	0.41488	-0.7543	-0.81473	0.08787	0.47476	0.24031	-0.18291	0.64776	1.29465	0.27588

S57	-0.04208	-0.56393	-0.70108	-0.45629	-0.23788	-0.03778	-1.13764	-1.19292	-0.31347	-0.0489	-0.31384	-0.23797	0.71777	0.71521	0.42863
S58	-1.04342	-0.82009	-0.72651	-0.65036	-1.08307	-1.16034	-1.48327	-1.41868	-1.00614	-1.45579	-1.44744	-1.24434	0.09965	-0.22809	-0.58907
S59	-0.88214	-0.84879	-0.98825	-0.93217	-0.8138	-0.80405	-1.40587	-1.34194	-0.67261	-0.83755	-1.00317	-1.19443	-0.57324	-0.53725	-0.49649
S60	2.44846	0.39672	0.13604	1.02612	1.71028	1.94165	1.20838	0.97143	0.70621	1.87795	1.1547	0.78989	0.85208	1.07372	0.73503
S61	-0.00565	-0.60084	-0.79356	-0.3957	-0.04047	-0.37748	-0.08132	0.11975	0.8883	0.38559	0.03585	-0.66451	0.03385	-0.33966	0.01427
S62	-0.77638	-0.7343	-0.80348	0.55361	1.28506	-0.61469	0.8313	1.07505	0.96253	-0.35728	-0.30453	0.10721	0.75105	-0.0725	0.04069
S63	1.85531	0.19312	0.15007	0.86842	1.14189	1.66227	-0.73165	-0.86296	0.56864	1.84673	1.00862	0.53934	1.4971	1.79013	0.36576
S64	-0.91015	-0.74156	-0.72593	-0.2928	0.10724	-0.15666	-0.32176	-0.29906	0.57381	0.54935	0.76786	0.09053	0.78183	0.71419	0.13599
S65	-0.00509	-0.22421	-0.18866	-0.049	-0.00636	0.13681	-1.09174	-1.04632	0.2465	0.74474	0.73981	1.2738	2.04211	1.03509	-0.65283
S66	0.01657	-0.40184	-0.40915	-0.10617	-0.02026	0.13004	-1.08155	-1.0438	0.15122	0.43586	0.28354	0.64297	1.47213	0.69218	-0.66406
S67	0.07995	-0.33246	-0.5094	-0.29269	0.37654	0.81549	-0.89138	-0.86301	0.72627	1.08039	0.84761	0.68931	1.0698	0.46289	0.31718
S68	0.5872	0.19638	0.34181	0.58418	0.05062	-0.23752	0.49164	0.48751	-0.24304	-0.25296	-0.15344	1.32627	1.76026	0.8342	0.00325
S69	1.51442	1.88074	1.68299	1.04816	1.55743	1.75278	-0.57285	-0.78319	0.4545	1.88259	2.00291	1.08033	-0.13766	-0.43621	0.22639
S70	-0.26352	0.13049	0.1332	-0.14509	-0.39621	-0.27021	-0.66022	-0.75721	-0.53895	-0.47573	-0.39435	-0.82815	-1.04758	-1.00967	-0.38699
S71	-0.35626	0.57025	0.84394	0.49465	-0.07597	0.23606	-0.38253	-0.54673	-0.19256	0.06642	0.41513	-0.21093	-0.64612	-0.66725	0.71988
S72	-0.51305	0.93561	1.39871	0.8618	0.2404	0.45675	0.67751	0.46158	-0.07146	0.05078	0.88796	0.23295	-0.61843	0.1189	1.02601
S73	-1.18925	-1.06024	-1.18998	-1.59578	-1.62626	-2.02609	1.38197	1.63917	-1.17818	-1.90726	-2.12704	-0.98344	-0.75956	-1.31949	-0.87349
S74	1.85434	1.16448	0.70363	0.44493	0.9993	1.42901	-0.05359	-0.25161	0.30319	1.30457	0.94663	0.14229	-0.56082	-0.24493	0.57165
S75	-0.70289	-0.04915	0.04823	-0.27104	-0.75504	-0.55969	-0.85043	-0.95	-0.78543	-0.84352	-0.68764	-1.09175	-1.1662	-1.26231	-1.20704
S76	1.22621	2.64855	2.23558	1.74759	4.81507	1.96482	-0.08173	-0.27474	0.31778	1.55084	2.82898	1.1836	-0.52885	-0.5752	0.96255
S77	-0.39969	-0.23538	-0.12645	-0.08932	-0.40716	-0.26098	-0.68649	-0.75599	-0.61656	-0.69462	-0.50809	-0.8737	-0.9501	-0.67784	0.02085
S78	0.72195	2.05103	2.07618	0.62942	0.81184	1.05395	-0.51981	-0.75108	-0.05471	0.55174	0.90596	0.5199	-0.63579	-1.06833	-1.29348
S79	0.2467	1.3693	1.19441	0.40418	0.97387	0.82934	-0.25572	-0.42087	-0.04986	0.51681	1.25784	0.70468	-0.30397	0.10297	0.49622
S80	0.57965	1.50643	1.50763	0.57621	1.15694	1.5668	0.22644	-0.14456	0.42242	1.38583	2.07698	0.81669	-0.53797	-0.7517	0.1994
S81	-0.52029	0.10402	0.56265	0.5253	-0.45801	-0.4567	1.15553	1.12401	-0.32142	-0.66776	-0.5941	-0.43243	-1.08104	-1.20838	-0.02864
S82	-1.18925	0.45823	0.56346	-1.36641	-1.62626	-1.96831	-0.39603	-0.43729	-1.24257	-1.90726	-1.1619	-1.00317	-1.35429	-1.67551	-1.89277
S83	2.0658	0.22561	2.13604	4.52188	1.41033	1.85186	-0.63014	0.3055	3.50072	1.88937	0.04361	0.42356	-0.40546	-0.12232	1.40666
S84	3.54866	1.02223	3.66197	6.64333	2.77556	3.35025	-0.27985	0.3129	3.1872	3.85812	1.27412	1.50586	0.10003	0.39545	2.68782
S85	0.3297	1.39919	1.64712	1.10942	4.00339	3.92671	0.1532	-0.68969	0.67453	2.91508	3.88416	1.6298	-0.19	-0.68558	-1.19662
S86	-0.20862	0.11454	0.29232	0.19997	-0.39428	-0.23279	-0.21709	-0.25917	-0.39636	-0.31022	-0.30298	-0.45031	-0.89858	-0.3468	1.82423
S87	1.21863	1.70291	2.22906	1.77961	0.67684	0.95192	-0.62531	-0.75912	0.27171	0.75932	0.57031	-0.24951	-0.61089	-1.01467	-1.29767
S88	-0.52517	1.97318	1.89968	-0.14516	-0.70742	-0.85413	-0.30555	-0.3484	-0.74625	-0.92906	-0.59306	-0.51356	-0.84158	-1.06443	-0.99588
S89	0.0259	0.53466	0.79802	0.36321	-0.39775	-0.26909	0.28548	0.23566	-0.38603	-0.34562	-0.41022	-0.36907	-0.44089	0.76389	2.20268
S90	-0.40698	-0.62279	-0.01922	0.63164	-0.68083	-0.4052	-1.2192	-0.4519	1.40426	-0.27172	-1.25628	-0.8005	-1.06008	-1.08533	-0.5686
S91	-0.6473	0.65235	0.56066	-0.59956	-0.14354	-0.1622	-0.44025	-0.55821	-0.29525	0.06188	0.34904	0.07064	-0.76634	-1.22176	-1.39114
S92	0.10766	0.07338	0.06082	0.09196	-0.06525	0.08977	-0.26354	-0.28441	0.35231	-0.04673	-0.1084	-0.44721	-0.74682	-1.09015	-1.15
S93	-0.2392	0.7984	1.52267	1.45027	0.14237	-0.24778	0.02484	-0.00677	-0.11663	-0.39246	-0.16693	0.11813	-0.65182	-0.99177	0.00635
S94	-0.82307	-0.43036	-0.6331	-0.8158	-0.53452	-0.10776	-0.32466	-0.54253	-0.66449	-1.0697	-0.05553	0.89428	-1.10397	-1.17843	-0.43028
S95	-0.12032	0.52023	0.0645	-0.79054	-0.58442	-0.57996	-0.38004	-0.43206	-0.60667	-0.71157	-0.57186	0.49529	-1.10435	-1.34998	-1.13638
S96	-0.04432	0.60657	0.43943	-0.10813	0.11068	0.46893	-0.47575	-0.63241	-0.08286	0.38122	0.55841	1.34131	-0.54886	-0.70977	-0.3364
S97	-1.16337	-0.47043	-0.36175	-0.56819	-1.12113	-1.13703	-0.52076	-0.526	-0.85518	-1.14681	-1.04329	-0.80972	-1.21243	-1.28131	-0.94315
S98	-0.16106	0.18614	-0.14731	-0.52431	-0.11462	-0.15182	0.2733	0.12002	-0.4378	-0.50456	0.20934	2.11522	-0.4306	-0.63506	-0.86986
S99	-1.18925	-0.44921	-0.56578	-1.08563	-1.37373	-1.49823	-1.3349	-1.27223	-1.15717	-1.63258	-1.60547	-1.10965	-1.33605	-1.53926	-1.86204
S100	0.29359	0.15852	0.14574	0.18503	0.06312	0.5687	0.19654	0.0015	-0.16938	0.03391	0.07631	0.04623	-0.8606	-0.64352	0.94397

S101	-0.09287	2.07502	2.00698	0.05264	-0.01097	0.26932	-0.28976	-0.50985	-0.36526	0.01483	0.4864	0.37668	-0.77527	-0.92135	-0.41939
S102	-0.35722	0.27623	0.54334	0.28824	-0.56135	-0.3826	0.05541	-0.04103	-0.58828	-0.76992	-0.6025	-0.80117	-1.12772	-1.26762	-0.92048
S103	3.11941	5.91525	3.94178	0.39386	1.14968	1.17777	0.46461	0.29515	0.64383	1.06648	1.9823	1.63884	-0.45152	-0.19398	1.40246
S104	0.16648	0.70884	0.99472	0.70161	-0.02259	0.23242	-0.5476	-0.63785	-0.06077	-0.03794	-0.05355	-0.50676	-0.7573	-0.50313	1.24462
S105	-1.16313	-0.14008	0.35379	0.36508	-0.24156	-0.38257	0.48925	0.34943	-0.35419	-0.47348	0.22076	0.44601	-0.88057	-1.07914	-0.29193
S106	0.39009	2.01253	2.07585	0.50083	0.67476	1.03529	-0.74901	-0.99073	-0.17076	0.53544	0.94802	0.60053	-0.44494	-1.02974	-1.32936
S107	-0.3565	2.28972	1.80824	-0.55724	-0.33111	0.05731	0.03782	-0.1805	-0.35052	-0.39741	0.06102	-0.15243	-1.06295	-1.15312	-0.55054
S108	-0.63573	-0.22814	-0.0724	-0.25748	-0.67774	-0.73255	-0.76817	-0.82936	-0.74584	-0.76641	-0.71813	-1.30958	-1.27619	-1.41098	-1.51221
S109	-0.18607	0.93247	0.8475	-0.00491	0.30844	0.57676	-0.33753	-0.42839	0.60222	0.40461	0.62625	0.10099	-0.34488	-0.92794	-0.81673

Sample No.	integrated bins														
	4.00 .. 3.98	3.98 .. 3.96	3.96 .. 3.94	3.94 .. 3.92	3.92 .. 3.90	3.90 .. 3.88	3.88 .. 3.86	3.86 .. 3.84	3.84 .. 3.82	3.82 .. 3.80	3.80 .. 3.78	3.78 .. 3.76	3.76 .. 3.74	3.74 .. 3.72	3.72 .. 3.70
S1	-0.90026	-1.03986	0.53095	0.50111	-0.51753	-0.50312	-0.44229	0.60228	1.18948	-0.48258	-0.75975	-1.29655	-0.82658	0.28665	-0.34617
S2	-0.56004	-0.374	0.17273	-0.51864	-0.49424	-0.53243	-0.3454	-0.55003	0.47371	-0.47089	-0.69162	-0.40674	-0.22839	-1.12354	-0.88107
S3	0.6085	-0.01807	-0.31337	-1.36007	-0.9658	-1.12787	-1.18519	-1.52672	0.38046	-0.7727	-0.80977	0.52379	1.23556	-0.49973	0.83091
S4	0.48959	-0.83064	-1.15642	-1.43326	-0.5324	-0.45968	-0.245	-0.36942	-1.00865	-0.46388	-0.12826	1.42416	0.82936	-0.22134	-0.12064
S5	-1.7677	-1.19525	0.18553	0.1999	0.07997	0.37324	0.878	1.23311	0.12619	0.18546	0.12655	-1.14475	-1.23896	0.80948	1.72099
S6	-0.48028	-1.02565	-0.86308	-0.54959	0.21522	0.21794	0.32982	-0.06567	-0.21606	0.29169	0.38825	0.37819	-0.317	0.40207	0.83358
S7	-1.27382	-1.3012	-0.60937	0.74577	1.22666	1.11267	1.1455	0.58283	-0.5619	1.26882	1.45878	0.03436	-0.94196	1.69122	0.75106
S8	0.72831	-0.48737	-0.91258	-1.56777	-0.89376	-1.02114	-1.15743	-0.85927	-0.08184	-0.72477	-0.37428	1.64115	1.33238	0.08489	1.04225
S9	-1.76098	1.52244	2.03883	0.434	-1.09003	-1.10237	-1.09948	-0.02944	0.83811	-1.10039	-1.57645	-1.71469	-0.8292	-0.40993	0.56491
S10	0.42161	0.582	0.05491	-1.83729	-1.17596	-1.33114	-1.43207	-1.61073	-1.95152	-1.42617	-1.14876	0.99797	0.85086	-1.33813	0.86146
S11	-1.3054	-1.1518	-0.77676	-1.03415	-0.55662	-0.42052	-0.13311	-1.18945	-2.79181	-1.02637	-1.08785	-1.31574	-0.86377	-1.79087	-2.05131
S12	-1.55714	-1.60872	-0.19224	0.76911	0.87664	0.91435	0.99783	0.56372	0.57291	0.87695	0.81848	-0.77012	-1.14192	1.2728	-0.01287
S13	-1.59264	-0.52243	-0.02744	-0.67137	-0.42426	-0.25122	0.21126	0.95164	0.6517	-0.4931	-0.5528	-1.04848	-0.81083	-0.04389	0.63644
S14	-1.24446	-0.40854	0.28922	0.6608	0.75228	1.16002	2.10976	1.10973	-1.30855	0.87124	0.77771	-1.04965	-1.31576	3.12264	2.3905
S15	-0.37008	1.76486	2.38643	-0.52482	-0.94616	-0.80467	-0.42473	0.15196	-0.85209	-1.26731	-1.37268	-1.29091	-0.3283	0.27053	0.31555
S16	-0.6268	0.06881	0.53824	-0.15131	-0.35179	-0.37519	-0.07165	0.07944	0.12109	-0.36658	-0.12477	-0.02197	-0.14377	-0.06921	0.48233
S17	1.10337	-0.08355	-1.53775	-2.01167	-1.26366	-1.47081	-1.37557	-0.03202	2.54592	-0.95565	-0.98918	0.73584	1.588	-1.48271	-0.08323
S18	0.24869	-0.33515	-1.73614	-1.58824	-0.93306	-0.94052	-0.66763	0.77317	2.64693	-0.73898	-0.8969	-0.02918	0.96947	-1.71009	-0.45927
S19	2.51708	1.3611	-0.82357	-0.43164	-1.32987	-1.49026	-1.76755	-3.50064	-2.13614	0.15622	-1.34431	-1.34598	4.0753	-0.61719	-2.68297
S20	0.91837	0.51355	-0.12026	-1.42822	-1.16854	-1.34471	-1.33265	-1.47569	-0.08035	-0.91402	-0.97778	0.77002	1.48845	-1.23346	-0.77119
S21	-1.84129	0.40906	2.9793	0.48706	-0.65513	-0.57307	-0.63414	-1.02834	-1.21982	-1.01816	-1.41124	-1.91014	-1.26863	-1.56929	-1.92016
S22	-0.18704	-1.20773	-0.07204	1.73849	1.57568	1.30175	0.75824	0.65298	0.80353	1.77992	1.78457	0.03278	-0.95542	0.31088	-1.63327
S23	-0.57097	-1.37119	-0.46343	-0.60057	-0.19564	0.15614	0.56361	-0.97831	-0.52266	0.1264	-0.10271	-0.43293	0.75724	-0.76911	1.20776
S24	-0.5292	-1.25617	-0.3376	0.12202	0.13368	0.34349	0.97653	0.71499	0.05131	0.36264	0.33796	-0.47452	-0.7178	1.5707	1.31321
S25	-0.04662	0.64841	1.19505	-0.16215	-1.08817	-1.14184	-0.95358	-0.50579	0.33566	-1.23453	-1.2307	-0.46814	0.27196	-1.1091	-0.84479
S26	0.6752	0.16789	0.41281	-1.00446	-1.28409	-1.35602	-1.40296	-1.29379	-0.19572	-1.21197	-1.21941	0.26485	1.19539	-1.87611	-1.04413
S27	1.2854	0.5219	-0.27811	-1.18297	-1.29324	-1.49791	-1.60986	-0.88882	0.43923	-1.20341	-0.81898	1.54552	1.76422	-0.96031	-0.54829
S28	0.99992	1.68189	2.32048	-0.25776	-1.44742	-1.723	-2.0091	-1.75594	-0.47083	-1.45179	-1.25444	0.77637	2.0146	-0.80322	0.02735
S29	0.85275	0.76755	0.84386	-0.60831	-1.28315	-1.50942	-1.77778	-1.41479	-0.0104	-1.25064	-1.05949	0.76243	1.70756	-0.50391	-0.03515
S30	0.55027	-0.15552	-0.84446	-1.72492	-1.05869	-1.19218	-1.14767	-0.64316	-0.23947	-1.09574	-0.84496	0.84166	1.18022	-1.55952	-0.2472
S31	-1.07623	-0.63847	1.71318	0.92883	-0.77195	-0.78648	-0.77185	0.61593	0.80588	-0.91426	-0.85446	-1.27034	-0.79328	0.19861	-0.76134
S32	-0.42582	-0.26034	0.33726	-0.6081	-1.1589	-1.21724	-0.98989	-0.98705	-0.08673	-1.00206	-1.37511	-0.89176	-0.06862	-1.34088	-0.77192

S33	-1.02443	-0.61878	1.10681	0.83099	0.0392	0.12763	0.29749	0.90881	0.64146	-0.03727	0.02454	-0.64996	-0.67528	1.00204	0.13722
S34	1.30647	0.69134	0.2892	-0.98384	-0.99836	-1.0355	-0.76751	0.09843	-0.00079	-0.80223	-0.29712	2.10904	1.10773	0.31921	1.20594
S35	0.34467	0.84061	1.98249	-0.0459	-1.37347	-1.50078	-1.52407	-0.87854	0.71775	-1.21714	-1.23037	-0.01049	1.04862	-1.533	-0.82675
S36	0.51619	0.77991	1.04922	-0.46368	-0.9203	-1.02502	-1.08669	-0.87043	0.64665	-0.79409	-0.62438	0.76265	1.12405	-0.62148	0.39326
S37	0.14939	-1.44216	-0.91835	1.0147	1.32303	1.03558	0.56654	0.37731	0.17413	1.39809	1.5053	0.33757	-0.70628	0.61565	-1.19516
S38	1.18773	0.28245	-1.01896	-1.47263	-0.746	-0.81389	-0.78412	-0.59282	-0.48767	-0.66939	-0.32707	1.74702	1.1329	-0.3616	0.27334
S39	-1.93317	-2.5097	-0.96562	0.5647	0.95685	1.1097	1.82199	1.48981	-0.27792	0.95361	1.24998	-0.82153	-1.25053	3.66341	1.89455
S40	0.34863	0.14017	-0.10658	-1.16017	-0.9858	-0.89891	-0.64341	-0.23034	0.06329	-0.84673	-0.75646	0.29115	1.03875	-1.1919	-0.63593
S41	2.0247	1.31811	-0.09948	-1.66098	-1.28525	-1.43593	-1.47821	-0.72547	0.82296	-1.07373	-0.67036	2.25394	2.29343	-0.74219	0.97567
S42	1.15273	1.02535	0.0937	-1.27526	-1.06179	-1.18546	-1.21248	-0.52645	0.80975	-0.94787	-0.70013	1.33414	1.38672	-0.36341	0.84368
S43	-1.02742	-0.03006	-0.39238	1.25183	1.35743	1.3944	1.83438	1.61722	0.62878	1.36034	1.03946	-0.87443	-1.21354	1.46048	0.43875
S44	-0.26371	-0.43128	-0.33135	1.67474	1.38257	1.25365	1.75034	2.05186	1.35582	1.50524	1.06833	-0.86221	-1.13249	1.60884	-0.30397
S45	-0.80802	-0.80603	-0.60706	1.57784	1.53158	1.38156	1.47721	1.44005	0.68986	1.51018	1.38505	-0.67122	-1.12759	1.27763	-0.55714
S46	1.33059	0.58394	-0.40754	-1.3337	-0.86047	-0.99949	-1.14934	-0.57549	0.91654	-0.65924	-0.31947	1.99439	1.6257	-0.61471	0.45234
S47	0.60822	0.37966	0.14327	-1.15546	-1.13733	-1.25881	-1.34839	-0.90941	1.05877	-0.88601	-0.84352	0.51579	1.28257	-0.9281	-0.18063
S48	1.4597	0.74553	-0.2604	-1.48737	-1.20932	-1.37826	-1.48839	-0.77211	1.19714	-1.00345	-0.76632	1.6225	1.79326	-0.62085	0.41563
S49	-0.15297	-0.1411	0.17509	-0.7426	-0.941	-0.9968	-1.09439	-1.10945	0.65945	-0.76663	-0.93101	-0.35221	0.5646	-0.95313	-0.65961
S50	-0.46967	-1.01393	0.21392	0.64725	-0.08244	0.08535	0.02889	0.92409	0.17335	-0.17661	-0.34776	-0.94938	-0.86534	0.04243	-1.05606
S51	-1.13068	-0.37389	1.81986	0.81621	-0.73817	-0.61352	-0.43139	0.74005	0.28985	-1.1649	-1.09307	-1.47849	-0.92996	1.01416	0.11881
S52	-0.69583	-0.58415	1.73412	0.94403	-1.14715	-1.17352	-0.9433	0.89649	1.13889	-1.2831	-1.38384	-1.42121	-0.76139	0.03013	-0.88185
S53	1.14457	-0.57695	-1.3757	-1.3985	-0.86085	-0.91556	-0.8909	0.02782	0.82613	-0.69281	-0.56323	0.9568	1.17632	-0.66226	0.01619
S54	-0.37106	-0.75952	-0.20205	-0.24269	-0.14892	-0.10964	-0.12374	-0.19077	0.07579	-0.16886	0.02513	0.21927	-0.0413	0.23022	0.40777
S55	-1.80773	-1.09925	0.03081	0.33826	-0.13052	-0.09502	0.34442	1.22349	1.0651	-0.40137	-0.49351	-1.25116	-1.0549	0.24591	0.26497
S56	-0.98443	-0.74021	0.41441	0.31136	-0.46153	-0.37127	0.11952	0.66538	0.80337	-0.55772	-0.8645	-1.40002	-0.93073	-0.348	-0.5884
S57	-1.11665	-1.0065	0.54365	0.75715	-0.29151	-0.20789	0.15669	1.26218	1.40551	-0.41197	-0.56765	-1.35552	-1.04345	0.48892	0.03815
S58	-1.45966	-2.1534	0.37608	0.66152	0.02594	0.23006	0.82691	1.60204	0.75033	-0.00987	0.16056	-1.13767	-1.10289	2.1222	1.05479
S59	-1.07448	-1.61359	-0.35601	1.37978	1.20752	1.06444	0.97612	1.19453	1.16286	1.11791	1.04624	-0.66042	-1.10097	0.74003	-0.72488
S60	0.78018	2.00054	2.35809	0.01001	-1.05167	-1.06556	-0.70784	0.60535	0.77074	-1.18449	-0.86879	0.55318	0.25633	-1.05041	-0.28093
S61	-0.0527	-0.10426	1.45623	0.57792	-0.68819	-0.66679	-0.66839	0.07659	-0.76251	-0.96892	-0.67725	-0.26699	-0.01311	0.42728	0.29676
S62	0.72169	0.10923	0.39949	-1.00769	-1.45968	-1.55061	-1.59121	-0.40128	-0.13752	-1.17861	-0.69136	0.94538	1.61138	0.27149	-0.10227
S63	-0.95198	-0.35511	1.08836	0.89116	-0.64642	-0.57941	-0.36201	1.78973	2.19721	-0.97332	-1.11913	-1.44128	-0.96751	-0.37392	-1.26299
S64	-0.2004	0.13853	1.60339	0.70051	-0.79895	-0.81341	-0.46261	0.90533	0.71765	-1.01856	-0.79584	-0.58194	-0.26305	0.55902	0.25725
S65	-1.45897	0.25275	1.62584	1.39866	-1.21643	-1.17474	-1.08687	1.0415	0.2411	-1.65123	-1.63449	-1.48281	-0.63334	0.09595	-1.56566
S66	-1.02066	-0.42658	1.91467	1.53728	-0.4995	-0.42839	-0.24096	1.51018	0.97589	-0.94435	-0.73828	-1.15208	-0.75895	0.89342	-0.50668
S67	-0.68026	-0.03089	2.28441	1.36924	-0.64946	-0.43096	0.74211	2.09765	-0.56958	-1.35781	-1.45549	-1.73663	-0.92573	0.5918	-0.04956
S68	-0.03707	0.38976	0.09553	-0.66047	-1.09527	-1.18965	-1.28073	-0.41558	0.91421	-1.03941	-0.9159	-0.04931	0.65807	-0.61932	0.4532
S69	0.97704	0.27874	0.23091	0.16096	0.1271	0.36492	0.63343	0.6498	-0.7385	0.03836	-0.4451	-1.30424	-1.07526	0.07735	-0.49688
S70	-0.56944	-1.18539	-1.10951	1.13549	1.82428	1.57976	0.82144	-0.04161	0.15969	1.77888	1.87688	0.50152	-0.59767	0.09778	-1.22188
S71	0.01247	-0.74117	-0.60151	0.5356	1.1318	1.03843	0.42558	-0.15087	-0.07823	1.06201	1.15689	0.38933	-0.38767	0.48798	-0.43461
S72	0.86866	0.70722	0.19214	-0.54034	0.12072	0.14309	-0.2031	-0.6089	-0.3605	0.06615	0.05174	0.6294	0.38824	0.05824	0.1323
S73	0.75184	-1.19273	-0.19055	-1.92579	-0.79671	-1.0685	-2.00366	-1.64698	-1.65971	-0.8104	0.01888	2.99964	1.86055	0.8063	1.16684
S74	0.10223	-0.08838	-0.19532	0.48788	0.97171	0.97511	0.85826	0.31236	0.03206	0.90814	0.76796	-0.00787	-0.54339	0.11493	-0.41457
S75	-0.51268	-0.9587	-0.67043	2.93387	2.80876	1.99696	0.7368	0.2456	1.2092	2.63306	2.94154	0.88079	-0.55255	-1.07075	-3.23729
S76	2.06445	2.3011	0.45339	-0.04832	0.38602	0.63234	0.78303	0.14704	-1.5297	0.22401	-0.27483	-0.83997	-0.74836	-0.39807	-0.15176

S77	-0.39727	-1.10515	-0.80769	1.13269	1.76726	1.53609	0.90707	0.20914	0.76636	1.78359	1.80722	0.37334	-0.63596	0.56124	-0.90902
S78	-0.24438	0.5847	-0.2536	0.52282	0.83274	0.99735	1.00738	0.21314	-0.95436	0.54427	0.5033	-0.49172	-0.74403	0.51511	0.1755
S79	0.21185	0.05255	-0.16703	0.16098	0.56358	0.62433	0.62233	0.39468	0.12653	0.43049	0.42034	-0.15491	-0.46398	0.65701	0.66682
S80	0.93303	1.12385	0.09676	0.06582	0.50002	0.64688	0.5809	-0.01942	-0.9459	0.29975	0.08935	-0.18394	-0.22778	-0.30412	-0.41647
S81	1.07552	0.14858	-1.45	-1.02756	0.00926	-0.03461	-0.30428	-0.7152	-1.14455	0.05965	0.34714	1.67382	0.93729	-0.29775	0.18945
S82	-0.42575	0.23728	-1.14252	0.03113	1.04135	0.80723	-0.14537	-1.08448	-1.13338	0.96881	1.29976	0.93369	0.06811	0.30392	-0.50202
S83	2.15541	1.132	-1.16503	-1.01162	-0.10094	0.80806	0.42361	-2.00806	-2.65142	0.25711	0.09368	-0.35538	1.04961	-1.62102	-0.8628
S84	1.02651	1.69869	-0.49707	-0.29293	-0.02604	0.88156	0.65448	-1.49572	-2.17074	0.40983	0.07973	-0.2751	0.88989	0.00378	0.23218
S85	-0.77567	1.4067	1.47295	0.39857	0.36741	1.04443	1.68782	1.20276	-1.31236	-0.26736	-0.44054	-1.22325	-0.81188	0.93702	0.36961
S86	0.641	0.15557	-0.1276	0.31366	0.95989	0.9468	0.45419	-0.60833	-0.18265	1.16185	0.88645	0.26132	-0.28289	-0.33715	-0.37164
S87	-0.72121	-0.07929	-0.30677	0.62855	0.82635	0.96079	1.24346	0.7527	-0.64556	0.53007	0.38531	-0.82266	-0.93109	0.60685	-0.54309
S88	-0.52997	-0.727	-0.68459	0.09577	0.55784	0.57705	0.62446	0.45592	-0.38093	0.43219	0.66724	0.32807	-0.28162	1.04028	0.83882
S89	0.07159	-0.87775	-1.37058	-0.708	0.37237	0.31048	-0.11009	-1.08645	0.35531	0.56597	0.4661	0.48843	0.17961	-0.87655	-0.61424
S90	0.09253	-0.5521	-1.00952	-0.32387	0.3225	1.42047	0.96742	-1.49084	-2.59022	0.40889	0.48108	0.02833	0.40108	-1.56492	0.47107
S91	-0.49027	1.83723	1.23253	0.94327	0.87637	0.82315	0.71079	0.29348	-0.51376	0.77571	0.74832	-0.13548	-0.49234	-0.23726	-0.35246
S92	-0.0533	-0.27651	-0.79459	1.14493	1.65737	1.47837	0.93099	0.66142	0.46701	1.52304	1.73481	0.70926	-0.39841	0.77089	-0.50111
S93	0.03246	-0.29893	-0.73744	-0.39989	0.13625	0.14042	0.14393	0.00594	-0.87124	0.19286	0.37217	0.47726	0.04538	0.87111	1.2658
S94	1.68252	2.78524	-0.64127	0.29996	0.89894	0.85853	0.73861	-0.29387	-0.56363	1.10343	0.58176	-0.09957	-0.2785	-0.26755	1.31384
S95	-0.27977	0.20903	-1.12321	0.83781	1.53767	1.36514	0.75216	-0.01403	-0.20936	1.42178	1.72951	0.90123	-0.20869	0.55266	1.15681
S96	-0.04926	1.13473	-0.17087	0.67718	0.83394	0.88337	0.99214	0.61245	-0.13503	0.6289	0.64127	-0.25301	-0.53637	0.79254	2.13941
S97	-0.50293	-0.80806	-1.30232	1.23429	1.84381	1.49788	0.86235	0.36586	0.48843	1.86207	2.04334	0.89826	-0.42355	0.44263	-0.51463
S98	0.23576	2.29314	-0.31435	0.02744	0.45863	0.40152	0.44028	0.17302	-0.33647	0.34084	0.43628	0.6296	0.0932	0.7351	3.1615
S99	-1.2499	-1.08084	-1.04726	1.95196	2.19226	1.85862	1.57397	1.12367	0.94403	2.25314	2.32164	0.06221	-0.90114	1.39515	-0.10207
S100	1.05687	0.79468	-0.59244	-0.01671	0.6738	0.72613	0.66041	-0.12257	-0.32264	0.86319	0.53784	0.27445	-0.142	0.09845	0.58849
S101	0.57622	0.60615	-0.38167	0.2069	0.69567	0.79654	0.93575	0.50543	-0.29094	0.65484	0.54393	-0.07904	-0.35441	0.57136	1.26224
S102	-0.0115	-0.55292	-1.05717	0.55879	1.36029	1.14056	0.51073	-0.01249	-0.09093	1.32365	1.55915	1.15621	-0.12837	0.34079	-0.5236
S103	1.10169	1.30408	-1.25126	-1.29223	-0.41829	-0.31975	-0.3394	-1.054	-2.15672	-0.52376	-0.71375	-0.41651	0.26125	-1.19551	1.80058
S104	0.16643	-0.39513	-0.87959	0.28675	0.81739	0.87744	1.01655	0.30697	-0.24389	1.06122	0.53946	-0.55191	-0.83511	0.32694	0.00013
S105	0.60717	0.39775	-0.96742	-0.48687	0.29451	0.24048	0.00969	-0.31499	-1.02043	0.25031	0.41613	0.99352	0.28225	0.09134	0.78296
S106	-0.4737	0.4338	-0.01634	0.3958	0.4974	0.92619	1.48713	0.82992	-1.0216	0.28485	0.18002	-1.01784	-0.98562	1.44611	1.51675
S107	2.69303	2.22612	-0.80598	-0.57986	0.38485	0.46379	0.29603	-0.72929	-1.05514	0.70583	0.25809	-0.0976	-0.07274	-0.26936	0.26212
S108	-0.70235	-0.15726	-0.60659	1.29505	1.52053	1.55402	2.22284	1.73074	0.60536	1.64559	1.3082	-0.29045	-0.84268	1.73857	0.38364
S109	1.25808	1.22724	1.03026	0.34629	0.14977	0.36628	0.76457	0.88021	-0.79754	0.07565	-0.11054	-0.7883	-0.75583	0.95377	0.68247

Sample No.	3.70 .. 3.68	3.68 .. 3.66	3.66 .. 3.64	3.64 .. 3.62	3.62 .. 3.60	3.60 .. 3.58	3.58 .. 3.56	3.56 .. 3.54	3.54 .. 3.52	3.52 .. 3.50	3.50 .. 3.48	3.48 .. 3.46	3.46 .. 3.44	3.44 .. 3.42	3.42 .. 3.40
S1	-0.57895	1.21178	1.09382	-0.06746	-2.02487	-1.30151	-0.88231	-0.36337	-0.18068	0.12312	0.10122	1.14362	1.13804	0.19719	-0.01889
S2	-1.04443	-0.6126	-0.6614	-0.91722	1.27546	0.84128	0.01385	-0.64654	-0.75822	-0.92041	-0.59313	-0.84822	-0.59138	-0.28006	-0.32078
S3	0.38632	-1.06271	-1.30705	-1.22058	-0.10703	-1.61773	-1.22181	-0.87427	-0.77588	0.64352	0.84966	-0.28629	-0.47814	0.30405	0.66728
S4	0.03668	-0.71269	-0.58618	1.17323	0.66318	-0.22086	-0.32856	-0.33212	0.16878	0.99217	0.78017	-0.26527	-0.0479	0.6608	0.91399
S5	2.09705	1.86948	1.97665	-0.46915	-1.73328	-0.8093	-0.23164	0.82813	1.36712	0.14136	-0.14309	1.35971	1.65342	1.43816	1.22719
S6	0.89395	-0.23477	-0.06784	0.31356	0.21063	0.15898	0.18435	0.47713	0.92526	-0.10494	-0.49799	-0.77157	-0.6534	-0.10206	0.00624
S7	1.47416	0.18032	0.43807	0.72499	0.51436	1.14217	1.17565	1.50528	1.45765	-0.87063	-1.41697	-1.06845	-0.99211	-0.92465	-0.89903
S8	0.49751	-0.55283	-0.66683	0.83243	-0.0067	-1.28517	-1.00566	-0.6078	-0.18386	1.64498	1.51963	0.09647	-0.08207	1.35958	1.7255

S9	-1.55462	0.1798	0.94371	-0.45263	-2.35425	-1.06537	-1.40446	-1.24997	-1.2402	-1.30859	0.17037	0.97035	1.11682	0.03576	-0.2668
S10	-1.70554	-2.13158	-2.00599	-0.2956	2.01822	0.28538	-0.72912	-1.48892	-1.58209	-0.0239	0.29868	-1.44595	-1.2617	0.06056	0.38387
S11	-1.86132	-0.73328	-0.63059	-1.50791	-1.04089	-0.63421	-0.73075	-0.64631	-0.83854	-1.54579	-1.1004	-0.70006	-0.54519	-1.04519	-1.07909
S12	0.84732	0.70739	0.97166	-0.23504	-0.68521	0.29724	0.67771	1.30389	1.41668	-0.32838	-0.95903	-0.27694	-0.29137	-0.58382	-0.77538
S13	0.27993	0.66433	0.69032	-0.92846	-1.21853	-0.89028	-0.6273	-0.07685	0.35305	-0.12724	-0.28507	0.54799	0.70437	0.61319	0.31088
S14	2.96226	1.00955	1.27104	-0.10526	-0.56072	0.284	0.57791	1.78747	2.30237	-0.05193	-1.59771	-1.13262	-1.06423	-1.26677	-1.18033
S15	-0.41139	0.13266	-0.29727	-1.24242	-0.90543	-0.64537	-0.95526	-1.14741	-1.18382	-1.07907	-0.23995	0.65339	0.9218	-0.13269	-0.50489
S16	0.67397	0.5064	0.52931	-0.18258	0.21901	-0.10232	-0.22283	-0.07335	0.17772	0.26758	0.09979	0.24897	0.45689	0.83325	0.87603
S17	0.26094	-1.10706	-1.44259	-1.41768	0.01439	-1.9146	-1.56798	-1.28768	-1.34899	0.43031	1.10853	-0.03877	-0.06546	1.029	1.27514
S18	-0.0025	-0.95859	-1.07458	-1.71024	0.16563	-0.87332	-1.03339	-1.03187	-1.09151	-0.01379	0.36333	-0.39919	-0.28101	0.17051	0.21508
S19	1.17113	-0.36094	-1.77221	-3.26998	0.98164	-1.23572	-0.12382	-1.61512	-1.88436	-1.73891	1.28111	2.4545	-0.96686	-2.02853	0.94557
S20	-0.20595	-1.28509	-1.57243	-0.88549	0.90913	-0.46953	-0.73496	-1.14101	-1.28545	0.3275	1.08484	0.03975	-0.13681	0.85901	1.1438
S21	-2.24412	-0.51561	-0.25261	-1.36761	-0.0086	3.75726	0.75777	-0.57394	-0.76712	-1.52977	-0.7362	0.32343	0.85968	-0.62712	-1.02179
S22	-0.58065	-0.03154	0.42934	0.92885	0.04394	0.82924	1.01819	0.99077	0.44366	-1.72907	-1.56725	-1.23548	-1.29978	-1.03825	-0.98893
S23	2.25198	-0.20487	0.04148	-1.11227	0.21731	1.59178	0.50961	0.01948	1.36738	1.97874	-0.15122	-1.19412	-1.3415	-1.18967	-0.71259
S24	1.5213	0.99019	1.24281	0.3677	-0.57326	-0.10797	0.12202	0.79508	1.5297	1.02998	-0.44024	-0.40841	-0.3932	-0.29034	-0.26222
S25	-1.30998	-0.18644	-0.39449	-0.76637	-0.2946	-0.916	-1.23876	-1.3109	-1.36737	-0.04135	0.52465	0.31222	0.54475	0.71384	0.56574
S26	-1.43173	-1.2279	-1.29655	-1.20558	1.37448	-0.09949	-0.90938	-1.37377	-1.55827	0.57259	0.92852	-0.25811	-0.07273	0.77804	0.95261
S27	-0.52778	-0.72534	-0.90366	0.29299	2.41206	0.27479	-0.57703	-1.28332	-1.45142	1.7472	1.93706	0.22702	0.34719	1.78141	2.09339
S28	-0.33312	-1.05899	-1.41457	-1.13039	0.24663	-1.88147	-1.69999	-1.52027	-1.75015	1.15209	1.80524	0.39532	0.08129	1.23255	1.71324
S29	-0.35771	-0.4972	-0.79831	-0.59522	0.04139	-1.76499	-1.51902	-1.27114	-1.4454	1.29206	1.77217	0.65527	0.33355	1.33168	1.6737
S30	-0.51597	-0.68152	-0.83307	-0.96618	1.77445	0.12077	-0.63802	-1.15148	-1.03558	0.28941	0.97122	0.10513	0.55007	1.82283	1.9083
S31	0.0921	2.22162	1.59436	0.3146	0.00112	0.40341	-0.29344	-0.76811	-0.62759	0.52181	0.85788	1.9218	2.26654	1.16411	0.73945
S32	-1.24589	-0.14671	-0.15387	-0.87696	-0.29967	0.11795	-0.82749	-1.23945	-1.48712	-0.63464	0.33858	0.65323	0.93817	0.63098	0.36563
S33	0.32252	1.14797	1.25444	0.31645	-0.74317	-0.23177	-0.0683	0.42392	0.7054	0.68565	-0.00332	0.31777	0.29919	0.20186	0.10681
S34	0.90984	-0.45363	-0.30928	2.59741	0.70281	-0.57208	-0.8286	-0.6853	-0.22687	2.56437	1.65853	-0.46964	-0.54647	1.35385	1.71559
S35	-0.85075	-0.59574	-0.76898	-1.05095	0.78946	-0.21392	-1.08094	-1.49451	-1.58494	0.47328	1.03087	0.13515	0.16062	0.84049	0.97212
S36	-0.29124	-0.76466	-0.94903	-0.52345	-0.25063	-1.61983	-1.29366	-0.94417	-0.99163	0.89452	0.86296	-0.44309	-0.58448	0.558	0.86514
S37	-0.34454	-0.05203	0.26205	1.33768	-0.37239	0.29008	0.61026	0.76891	0.23541	-1.14112	-1.10713	-1.05408	-1.08398	-0.79981	-0.82472
S38	-0.31647	-1.06897	-1.0074	1.20259	0.91656	-0.5186	-0.75181	-0.74343	-0.79223	1.08714	0.9375	-0.76605	-0.64588	0.85072	1.17725
S39	3.28682	1.79362	2.07919	0.37464	-0.39237	0.6026	0.8869	2.06086	2.68263	0.68973	-0.92665	-0.33738	-0.353	-0.42047	-0.58048
S40	-0.66383	-0.5947	-0.60895	-0.49049	1.09085	0.66787	-0.73714	-1.21944	-1.30109	0.07463	0.56203	-0.40792	-0.2106	0.44649	0.52175
S41	-0.3955	-1.65554	-1.69747	0.31749	1.42859	-1.14325	-1.33936	-1.40208	-1.30178	2.09402	1.87702	-0.94121	-0.99123	1.24887	1.76682
S42	-0.45526	-0.97497	-1.12106	0.22008	0.00499	-1.52058	-1.41003	-1.19831	-1.20847	1.12225	1.25989	-0.41166	-0.50519	0.90424	1.19072
S43	1.83943	1.15305	1.27898	-0.08084	-0.15415	0.88949	1.24142	1.78687	1.60182	-0.49949	-0.985	0.02035	0.39371	-0.16334	-0.6065
S44	1.21038	1.10037	1.38525	0.21205	-0.19006	0.86517	1.15159	1.69822	1.29703	-0.74329	-0.90529	-0.24212	-0.23029	-0.23325	-0.54716
S45	0.49317	0.59087	0.90473	0.41	0.63962	1.46895	1.39842	1.52034	1.04803	-1.26637	-1.54492	-0.92159	-0.81891	-1.06383	-1.17538
S46	-0.32702	-1.32947	-1.34112	0.72527	1.14407	-0.68499	-0.83314	-0.90693	-0.94278	1.51723	1.34089	-0.81793	-0.89561	0.86427	1.3292
S47	-0.7364	-0.55784	-0.96903	-0.85669	1.1884	-0.23569	-0.84004	-1.20529	-1.27676	0.42031	0.94219	-0.02552	-0.18332	0.81149	1.09121
S48	-0.33099	-0.98074	-1.16953	0.14375	0.39698	-1.54684	-1.42704	-1.21305	-1.18059	1.60206	1.72184	-0.14634	-0.30894	1.38179	1.78993
S49	-0.8111	-0.21468	-0.54895	-1.24506	0.07555	-0.41686	-0.76081	-0.9617	-1.10555	-0.17316	0.46111	0.39171	0.25245	0.37523	0.50551
S50	-1.38726	0.71532	0.96589	0.10998	-0.94043	0.2368	-0.19502	-0.27329	-0.42832	-0.55323	-0.1728	0.29512	0.55452	-0.1217	-0.32682
S51	-0.80371	1.68417	1.46825	0.12322	-1.14495	-0.45052	-0.79233	-0.63256	-0.17511	0.73117	0.31484	1.10775	1.41978	0.60069	0.22538
S52	-0.85166	1.94885	1.50867	0.30204	-1.98799	-1.35336	-1.44777	-1.26414	-0.98596	0.00871	0.68278	1.81768	1.95109	1.33309	0.80479

S53	0.00302	-0.80484	-0.90717	-0.25083	1.35359	-0.1501	-0.54267	-0.81732	-0.77437	0.94495	0.97177	-0.27591	-0.27706	0.65229	0.94061
S54	0.22444	0.15009	0.18541	0.12234	0.33971	0.02347	-0.05781	0.04974	0.16576	0.4266	0.07455	-0.15201	-0.05966	0.21497	0.31283
S55	0.02359	1.35286	1.18211	-0.53727	-1.51704	-0.66292	-0.40514	-0.07079	0.05812	-0.50734	-0.40174	0.71855	0.96283	0.43349	0.05626
S56	-0.54975	0.99771	0.8054	-0.65765	-0.1731	0.29012	-0.24921	-0.41377	-0.24627	-0.48782	-0.41715	0.50556	0.74374	0.11263	-0.07748
S57	-0.1446	1.61388	1.37052	-0.02385	-1.23825	-0.41594	-0.39727	-0.06696	0.28251	0.3201	-0.05045	0.98962	1.17253	0.42475	0.08367
S58	2.00995	2.29226	2.58896	0.19791	-1.16329	-0.20116	0.01731	0.81056	1.71356	1.54216	0.31737	1.02916	0.99024	0.80999	0.42493
S59	-0.10589	0.52278	0.84002	0.11688	0.53988	1.38555	1.26562	1.10421	0.73047	-1.03012	-1.28401	-0.63046	-0.61019	-0.99926	-1.09034
S60	-1.30687	-0.07242	-0.17459	0.25235	-1.65143	-2.18368	-1.86943	-1.37259	-1.32659	0.41294	0.82821	0.05336	0.3814	1.71302	1.42093
S61	0.1435	0.89025	0.59499	0.48999	-1.00549	-1.19364	-0.99452	-0.66705	-0.52961	0.82596	1.04701	1.56297	1.22831	0.85212	0.76315
S62	-0.14323	1.04628	0.27693	0.74509	2.5036	1.03621	-0.37797	-1.34868	-1.11032	1.21242	2.05703	1.49631	1.27804	2.13935	2.14807
S63	-1.00456	1.99171	1.49901	-0.126	-2.39702	-1.53145	-1.28479	-0.85111	-0.73694	-0.03545	0.56083	1.90632	2.53144	1.69269	0.98897
S64	0.33714	1.60819	1.17148	0.30521	-1.77002	-1.63249	-1.38814	-0.94672	-0.71137	0.36573	0.73882	1.38675	1.19383	1.09232	0.80169
S65	-1.9973	2.09288	2.36592	1.46694	-0.43082	1.30712	-0.50085	-1.13265	-0.60924	1.21577	1.94162	3.12863	3.58368	1.90593	1.28321
S66	-0.69417	2.26957	2.02825	0.70205	-0.42867	0.25539	-0.39047	-0.56246	0.02388	1.23745	0.9079	1.93924	1.77154	0.82246	0.52631
S67	0.91157	2.55558	1.70195	0.09737	-1.10845	-0.42689	-0.86303	-0.80994	0.61163	1.80116	1.73309	4.54529	4.19598	1.00658	0.30139
S68	-1.03471	-0.00739	-0.11011	-0.44034	0.62857	-0.2484	-0.84096	-1.03152	-0.80873	0.88079	0.91332	0.32038	0.38612	0.88083	1.02153
S69	-0.13492	0.68632	0.67996	-0.83844	-0.10654	0.5168	0.22843	0.1248	0.15116	-0.94698	-0.73588	0.38303	0.81325	0.02927	-0.56823
S70	-0.5765	-0.61974	-0.35357	0.72083	0.63844	1.30894	1.5835	1.29947	0.62854	-1.25955	-1.39998	-1.08596	-0.9987	-1.54869	-1.52128
S71	-0.32801	-0.11357	0.01791	0.37863	-0.41614	0.13998	0.69423	0.78757	0.35239	-0.61149	-0.67475	-0.24337	-0.32941	-0.81395	-0.94462
S72	-0.25059	-0.68872	-0.82921	-0.14327	-0.43499	-0.74782	-0.29921	-0.02787	-0.0412	-0.10978	0.06213	-0.28755	-0.50419	-0.30287	-0.25808
S73	1.22201	-0.94053	-0.9994	3.06191	-0.12037	-0.96863	-0.60749	-0.46709	1.97613	3.20273	2.84993	0.8614	0.24871	1.65277	2.23608
S74	-0.32535	-0.37461	-0.25931	0.08445	-0.03303	0.27629	0.44571	0.58665	0.43462	-1.28566	-1.32841	-1.1771	-1.05122	-1.1533	-1.19148
S75	-2.32268	-1.17575	-0.54724	2.08155	1.21063	1.89858	1.91225	0.86347	-0.24841	-2.36703	-1.81088	-1.3679	-1.3261	-2.04831	-1.97038
S76	-0.15616	-0.15728	-0.48155	-1.45856	-0.92726	-0.41188	-0.2006	-0.05445	-0.37187	-1.67982	-1.2859	-0.32503	0.27313	-0.93002	-1.43305
S77	-0.2754	-0.48551	-0.2285	0.76623	0.04394	0.8463	1.3542	1.39083	0.83968	-1.11874	-1.26299	-0.84482	-0.78047	-1.36598	-1.40085
S78	0.15333	0.35832	0.39775	-0.16508	-0.25404	0.57485	0.83503	0.94478	0.77053	-0.40399	-0.58718	0.37301	0.48598	-0.38504	-0.98447
S79	0.32102	0.39335	0.36338	-0.12564	-0.29029	0.1011	0.24878	0.4196	0.36238	-0.5707	-0.65022	-0.14387	0.03373	-0.34048	-0.55136
S80	-0.42513	-0.09023	-0.25804	-0.61439	0.23147	0.38215	0.3156	0.13459	0.0059	-0.90354	-0.66415	-0.29486	-0.29924	-0.59955	-0.90338
S81	-0.1946	-1.21978	-1.08842	1.2322	3.18638	2.14386	1.15851	0.05803	-0.16641	0.69216	0.37634	-1.09544	-1.09486	-0.05855	0.2391
S82	-0.10576	-0.61641	-0.61436	0.62255	-0.26358	0.16519	1.01049	1.24981	0.72791	0.20638	0.11229	0.33003	0.13311	-0.41428	-0.6059
S83	0.47537	-0.63305	-0.70177	-2.06651	-0.2215	0.32591	2.50628	0.03253	-0.36292	-0.6961	-0.3976	0.47147	-0.68087	-1.74146	-0.56362
S84	1.60582	0.15578	0.43133	-1.12261	-0.81277	-0.58137	0.63809	-0.062	-0.07899	-0.3371	-0.12385	0.98968	-0.16217	-1.32225	-0.2736
S85	1.28626	1.10146	1.02135	-0.70458	-1.46958	-0.54075	-0.11914	0.40807	1.34905	-0.56752	-0.25817	1.45428	1.64516	0.54985	-0.56545
S86	-0.83698	-0.97931	-0.83924	-0.54388	0.32248	0.48365	0.80075	0.73063	-0.01774	-1.09784	-1.15795	-1.16348	-1.14845	-1.48811	-1.30422
S87	0.20603	0.61286	0.69974	0.04943	-0.23122	0.44658	0.6643	0.9709	0.75807	-0.64887	-0.60688	0.4086	0.61128	-0.23189	-0.76795
S88	0.96685	0.36036	0.57688	0.89151	-0.22106	0.10305	0.35944	0.82879	0.99779	0.39444	-0.12549	-0.15041	-0.1897	0.1218	-0.08535
S89	-0.8802	-1.15797	-1.02827	-0.77993	1.34873	0.78543	0.58601	0.23871	-0.21238	-0.45852	-0.6783	-1.27301	-1.15151	-0.93599	-0.6795
S90	1.05755	-0.16059	0.04558	-1.50741	-0.39462	0.77112	3.33812	0.91744	1.08338	0.74737	-0.36963	0.33306	-0.46347	-1.80721	-0.68493
S91	-0.38795	-0.21188	-0.10638	0.06304	-0.09966	0.37102	0.58804	0.63089	0.28553	-0.95749	-0.88783	-0.43455	-0.4266	-0.79421	-1.01188
S92	0.12598	-0.0869	0.17608	1.65424	-0.23412	0.37638	0.88723	1.14429	0.70435	-0.71212	-0.74392	-0.66553	-0.8035	-0.80175	-0.85057
S93	1.0848	0.41869	0.43384	0.84444	-0.01507	0.04721	0.12488	0.44594	0.94251	0.75521	0.33332	0.14647	-0.02186	0.3151	0.26198
S94	0.34214	-0.44037	-0.47096	-0.4553	-0.49602	0.05976	0.66948	1.09724	0.89166	-0.50428	-0.63401	-0.18069	-0.17423	-1.00224	-1.20814
S95	0.10623	-0.56103	-0.32371	1.30997	0.42311	0.98369	1.31566	1.34893	0.82032	-0.55873	-0.85937	-0.83029	-0.80311	-1.07035	-1.05942
S96	0.61753	0.31558	0.41899	0.24117	1.51936	1.76865	1.09964	0.68075	0.54955	-0.7535	-1.05434	-0.67058	-0.46813	-0.86222	-0.95962

S97	-0.27222	-0.70088	-0.37544	1.79714	1.4361	1.84995	1.77922	1.39395	0.65837	-1.03306	-1.25971	-1.38862	-1.36472	-1.4521	-1.26615
S98	0.8288	-0.46252	-0.38642	1.21197	-0.40777	-0.13315	0.22165	0.73791	0.44431	0.16583	-0.03291	-0.23842	-0.25316	-0.26233	-0.34686
S99	0.52422	-0.02589	0.3507	1.20893	0.70895	1.7045	1.99613	2.13778	1.45713	-1.15655	-1.57709	-1.01926	-0.99433	-1.54087	-1.51137
S100	-0.01312	-0.5868	-0.56248	-0.07234	0.12543	0.21451	0.46968	0.648	0.34863	-0.42188	-0.60064	-0.7222	-0.70417	-0.85358	-0.83021
S101	0.79538	0.43091	0.28816	0.03757	-0.31082	0.04892	0.44518	0.78279	0.66594	-0.26656	-0.43995	0.07496	0.35792	-0.25006	-0.53968
S102	-0.16639	-0.80526	-0.60272	1.64749	0.43208	0.7674	1.09809	1.07883	0.45139	-0.4968	-0.67793	-1.01666	-1.0029	-0.87433	-0.79158
S103	-0.68687	-0.69835	-0.78848	-1.38792	0.11106	-0.09977	-0.41503	-0.52003	-0.89437	-0.71412	-0.1064	0.17888	0.34151	0.0587	-0.25479
S104	0.30405	0.00061	0.19517	-0.32838	0.21976	0.71317	0.74131	0.89618	0.74928	-0.65416	-1.08448	-0.59674	-0.4768	-0.90624	-1.01239
S105	0.1602	-0.522	-0.48546	1.05251	-0.3597	-0.43493	-0.04945	0.28026	0.20056	0.16222	0.1535	-0.42591	-0.57361	0.02935	-0.06212
S106	1.38177	1.22786	1.28281	-0.19689	-0.60804	0.30609	0.48549	0.99806	1.29601	0.28856	-0.482	0.59607	0.83796	0.05473	-0.65774
S107	0.20736	-0.22919	-0.40829	-0.8668	-0.59093	-0.45357	0.2177	0.63847	0.4312	-0.06843	-0.21404	0.1828	0.16994	-0.47817	-0.68877
S108	1.47104	0.2471	0.57017	0.85971	-0.05016	0.92712	1.43369	2.12007	2.16577	-0.15809	-1.20354	-0.87514	-0.87844	-1.51413	-1.53801
S109	0.81602	1.02188	1.07501	0.14222	-1.16323	-0.62415	-0.28432	0.36207	0.76207	0.2822	0.00301	0.67944	0.54677	0.2511	-0.2336

Sample No.	integrated bins														
	3.40 .. 3.38	3.38 .. 3.36	3.36 .. 3.34	3.34 .. 3.32	3.32 .. 3.30	3.30 .. 3.28	3.28 .. 3.26	3.26 .. 3.24	3.24 .. 3.22	3.22 .. 3.20	3.20 .. 3.18	3.18 .. 3.16	3.16 .. 3.14	3.14 .. 3.12	3.12 .. 3.10
S1	-0.06755	-0.07893	1.04846	1.72996	1.34288	0.55917	0.46525	0.15101	-0.28967	0.75258	1.73826	2.03551	2.08274	1.81787	0.84122
S2	-1.23648	-0.75025	-0.67458	-0.63717	-1.0653	-0.70298	-0.77024	-0.88562	-0.78227	-0.17943	0.54456	0.31308	-0.36088	-1.25255	-0.80006
S3	-0.048	-0.90539	-1.08188	-0.58193	0.24721	0.34285	0.23565	-0.23742	-0.81679	-0.07351	1.05372	1.41186	1.55926	1.4069	0.20258
S4	0.73041	-0.26247	-0.52341	-0.58524	0.30879	1.01039	0.82403	-0.11282	-0.45909	-0.89088	-0.88202	-0.93373	0.98212	-0.13927	-0.38674
S5	2.98808	1.81696	3.41368	3.2473	1.9442	0.84418	0.48416	0.42115	0.76341	1.80445	1.22503	1.39869	1.26366	1.74458	1.04273
S6	0.82876	0.92554	0.53102	-0.35644	-0.35582	0.31809	0.13145	-0.4861	0.64835	0.79207	-0.36178	-0.56585	0.0652	-0.76384	-0.41543
S7	0.59938	1.71111	0.54073	-0.71746	-1.16276	-0.01996	-1.32331	-0.75958	2.02607	1.55233	-0.50951	-0.6977	-1.2429	-1.46081	-0.6675
S8	1.66109	-0.13173	0.04134	0.03184	0.56445	1.09207	1.08418	0.08923	-0.38344	0.49582	0.54673	0.63635	1.94953	0.99453	0.17622
S9	-0.9483	-1.18201	0.01775	1.5329	1.51156	0.37328	0.54341	0.54012	-0.60095	-1.47027	1.3537	2.3685	-0.55175	1.49492	-0.27408
S10	-1.38677	-1.68489	-1.99911	-1.5626	-0.95781	-0.59053	-0.49882	-0.66346	-0.93078	0.91884	1.82784	1.35366	0.14804	-0.92119	-0.59495
S11	-2.14041	-0.93339	-1.06986	-0.76748	-0.88643	-1.20907	-1.25103	-0.95041	-0.83144	-1.28561	-0.78474	-0.86574	-1.38665	-1.23476	-0.80006
S12	0.53851	1.62751	1.10868	0.13417	-0.29391	-0.39939	-0.65809	-0.0868	1.6584	1.689	-0.28201	-0.36045	-0.70654	-0.39476	-0.06178
S13	0.95849	0.74988	1.35082	1.19769	0.6264	0.22101	0.28875	0.76873	0.71903	1.33886	0.52209	0.38315	-0.07077	0.0594	0.16061
S14	1.27881	2.59415	0.9686	-0.64437	-1.26688	-1.21822	-1.21977	-0.20891	3.23187	2.54184	-0.47469	-0.63643	-1.04997	-1.21323	-0.43772
S15	-1.66659	-1.33261	-0.97501	0.27587	2.21409	2.26869	3.09267	5.6357	1.1392	-0.36198	0.14735	0.59383	1.11994	2.18011	1.41984
S16	1.33638	0.523	1.04241	0.69099	-0.52635	-0.69515	-0.73282	-0.76275	-0.16801	0.51705	0.24368	0.01263	-0.14501	-0.53002	-0.5809
S17	0.16276	-1.36198	-1.18987	-0.41988	0.01883	0.01159	-0.09088	-0.44181	-1.21616	-1.40923	0.09498	0.13593	0.92097	0.86111	-0.29774
S18	-1.04982	-1.27949	-1.35771	-0.78228	-0.55013	-0.60593	-0.64603	-0.83975	-0.8905	-1.38592	-0.08076	-0.19967	-0.06304	0.73623	-0.5775
S19	3.48144	-1.72363	-2.23065	-1.07918	-1.46253	-1.97278	-1.05733	-1.41383	-2.00286	-2.84958	-1.07604	1.17058	-0.79782	2.08092	-0.80006
S20	0.36007	-1.20955	-1.26013	-0.56291	0.097	1.10724	0.22417	-0.08408	-0.9864	-1.24603	-0.12967	0.12506	1.02178	0.91461	-0.02027
S21	-1.59816	-0.60824	-0.44953	0.45564	1.28455	0.67995	0.7314	1.1499	-0.22224	-0.71191	0.60276	0.95875	-0.10414	0.73671	0.18933
S22	-0.54555	0.87222	0.1103	-0.96929	-2.02467	-1.91279	-2.04056	-1.49855	0.65031	0.51442	-0.52925	-0.85873	-1.81533	-1.72672	-0.80006
S23	-1.4583	0.17036	0.51119	0.11806	-1.18786	-1.34729	-1.30881	-1.45496	0.22823	0.54489	-0.14502	-0.23632	-1.60864	0.52575	-0.66001
S24	1.08046	1.64261	1.1662	0.05299	-0.66508	-0.68029	-0.70447	-0.51096	1.54419	2.33136	0.14637	-0.12592	-0.30244	-0.46652	-0.15254
S25	-0.72859	-1.24306	-0.48832	0.29768	0.05448	0.51087	-0.13158	-0.15388	-0.88845	-0.1487	1.40749	1.11342	0.52772	0.10484	-0.10166
S26	-0.39442	-1.36228	-1.05888	-0.38678	-0.20688	-0.12361	0.19014	-0.1748	-1.02872	-0.52916	0.83831	0.64163	0.43877	0.48433	0.11254
S27	1.06884	-1.10036	-0.45637	0.02412	-0.12824	0.01984	0.28499	-0.42398	-1.28039	-1.21807	-0.01498	-0.07505	1.59295	0.20894	-0.2211
S28	0.78266	-1.39602	-1.01929	-0.04902	0.45197	0.29057	0.38546	-0.21661	-1.40741	-0.94542	1.02288	1.3516	2.07926	2.10273	0.24947

S29	1.003	-1.0719	-0.46241	0.44584	0.90221	0.60154	0.76456	0.25171	-1.09722	-0.59637	0.74383	1.19147	2.77484	2.23297	0.5789
S30	1.20889	-0.89749	0.00813	0.53337	0.02111	0.11354	0.06298	-0.65204	-1.26102	-1.04115	0.34364	0.35625	1.01013	0.12229	-0.52667
S31	0.59779	-0.05798	1.57362	1.91519	0.14673	-0.68449	-0.65204	-0.76873	-0.92186	0.6967	1.69616	1.50256	0.20123	-0.38667	-0.43857
S32	-0.66732	-1.18632	-0.38262	0.60448	0.58565	0.35167	0.0508	-0.10666	-0.91735	-0.05306	1.97262	2.28104	0.96179	1.30531	0.18099
S33	0.80527	1.03532	1.48664	0.74216	-0.03018	0.54812	0.19161	0.02409	0.61686	1.58305	0.49845	0.35984	-0.19415	-0.36873	-0.16468
S34	1.36624	-0.1627	-0.25563	-0.64053	-0.95921	-0.75656	-0.76205	-0.97954	-0.25067	0.34131	-0.20251	-0.35819	0.1452	-0.91108	-0.61454
S35	-0.37221	-1.56903	-0.81869	0.13881	-0.34485	-0.9074	-0.93004	-1.26942	-1.58701	-0.95799	1.51475	1.43291	0.0955	0.14598	-0.76551
S36	-0.10403	-0.96351	-0.78816	-0.47674	-0.735	-0.73011	-0.68317	-1.08622	-1.03963	-0.00198	0.93575	0.75243	0.18415	-0.44299	-0.64803
S37	-0.44	0.69461	-0.15168	-0.98945	-1.42426	-1.25526	-1.26639	-0.70293	0.77899	0.59346	-0.41131	-0.61078	-0.82866	-1.19006	-0.4517
S38	0.51954	-0.58828	-0.84235	-0.97522	-0.7749	-0.26535	-0.06565	-0.50997	-0.64473	-0.26	-0.16694	-0.32636	0.45141	-0.58606	-0.31796
S39	2.32537	3.37945	1.93905	0.1545	-0.60192	-0.85253	-0.89154	0.09751	3.73228	3.15864	-0.28157	-0.44378	-0.91649	-0.70333	-0.19682
S40	-0.91551	-1.35787	-1.25712	-0.73753	-0.97447	-0.94637	-0.85848	-1.11416	-1.24609	-1.5183	0.01387	-0.19041	-1.14485	-0.75664	-0.80006
S41	0.28166	-1.45696	-1.59436	-1.28383	-0.83246	-0.2825	-0.16595	-0.807	-1.20176	-0.96306	-0.05547	-0.26579	0.70207	-0.72609	-0.72237
S42	-0.04731	-1.16746	-1.08344	-0.76774	-0.38986	0.06987	0.25448	-0.09709	-0.70304	-0.32982	0.41192	0.21316	0.792	-0.35657	-0.35468
S43	1.18732	1.99456	1.52697	0.53682	-0.05607	-0.48852	-0.65177	0.7485	1.66309	1.34067	-0.4688	-0.46025	-0.90051	-0.5796	-0.08365
S44	1.00223	1.95517	1.37933	0.20974	-0.52391	-0.79433	-0.78775	0.29595	1.17137	0.7188	-0.57082	-0.61552	-1.0752	-0.88267	-0.22071
S45	0.10854	1.58046	0.57147	-0.62142	-1.30259	-0.77918	-1.23458	-0.20325	1.44837	0.69671	-0.79659	-0.97719	-1.73407	-1.39569	-0.53405
S46	0.34534	-0.9884	-1.20024	-1.08668	-0.82641	-0.38018	-0.34133	-0.73739	-0.87754	-0.66672	-0.23123	-0.39254	0.42171	-0.80235	-0.66337
S47	-0.04049	-1.19972	-0.81772	-0.25163	-0.42232	-0.41023	-0.36759	-0.66648	-1.15118	-0.79595	0.58337	0.40883	0.05026	-0.21154	-0.56285
S48	0.76772	-1.09641	-0.84112	-0.42794	-0.15031	0.47916	0.14517	-0.25834	-1.01026	-0.66106	0.12121	0.10406	1.01719	0.20599	-0.18392
S49	-0.21913	-0.95022	-0.50833	0.2888	0.41874	0.44686	0.03053	0.09206	-0.72849	-0.51632	0.85248	0.94495	0.36421	0.79837	0.14835
S50	-0.63036	-0.19735	0.44006	0.71521	-0.12014	-0.67983	-0.64326	-0.66037	-0.09678	0.64517	1.70835	1.52379	-0.64422	-0.54998	-0.80006
S51	-0.06651	0.03693	1.47316	1.69635	0.49358	0.36453	0.40969	0.24224	-0.19355	1.59435	2.36288	2.1985	0.88755	0.19496	-0.19827
S52	0.04877	-0.82395	1.36397	2.38418	0.60513	-0.36987	-0.55816	-1.48958	-1.29847	-0.04716	2.83193	2.62803	1.04941	0.4871	-0.52139
S53	0.12107	-0.83742	-0.81956	-0.55198	-0.403	-0.07051	-0.05749	-0.69273	-0.76193	-0.17456	0.42796	0.27838	0.2944	-0.05516	-0.29802
S54	0.4812	0.29854	0.42306	0.12316	-0.44514	-0.44395	-0.40474	-0.60523	0.07309	0.97831	0.52856	0.29641	0.23248	-0.38146	-0.2065
S55	0.25892	0.28651	1.3185	1.3245	-0.10362	0.05309	-0.65064	-0.35678	0.01387	1.46606	1.95842	1.53223	-0.13181	-0.82234	-0.74892
S56	-0.28456	-0.11124	0.75925	0.92019	-0.471	-0.95699	-0.90384	-0.96441	-0.49653	0.41861	1.42713	1.07623	-0.14254	-0.74285	-0.69018
S57	0.23659	0.44312	1.50417	1.42423	0.1636	-0.20128	-0.24716	-0.34299	0.01901	1.34323	1.42581	1.1532	0.76802	0.16425	0.01914
S58	1.95743	2.18014	2.72662	1.462	0.31349	-0.01312	-0.23428	-0.0984	1.79946	2.89125	0.54948	0.50458	0.57956	0.16561	0.15532
S59	-0.4537	0.95574	0.588	-0.30316	-1.22616	-0.80137	-1.27701	-0.74561	0.76093	1.07334	-0.01288	-0.2444	-1.11729	-1.15525	-0.6111
S60	-0.6572	-1.14068	0.08287	-0.0855	-1.71581	-1.16096	-1.06588	-1.05187	-1.19101	1.06194	1.3575	0.58423	-0.35949	-1.72672	-0.80006
S61	0.26752	-0.44606	0.62741	1.1762	0.85675	0.30027	0.19151	-0.07209	-0.5183	0.98878	1.80157	1.95035	2.24158	1.87082	0.99439
S62	1.20547	-0.96475	0.18142	0.8167	0.58695	0.83756	0.89573	-0.10191	-1.22677	-0.97887	0.63002	0.49237	1.14278	0.10486	-0.49536
S63	0.67213	-0.19043	1.78613	2.38561	0.62467	0.51057	0.21036	-0.05433	-0.76221	0.35974	1.93313	1.48987	0.29054	-0.06569	-0.11307
S64	-0.21907	-0.62979	0.96946	1.18603	-0.42682	-0.07349	-0.7254	-1.24743	-1.07618	0.80444	1.87637	1.79892	1.40663	-0.17653	-0.53006
S65	1.40944	-0.33406	1.8237	3.40599	3.12152	1.95405	2.12863	0.89947	-0.96668	-1.02888	1.57159	2.09913	1.34863	2.3042	0.5849
S66	0.06725	0.0265	1.74432	1.58543	-0.11318	0.00362	0.16532	-0.62465	-0.73168	0.44903	0.90439	0.67534	-0.06226	-0.87475	-0.67681
S67	-0.12932	-0.45591	0.7519	1.67052	1.87727	1.84378	1.63521	0.81945	-0.72552	-0.59574	0.6324	0.62861	0.88631	1.14332	0.59519
S68	0.51875	-0.54467	0.29347	0.73167	0.30693	0.39137	0.62383	-0.33755	-0.9768	-0.55295	1.02317	0.76501	0.35771	0.03543	-0.50911
S69	-0.62101	0.30163	0.7605	0.57244	0.68058	0.62613	0.60554	0.66277	0.14223	0.34325	-0.25765	0.15748	-0.21731	0.3578	0.58586
S70	-0.90302	0.52819	-0.57251	-1.02359	-0.43282	-0.41203	-0.3371	0.00296	0.63801	-0.3438	-1.14814	-1.21587	-1.09065	-0.49436	-0.07129
S71	-0.64013	0.32244	-0.10911	-0.29211	0.30611	0.22422	0.34415	0.58824	0.49878	-0.04787	-0.68964	-0.71105	-0.32021	0.18116	0.25237
S72	-0.45947	-0.29882	-0.70099	-0.48495	0.74408	0.96648	1.17674	1.32771	0.08692	-0.53088	-0.72782	-0.93423	0.13276	0.39751	0.29872

S73	3.22724	0.37526	0.29206	0.4445	4.11819	5.96251	5.69781	3.36668	0.82803	-0.08185	-0.76169	-0.40849	3.09754	2.83376	2.22123
S74	-0.91715	0.2665	-0.59004	-1.07461	-0.66312	-0.24964	-0.15002	0.20706	0.30062	-0.35902	-0.94258	-1.25121	-1.14721	-0.87638	-0.16796
S75	-2.08882	-0.26697	-1.45851	-1.56522	-0.87263	-0.90653	-0.86677	-0.48741	-0.32601	-1.66986	-1.52461	-1.44044	-1.25818	-0.96892	-0.19626
S76	-1.95632	-0.46483	-0.79502	-0.45448	0.1138	-0.24317	-0.32824	-0.07594	-0.35528	-0.7507	-0.63126	-0.37479	-0.5353	-0.17385	0.86004
S77	-0.61947	0.70947	-0.35427	-0.79936	-0.06915	-0.09223	-0.00517	0.65576	0.86283	-0.20743	-0.91109	-1.10759	-0.52003	-0.09286	0.16758
S78	-0.51916	0.51892	0.13817	0.18042	1.27518	0.89834	0.81392	1.46447	0.80807	0.25818	-0.80381	-0.54737	0.66823	0.78129	1.1433
S79	-0.31223	0.4007	0.35516	-0.01599	-0.23949	-0.04483	0.03548	-0.05136	0.15948	0.15405	-0.551	-0.75322	-0.98153	-0.74093	-0.37782
S80	-1.18822	-0.20769	-0.51055	-0.4007	0.43545	0.72627	0.92794	0.5084	-0.21343	-0.73834	-0.7633	-0.52128	-0.30339	-0.07711	0.38327
S81	-0.02757	-0.409	-1.13673	-1.32347	-0.94332	-0.64838	-0.54846	-0.79836	-0.38293	-1.13726	-1.34677	-1.46692	-1.31688	-1.29611	-0.73233
S82	0.12159	0.58762	-0.04436	0.08097	1.99691	1.81326	2.11619	2.02332	0.83443	0.01318	-0.91682	-0.58237	1.24487	2.09633	1.4244
S83	-1.17335	-0.88401	-0.38008	0.32996	-0.10406	-0.47265	1.02878	0.72385	-0.35465	-1.30527	-0.87104	-0.29417	-0.33364	1.12873	0.03216
S84	-0.77806	-0.83841	-0.07885	0.8333	0.02866	-0.47999	0.95476	0.71059	-0.50182	-1.42319	-0.70088	-0.12408	-0.36291	0.42122	-0.58962
S85	-0.39476	0.25914	0.43419	0.91463	2.6607	3.07955	1.60888	1.69774	0.35812	-0.16464	-0.61486	-0.29546	0.6371	1.00861	0.54837
S86	-1.16822	0.08894	-0.8031	-1.0189	-0.22011	-0.54416	-0.14047	0.33901	0.28665	-0.29552	-0.62499	-0.82585	-0.74583	-0.22146	-0.04894
S87	-0.04578	0.78083	0.36463	0.13036	0.6208	0.34511	0.41317	1.39911	0.90002	0.29859	-0.76385	-0.70501	-0.19351	0.29589	0.51941
S88	0.7117	0.94764	0.62298	-0.06404	-0.28811	-0.27526	-0.27133	-0.04979	0.76835	0.58502	-0.74297	-0.90421	-0.8099	-0.87829	-0.38526
S89	-0.75141	-0.0416	-0.81972	-1.06272	-0.78373	-0.42143	-0.06392	-0.40751	-0.18724	-0.48541	-0.68809	-0.9704	-1.06802	-0.77137	-0.38606
S90	-0.89532	-0.19481	0.8438	1.72741	0.65758	-0.56851	0.36556	0.16049	-0.16581	-0.45412	-0.8071	0.28946	0.03168	0.77474	0.02295
S91	-0.914	0.04085	-0.37974	-0.52099	-0.29095	-0.32672	-0.53098	-0.44703	-0.33113	-0.89612	-0.97875	-1.04626	-1.1161	-0.39762	0.28017
S92	-0.21205	0.70402	-0.13766	-0.77822	-0.58439	-0.52238	-0.48988	-0.02886	0.62073	-0.26238	-1.11002	-1.16112	-0.48309	-0.82803	-0.16556
S93	0.95035	0.78554	0.69849	0.08484	0.41014	0.86792	0.8562	0.25938	0.5145	0.43589	-0.6842	-0.66705	-0.50055	-0.25452	0.19179
S94	-0.70451	0.35489	-0.48199	-0.32346	1.21407	0.94114	0.91034	1.55682	0.49616	-0.6098	-1.18192	-0.74961	0.21616	0.83273	0.96861
S95	-0.21003	0.71922	-0.37588	-0.88424	-0.00667	0.15619	0.287	0.53129	0.92344	-0.14337	-1.22554	-1.06714	-0.71109	-0.18388	1.1685
S96	-0.54368	0.51309	0.05065	-0.54726	-1.15195	-1.0266	-0.94117	-0.54318	0.23709	-0.10637	-0.89827	-1.14606	-1.86792	-1.70382	-0.80006
S97	-0.466	0.67531	-0.65293	-1.34476	-1.21181	-1.15522	-1.10338	-0.61434	0.63052	-0.61609	-1.35637	-1.47656	-1.55074	-1.29662	-0.6066
S98	0.35882	0.55648	-0.34206	-0.42543	1.077	1.0329	1.48407	2.54836	1.579	-0.20955	-1.08322	-0.97912	-0.09117	0.54075	0.99475
S99	0.07962	1.61229	0.13291	-0.79509	-0.77038	-0.90246	-0.99564	-0.00865	1.71321	0.4199	-1.16191	-1.23578	-1.3838	-0.86531	-0.22322
S100	-0.54031	0.22174	-0.59697	-0.77879	0.05352	0.00078	0.16468	0.64542	0.53876	-0.30435	-0.90627	-0.93203	-0.54336	0.14805	0.23321
S101	0.13609	0.58492	0.17969	0.05208	0.56844	0.48625	0.51457	0.87542	0.47842	0.04709	-0.77343	-0.69712	-0.44126	0.00531	0.19444
S102	-0.17241	0.46731	-0.63267	-1.00904	-0.1495	-0.18912	-0.06167	0.55301	0.86749	-0.38471	-1.24724	-1.02155	-0.82528	-0.27181	0.07247
S103	-0.82525	-0.74178	-0.76397	-0.0975	0.75998	0.46588	0.69614	0.92771	-0.42961	-1.37361	-0.55226	-0.69622	-0.18455	1.5598	8.43311
S104	-0.25829	0.86257	0.30721	-0.31111	-0.43187	-0.40007	-0.21736	-0.03613	0.63104	0.41707	-0.78976	-0.8501	-0.96021	-0.68399	-0.1778
S105	0.0811	0.13246	-0.35358	-0.64241	0.21176	0.64845	0.70827	0.33843	0.04499	-0.60173	-1.08345	-1.03036	-0.54189	-0.4589	-0.15511
S106	0.17148	1.07017	0.97546	0.61904	0.70422	0.37692	0.20593	0.6919	1.1795	1.19343	-0.54422	-0.53512	-0.49954	-0.02639	0.29741
S107	-0.3268	0.29274	-0.18826	0.09758	1.53565	1.24555	1.48365	1.63736	0.44025	-0.11603	-0.87843	-0.49759	1.70919	1.5134	2.07671
S108	0.34463	1.66518	-0.02527	-0.7966	0.05721	-0.25061	-0.40148	0.43101	1.96089	0.65128	-1.28714	-1.32538	-0.85097	-0.44763	-0.01813
S109	0.27838	0.81777	1.00768	0.58414	0.63212	0.49435	0.58369	0.57585	0.65263	0.88493	-0.32386	-0.22827	0.14914	0.14529	0.29378

Sample No.	integrated bins														
	3.10 .. 3.08	3.08 .. 3.06	3.06 .. 3.04	3.04 .. 3.02	3.02 .. 3.00	3.00 .. 2.98	2.98 .. 2.96	2.96 .. 2.94	2.94 .. 2.92	2.92 .. 2.90	2.90 .. 2.88	2.88 .. 2.86	2.86 .. 2.84	2.84 .. 2.82	2.82 .. 2.80
S1	0.56503	1.26569	1.45392	1.37422	1.39028	1.06401	1.43598	1.69198	0.37836	-0.00307	-0.27658	-0.18857	0.65494	-0.26444	-0.30027
S2	-1.1371	-1.44536	-1.34285	-1.15069	-0.97896	-0.91252	-0.81426	-0.47009	0.87519	0.59257	0.94165	0.5238	0.24941	1.04716	0.8118
S3	0.0721	0.36194	0.47939	0.01869	-0.21514	-0.48678	0.40862	1.08422	0.29894	0.84707	0.61632	0.91001	0.10513	0.72798	0.75714
S4	-0.64574	-0.74468	-0.77197	-0.66208	-0.85315	-1.11534	-1.23049	-0.93234	-0.8161	-0.81001	-0.74229	-0.77348	-0.73514	-0.73801	-0.78159

S5	0.60955	1.25072	1.36584	1.25566	0.97222	0.40806	1.2827	1.32569	0.24618	0.22611	0.07575	0.1889	0.38303	0.0138	0.11094
S6	-0.5894	-0.83382	-0.88761	-0.88226	-0.95851	-1.10461	-0.37355	0.20526	-0.17201	-0.35064	-0.16265	-0.30127	-0.77597	-0.08368	-0.26548
S7	-0.8181	-1.11157	-1.34285	-1.15069	-1.41002	-1.75763	-0.45524	0.10241	-0.13874	-0.23401	0.18723	-0.13091	-1.05504	-0.15767	0.15822
S8	0.09251	0.33515	0.46829	0.49601	0.49412	0.15408	0.04384	0.28166	0.33147	0.07263	0.17122	0.09533	0.26263	0.21318	0.06358
S9	-0.2894	0.09837	0.17848	-0.02891	0.0347	0.31459	0.25634	0.25678	0.1255	0.27693	-0.10863	0.15919	0.91083	0.18793	0.12167
S10	-0.57683	-0.7003	-0.22868	-0.09576	0.24127	0.15225	-0.00022	0.09897	2.90411	1.54247	2.27962	1.4695	0.98136	2.60571	1.67241
S11	-1.11877	-1.44536	-1.34285	-1.15069	-1.41002	-1.34614	-1.52612	-1.36782	-0.68481	-0.39863	-0.35823	-0.33152	0.12465	-0.16476	-0.25826
S12	-0.38736	-0.18238	0.099	0.11207	-0.11526	-0.41949	-0.42441	-0.55375	-0.00105	-0.24228	0.07102	-0.20238	0.04264	0.08534	-0.10442
S13	0.10968	0.55175	0.98855	0.92168	0.90561	0.39116	1.17158	1.03464	1.04353	0.38315	0.64761	0.59425	1.3752	0.93618	0.48146
S14	-0.44401	-0.40173	-0.6224	-0.64112	-0.65953	-0.70528	1.37364	1.75697	1.21385	0.57513	1.17517	0.67539	-0.24921	0.66771	1.08157
S15	1.32436	3.08062	3.75952	3.83611	3.55725	2.79407	2.38983	1.7619	1.14248	0.22809	0.06949	0.19172	4.27368	0.49177	0.19269
S16	-0.60882	-0.77753	-0.6515	-0.71788	-0.48387	-0.18637	-0.61163	-0.30242	1.11941	1.04058	1.3191	1.03769	-0.07692	1.42009	1.14601
S17	-0.24562	-0.18896	-0.22335	-0.7451	-0.63974	-0.11439	0.31471	0.9761	-0.16401	-0.42142	-0.57799	-0.48622	-1.05504	-0.52216	-0.60537
S18	-0.51009	-0.4411	-0.39117	-0.74423	-0.41914	0.42601	0.86579	0.92396	-0.26197	-0.88885	-0.98143	-0.91701	-0.16609	-0.80465	-0.91786
S19	-1.16288	-1.44536	-1.34285	-1.15069	-1.41002	-1.78463	-2.23629	0.01937	-0.368	1.04708	-0.45246	3.42767	-1.05504	-1.05348	0.91817
S20	-0.03887	0.30351	0.36016	0.25288	0.49889	0.53817	0.74125	1.10974	1.3886	1.29221	1.31669	1.37657	1.5003	1.14699	1.3602
S21	-0.09929	0.33714	0.80006	0.93847	1.08942	0.51063	0.90354	0.42374	2.02818	3.30382	2.53191	2.99885	5.71722	2.67431	3.12242
S22	-1.16288	-1.44536	-1.34285	-1.15069	-1.41002	-1.78463	-1.92842	-1.72238	-0.33318	-0.39275	0.02874	-0.38133	-1.05504	-0.11362	-0.14905
S23	-0.59357	-0.98048	-1.16111	-1.15069	-1.25458	-1.54049	-1.1857	-0.83093	-0.84074	0.99592	0.12357	1.10041	-0.58207	-0.49648	1.27894
S24	-0.49244	-0.59638	-0.47005	-0.3279	-0.45096	-0.88091	-0.74574	-0.55	0.10628	-0.0929	0.15283	-0.03423	-0.53119	0.05198	0.05543
S25	-0.02312	0.39353	0.96626	1.16257	1.73181	1.72549	1.59302	1.2476	1.8862	1.63544	1.55156	1.23053	2.11893	1.90506	1.33573
S26	0.03676	0.44959	0.9334	1.22427	1.64597	1.52403	1.00032	0.68317	1.33986	1.95657	1.65377	1.44418	1.6079	1.62324	1.6417
S27	-0.23673	-0.17693	-0.03256	0.03533	0.14776	-0.04915	-0.50448	-0.44355	1.05517	0.68012	0.94597	0.75918	0.26166	0.91026	0.83778
S28	0.16878	0.31839	0.28188	0.04827	0.51951	0.25522	-0.25029	-0.01323	1.29837	3.9654	3.46689	4.19364	1.97542	3.3478	3.89524
S29	0.62045	1.02536	0.9529	0.82046	1.24685	0.69369	0.27024	0.85772	1.09225	1.98882	1.60715	2.08281	0.79863	1.47274	1.83082
S30	-0.49144	-0.66466	-0.60505	-0.65645	-0.34661	0.14594	0.01584	0.47338	0.31462	0.15588	0.06041	-0.00273	-0.28031	0.10589	0.03612
S31	-0.50617	-0.55907	-0.37235	-0.32023	0.16519	0.31569	0.06644	-0.45823	0.45663	0.69509	0.8283	0.72582	0.01982	0.8593	0.80316
S32	-0.12637	0.05089	0.25242	0.17196	0.77133	1.1223	0.82587	1.15253	0.94904	1.07519	0.64994	0.74676	0.33502	0.29718	0.91381
S33	-0.43318	-0.27869	-0.00079	0.1144	0.06075	-0.34025	-0.73604	-0.7501	0.91093	0.56952	0.94329	0.6413	0.41753	1.03411	0.74671
S34	-0.75498	-0.9289	-0.80005	-0.80388	-0.77618	-1.08963	-1.08703	-0.81557	1.85543	0.86988	1.79566	0.95167	-0.32193	1.63387	1.29827
S35	-0.86686	-1.19757	-1.0388	-1.11729	-0.837	-0.73454	-0.89128	-0.44871	0.46377	2.93903	2.31724	2.36335	-0.40194	1.90606	2.68715
S36	-0.76474	-1.11199	-1.06984	-1.11297	-0.79275	-1.09079	-1.31203	-0.86436	0.94651	2.15388	2.09924	2.07691	-0.42091	2.11375	2.09897
S37	-0.57056	-0.73518	-0.64358	-0.57464	-0.78934	-0.72663	0.03239	-0.02696	-0.53955	-0.77695	-0.72685	-0.81564	-0.68699	-0.69992	-0.79416
S38	-0.40393	-0.34412	-0.10578	0.11367	0.30822	0.23233	0.69376	0.96776	0.81644	0.15106	0.40718	0.04738	0.67915	0.49206	0.18541
S39	-0.42597	-0.31024	-0.23458	-0.10056	-0.36776	-0.77508	-0.50614	-0.56681	-0.69303	-0.73613	-0.47195	-0.76665	-0.35	-0.4926	-0.66278
S40	-1.16288	-1.44536	-1.34285	-1.15069	-1.41002	-1.48057	-1.0925	-0.69721	-0.21048	0.38591	0.25703	0.1574	-0.49116	0.1458	0.41457
S41	-0.67021	-0.93168	-0.9176	-1.0305	-0.80502	-0.92536	-0.6257	-0.04651	1.03317	0.71592	1.0126	0.7557	-0.31933	1.13618	0.84176
S42	-0.29027	-0.24655	-0.02712	-0.09569	0.27771	0.25883	0.28748	0.43366	1.28839	0.48876	0.77844	0.53775	0.71125	0.99508	0.57978
S43	-0.34198	-0.03798	0.16163	0.27021	0.00026	-0.25732	-0.7706	-1.24563	-1.10876	-0.87906	-0.88309	-0.82854	-0.47434	-0.92362	-0.83524
S44	-0.50629	-0.35152	-0.16312	0.0149	-0.28477	-0.33624	-0.65871	-1.05136	-1.11306	-1.03409	-1.00512	-0.96319	-0.57043	-0.94241	-1.02117
S45	-0.76509	-0.71724	-0.45796	-0.34982	-0.59104	-0.71634	-1.20058	-1.36722	-1.30505	-1.08985	-1.03504	-0.973	-0.44984	-0.91376	-1.01407
S46	-0.68151	-0.90175	-0.85508	-0.93287	-0.69	-0.78933	-0.65463	-0.16365	0.88441	0.4397	0.8014	0.52416	-0.31498	0.87978	0.61107
S47	-0.56002	-0.68124	-0.55171	-0.66821	-0.38778	-0.37306	-0.26247	0.09758	0.53917	0.97972	0.86563	0.80316	-0.08637	0.89886	0.88549
S48	-0.20904	-0.10272	0.0865	0.01137	0.21224	0.11426	0.24507	0.68782	1.01758	0.67552	0.8162	0.73464	0.26855	0.90875	0.70858

S49	0.07537	0.56222	0.98946	1.05831	1.32555	1.15346	0.92407	0.84903	0.717	1.14267	0.78705	0.9186	1.47171	0.85752	0.8913
S50	-0.95055	-1.44536	-1.34285	-1.15069	-1.41002	-1.78463	-0.63876	0.39313	-1.08134	-1.07053	-1.04209	-1.0167	-1.05504	-1.05348	-0.96663
S51	-0.07608	0.24096	0.24836	-0.01931	0.08243	0.38855	1.06438	1.13619	0.41488	-0.08139	-0.14824	-0.10693	0.43389	0.10103	-0.13804
S52	-0.6982	-1.09017	-1.32268	-1.15069	-1.41002	-1.50613	1.2022	2.16127	-0.20501	0.12143	-0.12694	-0.04225	-1.05504	-0.20266	-0.02532
S53	-0.43376	-0.40967	-0.33239	-0.45937	-0.44559	-0.45689	0.19964	0.86339	-0.54145	-0.62193	-0.66986	-0.63758	-0.48788	-0.58516	-0.66066
S54	-0.40275	-0.39509	-0.33945	-0.34946	-0.24847	-0.38312	0.05882	0.40128	0.39507	0.31083	0.44541	0.30216	-0.16834	0.45003	0.38082
S55	-0.70337	-0.97157	-0.95156	-1.0311	-1.0196	-0.30388	0.95036	1.12028	-0.37343	-0.6316	-0.60243	-0.60534	-0.96989	-0.45102	-0.61454
S56	-0.83069	-1.0445	-0.93091	-0.94792	-0.7442	-0.24486	0.52379	0.8303	-0.05605	-0.03305	-0.13132	-0.16938	-0.36791	-0.04582	-0.09089
S57	-0.18441	0.06112	0.23555	0.21145	0.25132	0.40658	0.85528	0.96067	-0.12051	-0.38036	-0.46263	-0.46961	-0.11932	-0.364	-0.49681
S58	-0.055	0.24062	0.38013	0.41769	0.19291	-0.01868	0.67847	0.92872	-0.16191	-0.44717	-0.27811	-0.39652	-0.04012	-0.23219	-0.39087
S59	-0.79398	-1.05762	-1.03325	-1.0179	-1.00446	-0.81899	-0.56789	-0.463	-0.93638	-0.8801	-0.80704	-0.84683	-1.05504	-0.74148	-0.83075
S60	-0.86982	-1.00599	-0.47284	-0.08433	0.62504	0.81132	2.09467	2.67437	4.46971	2.08863	3.11704	1.33043	0.63081	3.45838	2.00215
S61	0.93541	1.51265	1.53769	1.52158	1.6027	1.04797	2.06348	3.12835	1.39172	1.39934	1.14746	1.25409	0.99998	1.03901	1.18548
S62	-0.50292	-0.61663	-0.67958	-0.70226	-0.72719	-0.87431	-0.26036	0.08792	0.17391	0.14848	0.25713	0.1499	-0.09307	0.18665	0.30264
S63	-0.11268	0.27935	0.54302	0.7015	1.16762	1.66667	1.98686	1.51653	0.02287	-0.50629	-0.6859	-0.65712	0.88473	-0.46745	-0.66236
S64	-0.47481	-0.7068	-0.96992	-1.15069	-0.90255	-0.35951	1.50395	2.51714	0.63141	0.30813	0.21189	0.13195	-1.05504	0.17159	0.13557
S65	0.27346	0.81441	0.91407	0.85882	0.78125	1.05517	1.54037	1.30743	0.01253	-0.64558	-0.80463	-0.78375	0.03113	-0.74296	-0.8205
S66	-0.60494	-0.79101	-0.96012	-0.92608	-0.80064	-0.43171	0.20067	0.53744	-0.64083	-0.72039	-0.73489	-0.74374	-0.58112	-0.58926	-0.70184
S67	0.4596	1.08043	1.0969	1.02067	1.22878	2.23575	2.68215	1.30628	-0.3676	-0.7962	-0.93602	-0.91854	0.00767	-0.81716	-0.9789
S68	-0.53462	-0.59392	-0.44459	-0.50238	-0.31959	-0.23098	-0.77429	-0.75867	-0.1106	0.04152	-0.02343	-0.02256	-0.05488	0.09726	-0.01993
S69	1.45404	0.84374	0.77947	0.63513	0.48931	1.27534	1.66269	0.68359	-0.44806	-0.79831	-0.87132	-0.83948	-0.06202	-0.81009	-0.90586
S70	0.1283	-0.02999	-0.12016	0.08426	-0.02006	0.02057	-0.29293	-0.78879	-0.90287	-0.89413	-0.89328	-0.87018	-0.37133	-0.84297	-0.92766
S71	0.33809	0.66513	0.35086	0.3121	0.16483	0.1412	-0.10391	-0.36086	-0.74784	-0.80706	-0.84308	-0.82138	-0.3323	-0.81213	-0.90379
S72	1.11031	0.69417	0.29772	0.01944	0.35977	0.69039	0.18681	-0.11699	0.25797	0.41949	0.33461	0.40434	-0.33473	0.27738	0.38051
S73	1.6012	2.5639	2.9661	3.23047	2.76251	1.79422	0.84211	0.31015	-0.11727	-0.56576	-0.63731	-0.63154	1.50999	-0.57645	-0.7677
S74	0.43736	-0.52538	-0.46637	-0.29033	-0.25525	0.01281	-0.06043	-0.38793	-0.49629	-0.55801	-0.54006	-0.63908	-0.1509	-0.45077	-0.59349
S75	0.41312	-0.10167	-0.49006	-0.3251	-0.49721	-0.78564	-1.27349	-1.46931	-1.30812	-1.07249	-1.06294	-1.01909	-0.71218	-0.98409	-1.09301
S76	1.47872	0.30625	0.00465	-0.2189	-0.39407	-0.22948	-0.54938	-0.88903	-0.96711	-0.13765	-0.39433	-0.27369	-0.76573	-0.8045	-0.11801
S77	0.41262	0.63373	0.45192	0.4877	0.32744	-0.02244	-0.51613	-0.82384	-0.90864	-0.90111	-0.92735	-0.89886	-0.09807	-0.84697	-0.98293
S78	2.79047	1.88479	1.92785	2.15345	1.86508	1.56041	0.69475	-0.26789	-0.59337	-0.77812	-0.84708	-0.78451	0.63719	-0.76928	-0.91694
S79	-0.60203	-0.67939	-0.5936	-0.74434	-0.78265	-0.17356	-0.51092	-0.88158	-1.09007	-0.92474	-0.92153	-0.91847	-1.04264	-0.8839	-0.93904
S80	0.85599	0.57514	0.23254	0.04483	0.2341	1.01201	0.60531	-0.10739	-0.6202	-0.34751	-0.53607	-0.4409	-0.47256	-0.78503	-0.40928
S81	-0.25688	-0.95154	-1.34285	-1.15069	-1.39154	-1.28601	-1.52687	-1.42121	-1.20141	-0.84689	-0.73648	-0.71652	-1.05504	-0.79685	-0.67746
S82	2.04607	2.74963	2.8622	2.7043	2.45662	2.58889	1.07538	0.13713	-0.43477	-0.36961	-0.57737	-0.348	0.85965	-0.84985	-0.46217
S83	-0.31453	1.17857	0.71499	0.31018	0.24619	-0.07881	-0.33998	-0.40552	-0.37929	-0.55543	-0.8093	-0.41894	0.50814	-0.74769	-0.70793
S84	0.12379	0.03202	-1.01045	-1.15069	-1.26506	-0.60031	-0.45014	-0.34717	-0.48611	-0.86136	-1.00458	-0.80844	-1.05504	-1.05348	-1.00423
S85	0.8547	3.1466	1.58669	1.73044	1.30994	0.75831	0.01089	-0.35573	-0.77195	-0.82938	-0.93008	-0.88574	-0.3863	-0.96706	-0.98835
S86	0.1183	-0.09899	-0.31492	-0.34531	-0.3528	-0.35563	-0.82183	-0.7645	-0.09494	0.4665	0.41423	0.3319	-0.44958	0.13519	0.53926
S87	0.4858	2.03458	1.56742	1.87256	1.72611	1.41399	1.00387	0.04125	-0.55335	-0.78227	-0.84877	-0.78866	0.84711	-0.72837	-0.90364
S88	-0.49061	-0.40728	-0.89289	-0.66643	-0.97055	-1.06284	-1.49186	-1.54162	-1.19442	-0.92781	-0.83665	-0.85192	-1.05504	-0.75863	-0.87032
S89	-0.71105	-0.85083	-0.66941	-0.73263	-0.46177	-0.04797	-0.39187	-0.16054	-0.67515	-0.40229	-0.45072	-0.44903	-0.65457	-0.51078	-0.32968
S90	-0.41741	0.0136	-0.02414	-0.06432	-0.10038	0.42322	0.07767	-0.8047	-0.76961	0.05807	-0.82353	0.83018	-0.05594	-0.9385	-0.2362
S91	6.05956	1.41846	-0.03206	-0.76696	-1.10277	-0.78105	-0.89455	-1.27804	-1.53512	-1.12614	-1.1148	-1.06212	-1.05504	-1.05348	-1.05232
S92	0.55473	-0.12137	-0.25251	-0.11858	-0.01019	0.45004	-0.60642	-1.15887	-1.24153	-1.06922	-1.04332	-0.99536	-0.50655	-0.95497	-1.06405

S93	-0.15426	0.29477	0.16595	0.30385	0.41575	1.17139	0.03769	-0.66637	-0.92721	-0.95015	-0.96679	-0.9176	-0.37893	-0.90199	-1.01594
S94	0.58584	1.27109	1.77087	1.7196	1.50628	1.60877	0.73568	0.14653	-0.41295	-0.84976	-0.93344	-0.90229	0.03016	-0.89819	-1.04991
S95	0.91912	0.28092	0.51779	0.6683	0.26211	-0.12722	-0.76249	-1.07201	-0.98793	-0.93932	-0.93259	-0.88317	-0.02312	-0.84562	-0.9625
S96	-1.13922	-1.44536	-1.34285	-1.15069	-1.41002	-1.66084	-1.98502	-2.06431	-1.69647	-1.22557	-1.14091	-1.08115	-1.05504	-1.05348	-1.14872
S97	-0.54971	-1.07945	-1.16618	-1.14467	-1.41002	-1.49355	-1.95236	-2.02332	-1.6418	-1.22557	-1.14091	-1.08115	-1.05504	-1.05348	-1.1498
S98	0.50681	1.19815	1.68459	1.90241	1.90824	1.72838	0.76894	-0.1907	0.65901	-0.29129	-0.01118	-0.249	1.88922	0.12808	-0.19304
S99	-0.48168	-0.23504	-0.28476	-0.17013	-0.46745	-0.87693	-1.44888	-1.64749	-1.37359	-1.09896	-1.06269	-1.00894	-0.62819	-0.9717	-1.09048
S100	1.03482	0.93538	0.3991	0.25601	0.26214	0.55413	0.49507	0.15168	-0.47723	-0.45388	-0.44046	-0.44943	-0.05156	-0.51328	-0.41083
S101	0.01729	0.27311	0.31159	0.29525	0.15487	0.25871	-0.10925	-0.76153	-0.97609	-0.91965	-0.94279	-0.90598	-0.58772	-0.93265	-0.98349
S102	0.21259	0.09898	0.12788	0.30314	0.22914	0.02397	-0.74649	-1.10581	-0.628	-0.64286	-0.52546	-0.56616	-0.05175	-0.54086	-0.55422
S103	2.82891	0.91615	1.08923	0.62984	0.63596	1.13481	0.74708	0.33029	-0.59061	-0.81598	-0.87794	-0.83482	-0.28247	-0.82335	-0.86228
S104	-0.49963	-0.46922	-0.26572	-0.37135	-0.47457	-0.27989	-0.72256	-0.93017	-0.89373	-0.74783	-0.72175	-0.70867	-0.58318	-0.7242	-0.69966
S105	0.9884	0.22296	-0.35276	-0.42309	-0.52057	0.14591	-0.15814	-0.59203	-0.96757	-0.76094	-0.73238	-0.68776	-1.05504	-0.73493	-0.76457
S106	0.30501	0.90878	0.88227	1.06293	0.74173	0.67224	-0.03545	-0.81984	-0.99824	-0.96268	-0.98208	-0.92154	-0.1854	-0.90067	-1.03818
S107	3.23791	2.29506	2.31317	2.16902	2.00208	2.28409	1.50554	0.3813	-0.39388	-0.59859	-0.74378	-0.66579	0.20839	-0.88352	-0.79065
S108	0.88306	0.45234	0.17844	0.32075	0.09963	-0.11725	-0.36296	-0.80092	-1.12247	-1.00082	-0.98677	-0.93983	-0.28282	-0.91778	-1.02109
S109	0.25575	0.94829	0.67069	0.60442	0.53551	1.00134	0.78654	0.51377	-0.24835	0.36215	0.285	0.38217	0.43786	-0.21178	0.55311

Sample No.	integrated bins														
	2.80 .. 2.78	2.78 .. 2.76	2.76 .. 2.74	2.74 .. 2.72	2.72 .. 2.70	2.70 .. 2.68	2.68 .. 2.66	2.66 .. 2.64	2.64 .. 2.62	2.62 .. 2.60	2.60 .. 2.58	2.58 .. 2.56	2.56 .. 2.54	2.54 .. 2.52	2.52 .. 2.50
S1	-0.06197	0.94394	1.61363	1.68155	0.55947	0.57425	0.5496	0.08702	1.08286	1.1046	1.60142	0.6787	0.48147	0.82614	0.62819
S2	0.91856	0.69086	0.64533	0.94309	1.47212	0.49023	-0.24883	-0.80341	-0.78367	-0.31026	1.19304	2.33764	1.14956	-0.10428	-0.57806
S3	0.65122	0.73515	0.13322	0.21612	0.38404	0.94203	1.11292	0.75115	0.76777	0.11978	0.49927	0.39668	0.78244	1.62995	1.23623
S4	-0.93772	-1.05429	-0.94849	-0.74831	-0.49836	-0.76685	-0.96325	-1.24289	-1.39672	-1.54094	-0.88396	-0.52038	-0.50324	-0.77051	-0.99101
S5	0.11273	0.05212	-0.12161	-0.12842	-0.35813	-0.67043	-0.84088	-1.10543	-0.70517	-0.84429	0.03097	-0.18457	-0.15173	-0.59237	-0.83809
S6	-0.37891	-0.57054	-0.73419	-0.69031	-0.55524	-0.3042	0.07497	-0.05109	0.10283	-0.76049	-0.45399	-0.55761	-0.4464	0.57117	-0.28378
S7	0.00699	-0.28876	-0.31521	-0.76593	-0.9025	-1.25963	-1.09061	-1.2975	-0.85749	-1.38382	-0.75565	-0.82691	-0.75912	-0.70985	-1.18113
S8	0.08056	-0.05683	-0.15614	-0.11525	0.14051	0.13377	0.02085	-0.31986	-0.09128	-0.25475	0.09775	0.27101	0.52931	0.32288	0.00716
S9	0.41084	1.27789	2.66384	3.70059	5.32356	4.37358	3.15501	1.56247	1.18561	1.17465	2.67052	4.04783	4.10473	3.52148	3.21352
S10	1.79161	1.31932	0.52992	0.66881	1.68913	2.0402	1.72484	0.87049	0.1169	-0.02314	0.77805	2.48605	3.11673	1.13652	0.53357
S11	-0.14914	-0.19797	0.32296	0.85946	2.27918	2.03929	1.52602	0.33445	-0.09767	-0.29342	0.92949	1.83147	1.93315	1.70113	1.4652
S12	-0.03817	-0.21757	-0.16264	-0.20194	0.17098	0.44308	0.42843	0.12185	0.39279	0.03072	0.13433	0.03277	-0.01015	0.65048	0.54813
S13	1.11698	1.1323	2.1002	1.72209	1.88607	1.38004	0.7933	0.80712	0.5592	0.94968	1.46937	1.98848	1.95184	0.75375	0.32347
S14	0.72221	0.18517	-0.7161	-0.87954	-0.85929	-1.31709	-1.24539	-1.50769	-1.16708	-1.73386	-0.94413	-0.8677	-0.88887	-1.04376	-1.39974
S15	1.29888	1.44755	3.78395	3.27759	3.65239	2.11312	1.00876	0.13616	0.40037	0.55636	3.51414	5.00003	2.65714	0.98275	0.29093
S16	1.01569	0.70376	-0.1832	-0.02588	0.63332	0.84974	0.63179	0.04849	-0.47922	-0.78414	-0.28652	-0.00537	0.07352	0.69866	0.55163
S17	-0.35451	1.70809	2.94565	3.23384	1.69777	1.97563	1.78485	1.0625	2.93657	2.14267	2.85267	1.66809	2.06883	2.18981	1.95978
S18	-0.26338	2.69547	4.78601	4.69512	2.38332	2.13372	1.61806	0.8777	3.40549	2.43294	3.60281	2.3682	2.5251	1.64901	1.24121
S19	2.39884	1.72958	0.34167	0.75325	0.17818	0.48338	0.92839	0.50701	1.1099	-0.1462	1.45154	0.73643	0.46461	1.3263	1.12321
S20	1.58845	1.20694	0.68644	0.73434	1.32591	1.35365	1.10638	0.39202	0.22217	0.14478	0.8775	1.55498	1.88925	1.09782	0.78289
S21	3.0057	2.34433	1.17748	1.11458	1.39103	0.29871	-0.33037	-0.44772	-0.09413	1.00386	1.4553	2.0307	0.76771	-0.45173	-0.86771
S22	-0.48206	-0.70109	-1.14727	-1.05281	-0.96444	-1.02499	0.25097	-0.43288	0.42649	-1.5338	-1.02127	-0.88795	-0.80727	0.09788	0.16809
S23	0.369	1.46468	-0.23962	2.0527	-0.27638	-0.31028	-0.19509	-0.44979	0.46284	0.15432	0.7719	-0.21576	-0.29691	0.04876	-0.10329
S24	-0.09417	-0.30551	-0.5974	-0.57487	-0.54156	-0.49451	0.06262	-0.21218	0.2009	-0.84951	-0.21341	-0.42352	-0.40767	0.46488	-0.03359

S25	1.40318	1.17721	0.74972	0.96108	1.76744	1.40359	0.89494	-0.02362	-0.1854	-0.24836	0.79353	1.69497	1.71208	0.73629	0.31368
S26	1.40765	1.28476	0.4468	0.44623	0.59724	0.53725	0.28583	-0.17948	-0.00325	-0.17569	0.33457	0.36044	0.33782	0.44007	0.06207
S27	0.7565	0.44789	-0.1638	-0.09318	0.29027	0.14029	-0.17045	-0.56129	-0.85116	-0.78324	-0.29026	0.24121	0.55177	-0.08213	-0.38822
S28	3.96806	3.42893	0.97872	0.62546	0.54855	0.43755	0.22956	-0.18755	-0.03097	-0.15742	0.37452	0.42204	0.52752	0.38716	0.14815
S29	1.77296	1.50199	0.12018	0.02535	0.0313	0.24117	0.41174	0.16516	0.11135	-0.48472	0.11881	0.07573	0.06408	0.79722	0.42518
S30	-0.05032	-0.10898	-0.13031	0.22762	1.05597	0.88536	0.53946	-0.12844	-0.2134	-0.06701	0.18831	0.65876	0.8094	0.6618	0.42911
S31	0.92142	1.07707	0.88611	0.65972	0.0646	0.25398	0.42136	0.10852	0.64783	0.24809	0.67969	0.41491	0.91721	0.7837	0.32126
S32	0.59662	0.36702	-0.37236	-0.47778	-0.4469	-0.27299	0.58758	0.2437	0.78354	-0.49432	-0.06676	-0.39898	-0.53025	0.28791	0.48693
S33	0.92903	0.7722	0.62694	0.47374	0.49407	0.1913	-0.15083	-0.54332	0.09963	0.3554	0.41582	0.34131	0.35887	0.01955	-0.21235
S34	1.20769	0.74868	-0.30785	-0.46683	-0.44941	-0.62245	-0.7696	-1.01374	-1.01011	-1.18163	-0.63946	-0.36431	-0.16523	-0.50377	-0.87053
S35	1.99499	1.88395	0.10707	0.22373	0.4166	0.80527	0.69434	0.24656	0.06147	-0.26988	0.00163	0.01406	0.1865	0.92278	0.55704
S36	1.86811	1.65718	0.08557	0.11076	0.24109	0.75627	1.08722	0.69087	0.59639	-0.24657	0.32193	0.34999	0.759	1.64802	1.10743
S37	-0.87439	-1.02379	-0.83781	-0.85329	-0.84045	-1.32587	-0.80255	-0.91045	0.32234	-1.01646	-0.60483	-0.64589	-0.60861	0.26245	-0.93757
S38	0.13175	-0.14981	-0.38892	-0.36258	-0.11779	-0.18426	-0.2669	-0.50158	-0.51314	-0.83621	-0.41763	-0.12254	0.05841	0.00353	-0.55404
S39	-0.70645	-0.85969	-0.61229	-0.8221	-0.80768	-1.15062	-1.24991	-1.37937	-0.94368	-1.1192	-0.73603	-0.69018	-0.62881	-0.87362	-1.30037
S40	0.08946	0.14892	-0.06283	0.06022	0.3373	0.23994	0.04791	-0.48461	-0.66459	-0.77532	-0.2104	0.25114	0.61146	0.08621	-0.31085
S41	0.75054	0.51252	-0.12124	-0.13338	0.07627	0.18726	0.03015	-0.31829	-0.56602	-0.73068	-0.29101	0.2621	0.89985	0.05633	-0.26595
S42	0.72407	0.53297	0.47993	0.36317	0.78795	0.87738	0.63919	0.06143	-0.23076	-0.44756	0.22992	0.96604	1.57	0.47409	0.1293
S43	-0.98546	-1.05538	-0.8022	-0.76849	-0.82177	-0.56772	-0.85592	-0.13912	-1.20187	-0.80327	-0.97929	-0.72887	-0.89122	-0.98488	-0.51303
S44	-1.18652	-1.27299	-1.07295	-0.9445	-0.86931	-0.72309	-0.64484	-0.25417	-0.94225	-0.9284	-1.14279	-0.84502	-0.91069	-1.27521	-0.00139
S45	-0.82393	-0.91681	0.0505	-0.39559	-0.26579	-0.09678	-0.4493	-0.58737	-1.07154	-1.38963	-0.69446	-0.56638	-0.57668	-0.34655	-0.49728
S46	0.64076	0.45503	0.12317	0.03074	0.1504	0.19949	0.01212	-0.35573	-0.43683	-0.58157	-0.10778	0.33285	0.88957	0.06304	-0.262
S47	0.74111	0.76599	0.22815	0.26542	0.42905	0.75901	0.68806	0.24598	0.12464	-0.2285	0.16183	0.38146	0.92039	0.93821	0.47416
S48	0.7809	0.68177	0.35581	0.31474	0.47195	0.6995	0.58368	0.14361	0.09057	-0.17571	0.24628	0.4846	0.9689	0.78913	0.42466
S49	0.90413	1.02681	0.71964	0.77196	0.97786	1.30613	1.23948	0.65684	0.58153	0.12211	0.74422	0.89727	1.26554	1.46082	1.00746
S50	-0.82414	-0.15297	0.67203	0.31186	-0.80974	-1.06832	-0.56999	-0.75308	0.58991	-0.2304	0.48592	-0.51833	-0.6222	-0.30559	-0.42988
S51	-0.11125	0.03249	0.28147	0.34663	0.6263	1.04889	0.90437	0.43778	0.13146	-0.33391	0.17326	0.37805	0.8478	1.16753	0.64574
S52	-0.19561	0.79596	0.71391	0.6627	-0.37196	0.22815	0.88922	0.70422	1.93776	0.84656	1.10604	-0.30556	-0.34065	1.05679	1.02919
S53	-0.31285	0.8115	1.9124	1.48256	-0.19279	-0.11327	0.34983	0.11617	1.26743	0.78073	1.0186	-0.05549	-0.11357	0.77065	0.19283
S54	0.31053	0.17108	-0.16174	-0.20813	-0.2632	-0.28449	-0.21835	-0.47216	-0.1737	-0.63995	-0.02458	-0.17212	-0.01138	0.16754	-0.31225
S55	-0.15806	-0.11732	1.26376	0.76581	1.26903	1.28674	0.90491	0.37402	-0.18159	-0.7169	0.42082	1.09204	1.91516	0.54417	0.21431
S56	-0.01943	0.24621	0.59197	0.59807	0.83046	1.04042	0.88237	0.26645	0.4255	0.14013	0.50215	0.3879	0.54493	1.03807	0.70495
S57	-0.16332	0.51326	1.36864	1.04667	0.12815	0.60773	0.93332	0.70299	1.35752	0.5466	0.89837	0.05194	0.00668	1.23114	0.82622
S58	-0.1033	-0.13818	0.50965	0.07114	-0.22616	-0.40291	-0.57205	-0.81114	-0.10145	-0.10157	0.128	-0.17549	-0.19018	-0.25489	-0.56144
S59	-0.50361	-0.50758	0.53338	-0.01886	-0.38004	-0.59978	-0.76152	-1.05272	-0.58774	-0.60314	-0.11884	-0.41547	-0.41655	-0.55858	-0.75032
S60	1.79589	1.17103	-0.13056	-0.09727	0.35935	0.39697	0.10227	-0.40043	-0.57538	-0.81947	-0.31918	0.11194	0.43334	0.20972	-0.33851
S61	1.2663	1.35484	0.76737	0.51077	-0.30434	-0.51347	0.17341	-0.18063	0.77472	-0.14963	0.42146	-0.1308	-0.44908	0.2928	0.09845
S62	0.15839	0.02627	-0.13119	-0.23909	-0.33918	-0.66123	-0.96242	-1.18772	-1.17895	-1.00356	-0.62524	-0.36324	-0.18689	-0.84019	-1.14808
S63	-0.22373	0.89216	2.1101	1.50633	0.33953	0.67928	0.74736	0.56328	1.48005	0.91054	0.99808	0.00178	0.00292	0.93833	0.23952
S64	0.09339	0.37427	0.22262	0.13149	-0.34882	0.07455	0.33376	0.12216	0.47098	-0.20559	0.06684	-0.40431	-0.32241	0.59625	0.23356
S65	-0.87539	-0.28432	0.15455	0.27791	-0.07753	0.1903	0.22433	0.19742	0.27344	0.06253	0.06341	-0.24206	-0.41079	0.1465	0.21466
S66	-0.55278	-0.27105	0.42531	0.29094	-0.00383	0.17413	0.01384	-0.21722	0.04083	-0.21342	-0.01897	-0.22238	-0.02067	0.3402	-0.20181
S67	-0.80108	0.07192	0.95486	0.86546	0.21948	0.76926	0.70525	0.65928	0.82263	0.13553	0.55932	-0.01892	-0.2627	0.54348	0.89557
S68	-0.06052	0.01798	0.03384	0.252	0.788	0.91161	0.661	0.38655	0.16358	0.58032	0.09893	0.35896	0.72712	0.84972	0.43939

Sample No.	integrated bins 2.50 .. 2.48	2.48 .. 2.46	2.46 .. 2.44	2.44 .. 2.42	2.42 .. 2.40	2.40 .. 2.38	2.38 .. 2.36	2.36 .. 2.34	2.34 .. 2.32	2.32 .. 2.30	2.30 .. 2.28	2.28 .. 2.26	2.26 .. 2.24	2.24 .. 2.22	2.22 .. 2.20
S69	-0.64656	-0.67059	0.04645	-0.19706	-0.44623	-0.5661	-0.92426	0.0385	-0.4411	1.2565	-0.39752	-0.51152	-0.66775	-0.76757	-1.05463
S70	-0.93778	-1.05686	-0.77461	-0.75461	-0.67963	-0.97744	-1.30209	-1.01279	-1.17183	-0.48321	-1.00069	-0.78449	-0.80682	-1.10982	-1.28373
S71	-0.82726	-0.93411	-0.64231	-0.69143	-0.70282	-0.83498	-1.10411	-0.56341	-0.55338	0.45073	-0.67382	-0.69838	-0.76465	-0.84722	-1.00389
S72	0.49468	0.27424	-0.12473	-0.21358	-0.23155	-0.24731	-0.53598	-0.01261	0.25561	1.61693	0.06422	-0.27207	-0.29245	-0.15327	-0.54501
S73	-0.78243	-0.91101	-0.68206	-0.67222	-0.66354	-1.39527	-1.79979	-1.54091	-1.83978	-1.19885	-0.96369	-0.62482	-0.80721	-1.60266	-1.841
S74	-0.3931	-0.50161	-0.06799	-0.30852	-0.38792	-0.71707	-1.08494	-0.58857	-1.06783	-0.29779	-0.89154	-0.73669	-0.7197	-1.06865	-1.1658
S75	-1.14365	-1.32496	-0.96225	-1.00323	-0.97338	-1.30645	-1.56826	-1.20781	-1.29759	-0.64585	-1.21515	-0.96175	-0.98746	-1.3997	-1.40061
S76	-0.40429	-0.02221	0.03615	-0.06686	-0.47612	0.2581	0.06841	1.79608	0.33477	1.79199	-0.25227	-0.47574	-0.71004	-0.29505	0.65538
S77	-0.9739	-1.09895	-0.88487	-0.85151	-0.83018	-1.19568	-1.35787	-1.23888	-1.15543	-0.91041	-1.04384	-0.83809	-0.8799	-1.14932	-1.36142
S78	-0.91492	-0.95774	-0.5381	-0.57492	-0.68721	-0.64291	-0.66321	0.74191	0.02652	1.88417	-0.43638	-0.45785	-0.77345	-0.86919	-0.31504
S79	-0.96795	-1.02819	-0.86253	-0.8223	-0.79222	-1.10906	-1.42145	-0.49536	-1.15049	0.41594	-1.0836	-0.89753	-0.87107	-1.32532	-1.32903
S80	-0.63139	-0.68748	-0.60146	-0.68875	-0.81692	-0.85051	-0.98683	0.0731	-0.67206	0.74613	-0.96372	-0.83238	-0.94628	-0.99799	-0.97854
S81	-0.91743	-1.06463	-1.06958	-0.94016	-0.79945	-0.41989	-0.25719	0.22214	0.12141	0.22514	-0.82376	-0.86131	-0.85983	-0.44688	0.00757
S82	-0.67077	-0.73585	-0.61823	-0.7619	-1.06235	-1.28431	-1.19576	-0.21683	-0.35708	1.23297	-0.21167	-0.43057	-0.91205	-1.3959	-0.44447
S83	-0.43132	-0.61862	-0.18864	-0.34212	-0.35294	-0.75907	-0.71394	-0.94769	1.32036	-0.47334	3.23398	-0.46209	-0.72231	-1.14528	-0.40599
S84	-0.835	-0.94325	-0.58057	-0.38848	-0.48487	-0.63034	-0.8605	-0.8343	1.89489	1.08518	2.20825	-0.90009	-0.85454	-1.08537	-0.83721
S85	-1.16005	-1.14669	-0.92684	-0.77893	-1.00777	-1.18746	-1.00655	-0.4651	-0.04187	0.65954	-0.48735	-0.66248	-0.88978	-1.56559	-0.49162
S86	0.23109	-0.00495	-0.65708	-0.72662	-0.89703	-0.94536	0.04858	0.41638	-0.21866	-0.22555	-1.01836	-0.89472	-0.94753	-0.81825	0.21511
S87	-0.87779	-0.92128	-0.44767	-0.45912	-0.40602	-0.38344	0.12324	0.96086	0.91464	2.14707	-0.36607	-0.39043	-0.63903	-0.91183	0.65217
S88	-1.02032	-1.11932	-1.00416	-0.75783	-0.29892	0.22618	-0.04149	1.32295	0.3071	2.49614	-0.46156	-0.50253	-0.56547	-0.15429	0.33855
S89	-0.56279	-0.69268	-0.88578	-0.80087	-0.63939	-0.29994	-0.5654	0.30591	-0.45539	0.48412	-0.97112	-0.76229	-0.69983	-0.13553	-0.74885
S90	0.34611	-0.1109	-0.32116	-0.56313	-0.75773	-1.01314	0.17471	-0.59944	1.29924	-0.68239	0.20141	-0.55092	-0.8446	-1.41426	0.93254
S91	-1.36435	-1.19791	-1.1061	-0.84762	-1.12254	-0.70625	2.07151	1.92846	1.97157	0.63313	-0.83312	-0.88021	-0.97222	-1.64084	3.53021
S92	-1.15454	-1.28079	-0.9229	-0.94771	-0.93935	-1.18029	-0.7577	-0.32929	-1.17043	-0.82045	-1.29669	-0.93461	-0.96932	-1.21929	-0.5529
S93	-1.09263	-1.15878	-0.90373	-0.7969	-0.77226	-0.50106	-0.00072	0.99464	0.48702	1.54871	-0.67963	-0.71742	-0.81965	-0.40401	0.17884
S94	-1.10132	-1.24836	-0.86593	-0.87085	-0.52529	0.69888	0.81957	1.436	0.10674	-0.62021	-0.50123	-0.39525	-0.64354	0.21766	1.80334
S95	-1.01103	-1.18002	-0.83527	-0.8586	-0.87344	-1.41444	-1.77618	-1.07208	-1.743	-0.69702	-1.17844	-0.84609	-0.9676	-1.60592	-1.7849
S96	-1.25785	-1.31556	-1.11614	-1.01727	-0.95513	-1.47243	-1.77828	-1.246	-1.60063	-0.53425	-1.33353	-1.02058	-0.89555	-1.54745	-1.815
S97	-1.33271	-1.4069	-1.24168	-1.12992	-1.14477	-1.33753	-1.44294	-1.08437	-1.39899	-1.05157	-1.41018	-1.10176	-1.04023	-1.18607	-1.46372
S98	-0.13999	-0.30093	-0.18767	-0.10384	0.41385	-0.1442	-0.53538	-0.50922	-1.05444	-0.57398	-0.32289	0.25462	0.22442	-0.4514	-0.68422
S99	-1.20819	-1.35207	-1.06663	-1.01838	-0.98599	-1.36482	-1.67861	-1.4067	-1.64799	-1.12687	-1.30946	-0.95296	-0.99661	-1.5029	-1.56343
S100	-0.37264	-0.58491	-0.42744	-0.53365	-0.31445	-0.16755	-0.42722	-0.24263	-0.56503	-0.399	-0.66091	-0.51903	-0.53696	-0.14191	-0.43026
S101	-1.02408	-1.17221	-0.77708	-0.85794	-0.79063	-0.81565	-1.02544	0.54386	-0.34552	2.00391	-0.68432	-0.63386	-0.81497	-0.97301	-0.78147
S102	-0.55274	-0.79649	-0.6606	-0.75111	-0.71314	-0.63869	-0.61255	0.02155	-0.34611	0.2565	-0.87279	-0.72333	-0.79522	-0.77891	-0.27516
S103	-0.89928	-0.92647	-0.58592	-0.41256	0.38044	2.4066	2.00887	6.10247	2.91617	3.16847	-0.01822	0.08984	-0.20777	1.84517	2.0453
S104	-0.7521	-0.88258	-0.76259	-0.72686	-0.58318	-0.5508	-0.7715	-0.22169	-0.27313	0.65557	-0.73594	-0.68675	-0.7164	-0.63857	-0.70523
S105	-0.97693	-1.0479	-1.1382	-1.04371	-1.06795	-0.30831	3.95273	3.68605	3.09424	0.45074	-0.57598	-0.78754	-0.91179	1.87664	3.67932
S106	-1.06555	-1.03086	-0.57821	-0.62505	-0.76229	-0.65263	-0.65642	0.81468	0.02383	1.90995	-0.61386	-0.60773	-0.83107	-0.8803	-0.35342
S107	-0.86183	-0.91165	-0.49141	-0.82238	-0.98715	-0.89326	-0.62834	1.42397	-0.21644	2.37951	-0.25138	-0.38647	-0.86356	-1.35016	0.62327
S108	-1.16881	-1.26702	-0.99973	-0.93125	-0.93283	-1.05516	-0.98838	-0.61756	-0.90314	-0.70122	-1.07158	-0.86058	-0.9433	-0.99633	-0.92991
S109	0.36287	0.12397	-0.00721	-0.30624	-0.28965	0.08801	-0.02313	0.73504	0.24729	0.98579	-0.2806	-0.41363	-0.64194	-0.61155	0.61805

S1	0.77524	0.85342	1.77903	1.62825	1.12108	0.23093	-0.26869	-0.62025	-0.64369	-0.51799	0.2022	0.07564	-0.06574	0.23761	0.87673
S2	-0.69188	-0.02244	0.44899	0.27724	0.24194	-0.02264	-0.35801	-0.57077	-0.42032	-0.49577	-0.2848	-0.05824	0.12302	1.22833	1.75681
S3	1.16617	0.88359	1.76591	1.56065	1.15308	0.29518	-0.30278	-0.55958	-0.59241	-0.45244	0.48272	-0.48852	-0.57443	-0.01311	1.48887
S4	-0.91754	-0.74674	-0.88596	-1.00565	-1.10171	-0.5968	-0.66442	-0.99439	-1.03232	-1.01576	-0.47426	-0.40752	-0.60345	-0.89082	-0.5945
S5	-0.88177	-0.47157	-0.05175	0.02833	0.24015	-0.01594	-0.44643	-0.9352	-0.82242	-0.79248	-0.68727	-0.70253	-0.62759	-0.46036	-0.57414
S6	0.42249	-0.42529	-0.29085	-0.09292	2.04666	0.79668	-0.24148	-0.50769	-0.33128	1.15653	1.5627	0.14901	0.03567	-0.2707	-0.24009
S7	-0.91018	-1.03489	-1.26789	-1.3393	-1.18537	-0.63164	-0.7351	-1.00829	-1.25495	-1.5638	-1.36752	-1.23399	-0.27442	-0.72759	-1.22903
S8	-0.24983	-0.06169	0.46848	0.67487	1.77613	0.6827	-0.16004	-0.42603	-0.31485	0.73103	1.5731	0.238	-0.29014	-0.71339	0.2863
S9	2.14227	2.27884	4.03416	3.69491	2.56252	0.80912	0.62689	0.13642	-0.06514	-0.35983	0.76072	2.41146	3.57656	3.15104	2.31999
S10	-0.69456	-0.33313	0.16207	0.47992	0.72751	0.10952	-0.29368	-0.33955	-0.11073	0.17017	0.11991	-0.08228	0.13301	0.69561	0.2239
S11	0.87463	0.90475	1.60019	1.31831	0.66207	-0.00574	-0.40011	-0.78086	-0.89007	-1.50299	-1.32273	-1.11995	-0.8389	-0.6705	-0.29485
S12	0.81189	0.51484	0.78603	0.67755	0.5817	0.04554	-0.3552	-0.16691	-0.17307	-0.01532	0.06587	-0.52149	-0.427	-0.7649	-0.1745
S13	-0.30485	0.17283	1.2405	1.49641	1.62504	0.68207	0.98571	0.51133	0.32201	0.3559	-0.33339	0.39128	1.35916	2.54479	1.2812
S14	-1.08812	-1.09564	-1.18153	-0.95873	-0.39644	-0.27196	-0.5571	-1.00726	-0.99431	-1.20385	-1.10181	-0.53746	0.76827	-0.03995	-1.1603
S15	0.0494	1.00798	3.19167	4.03981	3.57543	1.48765	0.69515	0.14232	0.2558	1.10384	2.01521	5.69582	6.65178	3.97043	0.60756
S16	0.83319	0.44423	0.35795	0.10729	0.15911	-0.09139	-0.43129	-0.69267	-0.63503	-0.69908	-0.43934	-0.76018	-0.65362	-0.62903	0.29399
S17	1.31898	1.2562	2.27436	1.98962	1.375	0.29884	-0.15194	-0.40565	-0.31076	0.13424	1.04209	-0.33717	-0.38724	0.07831	2.11941
S18	0.31405	0.45088	1.11603	0.89434	0.32288	-0.09118	-0.34218	-0.47962	-0.26162	-0.00487	0.7493	0.4147	0.50219	1.33564	2.13705
S19	1.15296	0.52156	0.06104	-0.49344	-0.77339	-0.21654	-0.47537	-1.09205	-0.64097	-0.16352	0.44029	0.35575	-0.28556	-0.15614	-0.19554
S20	0.05271	0.32462	1.11766	1.27462	1.60131	0.59151	-0.14013	-0.36962	-0.25265	0.14674	0.69618	0.21442	0.47329	0.87275	1.37099
S21	-0.90359	-0.12752	1.01079	1.25624	1.28149	0.50643	0.20153	0.65984	1.32826	1.40188	1.11587	1.53183	1.32587	2.13472	1.78733
S22	0.81813	-0.48505	-1.35235	-1.63273	-1.58412	-0.7675	-0.82177	-1.24898	-1.2502	-1.52666	-1.65053	-1.88433	-1.38955	-1.13582	-0.80521
S23	0.14264	-0.16527	-0.23054	-0.38139	-0.43771	-0.28697	-0.59808	-1.02302	-1.05172	-1.37796	-0.81439	-0.78197	-1.05534	-0.79173	-0.56409
S24	0.62055	0.00476	-0.13597	-0.32153	-0.1863	-0.2454	-0.56752	-0.99223	-0.80349	-0.4591	-0.6435	-1.14864	-1.01294	-0.83699	-0.17733
S25	-0.31011	0.02125	0.79872	1.09025	0.95345	0.29213	-0.04747	-0.28041	-0.13665	0.4707	1.19866	1.2524	1.52128	2.67593	1.81103
S26	0.09106	0.04998	0.59985	0.83661	0.91822	0.30548	-0.1354	-0.15783	-0.03982	0.74414	1.85337	1.127	0.47063	0.57154	1.22197
S27	-0.74564	-0.45603	-0.27997	-0.22091	-0.34025	-0.3133	-0.46856	-0.64672	-0.43605	0.58001	1.16745	-0.01367	-0.58887	-0.45216	0.72025
S28	0.0293	0.33508	1.01218	1.07575	0.73098	0.03289	-0.37552	-0.60476	-0.58905	0.52201	2.51783	0.4905	-0.52953	-0.48619	0.71169
S29	0.83714	0.71983	1.33861	1.28859	0.73869	0.00423	-0.45517	-0.78992	-0.83842	-0.61101	0.52803	-0.29332	-0.94161	-0.61716	0.77933
S30	0.22506	0.32767	0.57281	0.29191	0.03277	-0.15281	-0.43051	-0.25354	-0.02685	0.25639	0.638	-0.09279	-0.18654	-0.25084	0.29763
S31	-0.30339	-0.43873	-0.06296	0.17897	0.36715	-0.01186	-0.41907	-0.88459	-0.79964	0.42101	1.4958	-0.17177	-0.55439	-0.37284	0.73383
S32	1.17485	0.42056	-0.1248	-0.68974	-1.1701	-0.69455	-0.78176	-0.56609	-0.42199	-0.27098	0.52259	0.48149	0.52023	1.01698	1.78394
S33	-0.34189	-0.08909	0.34756	0.37	0.3545	-0.04044	-0.38204	-0.13455	-0.02573	0.47542	0.92514	-0.10347	-0.62106	-0.78662	-0.0321
S34	-1.0196	-0.87726	-0.92012	-0.8764	-0.88799	-0.52896	-0.60879	-0.98689	-1.02106	-0.04622	0.55063	-0.86156	-0.82787	-0.89253	0.01417
S35	0.80574	0.28952	0.14322	-0.33462	-0.65056	-0.43954	-0.59882	-0.67809	-0.60197	-0.23233	1.00214	-0.32415	-0.69462	-0.21304	1.63608
S36	0.97449	0.4385	0.69988	0.33107	-0.02237	-0.27214	-0.56914	-0.88571	-0.92099	-0.28039	0.95739	-0.36732	-0.84213	-0.27571	1.2106
S37	-0.29032	-1.05905	-1.14724	-1.00807	-0.92553	-0.48145	-0.60059	-1.02851	-0.85232	-1.05367	-0.96499	-0.41685	-0.40707	-0.52398	-0.77272
S38	-0.66565	-0.67224	-0.37524	-0.00536	0.32829	0.03021	-0.2809	-0.49411	-0.28056	0.48704	0.65724	0.3295	0.64892	0.22283	0.0196
S39	-1.21689	-1.06512	-1.04521	-0.86454	-0.61668	-0.41399	-0.59784	-0.96677	-0.74513	-0.4417	-0.98786	-1.38266	-0.6901	-1.16841	-0.86224
S40	-0.80792	-0.78905	-0.85847	-1.04967	-1.04137	-0.54888	-0.61969	-0.81404	-0.72298	-0.62397	0.32906	0.41953	0.67571	1.27528	0.84788
S41	-0.93602	-0.7339	-0.63374	-0.55102	-0.55144	-0.41659	-0.55218	-0.74651	-0.68481	0.00565	0.88627	0.14276	0.41094	0.83024	0.96911
S42	-0.68142	-0.38886	0.07426	0.22171	0.15532	-0.11021	-0.35516	-0.57758	-0.41233	0.20245	0.87819	0.88958	1.40418	1.90274	1.27561
S43	-0.36616	-0.37656	-0.85761	-0.85653	-0.80307	-0.27832	0.21646	-0.00413	-0.61882	-1.87523	-1.80058	-1.50822	-1.39132	-1.29287	-1.25816
S44	-0.37524	0.18684	-0.7792	-0.81763	-0.83602	-0.47708	-0.13189	-0.30117	-0.41789	-0.94453	-1.66494	-1.29695	-1.30568	-1.32534	-1.23359

S45	-0.12841	-0.40514	-0.75062	-0.79783	-0.73633	-0.40396	-0.46216	2.13768	-1.2499	-1.7291	-1.43588	-1.34524	-1.12487	-1.1757	-1.20637
S46	-0.8863	-0.67105	-0.54471	-0.53268	-0.4772	-0.36682	-0.54514	-0.76536	-0.72975	-0.22956	0.4729	-0.21233	0.10535	0.4919	0.90064
S47	-0.02852	-0.1878	0.04951	-0.00499	-0.17942	-0.2467	-0.48175	-0.6631	-0.6051	-0.33303	0.58041	0.06776	0.22742	0.88312	1.79159
S48	-0.07166	-0.01603	0.44192	0.47642	0.24291	-0.10545	-0.40317	-0.60827	-0.56685	-0.10347	0.66653	-0.03663	0.13025	0.36679	1.41773
S49	0.56162	0.43175	1.20128	1.32665	0.99851	0.22766	-0.17987	-0.43753	-0.37296	-0.17036	0.66953	0.60171	0.66666	1.26318	2.01045
S50	-0.0227	-0.33337	-0.31298	-0.50319	-0.8107	-0.41311	-0.72377	-1.09866	-0.97849	-0.75502	-0.20847	-0.32126	0.09603	0.84987	-0.38911
S51	0.28054	-0.03404	0.32531	0.2911	0.2374	-0.03199	-0.37502	-0.64441	-0.58213	-0.31723	0.6289	0.91109	0.94907	0.98766	0.66322
S52	1.84094	0.88431	0.62016	-0.09114	0.184	0.16236	-0.56272	-0.5597	-0.46503	0.02608	1.09815	-0.28472	-0.21565	0.64964	0.60342
S53	0.63807	0.00322	0.13806	0.06744	0.26268	0.02015	-0.40099	0.84916	-1.00576	-0.75491	0.35783	-0.11476	-0.08771	0.26187	0.25053
S54	-0.31886	-0.43916	-0.21969	-0.14308	0.65113	0.19856	-0.41034	-0.87332	-0.66003	-0.06365	0.0707	-0.56086	-0.4668	-0.27142	0.11748
S55	-0.72654	-0.52566	-0.31055	-0.49189	-0.25102	0.23089	-0.06132	-0.5315	-0.76223	-0.937	-0.56372	-0.30011	0.83694	1.98791	0.84383
S56	0.71319	0.33793	0.42662	0.13027	0.17752	-0.01426	-0.42755	-0.47725	-0.43968	-0.47078	-0.01597	-0.32553	0.05178	0.4506	0.92354
S57	1.48893	0.62694	0.78776	0.58778	0.40298	0.0064	-0.39056	-0.63969	-0.74573	-0.77471	-0.25167	-0.41343	-0.17735	0.21238	0.67345
S58	-0.4833	-0.36062	-0.02187	0.07292	0.28098	-0.06302	-0.4564	-0.54245	-0.57166	-0.3938	-0.22904	-0.96152	-0.13172	-0.56308	-0.03947
S59	-0.62996	-0.54314	-0.61447	-0.81195	-0.67203	-0.43859	-0.65443	-1.03167	-1.09115	-1.26984	-1.09791	-1.3353	-0.85829	-0.9119	-0.68199
S60	-0.67625	-0.90404	-1.2013	-1.07652	-0.8696	-0.40956	-0.39121	-0.5262	-0.45131	-0.3484	0.07035	0.54209	1.73448	2.09216	0.86473
S61	0.86121	0.41752	0.9157	1.1526	1.36417	0.43268	-0.23115	-0.79419	-0.86342	-0.82363	-0.16109	-0.10152	0.41455	0.93794	-0.61961
S62	-1.34602	-1.07983	-1.13095	-1.09309	-0.86881	-0.47833	-0.60281	-0.82696	-0.69483	-0.371	0.41448	0.2022	0.26894	-0.02329	-0.18655
S63	0.78127	-0.00761	0.34025	0.53067	0.79979	0.32436	-0.12784	-0.12535	-0.03273	0.54119	1.49076	1.21527	1.3004	1.71583	1.42878
S64	0.77419	0.12417	-0.18018	-0.64179	-0.55154	-0.36593	-0.70946	-0.89751	-0.95791	-0.82472	0.04903	-0.56984	0.01034	0.39512	0.23853
S65	0.71523	0.30341	0.34539	0.15244	-0.20018	-0.24578	-0.36943	-0.23359	0.16566	0.51727	1.59344	1.66178	0.70872	0.64767	0.44949
S66	-0.13314	-0.49052	-0.44199	-0.45397	-0.51828	-0.37604	-0.51108	-0.52169	-0.47594	0.4165	1.56844	0.82417	0.67622	0.59	0.36102
S67	1.40816	1.00575	1.08739	0.9307	0.50593	0.01705	-0.22291	-0.25269	-0.31879	0.00716	1.16435	1.39242	0.75941	0.6022	-0.22699
S68	0.16794	0.00762	0.26453	0.13934	-0.06379	-0.21548	-0.36561	0.1693	0.99489	1.64511	1.88213	1.24625	0.30603	0.4843	1.49689
S69	-0.70969	-0.64758	-0.50045	-0.30014	-0.15872	-0.05383	1.40779	1.25625	1.08787	1.40521	0.27526	1.21797	0.84734	0.34409	-0.58979
S70	-1.05369	-0.8777	-0.91958	-0.79379	-0.76143	-0.39046	-0.3259	0.09619	0.55146	-0.11279	-0.97438	-0.41418	-0.45322	-0.72227	-0.92068
S71	-0.65024	-0.62747	-0.66373	-0.65798	-0.7393	-0.43763	-0.27052	0.66643	1.48295	0.98279	-0.33674	0.47323	0.31774	-0.08385	-0.61491
S72	-0.33366	-0.34415	-0.1478	-0.18106	-0.44445	-0.40127	-0.36896	1.14501	2.45806	2.26005	0.70234	1.17333	0.65619	0.05561	-0.26625
S73	-1.78121	-1.24353	-1.18724	-1.05176	-1.07615	-0.67165	-0.08739	-0.5866	-0.54928	0.49519	0.30293	1.03618	0.33876	-0.76102	-0.77366
S74	-0.98612	-0.82723	-0.71993	-0.30304	0.00918	0.11636	0.87832	0.62245	0.16169	-0.34669	-0.33336	0.73219	0.78344	0.37762	-0.46418
S75	-1.27989	-1.07727	-1.3498	-1.25946	-1.20303	-0.58395	-0.55966	0.03808	0.55351	-0.44104	-1.499	-1.08669	-0.92124	-1.00137	-1.14711
S76	0.82834	0.64387	-0.01372	-0.25348	-0.3764	1.68573	2.18608	1.53439	0.85582	-1.05646	-0.73398	0.22037	0.2298	0.0926	-0.74341
S77	-1.0446	-0.95493	-0.89048	-0.72589	-0.71969	-0.43061	-0.56418	-0.24153	-0.16586	-0.37526	-0.28344	-0.27906	-0.12911	-0.43534	-0.60066
S78	-0.05252	-0.09936	-0.06047	0.37932	0.27201	-0.02404	2.09498	2.00576	1.63817	1.28564	-0.80032	0.00932	-0.16153	-0.50501	-1.07338
S79	-1.18932	-0.98625	-1.18905	-1.2189	-1.01011	-0.33402	1.25988	1.28225	0.66285	-0.12668	-0.32904	0.48243	0.33071	-0.01699	-0.33026
S80	-0.60659	-0.78904	-1.03337	-1.01646	-0.94768	-0.34356	1.34812	1.19233	0.85067	0.59007	0.03457	0.7271	0.08198	-0.21154	-0.89972
S81	0.28769	-0.2296	-1.07938	-1.21401	-1.17056	-0.58763	-0.61765	0.3723	1.25545	0.60461	-0.67622	-0.98907	-1.15283	-1.21518	-1.16765
S82	-0.43982	0.21994	0.06268	-0.14603	-0.83149	-0.76565	1.01406	0.98991	1.05932	1.4434	-0.00753	0.53779	-0.28304	-0.76697	-1.15793
S83	-0.57701	0.41976	0.21602	0.73379	0.61884	0.24281	0.01762	1.59656	2.19442	3.48806	3.43493	3.03447	1.79045	0.78723	-0.45568
S84	-0.83961	-0.50789	-1.26496	-1.17655	-1.17086	-0.33908	0.01488	2.71007	3.98051	3.80149	0.76304	1.19263	0.45957	0.35706	-0.29721
S85	-0.84789	0.32503	-0.21429	0.45529	0.34313	-0.05497	1.0342	0.86261	0.82834	0.89893	-0.68572	-0.42249	-0.49627	-0.80681	-1.22455
S86	0.43042	-0.42805	-0.81169	-0.74536	-0.71722	-0.38754	-0.18251	0.25137	0.588	0.20292	-0.58996	0.14324	-0.09664	-0.22835	-0.35722
S87	0.26396	0.66656	0.29029	0.81039	0.81434	0.34007	1.17995	2.45148	2.86326	2.37859	-0.61948	0.14888	0.14512	-0.29011	-1.07397
S88	0.64913	0.48828	0.00399	-0.29284	-0.52543	-0.29145	2.35817	2.37871	1.95186	1.70847	-0.92555	-0.54368	-0.72002	-1.08511	-1.07929

S89	-0.02603	-0.63952	-0.76476	-0.76992	-0.73052	-0.35878	0.28923	0.81704	1.1756	1.19602	-0.06688	0.50095	0.24174	0.3922	0.52307
S90	-0.17022	2.83316	0.50649	0.15523	-0.18577	-0.18398	-0.16626	0.07918	0.23743	-0.37378	-1.10445	0.12936	-0.48297	-0.91372	-1.13482
S91	0.08096	6.71602	0.76719	-0.09402	-0.51947	-0.17792	1.16809	1.3293	0.17922	-1.4716	-1.15413	-0.57344	-0.62841	-0.59397	-1.05025
S92	-0.45755	-0.94238	-1.24593	-1.02077	-0.95055	-0.46159	-0.43307	-0.05582	0.37376	-0.45732	-1.38145	-0.86559	-0.83168	-0.98764	-1.19377
S93	0.63574	-0.19494	-0.57434	-0.46034	-0.54694	-0.32514	-0.21488	1.47905	3.00569	2.32072	0.01835	0.16009	-0.4825	-0.88368	-0.96092
S94	2.2284	2.29503	2.23768	2.0335	1.10167	0.13875	0.03444	1.32772	-0.34341	-0.77854	-1.03786	-0.40349	-0.68659	-0.74146	-0.99797
S95	-1.64275	-1.2155	-1.15828	-0.85238	-0.61545	0.22504	0.81852	0.55794	-0.14322	-0.72731	-0.85506	-0.22541	-0.54406	-0.96976	-1.16139
S96	-1.75295	-1.43844	-1.66983	-1.63725	-1.33504	-0.55561	0.39955	0.46952	0.14592	-0.18491	-1.1348	-0.63203	-0.55476	-0.68489	-0.83993
S97	-1.07526	-1.29399	-1.68253	-1.71409	-1.60132	-0.73783	-0.73935	-0.31197	-0.07145	-0.67393	-1.54532	-1.46691	-1.34323	-1.41838	-1.19017
S98	-0.85324	-0.29973	0.49436	0.96695	0.69666	0.11709	0.64056	0.10894	-0.04717	-0.07507	-0.55568	0.35923	0.514	0.02481	-0.48991
S99	-1.42606	-1.14494	-1.39116	-1.26691	-1.16648	-0.59525	-0.41459	-0.29155	-0.28024	-1.17078	-2.00873	-1.6412	-1.37439	-1.4702	-1.30485
S100	0.04401	-0.2473	-0.07241	0.05954	-0.12325	-0.17118	-0.36786	0.01498	0.50498	0.23755	-0.22325	0.496	0.50039	0.28023	-0.58318
S101	-0.54347	-0.55457	-0.77759	-0.75861	-0.66464	-0.15415	2.37926	2.60972	1.87808	0.69803	-0.44478	0.27698	-0.10558	-0.39207	-0.90036
S102	-0.06791	-0.29458	-0.5811	-0.4432	-0.40257	-0.28936	-0.35661	0.54249	1.5295	0.66918	-0.74072	-0.25848	-0.40097	-0.75053	-1.09194
S103	3.19733	1.48662	1.17835	1.38982	4.73812	9.21025	6.95177	3.66965	0.09891	-0.67345	0.00285	0.89439	0.59789	0.24162	-0.53987
S104	-0.3437	-0.41997	-0.41427	-0.37162	-0.47558	-0.27355	-0.22769	0.87032	1.92478	1.27976	-0.30431	0.15026	-0.11494	-0.28452	-0.54752
S105	5.14181	1.15882	0.37034	0.32096	-0.05828	-0.17636	-0.51578	0.41229	0.84472	1.17594	0.849	0.12999	-0.50281	-0.90001	-1.07891
S106	-0.10023	-0.28133	-0.47644	-0.09914	-0.16099	-0.10416	2.14478	2.03493	1.58092	1.06636	-0.92273	-0.21543	-0.29009	-0.57707	-1.06328
S107	-0.06348	1.4358	0.54349	0.27832	-0.55984	3.01801	2.64915	1.87669	1.48839	-0.71469	-0.243	0.45981	-0.16176	-0.24246	-0.8987
S108	-0.54738	-0.7701	-0.95551	-0.67869	-0.65171	-0.40698	-0.57242	-0.11823	0.23216	-0.70161	-1.51002	-1.51849	-1.11469	-1.20903	-1.30147
S109	0.57646	1.15672	0.80089	0.81656	0.44663	0.06435	1.26012	0.9771	0.78844	0.80652	-0.4887	0.27451	0.37752	0.37635	-0.93647

Sample No.	integrated bins														
	2.20 .. 2.18	2.18 .. 2.16	2.16 .. 2.14	2.14 .. 2.12	2.12 .. 2.10	2.10 .. 2.08	2.08 .. 2.06	2.06 .. 2.04	2.04 .. 2.02	2.02 .. 2.00	2.00 .. 1.98	1.98 .. 1.96	1.96 .. 1.94	1.94 .. 1.92	1.92 .. 1.90
S1	1.25488	1.28598	1.39503	1.46728	1.62318	1.63414	1.32825	1.20631	0.78123	0.91787	0.90294	0.74723	0.44595	0.80872	1.2887
S2	1.17431	-0.55564	-0.63546	-0.37919	-0.07157	-0.1587	-0.30584	-0.49227	-0.43424	-0.4894	-0.34628	-0.42412	-0.17301	-0.44836	-0.46417
S3	1.56113	0.79529	1.2266	1.39752	1.46889	1.86262	1.69652	1.24425	0.54047	0.78913	0.7065	0.44009	0.28759	0.36271	0.33533
S4	-1.02869	-0.87167	-0.82808	-0.74572	-0.91663	-1.15939	-1.23415	-1.09393	-0.097	-0.2338	-0.00156	0.37053	0.736	0.40988	-0.19264
S5	-0.42763	-0.0409	0.36787	0.35022	0.40384	0.75834	0.60929	0.40089	-0.36073	-0.34137	-0.39784	-0.63439	-0.82841	-0.55784	-0.25282
S6	-0.0715	-0.26627	-0.50475	-0.5594	-0.05324	1.50473	1.46658	0.22618	0.45141	0.6747	0.53984	0.84531	1.04341	0.32011	-0.08188
S7	-1.47439	-0.8969	-1.02326	-1.32988	-1.62282	-1.60667	-1.65795	-1.70718	-1.73692	-1.73157	-1.77386	-1.70441	-1.45777	-1.60135	-1.42241
S8	-0.10101	-0.46963	-0.37565	-0.279	0.20486	1.30579	1.34389	0.45927	0.7719	1.00229	0.94337	1.01108	1.3827	0.91154	0.36665
S9	2.01331	0.42892	1.20311	1.73396	2.11361	1.9822	1.83124	1.44429	0.89817	1.23881	1.09632	1.37767	0.67736	1.68296	1.84342
S10	-0.48009	-1.26031	-0.72258	-0.43186	-0.00335	0.3713	0.39724	0.15859	-0.18716	0.10632	-0.01875	-0.09673	-0.18309	-0.1193	0.89557
S11	-0.73228	-0.85568	-0.67963	-0.77781	-0.99622	-1.24989	-1.29417	-1.33902	-1.7418	-1.83816	-1.71102	-1.96235	-1.86647	-1.74895	-1.64585
S12	-0.04532	-0.13417	-0.31864	-0.30691	-0.40295	-0.33118	-0.49575	-0.68289	-1.10669	-1.12407	-1.22053	-1.20757	-1.2157	-1.11092	-0.88597
S13	0.64866	-0.71215	0.07975	0.43825	0.60881	1.02707	1.06484	0.55371	-0.05963	-0.04001	-0.03078	-0.08752	-0.3859	-0.22558	0.14607
S14	-1.35802	-0.67193	-0.69453	-0.904	-1.07234	-0.84387	-0.97666	-1.1741	-1.35449	-1.37175	-1.43564	-1.31022	-1.28417	-1.25957	-0.68539
S15	-0.19885	-0.26459	1.54788	2.7792	3.10319	2.62278	2.80121	3.51779	2.59531	1.83156	1.46845	1.56381	0.51604	1.68567	3.43062
S16	-0.2177	-0.52047	-0.80204	-0.73673	-0.54786	-0.2682	-0.27403	-0.48285	-0.83727	-0.5032	-0.75158	-0.91995	-0.74526	-0.90761	-0.81247
S17	2.11772	0.69895	1.09246	1.29749	1.51296	2.05992	2.1328	1.74668	0.81865	1.57045	1.19138	0.78393	0.44413	0.67205	0.78932
S18	1.69211	-0.28596	-0.07865	0.06409	0.42745	0.86503	1.18207	1.1068	0.41925	0.91764	0.46296	-0.03117	-0.29723	-0.05963	0.2319
S19	2.59913	2.12791	-0.54229	-0.97749	-0.93876	-0.35653	0.6854	0.29918	-0.96427	-0.96496	-0.50296	-0.48908	-0.69596	0.0307	-0.48295
S20	1.20499	0.03137	0.11777	0.23193	0.49835	1.34806	1.56563	0.90447	0.22021	0.62069	0.4181	0.28021	0.07236	0.2802	0.43587

S21	1.36839	0.2764	0.85412	1.14483	0.97043	1.04866	1.25139	0.84264	0.2893	0.45229	0.23918	0.24315	-0.01879	0.47268	0.49872
S22	-0.24681	-0.14874	-1.07834	-1.46723	-1.78142	-1.84409	-1.83711	-1.9394	-2.15826	-2.10583	-2.22211	-2.24918	-1.99722	-2.00174	-1.85384
S23	0.14527	-0.11439	-0.76393	-1.17382	-1.24616	-0.93106	-0.65711	-1.06906	-1.55932	-1.40551	-1.25867	-1.42093	-1.45558	-0.99352	-1.25036
S24	0.37606	0.0469	-0.44397	-0.68221	-0.45159	-0.46263	-0.67917	-0.79597	-0.96315	-0.94504	-1.07103	-1.10145	-0.95557	-0.95646	-0.8529
S25	0.93908	-0.89945	-0.52653	-0.19874	0.09531	0.27292	0.42302	0.42304	0.08071	0.569	0.39949	0.20426	-0.00687	0.18672	1.22101
S26	0.68536	-0.12042	-0.59684	-0.44307	-0.07366	0.45687	0.55213	0.61502	0.71288	1.11522	0.90824	0.82149	0.78829	0.76321	1.23046
S27	0.22702	-0.8549	-0.62068	-0.5055	-0.23792	0.04183	0.10703	0.1655	0.3968	0.88174	0.53325	0.51259	0.8043	0.25475	-0.1052
S28	0.55957	0.03269	0.46478	0.48331	0.75726	1.50763	1.66427	1.59909	0.93996	1.96107	1.38099	1.00131	0.92174	0.94432	0.64887
S29	0.75312	0.29436	0.71324	0.79114	1.15412	1.4117	1.15768	1.17217	1.13343	1.40552	1.39353	1.22512	1.23241	1.5334	1.3415
S30	-0.21691	-0.55856	-0.54672	-0.32722	0.1303	0.25121	0.07107	0.23527	0.36507	0.60605	0.71003	0.52327	0.52887	0.45326	0.42007
S31	0.2689	-0.53958	-0.62252	-0.64962	-0.42217	0.42503	0.78289	0.32076	-0.28526	0.47558	-0.13254	-0.41394	-0.37899	-0.58672	-0.47263
S32	1.92786	1.4227	1.0692	1.04969	1.0328	0.77727	0.68561	0.81801	0.65674	0.97503	1.09971	0.93452	0.71478	1.08815	1.61637
S33	-0.43796	-0.58677	-0.58162	-0.48471	-0.40742	-0.43334	-0.596	-0.50363	0.11821	-0.07839	-0.04775	0.12538	0.46278	0.07228	-0.2494
S34	-0.53341	-0.9142	-0.96782	-0.99443	-0.85026	-0.57514	-0.44486	-0.57832	-0.78312	-0.03958	-0.658	-0.71753	-0.65418	-0.97939	-1.02814
S35	1.29193	0.20398	-0.47623	-0.50042	-0.16435	0.08928	0.11785	0.01212	-0.51195	0.30333	0.00247	-0.37567	-0.38819	-0.51907	-0.5588
S36	0.9676	-0.03986	-0.13492	-0.0751	0.32126	0.60039	0.38345	0.16701	-0.16935	0.5088	0.23581	-0.06021	-0.0737	-0.26857	-0.347
S37	-0.43033	-0.16687	-0.66484	-0.83713	-1.02731	-0.92413	-0.83481	-0.5764	-0.69004	-0.66651	-0.73173	-0.70846	-0.90394	-0.65558	-0.15177
S38	-0.67681	-0.90683	-0.92974	-0.8288	-0.67481	-0.5133	-0.46067	-0.17598	0.45878	0.31231	0.36492	0.71858	0.74303	0.31286	0.48664
S39	-1.11625	-1.01222	-1.05082	-1.23299	-1.54217	-1.51288	-1.50056	-1.57055	-1.67672	-1.84219	-1.8443	-1.72984	-1.52476	-1.48837	-1.42685
S40	0.21965	-0.80853	-0.68519	-0.6622	-0.59878	-0.54166	-0.56678	-0.73176	-0.61799	-0.32404	-0.30317	-0.08173	-0.00727	-0.19436	-0.32281
S41	0.37983	-0.90213	-0.60556	-0.47608	-0.2828	-0.16877	-0.23822	-0.33348	0.07921	0.28716	0.36662	0.48892	0.82064	0.0579	-0.17989
S42	0.65443	-0.84899	-0.31654	-0.01739	0.20743	0.29342	0.2209	0.1444	0.46532	0.57232	0.68202	0.76628	0.83663	0.41351	0.68942
S43	-1.47927	-0.06177	0.01123	-0.36148	-1.04345	-1.19547	-1.06637	-0.83776	-1.28985	-1.45973	-1.4896	-1.63575	-1.60823	-1.44375	-1.31219
S44	-1.19645	-0.35814	-0.4947	-0.80086	-1.09513	-1.31857	-1.21413	-1.00841	-1.21748	-1.42194	-1.44668	-1.48109	-1.38248	-1.25977	-1.16714
S45	-1.31994	-0.56412	-0.71095	-1.04568	-1.43291	-1.62324	-1.49793	-1.21444	-1.28232	-1.29802	-1.49169	-1.43295	-1.3749	-1.35281	-1.3032
S46	0.34726	-0.90609	-0.67923	-0.58903	-0.42576	-0.25034	-0.33014	-0.51283	-0.20415	-0.02718	-0.03252	0.07577	0.3524	-0.23532	-0.41384
S47	1.29411	-0.26786	-0.31582	-0.17336	0.06448	0.17357	0.00741	-0.19804	-0.21095	-0.02278	0.01057	-0.04059	0.14917	-0.1451	-0.10936
S48	1.02382	-0.28303	-0.02207	0.21541	0.53571	0.6546	0.48376	0.3096	0.31242	0.50801	0.45714	0.48878	0.50518	0.25275	0.23062
S49	1.62687	0.08742	0.08742	0.34486	0.65571	0.73949	0.60632	0.4517	0.05548	0.32003	0.27626	0.10436	-0.0699	0.1979	0.72204
S50	0.58058	0.63297	-0.06187	-0.6003	-0.78375	-0.39523	-0.34581	-0.73624	-0.71034	-0.52686	-0.54959	-0.36366	-0.27336	-0.13601	-0.00054
S51	0.11048	-0.66547	-0.22526	0.15033	0.33698	0.25794	0.37806	0.82025	1.16595	0.77535	1.0223	1.05693	1.23629	1.11517	1.60665
S52	1.28245	0.94658	0.40297	0.21366	0.65311	1.63316	1.62829	1.10267	0.53014	0.9003	0.88605	0.47702	0.38789	0.26152	0.15484
S53	0.40958	0.07994	-0.37354	-0.48746	-0.23757	0.22577	0.19058	0.11118	0.11233	0.39943	0.32409	0.25269	0.40263	0.07765	0.00059
S54	-0.12341	-0.34266	-0.62954	-0.7252	-0.46483	0.16492	0.14376	-0.30301	-0.36946	-0.32246	-0.3545	-0.36941	-0.15966	-0.38126	-0.25119
S55	0.32802	-1.06476	-0.59204	-0.56558	-0.44706	0.02007	0.25434	0.09054	-0.0851	-0.19723	-0.0696	-0.23264	-0.31235	-0.32838	0.0248
S56	0.64626	0.0115	-0.60702	-0.654	-0.4251	-0.17943	-0.16029	-0.16823	-0.37005	-0.32237	-0.27473	-0.46299	-0.44821	-0.31237	0.25726
S57	0.63886	0.25635	-0.12093	-0.1288	0.00769	0.15988	0.0853	0.12041	-0.0755	-0.01019	-0.06039	-0.22299	-0.25382	-0.18209	0.16367
S58	-0.24491	-0.21784	-0.40107	-0.45656	-0.55195	-0.30088	-0.22095	-0.47093	-0.72996	-0.70989	-0.83104	-0.82754	-0.78432	-0.75494	-0.62276
S59	-0.35531	-0.52368	-0.86246	-1.13047	-1.26779	-1.16254	-1.08774	-1.0545	-1.18893	-1.21775	-1.34348	-1.36722	-1.23593	-1.24404	-0.96883
S60	-0.21585	-1.50196	-1.66188	-1.54844	-1.40258	-1.5076	-1.25749	-0.87931	-0.66361	-0.51868	-0.5075	-0.45963	-0.38787	-0.435	0.86882
S61	-0.55502	-0.29659	0.00273	-0.00743	0.18078	0.86448	0.78453	0.49736	0.21526	0.38924	0.27951	0.11728	-0.0452	0.08306	0.50992
S62	-0.78779	-1.13265	-0.90969	-0.91009	-1.02576	-0.88074	-0.86505	-0.84894	0.30091	0.13434	0.45533	0.88921	1.60569	0.66406	-0.00203
S63	0.79597	-0.055	-0.5005	-0.18407	0.35285	0.69304	0.78557	0.84238	0.59517	0.94736	0.78362	0.59506	0.38975	0.50039	1.34265
S64	-0.11694	-0.24214	-0.32218	-0.32278	0.03339	0.2861	0.32425	0.42436	0.37197	0.44232	0.41252	0.30186	0.12092	0.047	0.29223

S65	0.12064	0.12565	0.14933	0.29895	0.35134	0.33124	0.53714	0.69254	1.64027	2.24059	2.12863	2.84649	2.85507	2.902	1.87144
S66	-0.15642	-0.51668	-0.5442	-0.40497	-0.3739	-0.29017	-0.23889	-0.02223	0.88631	0.98586	1.13808	1.3098	1.60318	0.67987	0.25608
S67	0.35103	0.513	0.79976	1.21583	1.45968	1.24034	1.24155	1.34015	1.34157	1.68901	1.68311	1.56517	1.36762	1.35714	1.51813
S68	1.00246	-0.44443	-0.1987	-0.15852	-0.07774	0.19542	0.18993	0.1228	1.10218	1.44534	1.50706	1.6309	2.40694	1.37017	0.66017
S69	0.29652	1.47076	1.15919	1.40556	1.41923	1.08475	1.09434	1.50181	1.43246	1.43564	1.71616	1.49042	1.07976	0.93068	0.9732
S70	-0.57922	-0.20711	-0.18741	-0.1186	-0.32639	-0.77112	-0.88125	-0.72305	-0.61997	-0.96592	-0.86062	-0.60928	-0.5754	-0.7629	-0.87778
S71	0.22671	0.5528	0.83801	1.08452	1.11809	0.54385	0.2917	0.37777	0.40346	0.04335	0.20827	0.34922	0.28783	0.23025	0.17732
S72	0.57031	0.83049	1.21375	1.63073	1.93516	1.42311	1.15973	0.97435	1.33365	0.97587	1.28027	1.30442	1.31504	1.30829	1.05576
S73	-1.24873	-0.9784	-0.28746	1.07838	0.85444	0.16826	0.43785	-0.67219	1.36267	1.2591	2.14298	2.61878	3.56124	3.54903	2.18568
S74	-0.12054	-0.43618	-0.38555	-0.14637	-0.16977	-0.47255	-0.67702	-0.48313	-0.13937	-0.40736	-0.24543	0.06511	0.01652	-0.57533	-0.58459
S75	-1.20043	-0.26321	-0.17244	-0.42684	-0.80709	-1.23464	-1.34635	-1.2778	-1.36699	-1.63728	-1.57547	-1.43248	-1.28574	-1.34595	-1.45633
S76	0.40576	2.52971	2.49703	2.68048	2.45513	1.19441	1.35211	2.62148	3.79577	0.48543	0.32729	0.06893	-0.22973	-0.07105	0.11492
S77	-0.10146	-0.03002	0.22077	0.34603	0.17463	-0.26526	-0.52163	-0.47189	-0.53104	-0.89648	-0.8291	-0.63132	-0.68289	-0.6816	-0.65862
S78	-1.15331	0.13958	1.02167	0.88263	0.55238	0.30326	0.45306	0.69641	0.40783	0.20825	0.01731	-0.07412	-0.51806	-0.14529	0.22461
S79	0.26915	0.08364	0.29133	0.64303	0.78857	0.07687	0.17452	1.07199	1.26049	0.15974	0.78895	0.71686	0.70616	0.28911	-0.03322
S80	-0.36937	1.13259	1.00913	0.90719	0.98984	0.49566	0.55316	0.97525	1.59256	1.15252	1.4537	1.40255	1.55047	1.28778	0.77493
S81	-1.24562	-0.81535	-1.00578	-1.35595	-1.73215	-1.86423	-1.77713	-1.50418	-0.80783	-1.03364	-0.86707	-0.61212	-0.3193	-0.69157	-1.29204
S82	-1.2919	1.24037	2.64	2.37338	1.57061	1.05354	0.80873	0.72247	1.20729	0.94963	1.06561	1.26459	1.51102	2.17734	2.06313
S83	0.7522	4.13562	0.52841	0.07848	0.14714	0.01382	-0.22798	-0.16477	0.09482	0.7038	0.8927	0.98578	1.14747	2.35641	0.65853
S84	0.5987	2.81403	-1.19218	-1.61607	-1.25079	-1.82001	-2.0026	-1.7103	-1.4804	-1.1153	-0.89779	-0.68458	-0.427	-0.03488	-2.22088
S85	-1.67972	-0.41214	0.93761	1.40602	1.69192	1.24	1.77487	3.04816	2.11485	2.4339	2.23383	1.3787	0.05156	0.93615	1.37021
S86	1.18695	0.84965	0.42995	0.39182	0.42429	-0.10271	-0.34098	-0.33682	0.05715	-0.31168	-0.05694	0.04535	0.26214	0.04869	-0.09681
S87	-1.34068	-0.35916	0.53616	0.56348	0.30031	0.17004	0.08547	0.24797	-0.16601	-0.22296	-0.41558	-0.50084	-0.95195	-0.51129	-0.05782
S88	-1.32381	-1.03459	-0.74459	-0.68265	-0.89832	-1.13291	-1.1635	-0.93863	-0.73752	-1.11208	-0.96555	-0.84941	-0.66182	-0.77431	-0.92233
S89	1.70887	0.84251	0.03905	-0.03436	-0.08972	-0.45087	-0.56021	-0.50515	0.35218	-0.09193	0.65315	0.72357	0.95762	0.39378	-0.13731
S90	-0.59559	1.78751	0.6657	-0.14597	-0.31808	-0.40801	-0.38292	-0.52049	-0.79914	-0.63641	-0.59326	-0.46692	-0.44701	0.55005	-0.0072
S91	-1.29611	0.95102	3.65243	2.73247	1.23061	0.16564	-0.13086	0.04332	0.12677	-0.01481	0.27193	0.07641	-0.16506	0.04576	-0.13772
S92	-1.40484	-0.61193	-0.32227	-0.5829	-0.93603	-1.2037	-1.25084	-1.10471	-0.97913	-1.14162	-1.06979	-0.86777	-0.8415	-0.95708	-1.12677
S93	-0.71013	-0.50298	-0.39129	-0.43527	-0.71087	-0.90959	-0.87131	-0.50702	0.54794	0.23049	0.41545	0.75168	0.89495	0.5708	0.14436
S94	-0.04217	2.45185	2.76078	2.13027	1.5153	1.06961	1.05746	1.33448	0.89985	0.72486	0.63047	0.4421	0.03193	0.70816	1.08567
S95	-1.43702	-0.62482	-0.35831	-0.42043	-0.6873	-0.95088	-1.05313	-0.80595	-0.23895	-0.51163	-0.44709	-0.05849	0.15182	-0.09391	-0.47406
S96	-0.88992	-0.78439	-0.67154	-0.75759	-1.03176	-1.39686	-1.32685	-0.99495	-0.80369	-1.14215	-1.09406	-1.0714	-0.80335	-1.11825	-1.35702
S97	-1.35796	-1.0159	-1.00594	-1.18169	-1.55364	-1.91101	-1.96201	-1.80539	-1.59127	-1.9526	-1.8324	-1.64689	-1.34499	-1.54577	-1.84467
S98	-1.0584	-1.01502	-0.25767	0.07047	0.01928	-0.33697	-0.24796	0.15833	0.33617	-0.00131	-0.0893	0.09053	0.1126	0.28335	0.37131
S99	-1.65525	-1.00459	-0.81464	-1.08058	-1.49394	-1.79946	-1.82585	-1.74977	-1.93668	-2.20582	-2.13951	-2.10877	-1.89614	-1.79055	-1.78068
S100	0.33322	0.96195	0.78054	0.81046	0.77912	0.43191	0.11751	0.03455	0.18217	0.0199	0.11707	0.25993	0.0582	-0.00451	-0.04454
S101	-0.71159	0.43256	0.60773	0.52718	0.28965	-0.1561	-0.06157	0.19542	0.34369	-0.05036	0.01552	0.04855	0.07688	0.16892	0.12449
S102	-1.29063	-0.65366	-0.39362	-0.47431	-0.7113	-1.03969	-1.17804	-0.89686	-0.57866	-0.86213	-0.84096	-0.57341	-0.48653	-0.58425	-0.74177
S103	0.58547	0.65565	0.53019	0.60643	0.79137	0.39144	0.4338	0.77134	0.8748	0.81545	0.97589	0.942	0.75867	0.9944	0.87314
S104	0.79354	0.93079	0.30371	0.34774	0.29787	-0.12061	-0.36332	-0.23319	0.16957	-0.15189	-0.03838	0.09596	0.1888	-0.091	-0.31594
S105	-1.05672	-0.38612	-0.28215	-0.29179	-0.58451	-0.8187	-0.73629	-0.41512	0.94988	0.53206	0.91112	1.02655	1.24971	0.85346	0.36114
S106	-1.11632	0.04008	0.58562	0.52699	0.26896	0.01796	0.1789	0.43817	0.15012	0.02388	-0.16452	-0.3176	-0.66276	-0.41319	-0.1505
S107	0.3505	3.73405	4.83292	3.73136	2.87761	2.28512	1.93645	1.9731	1.93624	1.65776	1.7185	1.65323	1.32531	2.19082	2.37356
S108	-1.71176	-0.82783	-0.35081	-0.46981	-1.04866	-1.19114	-1.0823	-1.07761	-1.21062	-1.58718	-1.63676	-1.66917	-1.66055	-1.46832	-1.2912

S109	-0.39786	1.2451	1.74954	1.21023	0.82481	0.65041	0.43774	0.50993	0.70345	0.40326	0.52983	0.66178	0.35027	0.42337	0.40283

Sample No.	integrated bins														
	1.90 .. 1.88	1.88 .. 1.86	1.86 .. 1.84	1.84 .. 1.82	1.82 .. 1.80	1.80 .. 1.78	1.78 .. 1.76	1.76 .. 1.74	1.74 .. 1.72	1.72 .. 1.70	1.70 .. 1.68	1.68 .. 1.66	1.66 .. 1.64	1.64 .. 1.62	1.62 .. 1.60
S1	1.13549	1.4034	1.62046	2.0732	2.74777	2.80278	2.70801	2.5657	2.42721	1.82762	1.7296	1.80798	1.91417	1.97954	1.94575
S2	0.43169	0.92167	0.62281	0.32729	-0.44867	-0.8211	-0.85036	-0.66481	-0.45388	0.20927	0.58386	0.71941	0.5979	0.54817	0.67218
S3	0.49549	1.68765	2.10984	2.63422	2.96627	2.55558	2.17226	1.92392	1.33931	1.23061	1.76423	2.01564	2.19697	2.17261	2.21359
S4	-0.42366	-0.58995	-0.78206	-0.81536	-0.67949	-0.71005	-0.89394	-0.97223	-1.02659	-1.24462	-1.13011	-0.96963	-0.93147	-0.86703	-0.81118
S5	-0.21171	-0.10856	0.0857	0.40977	1.01592	1.07709	0.94083	0.77388	0.44043	-0.08729	0.01363	0.2074	0.23879	0.22652	0.15844
S6	-0.0874	-0.19323	-0.22246	-0.28047	-0.3621	-0.49552	-0.52355	-0.53445	-0.54307	-0.58583	-0.43277	-0.3117	-0.33044	-0.32745	-0.33032
S7	-0.59213	-0.24046	-0.21752	-0.34231	-0.73273	-1.16559	-1.28969	-1.2461	-1.09056	-1.00106	-0.64233	-0.45618	-0.5405	-0.58822	-0.53543
S8	1.32893	0.82693	0.80714	0.87223	0.95813	0.85712	0.63818	0.49392	0.39464	0.54498	0.63425	0.74433	0.77225	0.79135	0.90171
S9	0.8378	0.7517	0.86106	1.22312	1.30112	1.40413	1.63045	1.81211	1.5591	1.45713	1.24091	1.09766	1.1065	1.13622	1.10916
S10	2.13539	3.51846	3.00874	2.05034	0.41656	-0.15064	-0.00247	0.32441	0.81491	2.42129	3.27192	3.16585	2.88317	2.73114	2.962
S11	-1.5846	-0.75289	-0.77833	-0.82172	-1.08569	-1.29517	-1.48968	-1.62916	-1.70066	-1.46235	-1.09677	-0.89136	-0.881	-0.90351	-0.8508
S12	-0.17521	-0.53986	-0.63115	-0.61186	-0.547	-0.46663	-0.50839	-0.55957	-0.7194	-0.88136	-0.71692	-0.61072	-0.62759	-0.6704	-0.63145
S13	0.55993	0.36554	0.49477	0.31818	0.44067	0.53028	0.57775	0.50472	0.43814	0.08532	0.02463	0.02632	-0.01054	-0.05102	-0.05861
S14	0.54026	0.06236	0.02845	-0.0793	-0.36891	-0.66244	-0.74984	-0.63093	-0.4867	-0.43665	-0.25145	-0.11157	-0.26581	-0.37559	-0.361
S15	1.67612	0.08013	-0.27746	0.08072	1.17978	2.236	3.04664	3.37343	3.32238	2.13549	1.03161	0.49905	0.32694	0.39979	0.3044
S16	-0.34396	-0.33961	-0.3798	-0.42639	-0.58877	-0.61135	-0.61887	-0.70398	-0.69723	-0.20397	-0.32184	-0.38984	-0.3452	-0.33284	-0.34496
S17	0.96002	0.47932	0.62358	1.00756	1.56216	1.70253	1.60624	1.48837	1.12904	1.10355	0.74252	0.60527	0.76622	0.91286	0.79641
S18	0.52651	0.17374	0.22384	0.41065	0.49372	0.48517	0.53826	0.50195	0.35463	0.68016	0.46531	0.32832	0.4052	0.47913	0.32602
S19	-1.48083	-0.30355	0.07873	1.60363	1.12417	-0.76388	-1.10343	-1.32242	-1.45305	-1.08137	-0.49496	-0.12783	0.42154	0.21942	-0.03851
S20	0.72165	0.28424	0.30566	0.59822	0.76713	0.67892	0.55545	0.54931	0.49953	0.54706	0.34655	0.33598	0.41599	0.47532	0.41809
S21	0.0826	0.3166	0.2762	0.38308	0.22181	0.06935	-0.10406	-0.10999	-0.39898	-0.33536	0.10777	0.26891	0.22799	0.14713	0.22451
S22	-0.24223	-0.57192	-0.62796	-0.90134	-1.3349	-1.50127	-1.64171	-1.56607	-1.52643	-1.28877	-0.91819	-0.6951	-0.76416	-0.80654	-0.69683
S23	-0.20304	-0.32964	-0.11503	0.13245	-0.46657	-0.7678	-0.88935	-0.97421	-1.08049	-1.07916	-0.59664	-0.30324	-0.29775	-0.35229	-0.21152
S24	-0.28543	-0.14008	-0.19567	-0.27407	-0.38707	-0.44192	-0.48017	-0.40957	-0.48613	-0.55138	-0.34193	-0.15738	-0.22113	-0.21887	-0.12461
S25	1.66673	1.56845	1.15247	0.71516	0.17673	0.23107	0.5702	1.03811	1.57647	1.89315	1.75486	1.5663	1.37155	1.31846	1.32527
S26	1.49635	1.32414	1.06047	0.69136	0.36796	0.57432	0.78698	1.22096	1.66907	1.80149	1.60461	1.36864	1.27732	1.31025	1.27402
S27	-0.01454	-0.1668	-0.24075	-0.27759	-0.30448	-0.31909	-0.39721	-0.40986	-0.45184	-0.27742	-0.24978	-0.23104	-0.17641	-0.14449	-0.13242
S28	0.25875	1.31454	1.61539	2.04658	2.42149	2.21582	1.84153	1.62082	1.72623	1.60456	1.49805	1.59633	1.76374	1.80614	1.81679
S29	0.33307	0.83929	1.02827	1.50952	2.37613	2.57811	2.41978	2.2886	2.62577	1.58528	1.05556	1.17644	1.33282	1.54226	1.54746
S30	0.23269	0.07177	0.03927	0.07311	0.16446	0.2556	0.21533	0.15057	0.14823	0.49207	0.19498	0.12042	0.23779	0.36521	0.26799
S31	0.30921	0.67721	0.57282	0.40016	-0.13349	-0.44849	-0.55357	-0.50157	0.04506	0.46305	0.58119	0.72866	0.61509	0.43636	0.47481
S32	1.81522	2.29151	2.48663	2.61325	2.7253	2.54787	2.17609	2.13234	2.5843	2.88058	2.61066	2.49588	2.69605	2.95213	2.86049
S33	-0.44676	0.01219	-0.09928	-0.16992	-0.32529	-0.34818	-0.33879	-0.35002	-0.37403	-0.32996	-0.14568	-0.0343	-0.09584	-0.12956	-0.03575
S34	0.41679	-0.08031	-0.19358	-0.32226	-0.59602	-0.75963	-0.93574	-0.98076	-0.90387	-0.63367	-0.39098	-0.21299	-0.25994	-0.28964	-0.17301
S35	0.57707	1.4246	1.59926	1.28161	0.4306	-0.103	-0.35207	-0.39363	-0.206	0.66086	1.33277	1.31638	1.39072	1.41814	1.49103
S36	0.82279	1.30265	1.30015	1.20689	0.86093	0.49435	0.23443	0.21987	0.5326	0.75374	1.08521	1.29809	1.2559	1.24081	1.41792
S37	0.18747	0.0009	-0.19942	-0.33834	-0.43416	-0.34202	-0.32483	-0.25389	-0.16991	-0.1653	-0.06991	0.06848	-0.00621	0.00045	-0.03061
S38	0.7572	0.53054	0.26127	-0.01136	-0.29882	-0.26746	-0.09969	0.17287	0.55749	0.63895	0.47844	0.42911	0.29459	0.2902	0.32394
S39	-1.14527	-0.83522	-0.89965	-0.88395	-0.99337	-1.05689	-1.12259	-1.10783	-1.16795	-1.29182	-1.10804	-0.96072	-1.00533	-1.0402	-0.96987
S40	-0.08126	0.11281	0.08687	-0.04817	-0.63013	-1.00855	-1.12354	-1.0889	-0.82446	-0.4193	-0.08744	-0.00194	-0.02605	-0.04071	0.00166

S41	0.36154	0.55669	0.47412	0.27218	-0.22598	-0.50646	-0.60628	-0.59622	-0.40522	0.0122	0.29567	0.39002	0.34405	0.30564	0.41889
S42	0.76446	0.92917	0.71699	0.51767	0.15777	0.01663	0.14226	0.30451	0.62947	0.98536	0.87339	0.86275	0.7742	0.73507	0.79237
S43	-1.28704	-1.01006	-0.79993	-0.99544	-1.00388	-0.90657	-0.89888	-0.9954	-1.10927	-1.20317	-1.07583	-1.00201	-0.96652	-1.00488	-0.99949
S44	-1.117	-1.05248	-1.05305	-1.0701	-1.02469	-0.83509	-0.78497	-0.86855	-0.96553	-1.13196	-1.08253	-1.00056	-0.96218	-1.00143	-1.03394
S45	-1.18377	-0.95108	-0.89682	-1.03219	-1.1004	-0.96292	-0.92226	-0.95178	-0.95085	-0.98999	-1.04133	-1.01432	-1.01578	-1.01138	-0.98222
S46	0.36802	0.21183	0.12754	-0.00314	-0.37293	-0.59077	-0.7083	-0.67757	-0.48957	-0.21501	-0.06409	0.06217	0.02361	-0.0058	0.09932
S47	0.55261	0.69233	0.64189	0.47108	0.01169	-0.24354	-0.33059	-0.25667	-0.08521	0.31738	0.52784	0.56341	0.56637	0.54307	0.60602
S48	0.78196	0.4492	0.3917	0.42201	0.46452	0.43636	0.41962	0.36544	0.36975	0.42782	0.3601	0.3728	0.39928	0.42241	0.45809
S49	0.86686	0.87331	0.81735	0.79291	0.65141	0.65333	0.78987	0.91528	1.13298	1.1031	1.00294	0.93318	0.91921	0.94105	0.93153
S50	0.99903	1.61125	2.03243	2.10556	1.37549	0.52266	0.0687	-0.0822	0.08729	0.69085	1.39162	1.57987	1.52248	1.47875	1.64077
S51	1.86205	1.94751	1.79832	1.62716	1.07609	0.70996	0.7172	0.76779	0.94	1.62352	1.98812	1.97168	1.88934	1.78155	1.79001
S52	1.04339	2.05431	2.60252	2.79038	2.35999	1.54869	1.04475	0.69307	0.38901	1.01515	2.08172	2.32489	2.52783	2.44275	2.28537
S53	0.66265	0.96508	1.18276	0.92169	0.45168	0.01162	-0.12107	-0.05135	0.08474	0.46305	0.78314	0.84184	0.82444	0.80147	0.85384
S54	0.48742	0.56251	0.53359	0.41215	0.12966	-0.107	-0.19206	-0.19807	-0.12532	0.13011	0.3974	0.50968	0.46023	0.40968	0.46688
S55	0.63534	2.72166	2.39319	1.95161	1.02124	0.43014	0.28641	0.23672	0.34214	1.08668	1.90626	1.84304	1.88465	1.79247	1.70101
S56	0.94053	1.20773	1.16757	0.91756	0.28795	-0.00552	0.00667	0.12856	0.45982	1.022	1.28475	1.25805	1.22806	1.18185	1.11996
S57	0.76808	0.92595	0.95292	0.86501	0.63884	0.49255	0.44132	0.51022	0.64384	0.91336	0.95593	0.99279	0.98357	0.97494	0.94771
S58	-0.03161	-0.27491	-0.26098	-0.1402	-0.04076	-0.09398	-0.1905	-0.24449	-0.36498	-0.49084	-0.42693	-0.31146	-0.31732	-0.35653	-0.3709
S59	-0.28985	0.1066	0.03185	-0.09148	-0.46767	-0.71921	-0.78528	-0.78192	-0.64373	-0.31721	-0.09409	0.04906	-0.02041	-0.08943	-0.04845
S60	2.19978	1.87884	1.01906	-0.09389	-1.58026	-1.64398	-1.50278	-0.86415	0.29783	1.3257	1.43391	1.34186	0.87674	0.76068	0.84729
S61	1.19064	1.84813	2.1366	2.38878	2.61617	2.32125	2.05078	1.97361	2.03154	1.87902	1.836	2.02516	1.99653	2.03066	2.12503
S62	-0.34415	0.06755	0.09469	-0.01844	-0.4771	-0.95981	-1.18936	-1.26599	-1.31128	-0.88082	-0.28556	-0.20154	-0.17915	-0.24309	-0.20461
S63	1.67094	0.94547	0.70534	0.41113	0.15678	0.32506	0.59669	0.98699	1.61965	1.68115	1.27666	1.10094	0.97504	0.92355	0.74475
S64	0.97945	1.68055	1.86987	1.91386	1.69005	1.21765	0.92049	0.68249	0.95987	1.52015	1.6159	1.82689	1.89785	1.92117	1.90063
S65	0.44885	0.07813	0.28307	0.43309	0.66792	0.69084	0.55615	0.4845	0.26732	0.21576	0.2166	0.1355	0.224	0.29762	0.12972
S66	0.10406	0.06343	-0.0386	-0.1315	-0.39647	-0.51616	-0.52687	-0.44887	-0.09178	-0.015	-0.05102	-0.00971	-0.07592	-0.16383	-0.17827
S67	0.99128	0.10075	0.73311	0.54393	1.1354	1.22607	1.24408	1.2969	1.24924	1.0066	0.65597	0.48255	0.39414	0.26047	0.00249
S68	0.01725	0.56213	0.50555	0.35741	0.01878	-0.14676	-0.21979	-0.20854	-0.07572	0.34634	0.54776	0.51404	0.55492	0.57149	0.58416
S69	0.92604	-0.44522	-0.4506	-0.29395	0.17397	0.53389	0.69822	0.58593	0.4078	0.0128	-0.03108	-0.06564	-0.04268	-0.09465	-0.34915
S70	-0.59123	-1.04913	-1.09594	-1.07441	-0.95565	-0.79701	-0.74638	-0.76504	-0.87906	-1.1234	-1.098	-1.05659	-1.06658	-1.06233	-1.0528
S71	0.26899	-0.82068	-0.85816	-0.76322	-0.29117	-0.0141	0.10499	0.13727	-0.08876	-0.49694	-0.7022	-0.75277	-0.73291	-0.70562	-0.77186
S72	1.4737	-0.60912	-0.70509	-0.52091	0.22889	0.72685	0.87795	0.82081	0.73721	0.42388	-0.33481	-0.55551	-0.52797	-0.44534	-0.52499
S73	2.97337	0.06191	-0.7648	-0.42078	1.13088	1.6809	1.4275	1.51564	1.37123	0.12322	-0.63205	-0.76998	-0.79249	-0.64239	-0.54349
S74	0.10326	-0.88433	-0.97372	-0.99019	-0.9038	-0.6686	-0.54841	-0.45719	-0.37888	-0.64552	-0.77738	-0.799	-0.82119	-0.82758	-0.81924
S75	-1.35071	-1.19977	-1.18676	-1.23184	-1.24743	-1.18246	-1.17527	-1.20267	-1.28691	-1.48118	-1.36856	-1.25322	-1.25769	-1.26316	-1.19176
S76	-0.38732	-0.44535	-0.33319	-0.29263	-0.04951	0.15279	0.20635	0.16342	-0.11826	-0.47663	-0.31637	-0.363	-0.30363	-0.24912	-0.32992
S77	-0.84443	-1.01132	-1.09455	-1.03937	-0.76069	-0.56622	-0.47286	-0.4648	-0.5246	-0.98209	-1.07021	-1.04195	-1.04027	-1.01456	-0.96972
S78	-0.82078	-0.95394	-0.99171	-0.85832	-0.34586	0.22813	0.63222	0.46073	0.23807	-0.36208	-0.65758	-0.86378	-0.90511	-0.8788	-0.89889
S79	-0.24891	-0.84483	-0.84897	-0.83325	-0.69354	-0.54915	-0.46998	-0.45096	-0.5322	-0.45674	-0.62648	-0.70762	-0.70891	-0.7741	-0.87385
S80	0.07656	-0.60768	-0.55383	-0.50949	-0.08441	0.26142	0.57503	0.52536	0.35474	0.44415	-0.15915	-0.43054	-0.38501	-0.29209	-0.41101
S81	-1.87848	-1.18517	-1.04888	-1.17012	-1.32947	-1.29815	-1.25851	-1.48547	-1.57898	-1.50394	-1.35159	-1.25997	-1.27203	-1.3016	-1.2559
S82	0.21376	-0.73061	-0.64399	-0.3342	0.7393	1.34768	1.70067	1.61951	1.05675	0.62071	-0.05859	-0.51947	-0.49431	-0.37743	-0.47967
S83	-1.07237	-0.59504	-0.71539	-0.56918	-0.60929	-0.58143	-0.52378	-0.38329	-0.45724	-0.8106	-0.78239	-0.67924	-0.56961	-0.5733	-0.63113
S84	-2.32221	-1.19883	-1.17464	-1.35247	-1.62864	-1.64398	-1.67109	-1.6481	-1.70548	-1.87288	-1.61868	-1.46213	-1.45507	-1.50262	-1.37711

S85	-0.41938	-0.85126	-0.62177	-0.4068	0.66877	1.5531	1.19104	1.20055	0.67062	-0.31294	-0.69475	-0.78832	-0.71166	-0.64882	-0.7029
S86	-0.15519	-0.57153	-0.57157	-0.58186	-0.47298	-0.34835	-0.25306	-0.22605	-0.33478	-0.41332	-0.49023	-0.57937	-0.53508	-0.48931	-0.50489
S87	-0.83767	-0.96107	-0.9786	-0.8831	-0.37955	0.14641	0.44234	0.31941	0.22403	-0.45089	-0.79102	-0.88998	-0.90035	-0.86751	-0.88807
S88	-1.33563	-1.07995	-1.08613	-1.09283	-1.03246	-0.84121	-0.82778	-0.80105	-0.91891	-1.24408	-1.19317	-1.13271	-1.148	-1.15543	-1.10773
S89	-0.40109	-0.40002	-0.18663	-0.39284	-0.48948	-0.56533	-0.59025	-0.61242	-0.46538	-0.13657	-0.31748	-0.46031	-0.41928	-0.37497	-0.27291
S90	-1.36871	-0.86702	-0.71322	-0.16752	-0.06134	-0.41222	-0.43601	-0.33825	-0.52451	-0.60326	-0.24168	-0.63086	-0.57591	-0.62955	-0.66977
S91	-0.84633	-0.95764	-0.70435	-0.78554	-0.60217	-0.46749	-0.55702	-0.60101	-0.78736	-0.92562	-0.89029	-0.91201	-0.87922	-0.87738	-0.92546
S92	-1.14546	-1.1608	-1.13459	-1.17708	-1.05099	-0.88264	-0.71606	-0.688	-0.70147	-0.50012	-0.98712	-1.12867	-1.13166	-1.13506	-1.10481
S93	-1.00462	-0.98679	-0.96993	-0.94294	-0.64905	-0.29092	-0.13287	-0.19363	-0.32004	-0.01784	-0.61202	-0.87076	-0.87154	-0.86121	-0.92499
S94	-0.38983	-0.88013	-0.79726	-0.6388	0.27464	1.00834	1.35006	1.31047	0.98812	0.28292	-0.36003	-0.65459	-0.59318	-0.44147	-0.57663
S95	-0.83091	-1.0994	-1.13202	-1.11882	-0.90448	-0.66106	-0.57226	-0.56387	-0.62105	-1.07517	-1.13642	-1.10011	-1.0981	-1.09178	-1.06707
S96	-1.62665	-1.13386	-1.11616	-1.21471	-1.38931	-1.3311	-1.30742	-1.33959	-1.313	-1.29046	-1.18194	-1.12571	-1.15357	-1.24566	-1.23951
S97	-1.72986	-1.26082	-1.27019	-1.36466	-1.54806	-1.57465	-1.59832	-1.64701	-1.70548	-1.72709	-1.51561	-1.36905	-1.37911	-1.40894	-1.30158
S98	-0.67539	-0.88224	-1.0716	-0.90367	-0.38747	0.21084	0.65255	0.66762	0.56027	0.12922	-0.55107	-0.8238	-0.8541	-0.83651	-0.87745
S99	-1.983	-1.25518	-1.233	-1.30192	-1.40195	-1.38262	-1.37067	-1.44178	-1.54135	-1.67189	-1.48495	-1.34571	-1.36335	-1.39629	-1.29639
S100	-0.45048	-0.8044	-0.73635	-0.66399	-0.35394	-0.09205	0.07715	0.02175	-0.16087	-0.31412	-0.59384	-0.7112	-0.72848	-0.66638	-0.59503
S101	-0.84289	-0.93958	-0.90994	-0.85042	-0.50911	-0.17352	0.05103	0.0264	-0.12228	-0.48162	-0.70375	-0.80633	-0.83349	-0.82292	-0.90461
S102	-1.36277	-1.12127	-1.15389	-1.12506	-0.97658	-0.70667	-0.49366	-0.53981	-0.63026	-0.92351	-1.07534	-1.10892	-1.13875	-1.12979	-1.0957
S103	-0.22255	-0.72075	-0.68607	-0.5358	0.0196	0.4046	0.67082	0.7132	0.43477	-0.12924	-0.40432	-0.5825	-0.58185	-0.5459	-0.65651
S104	-0.70909	-0.83508	-0.76824	-0.82961	-0.6985	-0.50209	-0.42629	-0.5884	-0.70133	-0.70357	-0.783	-0.83063	-0.81361	-0.80284	-0.82194
S105	-1.01091	-0.97912	-0.91568	-0.88255	-0.48792	-0.1914	0.01155	-0.11642	-0.3164	-0.171	-0.70681	-0.87819	-0.8838	-0.89092	-0.90389
S106	-1.01501	-1.00136	-0.98532	-0.9079	-0.59434	-0.16801	0.05968	-0.11848	-0.23037	-0.58482	-0.73706	-0.91314	-0.95289	-0.98058	-0.98544
S107	0.13988	-0.72855	-0.49878	-0.31708	0.86147	1.56219	1.81404	1.91322	1.49623	0.61787	-0.07311	-0.49362	-0.48526	-0.35232	-0.45341
S108	-1.69359	-1.17319	-1.10345	-1.09902	-1.02518	-1.01856	-0.94124	-1.02898	-1.19462	-1.4169	-1.2494	-1.21241	-1.24178	-1.24923	-1.19262
S109	-0.54111	-0.60821	-0.28118	-0.28675	0.17528	0.58104	0.65393	0.65972	0.52416	0.34832	-0.18853	-0.45745	-0.46231	-0.42933	-0.52429

Sample No.	integrated bins														
	1.60 .. 1.58	1.58 .. 1.56	1.56 .. 1.54	1.54 .. 1.52	1.52 .. 1.50	1.50 .. 1.48	1.48 .. 1.46	1.46 .. 1.44	1.44 .. 1.42	1.42 .. 1.40	1.40 .. 1.38	1.38 .. 1.36	1.36 .. 1.34	1.34 .. 1.32	1.32 .. 1.30
S1	2.04571	2.36854	3.02041	3.23239	3.14881	2.9358	2.94027	3.06705	2.18441	2.16929	2.78817	2.72213	2.57291	2.61544	2.7209
S2	0.60311	0.26787	-0.32952	-0.83776	-0.84046	-0.75399	-0.78184	-0.81931	-0.68985	-0.76961	-0.52508	-0.30748	-0.28346	-0.35325	-0.45498
S3	2.44393	2.78245	3.18996	3.12143	2.90796	2.69262	2.52891	2.47223	1.93423	1.97577	2.142	1.93781	1.69597	1.59955	1.55697
S4	-0.79226	-0.74961	-0.63851	-0.61033	-0.59467	-0.60585	-0.79827	-1.08738	-1.14248	-1.1596	-0.97553	-0.88952	-0.81534	-0.72544	-0.87409
S5	0.21254	0.35518	0.83596	1.05184	1.18576	1.23987	1.21871	1.35907	0.64585	0.70216	1.21547	1.09247	0.8799	0.76978	0.86976
S6	-0.34918	-0.39625	-0.47769	-0.53886	-0.57703	-0.60351	-0.53367	-0.44135	0.01401	-0.19604	-0.53551	-0.48217	-0.52883	-0.6041	-0.70196
S7	-0.54638	-0.72454	-1.03713	-1.27684	-1.35854	-1.38457	-1.23189	-1.25164	-1.59872	-1.62693	-1.42972	-1.40244	-1.4	-1.4882	-1.63779
S8	0.99461	1.06885	1.11796	1.07032	1.01701	0.75039	0.54853	0.42416	0.4044	0.29774	0.32666	0.35813	0.25414	0.24251	0.36131
S9	1.1614	1.28161	1.57936	1.2968	1.08444	0.86006	1.3269	1.44186	1.10144	1.11474	1.26407	1.26381	1.17843	1.22323	1.01048
S10	2.83569	2.21672	0.69769	-0.13116	0.00124	0.04814	0.14222	0.23264	0.66985	0.36963	0.56054	0.76142	0.82542	0.87081	1.0103
S11	-0.81738	-0.91925	-1.19982	-1.37666	-1.3747	-1.52174	-1.50184	-1.563	-1.80643	-1.82388	-1.44966	-1.37855	-1.35799	-1.28183	-1.55756
S12	-0.58327	-0.59954	-0.58183	-0.48167	-0.3658	-0.36046	-0.43215	-0.41833	-0.63266	-0.75685	-0.63759	-0.67548	-0.75579	-0.70881	-0.60526
S13	-0.03515	0.05094	0.30077	0.45193	0.61042	0.71077	0.7169	0.56109	0.46054	0.62062	0.59326	0.30182	0.0538	0.13267	0.45936
S14	-0.39969	-0.51321	-0.62891	-0.54229	-0.47513	-0.31078	-0.06075	0.15271	-0.62331	-0.88521	-0.21161	-0.38216	-0.44875	-0.48753	-0.39672
S15	0.21136	0.38202	1.21016	1.73221	1.99395	2.0484	2.63669	2.25355	1.34227	1.11583	2.04044	1.99592	1.79148	1.65857	1.74592
S16	-0.3598	-0.38149	-0.50817	-0.54746	-0.5191	-0.52142	-0.60574	-0.61655	-0.27298	-0.44964	-0.4992	-0.34712	-0.37069	-0.45727	-0.63506

S17	0.84151	1.12474	1.6064	1.70875	1.61317	1.43894	1.40201	1.39303	1.35369	1.67533	1.68906	1.71345	1.67927	1.65819	1.44254
S18	0.25875	0.3132	0.38138	0.28768	0.30825	0.2443	0.26073	0.47577	0.67189	0.65834	0.89437	1.06182	1.03146	0.8468	0.55884
S19	0.35065	0.50898	0.13387	-0.58028	-1.14255	-1.00679	-1.16911	-0.9575	-1.70234	1.13601	0.16666	-0.54585	-0.34628	-0.17551	-0.70751
S20	0.4284	0.50791	0.64861	0.4855	0.37741	0.19354	0.10504	0.14236	0.48909	0.55712	0.42243	0.40602	0.40158	0.27758	-0.06262
S21	0.23953	0.0943	-0.38522	-1.08593	-1.33343	-1.41269	-1.50184	-1.563	-1.76293	-1.87662	-1.69362	-1.75681	-1.78219	-1.59938	-1.63779
S22	-0.69195	-0.85237	-1.15398	-1.28211	-1.26403	-1.30421	-1.22831	-1.14558	-0.95646	-1.18042	-1.14185	-1.09552	-1.14269	-1.11487	-1.16741
S23	-0.13861	-0.20848	-0.32783	-0.74056	-0.93868	-0.98231	-1.02041	-0.97919	-0.54559	-0.44857	-0.96973	-0.98013	-0.94771	-0.88168	-1.13297
S24	-0.11007	-0.19138	-0.29244	-0.32013	-0.33372	-0.49233	-0.52144	-0.52152	-0.65117	-0.7904	-0.57312	-0.49072	-0.52143	-0.57956	-0.73546
S25	1.17932	0.89777	0.40455	0.31619	0.73043	0.86037	1.03233	1.33869	1.68215	1.16537	1.19247	1.30419	1.16499	1.195	1.60047
S26	1.17674	1.07425	0.78807	0.88094	1.23395	1.3087	1.48317	1.92749	2.00807	1.52094	1.63616	1.70578	1.68618	1.67237	1.94508
S27	-0.14642	-0.16735	-0.30083	-0.43977	-0.44857	-0.57092	-0.71752	-0.81097	0.4212	0.25717	-0.61571	-0.51165	-0.55106	-0.67897	-0.97118
S28	2.00741	2.31997	2.64825	2.57563	2.43898	2.06994	1.78792	1.74298	1.97915	2.08007	1.8633	1.61652	1.51396	1.42185	1.36296
S29	1.67105	1.98849	2.70721	2.91585	2.63653	2.11472	1.88248	1.8233	1.69967	1.96009	2.26513	2.60467	2.79694	2.76564	2.54465
S30	0.22462	0.27515	0.34133	0.24984	0.09301	-0.06318	-0.05665	-0.00383	0.43806	0.35633	0.44056	0.76369	0.8658	0.71895	0.31174
S31	0.48805	0.31381	-0.20039	-0.51582	-0.29096	-0.27764	-0.52567	-0.40547	0.6503	0.44081	-0.47977	-0.64686	-0.95656	-1.0713	-1.06912
S32	2.85681	3.01106	2.99006	2.52182	2.02772	1.45121	1.27364	1.26099	0.94825	0.87279	1.23474	1.5719	1.76162	1.443	0.47268
S33	-0.00889	-0.08101	-0.22617	-0.25569	-0.22309	-0.17759	-0.27436	-0.36236	-0.46214	-0.6465	-0.50673	-0.55754	-0.67333	-0.65035	-0.65656
S34	-0.15271	-0.26928	-0.52928	-0.67038	-0.73956	-0.86976	-0.96045	-0.97079	-0.21789	-0.39916	-0.92669	-0.94118	-0.94749	-0.94902	-1.13314
S35	1.52156	1.35771	0.57763	-0.13466	-0.35821	-0.55445	-0.64446	-0.66489	0.06288	-0.03756	-0.41129	-0.21209	-0.14233	-0.22356	-0.57901
S36	1.50458	1.41363	1.12205	0.74299	0.63863	0.38629	0.34121	0.31437	0.37707	0.27623	0.33076	0.40821	0.39269	0.41092	0.26654
S37	-0.11255	-0.17777	-0.10962	-0.09887	0.02717	0.09013	0.19866	0.42961	-0.06646	-0.27452	0.5797	0.8762	0.90253	0.73598	0.56817
S38	0.23475	0.10451	-0.01869	0.16354	0.44918	0.55668	0.70549	0.98894	1.36804	0.84715	0.86281	0.99432	0.90941	1.02855	1.47924
S39	-0.93066	-0.96998	-1.03038	-1.01738	-0.92461	-1.0082	-0.97079	-1.0259	-1.06979	-1.21049	-1.19705	-1.26392	-1.33559	-1.29006	-1.33855
S40	-0.03365	-0.21864	-0.6965	-1.18042	-1.355	-1.33837	-1.19858	-1.10765	-0.27483	-0.42428	-0.94477	-0.81744	-0.79123	-0.72622	-0.79271
S41	0.40781	0.23886	-0.24063	-0.55608	-0.63536	-0.74105	-0.58302	-0.50847	0.53105	0.2855	-0.61917	-0.50795	-0.5496	-0.582	-0.69746
S42	0.73952	0.60212	0.2614	0.06876	0.07804	0.06419	0.47178	0.33159	1.06741	0.73658	0.18813	0.23715	0.17353	0.13317	0.11277
S43	-0.99285	-0.99443	-1.01919	-0.99724	-0.88482	-0.7829	-0.89795	-1.08439	-1.14309	-1.21126	-1.02342	-1.09398	-1.23981	-1.1597	-1.19734
S44	-1.02529	-1.00094	-0.95327	-0.90079	-0.74675	-0.59101	-0.73001	-0.77407	-0.94122	-1.06345	-0.7443	-0.78581	-0.84687	-0.78904	-0.68585
S45	-0.96122	-0.95855	-0.85841	-0.72433	-0.50154	-0.41626	-0.41851	-0.48946	-1.24464	-0.61772	-0.60948	-0.59471	-0.52401	-0.27621	0.03956
S46	0.10246	-0.02836	-0.40275	-0.65455	-0.74453	-0.83295	-0.70521	-0.61972	0.19062	0.0106	-0.75446	-0.64262	-0.68465	-0.69527	-0.89948
S47	0.58233	0.4722	-0.02271	-0.34293	-0.44498	-0.55749	-0.35145	-0.36001	0.2269	0.02545	-0.43031	-0.34659	-0.43349	-0.46608	-0.745
S48	0.48027	0.51833	0.51806	0.55168	0.41719	0.21785	0.57253	0.39018	1.02022	0.93593	0.33265	0.38305	0.31216	0.30128	0.18926
S49	0.91534	0.91434	0.76291	0.73594	0.67274	0.63896	0.78122	0.90536	0.8469	0.64794	0.71129	0.72646	0.62961	0.62612	0.55606
S50	1.67425	1.47393	0.79971	-0.01963	-0.47164	-0.70249	-0.79781	-0.795	-0.1384	-0.08663	-0.72317	-0.6856	-0.62613	-0.67809	-0.99267
S51	1.743	1.60409	1.06844	0.66752	0.57285	0.57284	1.15221	1.02817	0.98554	0.76027	0.79955	0.85106	0.65771	0.46018	0.26111
S52	2.34165	2.48328	2.28072	1.78849	1.61485	1.60382	1.45993	1.85226	2.50532	2.55235	1.88299	1.75564	1.4941	1.08293	0.83989
S53	0.83281	0.70398	0.25646	-0.0688	-0.24229	-0.31752	-0.2111	-0.11567	0.4932	1.11347	-0.13922	-0.07058	-0.09512	-0.17772	-0.54511
S54	0.47037	0.38735	0.14182	-0.02287	-0.05031	-0.09345	-0.11909	-0.02984	0.26615	0.04589	-0.12538	-0.09464	-0.20292	-0.28449	-0.39453
S55	1.62911	1.57756	1.00619	0.45305	0.48109	0.51818	0.6288	0.83291	0.85655	1.27497	1.34938	1.15006	0.9482	0.83705	0.54036
S56	1.00093	0.81954	0.28085	-0.08955	0.01892	0.12355	0.24593	0.53562	0.56043	0.37791	0.82075	0.96359	0.91334	0.71977	0.58237
S57	0.90049	0.8473	0.63017	0.53194	0.52935	0.50083	0.57713	0.69347	0.26624	0.69896	0.73281	0.7977	0.75535	0.58159	0.36263
S58	-0.3606	-0.34337	-0.26612	-0.19597	-0.14709	-0.17086	-0.17606	-0.19904	0.02429	-0.06117	-0.19555	-0.30943	-0.53526	-0.58806	-0.71764
S59	-0.06777	-0.22864	-0.52291	-0.74012	-0.74967	-0.79737	-0.7495	-0.57819	-1.07757	-0.53969	-0.64936	-0.59271	-0.6461	-0.75722	-1.02295
S60	0.45109	-0.40675	-1.4368	-1.37666	-1.3747	-1.52174	-1.3904	-0.82103	0.9683	-0.05446	-1.04554	-0.49888	-0.59144	-0.90934	-1.15529

S61	2.23775	2.38976	2.61236	2.63432	2.49024	2.10857	2.09721	2.25318	1.5891	1.49449	1.84093	1.90524	1.83782	1.71069	1.62412
S62	-0.22733	-0.38384	-0.96278	-1.37666	-1.3747	-1.52174	-1.50184	-1.563	-0.15297	-0.34434	-1.69362	-1.75681	-1.78219	-1.59938	-1.63779
S63	0.57857	0.43426	0.21939	0.35048	0.76716	1.03229	1.12816	1.53285	1.30907	0.92691	1.52681	1.60255	1.25202	0.96402	1.16966
S64	1.88189	1.87254	1.70175	1.32234	0.95875	0.76233	1.23946	1.31081	1.62803	1.6923	1.73458	2.01837	2.17973	1.95595	1.5476
S65	0.0637	0.08909	0.09872	-0.15592	-0.233	-0.27827	-0.5204	-0.37332	0.15426	0.08981	-0.22847	-0.23443	-0.48335	-0.80379	-1.22699
S66	-0.22739	-0.32262	-0.47436	-0.47857	-0.35536	-0.20407	-0.06877	0.12619	0.54308	0.28595	0.06075	0.11007	-0.13723	-0.18157	0.0631
S67	-0.08192	0.03611	0.40623	0.70794	1.16597	1.72932	1.65285	1.70672	1.48658	1.32964	1.54164	0.92055	0.14531	-0.13181	-0.01441
S68	0.57582	0.53712	0.14731	-0.19072	-0.29151	-0.38149	-0.42016	-0.42957	0.04366	-0.0758	-0.32008	-0.1804	-0.1432	-0.33903	-0.55785
S69	-0.40105	-0.29371	0.08426	0.32451	0.44739	0.93384	0.89834	1.12961	1.32771	1.2355	1.19048	1.10995	1.18493	0.8643	0.69476
S70	-1.02051	-1.01065	-0.91843	-0.75276	-0.75221	-0.71859	-0.3753	-0.66028	-0.95096	-1.10982	-0.88713	-0.85891	-0.81744	-0.77553	-0.68314
S71	-0.75832	-0.67667	-0.35909	-0.07073	0.01343	0.12771	0.10966	0.08272	-0.39199	-0.53314	-0.08007	-0.04588	-0.02469	0.03756	0.11976
S72	-0.53746	-0.39356	0.18172	0.54842	0.64625	0.75718	0.65675	0.55357	0.60816	0.69843	0.59837	0.6081	0.73185	0.83319	0.96446
S73	-0.56442	-0.33209	0.70742	1.53421	2.57502	3.46013	2.72256	1.18589	1.06463	1.43895	0.95434	0.66116	0.22349	0.50301	0.69366
S74	-0.81701	-0.76267	-0.47437	-0.14149	0.1299	0.32715	0.8125	0.61443	0.19432	-0.24159	0.19281	0.28358	0.21285	0.47066	1.43188
S75	-1.15688	-1.16869	-1.17082	-1.09656	-1.04619	-1.05448	-1.08985	-1.18408	-1.52698	-1.67023	-1.32913	-1.30914	-1.25939	-1.17779	-1.12003
S76	-0.29138	-0.14237	-0.01815	-0.07479	-0.12606	0.1416	0.37472	0.53037	-0.32928	-0.44206	0.01672	0.05666	0.33279	0.53059	0.42982
S77	-0.96178	-0.89419	-0.61798	-0.35645	-0.30628	-0.20849	-0.24689	-0.38488	-0.91633	-1.11186	-0.66827	-0.6259	-0.58535	-0.50836	-0.29289
S78	-0.88343	-0.75556	-0.31311	0.02118	0.06135	0.10233	-0.1485	-0.29202	-0.75733	-0.62252	-0.30882	-0.41198	-0.49893	-0.46917	0.79291
S79	-0.8787	-0.86169	-0.73306	-0.60029	-0.39147	-0.11054	-0.09472	-0.07859	0.0081	-0.16672	-0.18871	-0.17894	-0.18538	-0.33335	-0.17594
S80	-0.46699	-0.35872	-0.00378	0.21725	0.18994	0.49674	0.55431	0.46248	0.19484	0.10036	0.42376	0.52226	0.75685	0.74554	0.67161
S81	-1.22719	-1.30466	-1.4368	-1.37666	-1.3747	-1.52174	-1.50184	-1.563	-1.28853	-1.38359	-1.63333	-1.56228	-1.38504	-1.44808	-1.53563
S82	-0.44489	-0.18419	0.54875	0.93933	0.99076	0.91716	0.81366	0.50121	0.08895	0.51735	0.78126	0.27618	0.96351	1.31349	0.77804
S83	-0.57416	-0.50241	-0.24416	0.16143	0.14485	0.42016	0.56595	0.87519	-0.31446	1.88115	0.96451	0.64878	1.97552	3.34809	1.63931
S84	-1.3211	-1.36001	-1.4368	-1.37666	-1.3747	-1.52174	-1.50184	-1.563	-2.12688	-0.94473	-1.69362	-1.75681	-1.78219	-1.59938	-1.63779
S85	-0.628	-0.44813	-0.06802	0.24375	0.13058	0.24822	0.24971	-0.16598	0.02068	0.38373	0.3921	0.24857	0.4451	0.76657	1.79259
S86	-0.51174	-0.46746	-0.39405	-0.24409	-0.26795	-0.175	-0.32004	-0.28659	-0.48806	-0.65138	-0.43662	-0.39591	-0.32958	-0.25537	-0.03511
S87	-0.86843	-0.76101	-0.34545	0.06742	0.33169	0.38542	0.40372	0.33474	-0.1599	-0.37634	0.01282	-0.07926	-0.10839	0.13107	0.85362
S88	-1.08118	-1.10232	-1.0834	-1.00364	-0.99544	-1.03864	-1.02912	-1.16311	-1.23306	-1.33409	-1.23727	-1.19475	-1.25967	-1.1817	-0.5514
S89	-0.39778	-0.37934	-0.22119	-0.00482	-0.41918	0.04632	-0.48624	-0.55658	-0.28398	-0.48648	-0.67549	-0.71227	-0.46662	-0.48312	-0.57528
S90	-0.51548	-0.42187	-0.3376	-0.22309	-0.46383	-0.51674	-0.39791	-0.50295	-1.48583	1.02084	-0.47464	-0.87838	-0.64602	-0.24021	-0.73186
S91	-0.92097	-0.89209	-0.82352	-0.9186	-0.94122	-0.75999	-0.78408	-0.7974	-0.54229	-0.51403	-0.65724	-0.68104	-0.65497	-0.57656	-0.41898
S92	-1.07953	-1.03758	-0.85114	-0.61105	-0.61617	-0.61822	-0.63632	-0.59256	-0.91171	-1.08716	-0.71509	-0.70603	-0.55452	-0.4907	-0.11625
S93	-0.92715	-0.85589	-0.61851	-0.37648	-0.25843	-0.35833	-0.40554	-0.46108	-0.33549	-0.43005	-0.37251	-0.40072	-0.04097	-0.22129	-0.32207
S94	-0.58099	-0.39881	0.24043	0.76659	0.81761	0.68546	0.65354	0.56077	-0.36454	-0.35358	0.65738	0.50403	0.79572	0.97522	0.95291
S95	-1.05476	-1.01558	-0.78223	-0.57011	-0.55679	-0.52981	-0.55928	-0.62587	-0.84026	-1.00223	-0.67346	-0.77239	-0.42725	-0.53736	0.47152
S96	-1.22323	-1.27831	-1.41338	-1.37666	-1.28638	-1.14517	-1.2041	-1.18517	-1.20962	-1.32398	-1.10772	-1.10333	-1.15086	-1.19805	-1.0521
S97	-1.27682	-1.33706	-1.4368	-1.37666	-1.3747	-1.52174	-1.50184	-1.563	-2.02222	-2.19805	-1.69362	-1.75681	-1.76731	-1.59938	-1.63779
S98	-0.8699	-0.73239	-0.24788	0.04778	0.29157	0.10133	0.1166	0.02629	-0.29516	-0.38366	0.20334	-0.04426	-0.20909	-0.09922	0.30647
S99	-1.25674	-1.30026	-1.39898	-1.29972	-1.3747	-1.35707	-1.50184	-1.563	-1.90252	-2.02215	-1.69362	-1.75681	-1.78219	-1.59938	-1.63779
S100	-0.77679	-0.70567	-0.31481	0.7233	0.10246	0.63706	0.24225	0.20048	0.59932	0.44006	-0.02788	-0.16488	-0.16289	-0.16848	0.02252
S101	-0.90913	-0.86645	-0.67671	-0.47195	-0.42864	-0.16756	-0.00151	-0.12461	-0.58164	-0.71766	-0.35319	-0.41189	-0.27815	-0.32993	-0.27352
S102	-1.08062	-1.04272	-0.90973	-0.65151	-0.58415	-0.5798	-0.6335	-0.69066	-0.71606	-0.85123	-0.84471	-0.86059	-0.34519	-0.53895	-0.55288
S103	-0.69253	-0.55843	-0.16995	0.15222	0.18292	0.15535	0.23915	0.32203	-0.02458	-0.05579	0.3076	0.47411	0.42263	0.42211	0.91009
S104	-0.82977	-0.7902	-0.69636	-0.58119	-0.47227	-0.22437	-0.39016	-0.43497	-0.25425	-0.41124	-0.56249	-0.53384	-0.48914	-0.47092	-0.29222

S105	-0.92078	-0.84862	-0.48066	-0.04611	-0.0959	-0.14715	-0.35168	-0.43263	0.08356	0.09541	-0.1625	-0.23637	-0.14331	-0.14967	0.45205
S106	-0.96062	-0.87598	-0.60592	-0.29452	-0.26591	-0.08732	-0.27791	-0.40122	-0.68354	-0.57539	-0.45002	-0.55053	-0.64557	-0.58143	0.76719
S107	-0.47659	-0.19965	0.65457	1.22774	1.21413	1.24278	1.1877	1.03811	0.41116	0.62767	1.12124	0.79653	1.17031	1.56014	1.38857
S108	-1.148	-1.14356	-1.01991	-0.8244	-0.84773	-0.8162	-1.0289	-1.00521	-1.16165	-0.95002	-0.70336	-1.04203	-1.14042	-1.0143	-0.2606
S109	-0.55294	-0.41434	-0.02885	0.41842	0.36072	0.64806	0.50416	0.5241	0.44143	0.58462	0.53967	0.3244	0.42707	0.38907	0.74683

Sample No.	integrated bins														
	1.30 .. 1.28	1.28 .. 1.26	1.26 .. 1.24	1.24 .. 1.22	1.22 .. 1.20	1.20 .. 1.18	1.18 .. 1.16	1.00 .. 0.98	0.98 .. 0.96	0.96 .. 0.94	0.94 .. 0.92	0.92 .. 0.90	0.90 .. 0.88	0.88 .. 0.86	0.86 .. 0.84
S1	1.61198	0.99754	0.92631	1.03314	1.07866	0.84354	1.19886	-0.11857	0.40991	0.54102	0.71335	0.96135	1.42882	1.71339	1.86789
S2	-0.53379	-0.17974	0.25293	-0.00202	-0.10923	0.05896	0.4281	-0.20404	-1.00959	-1.11499	-0.96576	-0.50396	-0.32131	-0.38456	-0.58733
S3	1.04878	0.65941	0.37787	0.43525	0.50522	0.53139	0.35571	0.0063	0.64519	0.63651	0.63587	0.91258	1.00622	0.87437	1.08192
S4	-0.69213	-0.5113	0.00678	-0.49286	-0.87616	-0.83497	-0.76986	1.35827	1.31144	1.41614	1.18121	0.90743	0.6985	0.90306	0.96643
S5	0.5038	0.62658	0.24545	0.49697	0.53679	0.59168	0.72293	-0.54781	-0.08543	-0.15364	0.01723	0.11235	0.22579	0.18363	0.24108
S6	-0.63682	-0.42902	-0.76468	-0.72814	-0.67011	-0.71207	-0.70852	-0.06295	-0.21272	-0.19439	-0.15545	-0.14067	-0.154	-0.21791	-0.13185
S7	-1.4418	-0.95229	-1.43351	-1.37016	-1.06891	-1.10761	-0.76986	-0.20294	-0.51169	-0.52836	-0.63512	-0.78987	-0.82874	-0.88025	-0.83705
S8	-0.06165	-0.34992	-0.57114	-0.36795	-0.42233	-0.53039	-0.39782	-0.12776	0.10728	0.06718	0.07378	0.09081	0.12844	0.26448	0.27942
S9	0.43573	0.02151	0.16589	0.29695	0.31072	0.41795	-0.00415	-0.1757	0.24734	0.28503	0.42503	0.38553	0.86947	1.12734	1.17646
S10	0.61736	0.64815	1.41186	1.29264	1.44762	1.47251	2.75455	-0.63539	-1.44363	-1.56941	-1.3837	-0.76055	-0.235	-0.27374	-0.63803
S11	-1.4474	-1.28266	-1.14577	-1.07585	-1.06579	-0.93901	-0.76986	0.71313	0.73532	0.5868	0.2935	-0.13002	-0.24976	-0.115	0.02184
S12	-0.47593	-0.28885	-0.62693	-0.32367	-0.27798	-0.37296	-0.57406	-0.45022	-0.32139	-0.31245	-0.3727	-0.57958	-0.78617	-0.80083	-0.84287
S13	0.4567	0.96117	1.21888	1.39532	0.6486	0.76108	-0.1222	0.12253	0.26493	0.22933	0.37681	0.0743	-0.03395	-0.03446	-0.057
S14	-0.33729	0.08482	-0.55576	-0.41009	-0.1271	-0.26666	0.12441	-0.75979	-0.78791	-0.7056	-0.72279	-0.61436	-0.74128	-0.87356	-0.88746
S15	1.12224	0.75246	1.27079	0.86355	0.77044	0.6113	0.07206	0.79979	1.0007	1.13657	1.1623	1.25524	1.0014	0.9045	1.09137
S16	-0.66182	-0.49476	-0.66799	-0.61443	-0.79921	-0.73874	-0.65887	0.00421	-0.16752	-0.16042	-0.11348	0.04114	-0.02067	-0.09211	-0.22121
S17	0.74323	0.45599	0.0479	0.28705	0.35503	0.42677	0.47473	-0.27585	0.21143	0.21762	0.32234	0.57908	1.14933	1.44446	1.44985
S18	0.02874	-0.24236	-0.38446	-0.28918	-0.20147	0.05216	0.0009	-0.10399	0.10899	0.04579	0.22287	0.64472	1.13973	1.23562	1.02054
S19	-0.73564	-0.76383	-0.73694	-0.35679	-0.76794	-0.56351	-0.13643	-0.79513	-0.31774	-0.86792	-0.85187	-0.60469	0.18586	0.59221	0.71884
S20	-0.44025	-0.50091	-0.73674	-0.81835	-1.04832	-0.96956	-0.76986	0.99851	0.93279	0.7845	0.76011	0.79355	0.96332	1.06449	1.0969
S21	-1.52491	-1.60373	-0.71382	-1.37016	-1.06891	-1.10761	-0.76986	5.49272	5.46984	4.94538	4.3378	2.78142	1.6328	1.30081	1.47942
S22	-1.06937	-0.71516	-1.0931	-0.85752	-0.5795	-0.59907	-0.07229	-1.1232	-1.21068	-1.09297	-1.13136	-1.27504	-1.27717	-1.2671	-1.25248
S23	-1.15448	-1.16198	-0.58772	-0.98739	-1.06891	-1.10761	-0.76986	-0.23645	-0.12783	-0.54049	-0.50684	-0.93648	-0.76927	-0.49612	-0.45471
S24	-0.86643	-0.88351	-1.01898	-0.78386	-0.81406	-0.75282	-0.55902	-0.45048	-0.41365	-0.40424	-0.44385	-0.39674	-0.39077	-0.45512	-0.41634
S25	1.11868	0.72244	0.87206	1.1411	1.65563	1.58046	2.47247	-0.92707	-1.12422	-1.10653	-0.73183	-0.18521	-0.03753	-0.21202	-0.4124
S26	1.32878	1.03149	1.59907	1.80393	2.20886	2.09294	3.62666	-0.59602	-1.07356	-0.84061	-0.42029	0.04082	0.13137	-0.0388	-0.23594
S27	-1.23204	-0.96803	-0.85621	-1.19318	-1.06891	-1.10761	-0.76986	1.3912	0.73775	0.59665	0.51465	0.53191	0.44529	0.34325	0.29427
S28	0.68358	0.38312	-0.15656	-0.05826	0.05026	0.03777	-0.04806	0.25136	0.72706	0.73063	0.91562	0.93806	1.07879	1.12943	1.27508
S29	1.07979	0.20139	0.03018	0.1257	0.13272	0.19784	0.33474	0.01475	0.3696	0.28556	0.22226	0.4396	1.30243	1.97164	2.06489
S30	-0.34937	-0.46317	-0.30222	-0.46735	-0.99471	-0.80775	-0.76986	1.23649	0.82518	0.90789	1.07788	1.59898	2.10945	2.24452	1.96693
S31	-0.86666	-0.14273	-0.63914	-0.70613	-0.91874	-0.98543	-0.76986	1.17624	0.59707	0.45143	0.6447	0.6536	0.07038	-0.36274	-0.46994
S32	-0.61207	-0.80356	-0.62863	-0.96522	-1.03158	-0.56042	-0.17078	3.20185	3.56194	3.66978	3.76792	3.65579	4.06326	4.39288	4.55919
S33	-0.66304	-0.62297	-0.7382	-0.62301	-0.5205	-0.59368	-0.6099	-0.37965	-0.34693	-0.34338	-0.41474	-0.58033	-0.77211	-0.79745	-0.78072
S34	-1.14887	-0.90771	-1.13732	-1.19984	-1.06891	-1.07952	-0.76986	-0.0038	-0.29987	-0.30065	-0.34466	-0.30493	-0.38498	-0.45098	-0.47871
S35	-0.9135	-0.86465	-0.7819	-1.03994	-1.06891	-0.98637	-0.76986	0.30387	0.01518	-0.12083	-0.03443	0.12139	0.48624	0.60402	0.46879
S36	-0.24132	-0.40834	-0.62912	-0.44283	-0.19336	-0.17084	0.02692	-0.7266	-0.41738	-0.30096	-0.16171	-0.00312	0.28358	0.33182	0.23157

S37	0.07867	-0.24818	-0.43091	-0.23397	0.02662	0.00488	0.81249	-0.8849	-0.66977	-0.50589	-0.22324	0.80329	1.1775	0.87465	0.60051
S38	1.09197	0.775	1.26204	1.54472	1.89147	1.80455	3.58789	-1.54684	-1.44363	-1.56941	-1.62519	-1.09201	-0.87705	-0.99037	-1.15189
S39	-1.19085	-0.89897	-1.2903	-1.04729	-1.04452	-1.09857	-0.76986	-0.66257	-0.68721	-0.664	-0.88458	-1.07736	-1.34091	-1.3652	-1.32594
S40	-0.76868	-0.55328	0.07307	0.03008	0.29927	0.65892	1.96626	1.07966	0.83727	0.65012	0.57265	0.27095	0.17826	0.15418	0.17255
S41	-0.82544	-0.72873	-1.01713	-0.9845	-0.93392	-0.80011	-0.76986	-0.41345	-0.59958	-0.65752	-0.60581	-0.53132	-0.36238	-0.30526	-0.37088
S42	-0.08292	-0.24755	-0.51858	-0.35254	-0.08964	-0.08383	0.05953	-0.76631	-0.59486	-0.57577	-0.44657	-0.10051	-0.00885	-0.07764	-0.12744
S43	-1.08443	-0.55565	-0.55921	-0.27847	-0.41009	-0.43119	-0.76986	0.14346	0.26195	0.2008	0.09076	-0.1677	-0.53006	-0.78238	-0.72123
S44	-0.62481	-0.59384	-0.53153	-0.40988	-0.32438	-0.41263	-0.60124	-0.36553	-0.34839	-0.40237	-0.485	-0.42532	-0.60594	-0.85669	-0.81934
S45	-0.06836	0.03266	0.44108	0.69415	0.54641	0.56543	1.34152	-1.11216	-1.44363	-1.51605	-1.55216	-1.45681	-1.40992	-1.52422	-1.54079
S46	-1.00406	-0.85545	-1.14552	-1.16555	-1.03275	-0.98565	-0.76986	-0.31333	-0.55516	-0.58596	-0.57618	-0.53126	-0.36921	-0.29183	-0.37484
S47	-0.94546	-0.79212	-0.80435	-0.95528	-1.06891	-0.96533	-0.76986	0.42817	0.04803	0.01381	0.07143	0.01989	0.09242	0.11371	0.04913
S48	-0.21981	-0.30887	-0.59072	-0.49943	-0.40627	-0.43156	-0.48753	-0.21833	-0.0378	0.05662	0.114	0.13998	0.29169	0.38839	0.37077
S49	0.04379	-0.05614	0.02324	-0.0796	-0.09576	-0.05942	0.04896	0.22358	0.18177	0.27728	0.36252	0.3541	0.35362	0.32481	0.28651
S50	-0.94862	-0.53227	-0.45179	-0.34147	-0.5288	-0.63338	-0.41068	1.11246	1.17842	1.0609	0.93987	0.4595	0.26643	0.2577	0.34594
S51	-0.04142	0.10107	0.01061	-0.05934	-0.23883	-0.16646	-0.53818	0.41638	0.46177	0.55119	0.7094	1.10474	1.07784	0.84926	0.75083
S52	0.54007	0.74444	0.20892	0.33192	-0.01005	0.12474	-0.36391	0.6018	1.19268	1.19246	1.68642	2.3088	2.57336	2.28385	2.35541
S53	-0.71381	-0.07664	-0.32997	-0.50803	-0.7682	-0.66024	-0.76986	0.52166	0.27302	0.39793	0.41201	0.50248	0.53278	0.33311	0.18488
S54	-0.54589	-0.44829	-0.65393	-0.7533	-0.8617	-0.75669	-0.6948	0.16848	0.00844	-0.00421	0.05427	0.17616	0.2609	0.14268	0.11481
S55	-0.04909	0.47258	0.64539	0.37873	0.0583	0.04374	0.19073	-0.09493	0.15204	0.18471	0.48304	0.7312	1.04085	1.00559	0.80303
S56	0.15033	0.23277	0.3596	0.4084	0.36729	0.46438	1.0781	-0.21416	-0.57223	-0.56668	-0.27135	0.24961	0.57967	0.46906	0.20672
S57	-0.08594	-0.02503	-0.32763	-0.18059	-0.21525	-0.2458	-0.10713	0.01791	0.14623	0.20346	0.3379	0.62117	0.6795	0.52671	0.42856
S58	-0.70109	-0.19533	-0.89191	-0.59278	-0.81807	-0.91571	-0.76986	-0.08924	0.17424	0.25988	0.14699	0.16661	-0.1623	-0.38567	-0.41687
S59	-1.08199	-0.49212	-0.93689	-0.95977	-1.02523	-1.08503	-0.76986	-0.03153	-0.37082	-0.35981	-0.39758	-0.25715	-0.20025	-0.23137	-0.24373
S60	-1.03899	-0.95138	-1.44809	-0.98933	-0.44485	-0.39479	0.07732	-1.28543	-0.91485	-0.70665	-0.27921	0.44888	0.2254	-0.43172	-0.87262
S61	0.76135	0.48861	0.13934	0.21561	0.1243	-0.05704	0.02137	0.35427	0.77905	0.92441	1.05204	1.0517	1.35997	1.55354	1.67662
S62	-1.52491	-1.36225	-1.47909	-1.37016	-1.06891	-1.10761	-0.76986	3.07762	2.29026	2.25081	2.13348	1.39904	0.59081	0.30027	0.37676
S63	0.92389	0.70863	0.2022	0.64011	0.68711	0.5177	1.13224	-0.29543	0.16819	0.47451	0.95899	1.59293	1.17238	0.66134	0.28141
S64	0.55863	0.7801	0.20522	0.33303	0.25899	0.34171	0.81835	-0.90279	-0.45424	-0.33088	0.01552	0.9713	1.88607	2.04591	1.84584
S65	-1.16501	-0.29775	-0.45873	-1.18571	-1.06891	-1.10761	-0.76986	3.74753	3.73524	3.4656	3.23876	2.64738	2.07767	1.868	2.08761
S66	0.11061	1.56014	0.13903	-0.07521	-0.18706	-0.14905	0.47273	-0.47024	-1.00002	-1.12714	-0.9885	-0.57968	-0.50584	-0.58521	-0.7024
S67	0.06695	6.99034	2.41781	0.80872	0.14539	-0.14859	-0.61541	2.19861	2.44309	2.78837	3.05076	2.75766	1.54083	1.0763	1.06573
S68	-0.75853	-0.77464	-0.78952	-0.97671	-0.84755	-0.67224	-0.76986	0.34258	0.04875	-0.03592	-0.02015	0.13592	0.25097	0.26269	0.19177
S69	1.04262	1.33968	1.68183	1.51612	1.30022	1.19103	-0.05729	1.1366	0.94253	1.21571	1.35925	1.72853	1.46137	1.25815	0.97288
S70	-0.48228	-0.44766	-0.44932	-0.55887	-0.41053	-0.43231	-0.76986	-0.36698	-0.53384	-0.51797	-0.65778	-0.91623	-0.98551	-0.89661	-0.85177
S71	0.32556	0.0987	0.21191	0.3035	0.4556	0.64451	0.1474	-0.585	-0.37019	-0.32	-0.31486	-0.3825	-0.36564	-0.15564	-0.19664
S72	0.91103	0.69319	0.92062	0.96153	0.99051	1.25956	0.45687	-0.32251	-0.00148	0.13343	0.03501	0.07417	0.19023	0.56819	0.60517
S73	1.0195	1.5995	4.12568	3.63522	2.63328	2.49518	2.06621	-0.3233	0.42965	1.18611	0.39254	0.99957	0.51215	0.6882	0.88163
S74	1.31818	0.7859	1.04411	1.25566	1.72373	1.61812	2.0921	-1.55637	-1.44363	-1.47137	-1.34117	-1.29486	-1.17894	-1.1272	-1.29962
S75	-0.9445	-0.82219	-0.84734	-0.85581	-0.53498	-0.65874	-0.76986	-0.74087	-0.91186	-0.9224	-1.07057	-1.38093	-1.43352	-1.33006	-1.23927
S76	0.6003	0.9157	1.82405	2.08963	3.12624	3.74859	1.17654	-0.13238	-0.04034	-0.01525	0.01413	-0.08337	-0.0604	0.05095	0.13412
S77	-0.05985	-0.23885	-0.25005	-0.31926	-0.09058	-0.10237	-0.3788	-0.60807	-0.58378	-0.54669	-0.71377	-0.93605	-0.98378	-0.91715	-0.83837
S78	1.9171	0.34781	0.75034	0.79779	0.64324	0.30181	-0.76986	0.48566	0.50557	0.52178	0.36766	-0.16631	-0.51364	-0.47443	-0.19359
S79	0.33621	0.53487	0.6695	0.58118	0.35013	0.34091	-0.38688	-0.14072	-0.00472	0.06572	0.19105	0.4196	0.05806	-0.3309	-0.39806
S80	0.88875	0.52412	1.24593	1.08285	0.95747	1.16241	0.04164	0.38223	0.35759	0.51781	0.58024	0.59135	0.70912	0.84667	0.79828

S81	-0.94068	-0.63696	-0.13484	-0.52775	-1.05485	-1.04754	-0.76986	0.64762	-0.53122	-0.74225	-0.93478	-1.16783	-1.23698	-1.15541	-1.06043
S82	1.35202	0.50881	0.88826	1.11225	1.08601	1.21014	1.08619	-0.57044	-0.27529	-0.35517	-0.48544	-0.79415	-0.60531	-0.19977	0.07568
S83	2.26091	1.16583	2.18397	2.55094	1.90285	1.97997	3.18765	-1.55637	-1.44363	-1.56941	-1.84577	-2.21029	-1.69065	-1.3461	-1.55826
S84	-1.4835	-1.18686	-0.79597	-0.79036	-0.93768	0.03058	0.11599	-1.55637	-1.44363	-1.56941	-1.84577	-2.21029	-2.29282	-2.16911	-2.00263
S85	4.67966	3.16341	4.02967	4.25629	4.55733	4.81392	2.56996	0.35112	0.78908	1.00895	1.24893	1.06766	0.92147	0.75354	0.85364
S86	-0.09176	-0.18397	-0.2081	-0.33833	-0.32392	-0.21603	-0.43013	-0.35678	-0.40859	-0.37909	-0.46106	-0.5901	-0.51304	-0.35962	-0.36093
S87	1.32741	0.62039	0.80293	0.82885	1.07324	0.7607	0.87373	0.17434	0.20746	0.37188	0.23446	-0.13356	-0.56263	-0.73179	-0.6367
S88	-0.02302	-0.64549	-0.59695	-0.40938	-0.32483	-0.28865	-0.76986	-0.50739	-0.48522	-0.41796	-0.57595	-0.86652	-1.02147	-0.84134	-0.76216
S89	-0.4884	-0.05253	-0.11769	-0.46932	-0.76232	-0.75827	-0.76986	0.08646	-0.295	-0.16576	-0.19019	-0.46793	-0.44316	-0.2042	-0.30881
S90	-0.16282	-0.74165	-0.26615	0.2643	-0.58701	-0.60513	-0.67083	0.24231	0.41179	-0.00047	-0.17781	-0.65783	-0.70529	-0.53952	-0.37554
S91	0.57199	0.60119	0.90935	0.23348	0.19369	0.04837	-0.76986	-0.07095	-0.05063	-0.06744	-0.07319	-0.2978	-0.35795	-0.33704	-0.24228
S92	0.22682	-0.34484	-0.33802	-0.09551	0.36972	0.2042	0.1436	-1.26432	-1.12239	-1.0845	-1.15661	-1.27078	-1.24638	-1.18223	-1.15088
S93	0.15424	0.05209	0.20648	-0.05259	-0.27915	-0.46412	-0.76103	-0.04409	-0.05769	-0.09897	-0.17924	-0.20552	-0.37906	-0.5264	-0.41811
S94	1.15566	0.67412	1.11975	1.25255	1.40243	1.17067	0.90167	-0.51721	-0.15565	-0.09737	-0.20783	-0.51919	-0.33417	0.00513	0.1533
S95	0.49897	-0.25272	-0.4053	-0.50551	-0.43057	-0.49201	-0.49225	-0.5744	-0.58808	-0.62402	-0.72447	-0.9146	-1.0239	-1.07168	-0.96695
S96	-0.49502	-0.12842	0.15766	-0.0064	-0.23584	-0.36915	-0.59112	0.01412	-0.80197	-0.97007	-1.01763	-0.96469	-1.20878	-1.38475	-1.39306
S97	-1.39373	-1.13202	-1.15013	-1.33947	-1.06891	-1.10761	-0.76986	-0.27778	-0.78967	-0.87182	-1.07336	-1.44723	-1.53505	-1.37422	-1.3543
S98	0.77132	-0.03462	0.03278	0.19933	0.44655	0.20086	-0.14741	-0.51798	-0.28774	-0.35559	-0.5407	-0.87776	-1.08947	-1.04151	-0.85449
S99	-1.52367	-1.24011	-1.47391	-1.37016	-1.06891	-1.10761	-0.76986	-0.51137	-0.67068	-0.75887	-1.03295	-1.36434	-1.59798	-1.56494	-1.44876
S100	0.39235	0.30472	0.16951	0.46043	0.40004	0.28757	-0.2934	-0.19944	-0.20566	-0.21236	-0.3173	-0.54912	-0.61822	-0.6171	-0.5193
S101	-0.08042	0.07165	0.07692	-0.14864	0.05151	0.01455	-0.35303	-0.14623	0.06158	0.0267	-0.07803	-0.2486	-0.41969	-0.42976	-0.39455
S102	-0.31666	-0.38898	-0.44184	-0.4784	-0.37461	-0.54232	-0.71423	-0.58681	-0.66588	-0.73294	-0.87752	-1.12057	-1.20382	-1.149	-1.03473
S103	0.94599	0.55652	0.8802	1.35459	0.83278	0.83526	-0.01655	0.24908	0.22485	0.16326	0.328	0.00661	0.15675	0.32748	0.28844
S104	0.21927	0.1492	0.3086	0.36949	0.0813	0.02482	-0.63442	-0.05253	-0.28764	-0.29529	-0.40298	-0.46197	-0.55688	-0.6704	-0.61726
S105	1.25937	0.61383	1.14624	1.14738	0.99172	0.53951	0.32728	-0.74586	-0.57161	-0.60231	-0.65871	-0.62079	-0.65315	-0.60192	-0.41286
S106	1.9096	0.5322	0.84535	0.94156	0.82944	0.46125	-0.58841	0.19278	0.11141	0.171	0.0652	-0.27568	-0.65146	-0.71588	-0.51656
S107	1.81325	1.03478	1.46549	1.4437	1.4991	1.42307	1.16824	-0.25176	0.06577	0.14101	-0.04128	-0.3248	-0.18815	0.09461	0.36397
S108	0.10518	-0.40105	-0.5091	-0.31074	-0.02261	-0.30678	-0.53452	-0.81504	-0.84123	-0.91269	-1.11934	-1.42063	-1.54057	-1.49235	-1.30078
S109	1.41065	0.81825	0.7735	0.70643	0.75116	0.40836	0.2035	-0.32202	-0.10007	-0.05859	-0.15147	-0.21031	-0.30752	-0.41751	-0.25708

Sample No.	integrated bins														
	0.84 .. 0.82	0.82 .. 0.80	0.80 .. 0.78	0.78 .. 0.76	0.76 .. 0.74	0.74 .. 0.72	0.72 .. 0.70	0.70 .. 0.68	0.68 .. 0.66	0.66 .. 0.64	0.64 .. 0.62	0.62 .. 0.60	0.60 .. 0.58	0.58 .. 0.56	0.56 .. 0.54
S1	1.90984	1.95703	2.02149	2.06178	2.04328	2.04084	2.09307	2.18264	2.19143	2.17841	2.11218	2.11984	2.07715	2.09337	2.03931
S2	-0.64043	-0.67282	-0.92116	-1.20366	-1.24687	-1.17867	-1.11974	-1.05615	-1.0003	-0.95691	-0.90794	-0.86156	-0.81691	-0.77025	-0.74747
S3	1.06317	0.99544	1.02451	1.14356	1.30493	1.36546	1.40552	1.44389	1.53459	1.55647	1.5988	1.67013	1.70892	1.73994	1.76701
S4	1.16513	1.20471	1.15718	1.12686	1.20911	1.22933	0.98921	0.88305	0.8	0.75821	0.73535	0.7036	0.66949	0.66682	0.70138
S5	0.46562	0.40427	0.46952	0.55213	0.62708	0.65035	0.6538	0.68526	0.68385	0.74666	0.75942	0.77992	0.81179	0.78881	0.8114
S6	-0.11029	-0.17128	-0.20087	-0.20474	-0.24899	-0.24067	-0.2265	-0.23087	-0.27822	-0.2983	-0.28567	-0.359	-0.3457	-0.39903	-0.39436
S7	-0.80478	-0.83795	-0.74689	-0.74789	-0.76103	-0.80748	-0.77604	-0.76446	-0.76279	-0.86882	-0.84356	-0.86156	-0.81691	-0.77025	-0.74747
S8	0.33614	0.33731	0.34207	0.38665	0.46842	0.49676	0.45088	0.46023	0.45824	0.5257	0.60548	0.68395	0.71307	0.71441	0.79113
S9	0.99154	1.18527	1.2546	1.14285	1.06635	0.9648	0.9758	1.04878	1.00514	0.94798	0.87664	0.81804	0.80388	0.71842	0.67815
S10	-0.66959	-0.62389	-1.07263	-1.33733	-1.24687	-1.17867	-1.11974	-1.05615	-1.0003	-0.95691	-0.90794	-0.86156	-0.81691	-0.77025	-0.74747
S11	-0.02981	-0.04767	0.14263	0.18973	0.19078	0.14759	0.12111	0.0144	-0.10412	-0.28726	-0.36978	-0.5256	-0.65236	-0.74038	-0.74747
S12	-0.76843	-0.6812	-0.76831	-0.71379	-0.6861	-0.62629	-0.60731	-0.61625	-0.58571	-0.60336	-0.5108	-0.4696	-0.47783	-0.46814	-0.50966

S13	-0.09671	-0.12129	-0.09652	-0.0482	-0.00747	0.00174	0.03933	0.12434	0.08987	0.13499	0.17041	0.24869	0.22467	0.22918	0.26156
S14	-0.78572	-0.90354	-0.84904	-0.85162	-0.87865	-0.91014	-0.86785	-0.90573	-0.91126	-0.95691	-0.90794	-0.86156	-0.81691	-0.77025	-0.74747
S15	1.02723	0.91645	0.90169	0.92118	0.96291	1.05324	1.03215	1.06522	1.06002	1.17245	1.24685	1.30009	1.27918	1.42637	1.46152
S16	-0.15653	-0.148	-0.30381	-0.31916	-0.33092	-0.3065	-0.30037	-0.38858	-0.3563	-0.36942	-0.38497	-0.35836	-0.36122	-0.42558	-0.46539
S17	1.39877	1.54746	1.45659	1.3569	1.34926	1.3565	1.347	1.26238	1.28147	1.30074	1.27077	1.24016	1.26975	1.35062	1.25368
S18	0.84825	0.80032	0.52041	0.16936	0.05741	-0.05276	-0.08302	-0.20188	-0.27952	-0.29307	-0.32897	-0.42885	-0.42898	-0.53503	-0.5355
S19	0.10797	0.20834	0.25836	-0.48635	-0.7685	-0.94176	-1.06875	-1.05615	-1.0003	-0.95691	-0.90794	-0.86156	-0.81691	-0.77025	-0.74747
S20	0.98454	0.98876	0.84228	0.69527	0.65555	0.6606	0.58978	0.597	0.56393	0.60294	0.5488	0.63662	0.70294	0.73823	0.77706
S21	1.53005	1.23324	1.69394	2.11483	2.35549	2.53391	2.57103	2.69773	2.80635	2.9931	3.12691	3.283	3.21952	3.46948	3.51304
S22	-1.18999	-1.19731	-1.13339	-1.12624	-1.14897	-1.17551	-1.11974	-1.05615	-1.0003	-0.95691	-0.90794	-0.86156	-0.81691	-0.77025	-0.74747
S23	-0.62509	-0.50753	-0.32309	-0.4582	-0.54913	-0.60937	-0.58785	-0.5525	-0.5445	-0.57498	-0.63505	-0.59817	-0.59954	-0.6069	-0.62003
S24	-0.36896	-0.42651	-0.27864	-0.22666	-0.19138	-0.18407	-0.1179	-0.09315	-0.03723	-0.11235	-0.13723	-0.16995	-0.12909	-0.25413	-0.25536
S25	-0.51524	-0.65833	-0.89864	-1.1277	-1.21981	-1.17867	-1.11974	-1.05615	-1.0003	-0.95691	-0.90794	-0.86156	-0.81691	-0.77025	-0.74747
S26	-0.21033	-0.26528	-0.47031	-0.5775	-0.58525	-0.62828	-0.42898	-0.41849	-0.50799	-0.63982	-0.66855	-0.66805	-0.75134	-0.77025	-0.74747
S27	0.28211	0.16409	0.14113	0.12342	0.14279	0.14806	0.14429	0.09901	0.15077	0.16288	0.20049	0.25531	0.25657	0.28272	0.27929
S28	1.35403	1.26618	1.32327	1.39022	1.44361	1.45718	1.60721	1.72995	1.79141	1.79207	1.73757	1.86306	1.92075	1.88737	1.89442
S29	1.99281	2.26642	2.32278	2.24065	2.13939	2.08224	2.09981	2.06996	2.17076	2.16785	2.21559	2.27433	2.32628	2.34386	2.29726
S30	1.97089	1.99365	1.46823	1.2033	1.15397	1.11642	0.89822	0.71788	0.6495	0.57295	0.51097	0.49837	0.46365	0.40733	0.39257
S31	-0.52702	-0.73498	-0.5936	-0.52162	-0.443	-0.41263	-0.38481	-0.37696	-0.39655	-0.43869	-0.43253	-0.41103	-0.42216	-0.41626	-0.46371
S32	4.49903	4.53185	4.53651	4.60208	4.58591	4.52591	4.5335	4.46197	4.43911	4.42458	4.43393	4.42573	4.47044	4.2801	4.1985
S33	-0.71766	-0.70772	-0.60335	-0.51525	-0.44643	-0.40374	-0.38967	-0.35386	-0.3403	-0.33764	-0.29494	-0.2653	-0.32488	-0.2195	-0.30213
S34	-0.40622	-0.49056	-0.5619	-0.59696	-0.58891	-0.59339	-0.58635	-0.62058	-0.64058	-0.59701	-0.64123	-0.59209	-0.57581	-0.62978	-0.70407
S35	0.37368	0.39003	0.15725	-0.10152	-0.17685	-0.23921	-0.33467	-0.40219	-0.45075	-0.50445	-0.58098	-0.6437	-0.58945	-0.73453	-0.74747
S36	0.26785	0.28507	0.25846	0.18041	0.12563	0.06059	0.10908	0.17844	0.08285	0.0488	0.00049	-0.07277	-0.06962	-0.16797	-0.21016
S37	0.61666	0.39448	-0.19027	-0.6021	-0.89855	-1.03151	-1.11974	-1.05615	-1.0003	-0.95691	-0.90794	-0.86156	-0.81691	-0.77025	-0.74747
S38	-1.20061	-1.30806	-1.49059	-1.33733	-1.24687	-1.17867	-1.11974	-1.05615	-1.0003	-0.95691	-0.90794	-0.86156	-0.81691	-0.77025	-0.74747
S39	-1.26556	-1.21769	-1.03744	-0.91137	-0.85939	-0.7828	-0.77858	-0.74345	-0.68811	-0.65622	-0.69204	-0.67061	-0.68827	-0.58645	-0.52352
S40	0.06919	-0.0374	0.06086	-0.03504	-0.08856	-0.08159	-0.15592	-0.25087	-0.30355	-0.34565	-0.35914	-0.45304	-0.48528	-0.5205	-0.57868
S41	-0.40535	-0.44542	-0.51372	-0.6919	-0.81751	-0.87517	-0.87179	-0.95673	-0.91519	-0.95691	-0.90794	-0.86156	-0.81691	-0.77025	-0.74747
S42	-0.1611	-0.24485	-0.41884	-0.57862	-0.65846	-0.70241	-0.75094	-0.79441	-0.81632	-0.84093	-0.84443	-0.82437	-0.81691	-0.77025	-0.74747
S43	-0.72871	-0.73673	-0.61081	-0.44532	-0.38274	-0.35416	-0.33496	-0.33647	-0.37721	-0.27968	-0.28819	-0.25213	-0.28858	-0.26573	-0.26683
S44	-0.82241	-0.89971	-0.81587	-0.72322	-0.67564	-0.66456	-0.65086	-0.60841	-0.58881	-0.61875	-0.62612	-0.57071	-0.63229	-0.57792	-0.64913
S45	-1.54398	-1.53093	-1.50438	-1.33733	-1.24687	-1.17867	-1.11974	-1.05615	-1.0003	-0.95691	-0.90794	-0.86156	-0.81691	-0.77025	-0.74747
S46	-0.39633	-0.37928	-0.48736	-0.62012	-0.66793	-0.73898	-0.74965	-0.81852	-0.79354	-0.88858	-0.80918	-0.86156	-0.81691	-0.77025	-0.74747
S47	0.00986	-0.01034	-0.05465	-0.10294	-0.1959	-0.23872	-0.21912	-0.23079	-0.2701	-0.2545	-0.29909	-0.28049	-0.32422	-0.42111	-0.41353
S48	0.39575	0.4223	0.38379	0.36503	0.32415	0.2899	0.30713	0.33175	0.28402	0.29107	0.34526	0.29189	0.29589	0.27202	0.33353
S49	0.2244	0.20165	0.23784	0.20075	0.222	0.24958	0.27626	0.26725	0.32251	0.33349	0.35071	0.38927	0.44795	0.45942	0.45312
S50	0.34679	0.35745	0.66166	0.71628	0.68428	0.6674	0.67888	0.75355	0.7256	0.66125	0.57067	0.62792	0.5728	0.50601	0.3799
S51	0.67552	0.51173	0.26127	0.16851	0.1151	0.10517	0.03941	0.02822	0.0009	-0.03526	-0.0337	-0.05918	-0.08825	-0.13077	-0.09753
S52	2.3388	2.16386	2.0076	2.07746	2.05967	1.95668	2.17918	2.30558	2.30094	1.99703	1.80028	1.84973	1.66946	1.40303	1.17658
S53	0.17169	0.14482	0.06632	0.0213	0.01779	0.00525	0.02206	-0.00913	0.0133	-0.04676	-0.09551	-0.07054	-0.05852	-0.03093	-0.1957
S54	0.11633	0.04436	-0.0306	-0.08058	-0.06541	-0.10255	-0.0595	-0.09375	-0.11671	-0.09921	-0.12013	-0.12838	-0.18874	-0.20104	-0.22745
S55	0.65174	0.68925	0.51877	0.41011	0.41361	0.31958	0.33758	0.21979	0.17396	0.12733	0.04611	-0.06867	-0.10392	-0.19156	-0.28686
S56	0.08335	-0.01402	-0.28412	-0.50417	-0.66873	-0.73494	-0.76517	-0.86683	-0.91327	-0.95691	-0.90794	-0.86156	-0.81691	-0.77025	-0.74747

S57	0.40956	0.35925	0.23985	0.24349	0.26041	0.26337	0.27554	0.23969	0.23933	0.26754	0.19003	0.2517	0.23392	0.19124	0.17485
S58	-0.32351	-0.33789	-0.24297	-0.0908	-0.02985	0.06117	0.05148	0.07081	0.10964	0.12835	0.18264	0.27027	0.31391	0.29224	0.3343
S59	-0.2794	-0.30849	-0.29134	-0.35599	-0.41068	-0.42865	-0.39423	-0.43942	-0.48365	-0.60608	-0.61611	-0.63216	-0.71775	-0.76446	-0.74747
S60	-0.93084	-1.25088	-1.50438	-1.33733	-1.24687	-1.17867	-1.11974	-1.05615	-1.0003	-0.95691	-0.90794	-0.86156	-0.81691	-0.77025	-0.74747
S61	1.71256	1.70733	1.81268	1.90504	1.91241	1.95558	2.05702	2.08198	2.17614	2.04967	2.02284	2.14138	2.19012	2.03134	2.0097
S62	0.40576	0.24746	0.42139	0.67696	0.77483	0.90725	0.85988	0.86656	0.88716	0.93839	0.95787	0.99762	1.04241	1.08705	1.10745
S63	0.21515	0.01488	-0.32102	-0.42989	-0.47429	-0.42737	-0.50813	-0.64144	-0.68235	-0.74255	-0.79467	-0.77054	-0.81262	-0.77025	-0.74747
S64	1.75604	1.8392	1.46263	1.07002	0.86582	0.67915	0.60423	0.35558	0.22293	0.1032	-0.04488	-0.07177	-0.09384	-0.27265	-0.35263
S65	1.97628	1.67349	1.97586	2.19784	2.32419	2.44002	2.54218	2.61765	2.65762	2.63933	2.68271	2.71652	2.70736	2.80774	2.74856
S66	-0.81104	-0.93001	-1.08108	-1.24735	-1.24687	-1.17867	-1.11974	-1.05615	-1.0003	-0.95691	-0.90794	-0.86156	-0.81691	-0.77025	-0.74747
S67	1.0628	0.85583	1.05449	1.30917	1.48336	1.61692	1.53694	1.50907	1.53987	1.62532	1.6898	1.69144	1.70312	1.87567	2.13529
S68	0.16847	0.1366	-0.04786	-0.17577	-0.20924	-0.20885	-0.27706	-0.30313	-0.30656	-0.33424	-0.31807	-0.36514	-0.39987	-0.39255	-0.38043
S69	1.1433	1.15935	0.64275	0.47936	0.40189	0.2838	0.18777	0.17142	0.08846	0.05349	0.04047	-0.02789	-0.04445	-0.11348	-0.13347
S70	-0.84302	-0.74457	-0.72069	-0.68768	-0.7067	-0.66451	-0.65547	-0.6408	-0.64256	-0.67337	-0.68174	-0.6542	-0.68779	-0.68598	-0.62602
S71	-0.1555	0.02399	-0.11391	-0.12764	-0.15075	-0.13675	-0.15354	-0.14844	-0.18392	-0.17776	-0.16122	-0.19001	-0.1627	-0.18141	-0.16135
S72	0.65326	0.84456	0.85485	0.76847	0.7602	0.71031	0.65643	0.54781	0.58837	0.58909	0.60121	0.5937	0.44062	0.55398	0.50744
S73	1.60858	1.77671	2.47896	2.29931	1.91935	1.97697	2.03565	1.99465	1.94772	2.00284	2.08485	2.03012	2.06804	2.11943	2.24595
S74	-1.2245	-1.11683	-1.50438	-1.33733	-1.24687	-1.17867	-1.11974	-1.05615	-1.0003	-0.95691	-0.90794	-0.86156	-0.81691	-0.77025	-0.74747
S75	-1.24248	-1.14414	-1.01542	-0.95848	-0.9126	-0.96823	-0.9426	-0.95073	-0.92033	-0.95691	-0.90794	-0.86156	-0.81691	-0.77025	-0.74747
S76	0.06274	0.11436	0.19462	0.19146	0.07501	-0.02079	-0.10857	-0.14719	-0.13605	-0.17585	-0.26477	-0.3374	-0.40762	-0.39655	-0.36565
S77	-0.82088	-0.68359	-0.62075	-0.4798	-0.47388	-0.42981	-0.47477	-0.43177	-0.41189	-0.41388	-0.4254	-0.38972	-0.40379	-0.39454	-0.35141
S78	-0.14399	-0.12944	0.11369	0.35535	0.37682	0.47533	0.37765	0.4737	0.47484	0.55452	0.54411	0.45324	0.50628	0.52684	0.47552
S79	-0.35476	-0.42348	-0.57899	-0.59554	-0.59102	-0.60548	-0.63269	-0.60599	-0.65708	-0.63708	-0.69329	-0.73142	-0.77108	-0.74679	-0.74747
S80	0.7869	0.82471	0.73656	0.63287	0.57391	0.55434	0.39143	0.33554	0.24092	0.2325	0.19941	0.04105	-0.03346	-0.05378	0.04323
S81	-1.08775	-1.07301	-0.95399	-0.9633	-1.01368	-1.04799	-1.11486	-1.05615	-1.0003	-0.95691	-0.90794	-0.86156	-0.81691	-0.77025	-0.74747
S82	0.13524	0.45964	0.7637	1.00615	1.17745	1.24457	1.24132	1.35041	1.38922	1.4901	1.63789	1.55369	1.61128	1.67925	1.76067
S83	-1.891	-1.66139	-1.50438	-1.33733	-1.24687	-1.17867	-1.11974	-1.05615	-1.0003	-0.95691	-0.90794	-0.86156	-0.81691	-0.77025	-0.74747
S84	-2.03729	-1.96049	-1.50438	-1.33733	-1.24687	-1.17867	-1.11974	-1.05615	-1.0003	-0.95691	-0.90794	-0.86156	-0.81691	-0.77025	-0.74747
S85	1.05547	1.26621	1.22454	1.50837	1.46572	1.36163	1.37659	1.44283	1.33857	1.39587	1.26664	0.96119	0.92511	0.85425	0.79399
S86	-0.38621	-0.2651	-0.32088	-0.31926	-0.38353	-0.41018	-0.42383	-0.41767	-0.49697	-0.51694	-0.48468	-0.49395	-0.55251	-0.46151	-0.5474
S87	-0.64042	-0.64176	-0.38501	-0.19698	-0.08918	-0.02566	0.01551	0.02237	0.05326	0.08114	0.12053	0.08244	0.08585	0.07789	0.10486
S88	-0.70011	-0.56433	-0.54164	-0.54781	-0.54382	-0.51131	-0.57674	-0.61428	-0.61272	-0.65525	-0.62545	-0.67141	-0.67084	-0.71929	-0.70747
S89	-0.1975	-0.05611	-0.47768	-0.56254	-0.5917	-0.65871	-0.64846	-0.68064	-0.66405	-0.70507	-0.69038	-0.72301	-0.71093	-0.743	-0.69134
S90	-0.5002	-0.43083	-0.0281	-0.1672	-0.15235	-0.15803	-0.17957	-0.20308	-0.17897	-0.17171	-0.20642	-0.22619	-0.26023	-0.22583	-0.20099
S91	-0.20127	-0.12251	-0.03766	0.05713	0.12445	0.13113	0.05233	-0.0581	-0.06502	-0.15201	-0.33327	-0.41851	-0.55489	-0.54731	-0.62463
S92	-1.09398	-1.03058	-1.08629	-1.02181	-1.0238	-1.04089	-1.05507	-1.00114	-1.0003	-0.95691	-0.90794	-0.86156	-0.81691	-0.77025	-0.74747
S93	-0.36459	-0.47787	-0.34666	-0.30538	-0.27669	-0.26071	-0.27906	-0.27271	-0.29271	-0.21675	-0.29605	-0.22605	-0.24882	-0.31954	-0.30755
S94	0.34492	0.60138	0.61056	0.70755	0.76974	0.71811	0.7306	0.81408	0.79437	0.86687	0.95942	0.9299	0.97461	0.99872	1.0185
S95	-0.93394	-0.89433	-0.86254	-0.79133	-0.73809	-0.70835	-0.70908	-0.71294	-0.70054	-0.66083	-0.64608	-0.67716	-0.68516	-0.60245	-0.67073
S96	-1.36143	-1.41086	-1.30556	-1.28012	-1.24687	-1.17867	-1.11974	-1.05615	-1.0003	-0.95691	-0.90794	-0.86156	-0.81691	-0.77025	-0.74747
S97	-1.33104	-1.17744	-1.11484	-1.04143	-1.04831	-1.0016	-1.02047	-1.0129	-1.0003	-0.95691	-0.90794	-0.86156	-0.81691	-0.77025	-0.74747
S98	-0.79289	-0.80804	-0.54762	-0.36616	-0.23631	-0.19419	-0.17529	-0.10107	-0.03241	0.04704	0.06636	0.07566	0.16421	0.19219	0.29271
S99	-1.41897	-1.35354	-1.12579	-0.97352	-0.92784	-0.90419	-0.86731	-0.84551	-0.84191	-0.80592	-0.82981	-0.79808	-0.80774	-0.77025	-0.70406
S100	-0.56963	-0.59526	-0.40093	-0.3015	-0.29047	-0.249	-0.25645	-0.22997	-0.24197	-0.23349	-0.23774	-0.32906	-0.25526	-0.33981	-0.3069

S101	-0.42364	-0.36826	-0.2379	-0.12527	-0.02785	-0.04829	-0.02177	-0.02492	-0.02748	0.03519	-0.00142	-0.04468	0.02174	-0.03455	-0.00651
S102	-1.0143	-0.97716	-0.823	-0.71644	-0.69025	-0.68191	-0.6474	-0.63634	-0.6627	-0.64028	-0.61268	-0.639	-0.5859	-0.57371	-0.63739
S103	0.20483	0.26725	0.07898	0.00795	-0.00601	-0.03097	-0.12272	-0.13002	-0.20884	-0.13081	-0.1461	-0.15408	-0.24517	-0.23495	-0.38604
S104	-0.67953	-0.70095	-0.56917	-0.52189	-0.50178	-0.51194	-0.48257	-0.50492	-0.5646	-0.52947	-0.59921	-0.57653	-0.63062	-0.68917	-0.73013
S105	-0.19941	-0.38807	-0.26668	-0.29381	-0.31016	-0.42059	-0.47257	-0.50221	-0.53427	-0.52281	-0.64477	-0.60012	-0.72825	-0.77025	-0.74747
S106	-0.48043	-0.49157	-0.29291	-0.1397	-0.16273	-0.15517	-0.17057	-0.12234	-0.15717	-0.09043	-0.14697	-0.28965	-0.2918	-0.2694	-0.27888
S107	0.40131	0.54455	0.91586	1.12735	1.26225	1.35397	1.34493	1.38975	1.41145	1.4263	1.56597	1.46907	1.51372	1.62731	1.64435
S108	-1.3242	-1.32581	-0.98781	-0.8363	-0.8785	-0.86429	-0.89273	-0.85873	-0.90905	-0.91248	-0.90794	-0.86156	-0.81691	-0.77025	-0.74747
S109	-0.15941	-0.19458	0.01359	0.13216	0.15959	0.20753	0.19229	0.13568	0.14788	0.1527	0.16507	0.08255	0.0766	0.07224	0.0866

Sample No.	integrated bins	
	0.54 .. 0.52	0.52 .. 0.50
S1	2.06955	2.09131
S2	-0.71163	-0.66332
S3	1.7267	1.81586
S4	0.56339	0.36459
S5	0.83786	0.79908
S6	-0.45348	-0.46847
S7	-0.71163	-0.66332
S8	0.74773	0.7585
S9	0.63422	0.55388
S10	-0.71163	-0.66332
S11	-0.71163	-0.66332
S12	-0.45799	-0.48412
S13	0.26386	0.34563
S14	-0.71163	-0.66332
S15	1.46645	1.4112
S16	-0.54186	-0.55734
S17	1.2172	1.06519
S18	-0.64026	-0.66332
S19	-0.71163	-0.66332
S20	0.69434	0.74182
S21	3.53585	3.5846
S22	-0.71163	-0.66332
S23	-0.64835	-0.61419
S24	-0.29821	-0.29581
S25	-0.71163	-0.66332
S26	-0.71163	-0.66332
S27	0.23797	0.27672
S28	1.94894	2.02068
S29	2.3351	2.36311
S30	0.19973	0.05161
S31	-0.54725	-0.48342
S32	4.17979	4.13457

S33	-0.33505	-0.33582
S34	-0.62351	-0.66332
S35	-0.71163	-0.66332
S36	-0.21789	-0.28874
S37	-0.71163	-0.66332
S38	-0.71163	-0.66332
S39	-0.59071	-0.57702
S40	-0.67203	-0.66332
S41	-0.71163	-0.66332
S42	-0.71163	-0.66332
S43	-0.23658	-0.24826
S44	-0.56531	-0.60664
S45	-0.71163	-0.66332
S46	-0.71163	-0.66332
S47	-0.53041	-0.47789
S48	0.26814	0.20283
S49	0.44471	0.39962
S50	0.39113	0.3544
S51	-0.19236	-0.28291
S52	1.20395	1.06738
S53	-0.12807	-0.16381
S54	-0.26379	-0.28691
S55	-0.39258	-0.48657
S56	-0.71163	-0.66332
S57	0.15357	0.01773
S58	0.28082	0.2884
S59	-0.71163	-0.66332
S60	-0.71163	-0.66332
S61	2.05382	1.95268
S62	1.04662	0.96277
S63	-0.71163	-0.66332
S64	-0.42369	-0.63211
S65	2.82416	2.8664
S66	-0.71163	-0.66332
S67	1.84357	1.83904
S68	-0.45959	-0.47228
S69	-0.11583	-0.29506
S70	-0.64931	-0.66332
S71	-0.05997	-0.1577
S72	0.64614	0.53379
S73	2.69722	3.05412
S74	-0.71163	-0.66332
S75	-0.71163	-0.66332
S76	-0.3853	-0.49103

S77	-0.38525	-0.35997
S78	0.53873	0.54498
S79	-0.71163	-0.66332
S80	-0.01821	-0.12867
S81	-0.71163	-0.66332
S82	1.72539	1.79689
S83	-0.71163	-0.66332
S84	-0.71163	-0.66332
S85	0.73878	0.73979
S86	-0.50606	-0.47069
S87	0.12452	0.1954
S88	-0.71163	-0.66332
S89	-0.69298	-0.66332
S90	-0.21885	-0.2174
S91	-0.68429	-0.66332
S92	-0.71163	-0.66332
S93	-0.31531	-0.40283
S94	0.98025	1.02664
S95	-0.63639	-0.64238
S96	-0.71163	-0.66332
S97	-0.71163	-0.66332
S98	0.25797	0.32925
S99	-0.71163	-0.66332
S100	-0.31898	-0.32821
S101	-0.04163	-0.07603
S102	-0.59775	-0.64374
S103	-0.38989	-0.2552
S104	-0.64519	-0.66332
S105	-0.71163	-0.66332
S106	-0.39868	-0.32393
S107	1.62118	1.58755
S108	-0.71163	-0.66332
S109	0.10378	0.02484

Table S2. The example 1H-NMR spectrum of APS

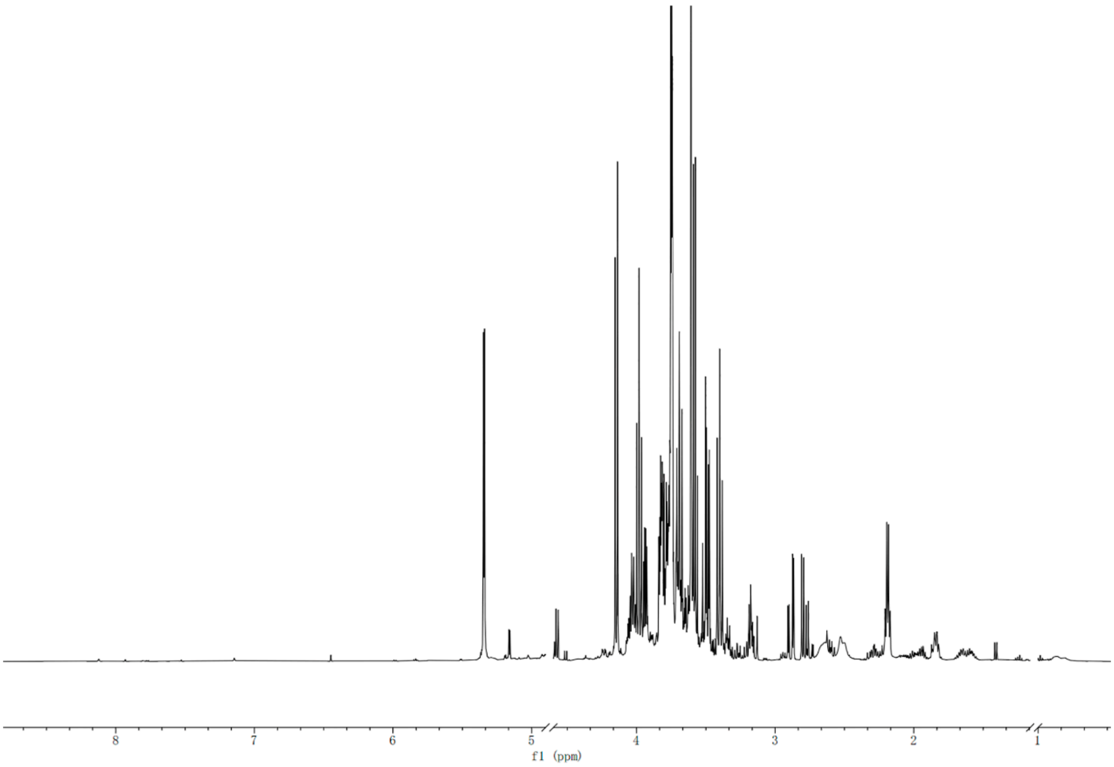

Table S3. Canonical Discriminant Function Coefficients of LDA models of growth patterns.

	Function	
	Cultivated	Wild-simulated
5.38 .. 5.36	-2.099	3.311
5.34 .. 5.32	-3.358	5.833
4.08 .. 4.06	-2.803	4.766
3.40 .. 3.38	1.631	-2.206
3.14 .. 3.12	-3.129	4.498
2.86 .. 2.84	1.440	-2.051
2.60 .. 2.58	4.125	-6.293
2.34 .. 2.32	-3.759	5.921
1.24 .. 1.22	-1.634	2.531
(Constant)	-4.057	-9.090

Table S4. Regression Coefficients of OPLS-DA models of growth patterns.

	Function	
	Cultivated	Wild-simulated
7.34 .. 7.32	0.032579	-0.032579
7.32 .. 7.30	0.034415	-0.034415
7.20 .. 7.18	0.0089417	-0.0089417
5.34 .. 5.32	-0.0398662	0.0398662
5.32 .. 5.30	0.0410606	-0.0410606
4.28 .. 4.26	-0.0749239	0.0749239

4.26 .. 4.24	-0.108533	0.108533
4.06 .. 4.04	0.0427325	-0.0427325
4.04 .. 4.02	0.044534	-0.044534
3.92 .. 3.90	-0.0126162	0.0126162
3.90 .. 3.88	-0.00674484	0.00674484
3.88 .. 3.86	0.0739816	-0.0739816
3.82 .. 3.80	-0.00263453	0.00263453
3.80 .. 3.78	-0.0103503	0.0103503
3.58 .. 3.56	-0.0350788	0.0350788
3.56 .. 3.54	0.041288	-0.041288
3.44 .. 3.42	0.0613441	-0.0613441
3.42 .. 3.40	0.0379698	-0.0379698
3.20 .. 3.18	0.0734642	-0.0734642
3.18 .. 3.16	0.0416285	-0.0416285
3.10 .. 3.08	-0.114896	0.114896
2.96 .. 2.94	0.0330623	-0.0330623
2.94 .. 2.92	0.0289275	-0.0289275
2.92 .. 2.90	-0.0227412	0.0227412
2.90 .. 2.88	0.0162392	-0.0162392
2.84 .. 2.82	0.0144031	-0.0144031
2.82 .. 2.80	0.00504036	-0.00504036
2.80 .. 2.78	0.00938344	-0.00938344
2.78 .. 2.76	0.015231	-0.015231
2.76 .. 2.74	0.0568908	-0.0568908
2.74 .. 2.72	0.0457756	-0.0457756
2.72 .. 2.70	0.0459584	-0.0459584
2.70 .. 2.68	0.0341996	-0.0341996
2.58 .. 2.56	0.0441252	-0.0441252
2.56 .. 2.54	0.0452316	-0.0452316
2.54 .. 2.52	0.0684092	-0.0684092
2.38 .. 2.36	-0.0218819	0.0218819
2.36 .. 2.34	-0.133024	0.133024
2.34 .. 2.32	-0.175489	0.175489
2.22 .. 2.20	0.0426987	-0.0426987
1.90 .. 1.88	-0.0170122	0.0170122
1.88 .. 1.86	0.00676424	-0.00676424
1.86 .. 1.84	0.0107262	-0.0107262
1.84 .. 1.82	-0.00884152	0.00884152
1.72 .. 1.70	-0.0723588	0.0723588
1.70 .. 1.68	-0.0336621	0.0336621
1.68 .. 1.66	-0.00168578	0.00168578
1.66 .. 1.64	-0.00598443	0.00598443
1.64 .. 1.62	-0.0113396	0.0113396
1.62 .. 1.60	-0.00777083	0.00777083
1.60 .. 1.58	-0.00912115	0.00912115
1.58 .. 1.56	-0.0230815	0.0230815
0.90 .. 0.88	0.0216613	-0.0216613
(Constant)	1.23724	0.79733

Table S5. Regression Coefficients of OPLS-DA models of growth years over and within 5 years.

	Function	
	5 years and less	More than 5 years
7.94 .. 7.	0.130183	-0.130183
7.90 .. 7.	-0.166088	0.166088
7.86 .. 7.	0.273277	-0.273277
7.80 .. 7.	0.155121	-0.155121
6.98 .. 6.	-0.330655	0.330655
6.48 .. 6.	-0.184206	0.184206
5.78 .. 5.	0.232068	-0.232068
5.58 .. 5.	0.12417	-0.12417
4.58 .. 4.	0.246388	-0.246388
3.94 .. 3.	-0.172869	0.172869
3.72 .. 3.	-0.23377	0.23377
2.66 .. 2.	-0.426228	0.426228
2.54 .. 2.	0.378059	-0.378059
1.90 .. 1.	0.479265	-0.479265
1.72 .. 1.	-0.446514	0.446514
1.58 .. 1.	0.100504	-0.100504
(Constant)	1.84095	0.535548

Table S6. Regression Coefficients of OPLS-DA models of growth years within 5 years.

	Function		
	2 years	3 years	4~5 years
8.28 .. 8.	0.0853063	-0.0412924	-0.0720749
8.24 .. 8.	0.426789	-0.547546	0.111674
7.92 .. 7.	0.363428	-0.32651	-0.0984709
7.84 .. 7.	-0.296126	0.312202	0.0163018
7.54 .. 7.	0.0293238	0.314178	-0.479606
7.52 .. 7.	-0.0405412	-0.180698	0.31172
7.24 .. 7.	0.305277	-0.320907	-0.018112
3.82 .. 3.	-0.0692086	0.789276	-0.988355
3.58 .. 3.	-0.0814826	-0.375798	0.643995
3.32 .. 3.	-0.269839	0.410977	-0.160345
3.30 .. 3.	0.501286	-0.411189	-0.190085
3.20 .. 3.	0.601536	-0.620319	-0.0523333
2.76 .. 2.	-0.0914339	0.134	-0.0470489
2.70 .. 2.	-0.505473	0.766064	-0.29511
2.42 .. 2.	0.289163	-0.248071	-0.0945804
2.40 .. 2.	0.0201528	0.0475844	-0.0964481
2.38 .. 2.	-0.587917	0.173989	0.649908
2.34 .. 2.	0.15401	-0.495671	0.453177
2.24 .. 2.	-0.447651	0.480484	0.012828
(Constant)	1.23272	0.609064	0.361551

Table S7. Canonical Discriminant Function Coefficients of LDA models of species.

	Function	
	MG	MJ
8.48 .. 8.46	0.611	-8.106
8.38 .. 8.36	-0.537	2.826
7.92 .. 7.90	-0.355	2.664
7.14 .. 7.12	0.373	-6.692
4.60 .. 4.58	0.332	-3.048
3.20 .. 3.18	-1.282	13.498
2.96 .. 2.94	-0.493	3.492
2.82 .. 2.80	0.679	-3.487
1.52 .. 1.50	0.495	-5.830
1.28 .. 1.26	-0.217	2.755
1.18 .. 1.16	0.492	-3.709
1.00 .. 0.98	0.063	-2.046
(Constant)	-0.897	-11.953

Table S8. Regression Coefficients of OPLS-DA models of species.

	Function	
	MG	MJ
7.96 .. 7.94	0.180534	-0.180534
7.92 .. 7.90	-0.272378	0.272378
7.18 .. 7.16	0.349831	-0.349831
7.14 .. 7.12	0.339908	-0.339908
4.48 .. 4.46	0.323125	-0.323125
3.96 .. 3.94	-0.307962	0.307962
3.24 .. 3.22	0.337305	-0.337305
3.20 .. 3.18	-0.537181	0.537181
2.96 .. 2.94	-0.711038	0.711038
2.82 .. 2.80	0.453328	-0.453328
2.12 .. 2.10	0.201836	-0.201836
1.52 .. 1.50	0.406824	-0.406824
(Constant)	2.22049	0.444097

Table S9. Canonical Discriminant Function Coefficients of LDA models of origins.

	Function		
	Inner Mongolia	Gansu	Shaanxi
7.98 .. 7.96	-3.179	-2.483	4.099
7.96 .. 7.94	6.522	1.536	-4.277
7.80 .. 7.78	2.554	4.236	-5.807
6.48 .. 6.46	-5.833	-6.718	9.587
5.18 .. 5.16	5.908	1.579	-5.300

4.58 .. 4.56	4.920	2.401	-6.235
3.98 .. 3.96	-5.319	-4.068	7.326
3.58 .. 3.56	-11.606	-10.285	17.069
3.32 .. 3.30	-4.034	-8.226	11.123
3.20 .. 3.18	-22.616	.907	11.074
2.66 .. 2.64	-1.600	-1.784	2.696
2.58 .. 2.56	11.021	3.745	-9.268
2.34 .. 2.32	-3.937	-4.931	7.943
1.86 .. 1.84	18.845	-1.610	-9.211
1.44 .. 1.42	-8.467	-2.245	6.328
0.52 .. 0.50	3.188	3.730	-5.694
(Constant)	-14.110	-9.197	-21.006

Table S10. Regression Coefficients of OPLS-DA models of origins.

	**Function**		
	Inner Mongolia	Gansu	Shaanxi
8.48 .. 8.46	-0.0457698	-0.003893	0.0380354
7.80 .. 7.78	-0.146809	0.264646	-0.140407
7.08 .. 7.06	-0.180426	0.0730166	0.0662689
6.48 .. 6.46	0.00587474	-0.185279	0.171025
4.94 .. 4.92	0.532202	-0.301292	-0.114124
4.60 .. 4.58	-0.0922391	0.330664	-0.243871
4.08 .. 4.06	-0.207812	-0.0732366	0.225303
4.06 .. 4.04	-0.248155	0.415362	-0.207056
3.54 .. 3.52	0.102659	-0.242174	0.152262
3.46 .. 3.44	0.0621863	-0.135057	0.0812114
3.44 .. 3.42	-0.166511	0.305826	-0.164612
3.00 .. 2.98	-0.381369	-0.0453532	0.329153
2.96 .. 2.94	0.692627	-0.309253	-0.226982
2.58 .. 2.56	0.91954	-0.387847	-0.322856
2.34 .. 2.32	0.161696	-0.411247	0.268045
(Constant)	0.447909	0.71002	0.974824
